# LukS-PV Induces Apoptosis *via* the SET8-H4K20me1-PIK3CB Axis in Human Acute Myeloid Leukemia Cells

**DOI:** 10.3389/fonc.2021.718791

**Published:** 2021-10-20

**Authors:** Liang Fei Xu, Lan Shi, Shan Shan Zhang, Peng Sheng Ding, Fan Ma, Kai Di Song, Ping Qiang, Wen Jiao Chang, Yuan Yuan Dai, Yi De Mei, Xiao Ling Ma

**Affiliations:** ^1^ The First Affiliated Hospital of USTC, Division of Life Sciences and Medicine, University of Science and Technology of China, Hefei, China; ^2^ University of Science and Technology of China, School of Life Sciences and Medicine, USTC Life Sciences, Hefei, China

**Keywords:** epigenetics, AML, LukS-PV, apoptosis, SET8

## Abstract

Evidence suggests that histone modification disorders are involved in leukemia pathogenesis. We previously reported that LukS-PV, a component of Panton–Valentine leukocidin (PVL), has antileukemia activities that can induce differentiation, increase apoptosis, and inhibit proliferation of acute myeloid leukemia (AML) cells. Furthermore, LukS-PV inhibited hepatoma progression by regulating histone deacetylation, speculating that LukS-PV may exert antileukemia activity by targeting histone modification regulators. In this study, the results showed that LukS-PV induced apoptosis by downregulating the methyltransferase SET8 and its target histone H4 monomethylated at Lys 20 (H4K20me1). Furthermore, chromatin immunoprecipitation sequencing and polymerase chain reaction identified the kinase *PIK3CB* as a downstream target gene for apoptosis mediated by SET8/H4K20me1. Finally, our results indicated that LukS-PV induced apoptosis *via* the PIK3CB-AKT-FOXO1 signaling pathway by targeting SET8. This study indicates that SET8 downregulation is one of the mechanisms by which LukS-PV induces apoptosis in AML cells, suggesting that SET8 may be a potential therapeutic target for AML. Furthermore, LukS-PV may be a drug candidate for the treatment of AML that targets epigenetic modifications.

## Introduction

Acute myeloid leukemia (AML) is a heterogeneous clonal disorder of hematopoietic progenitor cells, which is characterized by immature myeloid cell proliferation and bone marrow failure with a short course (1). AML occurs predominantly in older adults who are more than 60 years of age (2). Although hematopoietic stem cell transplantation combined with chemotherapy has substantially improved therapy for young adults, approximately 80% of older adults still succumb to the disease or related therapeutic toxicity. Thus, it is important to identify more targeted therapies for AML.

In recent years, bacterial toxins have received increasing attention as potential anticancer drugs because of their specificity and cytotoxicity, and bacterial toxin-containing anticancer drugs have entered clinical trials (3, 4). Panton–Valentine leukocidin (PVL) is a two-component pore-forming cytosolic toxin secreted by *Staphylococcus aureus*. It was first discovered by Van de Velde and isolated from hemolysin by Panton and Valentine in 1932 (5, 6). PVL is composed of LukF-PV and LukS-PV protein subunits encoded by *lukF-PV* and *lukS-PV* genes, respectively (7). PVL belongs to the pore-forming toxin family and has been reported to induce lysis of human polymorphonuclear neutrophils (8, 9). LukS-PV first binds to a specific receptor on the membrane of neutrophils, and LukF-PV binds to LukS-PV to form a dimer. The LukS-PV–LukF-PV dimers combine to form a ring structure, which is inserted into the cellular membrane and forms a planar vertical transmembrane pore that induces necrosis and apoptosis (10, 11). Our previous study demonstrated that the LukS-PV subunit alone did not cause perforation cytotoxicity; however, this subunit displayed antileukemia activity *in vitro* and *in vivo* without noticeable side effects in mice (12). Sun et al. reported that LukS-PV regulated microRNA-125a-3p-induced THP-1 cell differentiation and apoptosis by downregulating NF1 and BCL2 (13). Zhang et al. found that LukS-PV induced AML apoptosis by targeting the C5a receptor (14). Additionally, LukS-PV induced differentiation by activating the ERK signaling pathway and c-JUN/c-FOS in AML cells (15). The above investigations indicate that LukS-PV exerts antileukemia activity through several mechanisms and targets, and whether it has other mechanisms of action deserves further study.

Comprehensive genomic profiling of AML has shown that dysregulation of histone modifications plays an essential role in leukemia pathogenesis, and emerging evidence suggests that histone modification is a major epigenetic determinant for gene expression and is frequently dysregulated in AML (16). Moreover, histone modifications are potentially reversible, which provides opportunities for targeted therapy for AML. DOT1L methyltransferase inhibitors have been used extensively to reduce the leukemia burden in a variety of AML models with mixed lineage leukemia rearrangements (17). Tranylcypromine, an LSD1 inhibitor, either alone or in combination with all-trans retinoic acid, disrupted the oncogenic program of mixed lineage leukemia and induced expression of myeloid differentiation genes in AML cells with rearrangements (18). These studies suggest that histone modifications are potentially promising targets for leukemia therapy. Furthermore, in another study, we demonstrated that LukS-PV inhibited the proliferation and induced apoptosis in hepatocellular carcinoma (HCC) cells by downregulating histone acetylation (19), suggesting that LukS-PV may regulate histone epigenetic modifiers. However, whether LukS-PV exerts antileukemia activity by targeting regulators of histone modification remains unclear. Therefore, in this study, we investigated the underlying molecular mechanisms by which LukS-PV exerts antileukemia activities to determine whether this protein regulated histone modifications in AML cells.

## Materials and Methods

### Cell Culture and Reagents

Human acute leukemia cell lines HL-60 and NB4 were obtained from the Shanghai Institute for Biological Sciences (Shanghai, China). Cells were cultured in RPMI-1640 medium (Gibco, Grand Island, NY, USA) supplemented with 10% fetal bovine serum (HyClone, Logan, UT, USA) and 1% penicillin/streptomycin in an incubator at 37°C with 5% CO_2_. The medium was changed every 2–3 days. The PIK3CB inhibitor GSK2636771 and SET8 inhibitor UNC0379 were purchased from MedChemExpress (Shanghai, China).

### Total RNA Extraction of Peripheral Blood From Acute Myeloid Leukemia Patients and Healthy Individuals

AML patients were diagnosed in accordance with clinical and laboratory criteria, and healthy individuals with normal physical examination indices were used as controls. To extract total RNA, a fivefold volume of erythrocyte lysis buffer was added to fresh whole blood samples, which were placed on a shaker for 15–20 min. The cells were centrifuged for 5 min at 1,000 rpm, and the supernatant was discarded. The cell pellet was washed twice with phosphate-buffered saline (PBS), and the remaining erythrocytes were re-lysed. Total RNA was extracted using TRIzol (Invitrogen, Carlsbad, CA, USA) in accordance with the manufacturer’s instructions. Experiments using samples derived from AML patients were approved by the Ethics Committee and Institutional Review Board of University of Science and Technology of China, Anhui, China (approval number: 2019-N(H)-101).

### RNA Sequencing

Total RNA was isolated from HL-60 cells treated with LukS-PV or PBS. Paired-end libraries were synthesized using the TruSeq RNA Sample Preparation Kit (Illumina, San Diego, CA, USA) in accordance with the manufacturer’s instructions. Briefly, the mRNA molecules were purified using poly-T oligomers attached to magnetic beads. Library construction and sequencing were performed at Shanghai Sinomics Corporation of China.

### Separation and Culturing of Primary Bone Marrow Cells

AML patients were diagnosed in accordance with clinical and laboratory criteria. Primary AML cells were harvested from the bone marrow of AML patients immediately after lumbar puncture. Fresh bone marrow mononuclear cells were isolated by Ficoll density-gradient centrifugation, resuspended in RPMI-1640 medium supplemented with 10% FBS, and placed in an incubator. The primary AML cells were then incubated with LukS-PV for 24 h.

### Recombinant LukS-PV Production and Purification

The pET28a vector (Roche Diagnostics Corp., Basel, Switzerland) was used to generate six recombinant His-tagged LukS-PV proteins. The LukS-PV sequence was amplified from PVL-positive *S. aureus* isolates. PCR products were digested with *Xho*I and *Bam*HI (Promega Corp., Madison, WI, USA) and ligated into the pET28a vector. Recombinant LukS-PV was purified as previously described (20).

### RNA Isolation and Quantitative Real-Time RT-PCR

Total RNA was extracted using TRIzol (Invitrogen, Carlsbad, USA) as described above. Reverse transcription was performed using the RevertAid First Strand cDNA Synthesis Kit (Fermentas, Vilnius, Lithuania). All quantitative real-time PCR (qRT-pCR) assays were carried out using a StepOnePlus RT-PCR system (Applied Biosystems, Carlsbad, CA, USA). Relative expression levels were quantified using the comparative Ct method. Gene-specific primer sequences were as follows: *SET8*: 5′-ACTTACGGATTTCTACCCTGTC-3′ and 5′-CGATGAGGTCAATCTTCATTCC-3′; *PIK3CB*: 5′-ATCGCTCTGGCCTCATTGAAGTTG-3′ and 5′-ATGGCTCGGTCCAGGTCATCC-3′.

### Lentiviral Transduction

The lentiviral vectors used for SET8 silencing and overexpression and PIK3CB overexpression (HanBio, Shanghai, China) were transduced into HL-60 and NB4 cells. As controls, lentiviral vectors containing short hairpin RNA sequences targeting a non-mammalian gene were used. After 48 h of transduction, the cells were selected using puromycin and cultured.

### Flow Cytometric Analysis

To assess apoptosis, cells were harvested by centrifugation at 1,000 rpm for 5 min, washed twice with cold PBS, resuspended in 500 µl of staining buffer, and co-stained with Annexin V-PE and 7-AAD (eBioscience, San Diego, CA, USA) at room temperature for 15 min in the dark. The cells were analyzed using a FACSCalibur flow cytometer (BD Biosciences, Franklin Lakes, NJ, USA). The data were analyzed using FCS Express software (*De Novo* Software, Pasadena, CA, USA).

### Western Blotting

Cells were lysed in radioimmunoprecipitation assay (RIPA) lysis buffer containing 1% phenylmethylsulfonyl fluoride (Beyotime, Shanghai, China) on ice for 30–60 min and centrifuged at 12,000 rpm for 5 min, and the pellet was discarded. The protein samples were boiled in sodium dodecyl sulfate (SDS)-loading dye for 15 min. The proteins were separated by SDS–polyacrylamide gel electrophoresis (SDS-PAGE) and electro-transferred onto a 0.45-µm nitrocellulose membrane (Millipore, Bedford, MA, USA). The membranes were blocked with Protein Free Rapid Blocking Buffer (EpiZyme, Jiangsu, China) and subsequently probed with primary antibodies. The primary antibodies used were as follows: rabbit anti-human SET8 (#2996), anti-PIK3CB (#3011), anti-FOXO1 (#2880), anti-AKT (#4685), anti-p-AKT (#4060), and anti-BAK (#12105), anti-histone H4 (#13919), anti-BCL2 (#15071), and anti-GAPDH (#51332) purchased from Cell Signaling Technology (Beverly, MA, USA) and anti-H4K20me1 (Abcam; #ab177188; Cambridge, UK). Thereafter, the membranes were washed and incubated with the appropriate horseradish peroxidase-conjugated secondary antibody for 1.5 h at room temperature. Immunoreactive bands were visualized using an enhanced chemiluminescence detection system.

### Chromatin Immunoprecipitation Sequencing Assay and Chromatin Immunoprecipitation–PCR

Approximately 4 × 10^6^ HL-60 cells were fixed with 1% formaldehyde and subjected to chromatin immunoprecipitation (ChIP) with a ChIP grade anti-H4K20me1 antibody (Abcam; #ab177188) using the SimpleChIP enzymatic ChIP kit (Cell Signaling Technology, #9003) in accordance with the manufacturer’s instructions. Input and H4K20me1-immunoprecipitated chromatin samples were sequenced at GeneSky Biotechnologies, Inc. (Suzhou, China). The gene-specific primer sequences used for ChIP-PCR were as follows: *PIK3CB*: 5′-GGAAGAGCGGAATCTCTCGG-3′ and 5′-GCACGGCCTTTCCTAACTCT-3′. The PCR reaction program was as follows: initial denaturation at 95°C for 3 min followed by 40 cycles of denaturing at 95°C for 15 s and annealing/extension at 60°C for 60 s per cycle. The %Input = 2% * 2^(CT_Input sample_ − CT_IP sample_).

### Xenograft Mouse Assay

Male BALB/c nude mice (4 weeks old) were obtained from GemPharmatech, Ltd. (Nanjing, Jiangsu, China) and maintained in a specific pathogen-free facility at the Laboratory Animal Center of Anhui Medical University, and care was in accordance with institution guidelines. Mice were injected intraperitoneally (i.p.) with cyclophosphamide (100 mg/kg body weight) on each of three successive days to suppress immunity and then randomized into three groups: normal control (five mice), HL-60 group (10 mice), and NB4 group (10 mice). Mice in the normal control group received only PBS. Each mouse in the HL-60 and NB4 groups was injected with 5 × 10^6^ HL-60 and NB4 cells, respectively, *via* the tail vein and then randomized into PBS (five mice) and LukS-PV (five mice) groups. The LukS-PV mice were injected with LukS-PV (300 μg/kg body weight per mouse) *via* the tail vein for three successive days. After 30 days, mice were sacrificed, and their spleens and peripheral blood samples were collected for the next experiments. CD33, a myeloid lineage-specific antigen, is a sialoadhesin family member that is normally expressed on precursor myeloid cells and can be used as a specific marker to observe leukemic cell proliferation and infiltration in a mouse leukemia model (21). Hence, we used anti-CD33-PE (BD Biosciences, cat #555450) to assess invasion of AML cells *in vivo via* flow cytometry. This study was approved by the Ethics Committee and Institutional Review Board of University of Science and Technology of China, Anhui, China (approval number: 2019-N(H)-101), and all experiments conformed to the relevant regulatory standards.

### Statistical Analysis

All data are expressed as means ± standard deviations (SDs), and all experiments were performed in triplicate. All data met a normal distribution. Statistical analyses were performed using independent-sample t-tests for comparisons between two groups or ANOVA for multiple comparisons followed by Bonferroni’s or Dunn’s post-test to compare differences between the groups. The log-rank test was used for survival analysis. Sample sizes for all experiments were predetermined from our experience. Animals were randomly assigned, and no samples were excluded from the analyses. The investigators were not blinded to the team allocation at some stages in the draw materials and effect assessments. All statistical analyses were conducted using GraphPad Prism software (Version 5.0; GraphPad Software, Inc., San Diego, CA, USA). A p-value of p < 0.05 (*), p < 0.01 (**), or p < 0.001 (***) was considered statistically significant.

## Results

### LukS-PV Induced Cell Apoptosis *In Vitro* and Inhibited Cell Invasion *In Vivo*

**Figure 1:**
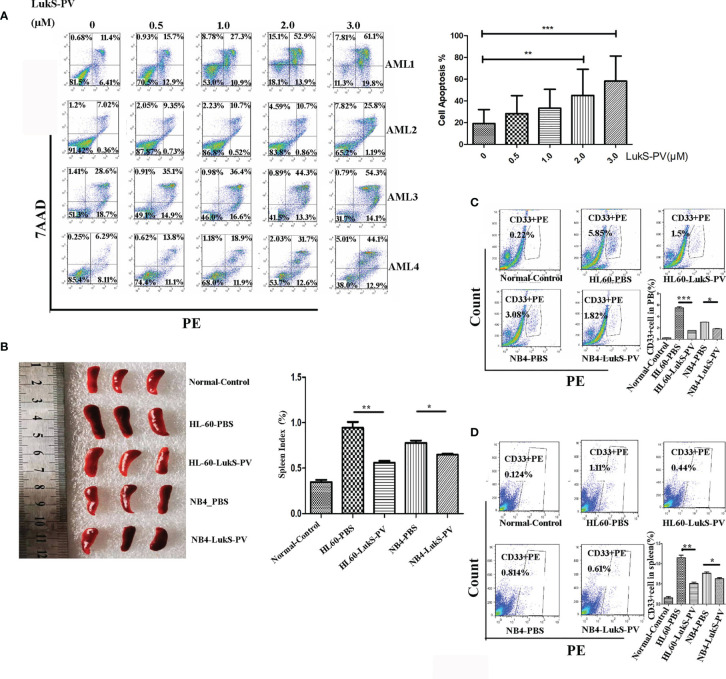
LukS-PV induces apoptosis *in vitro* and inhibits cell invasion *in vivo*. **(A)** Flow cytometric analysis shows that treatment of primary acute myeloid leukemia (AML) blasts with LukS-PV induces apoptosis in a dose-dependent manner. **(B)** The spleen volume (left) and spleen index [(spleen weight/body weight) × 100, right] of mice. **(C)** The percentage of CD33+ cells in PB (peripheral blood). **(D)** The percentage of CD33+ cells in the spleen. Data are expressed as mean ± SD (n = 3). ns, not significant; *p < 0.05; **p < 0.01; ***p < 0.001.

We randomly isolated bone marrow samples from four AML patients for *in vitro* culture and treated them with different concentrations of LukS-PV to detect apoptosis by flow cytometry. The demographics and clinical features of the four AML patients are described in **Table 1**. The results showed that LukS-PV induced apoptosis in a dose-dependent manner in primary AML blasts (**Figure 1A**). To further study the antileukemia activity of LukS-PV *in vivo*, we injected AML cell lines (HL-60 and NB4) into the tail vein of nude mice and treated the mice with LukS-PV. The results demonstrated that the spleen index for the LukS-PV treatment group was lower than that for the PBS control group (**Figure 1B**). Furthermore, flow cytometric analysis showed that the percentage of AML cells (CD33+ cells) in the peripheral blood and spleens was lower in the LukS-PV treatment group than in the control group (**Figures 1C, D**). These results indicated that Luks-PV induced AML apoptosis *in vitro* and inhibited tumor cell invasion *in vivo*.

**Table 1:** Clinical features of four AML patients.

	AML 1	AML 2	AML 3	AML 4
Age (years)	47	66	55	47
Gender	Female	Male	Female	Female
FAB	M2	M3	M4	M3
Mutation	AML1/ETO	PML/RARA	MLL/AF9	PML/RARA
Cytogenetic	46, XX, der(7)t(7;8)(p22;q22), t(8;21)(p22;q22)	46, XY, t(15;17)	46, XX, t(9;11)(p21;q23)	46,XX, del(13)t(15;17)
Treatment	IA+ARA-C	IA+ATRA	IA	ATRA+ATO

AML, acute myeloid leukemia.

### SET8 Is Downregulated in Acute Myeloid Leukemia Cells After LukS-PV Treatment

**Figure 2:**
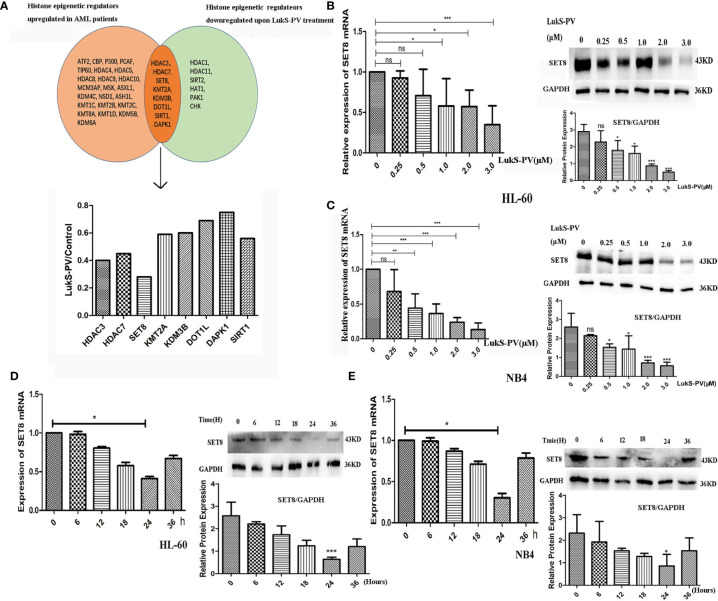
SET8 is downregulated in acute myeloid leukemia (AML) cells after LukS-PV treatment. **(A)** Downregulated histone epigenetic regulators after LukS-PV treatment. **(B)** HL-60 and **(C)** NB4 cells were treated with LukS-PV at different concentrations, and *SET8* mRNA and protein expression levels were determined by quantitative real-time PCR and Western blotting. **(D)** HL-60 and **(E)** NB4 cells were treated with 3.0 μM of LukS-PV at different timepoints, and *SET8* mRNA and protein expression levels were determined by quantitative real-time PCR and Western blotting. ns, not significant; *p < 0.05; **p < 0.01; ***p < 0.001.

Recent studies have revealed that dysregulation of histone modification plays an important role in leukemia pathogenesis. Several histone-modifying enzymes have been investigated as potential therapeutic targets for leukemia. We demonstrated that LukS-PV could inhibit the proliferation and induce apoptosis by downregulating histone acetylation in HCC cells. These studies suggested that LukS-PV may also exert antileukemia activity by targeting histone modification regulators. To determine whether LukS-PV induced apoptosis by regulating histone modification, we identified 31 highly expressed histone epigenetic modifiers in AML patients using The Cancer Genome Atlas (TCGA) database and Genotype-Tissue Expression (GTEx) datasets (22, 23). By RNA sequencing, we determined that LukS-PV downregulated 14 histone epigenetic regulating genes in AML cells. After overlap analysis, we identified a total of eight different histone modification regulators that were potential targets for LukS-PV. Because SET8 was decreased to the greatest extent among these potential targets, we chose SET8 for further evaluation (**Figure 2A**). We verified that both mRNA and protein levels of SET8 were downregulated by LukS-PV in AML cells in a dose- and time-dependent manner (**Figures 2B–E**). Collectively, these data demonstrated that LukS-PV decreased SET8 expression in AML cells.

### SET8 Is Highly Expressed in Acute Myeloid Leukemia and Is Associated With Poor Prognosis

**Figure 3:**
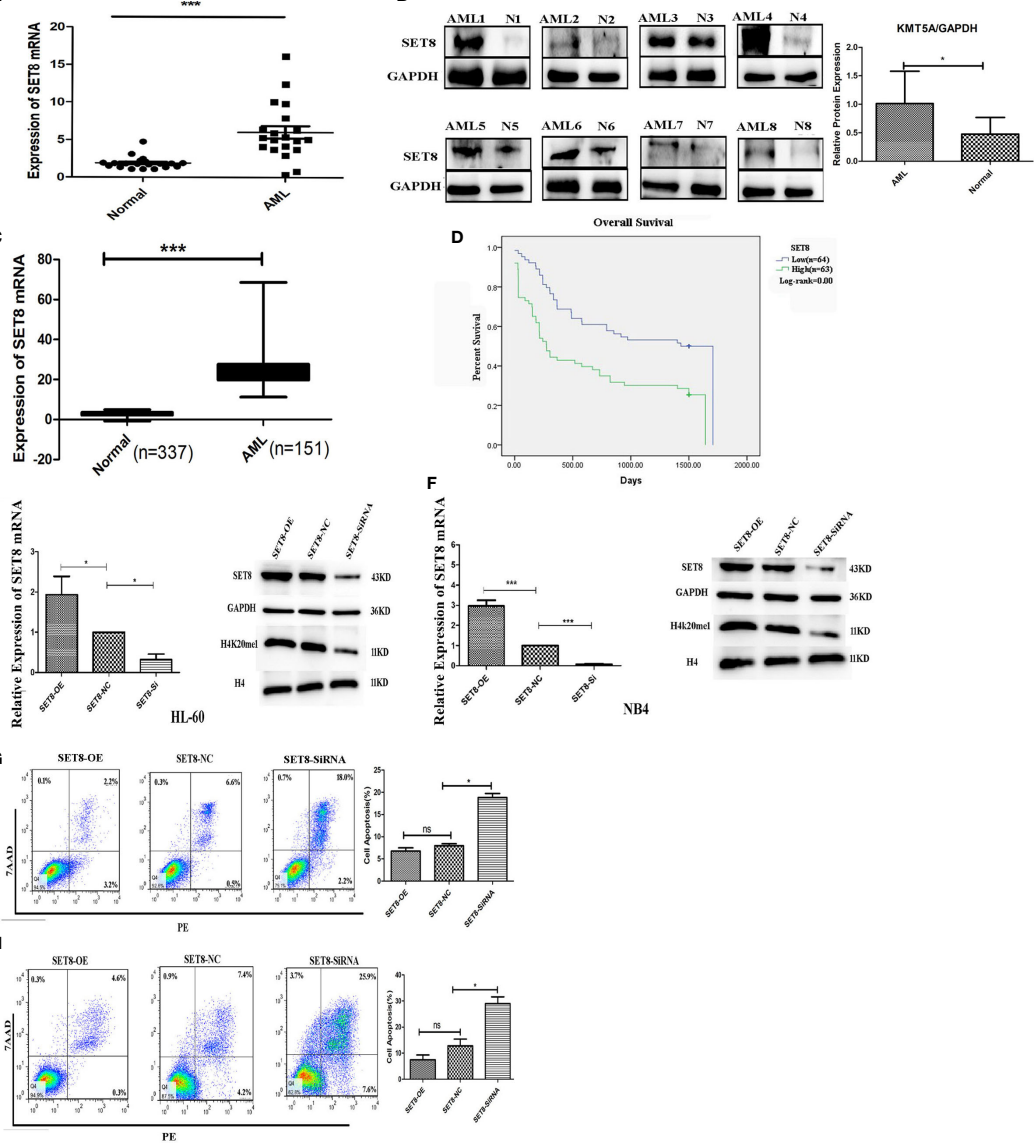
SET8 is highly expressed in acute myeloid leukemia (AML) and is associated with poor prognosis. **(A)** *SET8* mRNA expression in AML patients and healthy control subjects. **(B)** SET8 protein expression in AML patients. **(C, D)** The Cancer Genome Atlas and Genotype-Tissue Expression database analyses of *SET8* expression between AML patients and healthy individuals. **(E, F)** Relative expression of SET8 and H4K20me1 in cells (HL-60 and NB4) transduced with a lentiviral vector determined through quantitative real-time PCR and Western blotting. **(G, H)** Flow cytometric analysis of Annexin V-PE/7-AAD staining shows that the knockdown of *SET8* expression with siRNA induced apoptosis in HL-60 and NB4 cells. ns, not significant; *p < 0.05; ***p < 0.001.

To understand the role of SET8 in AML pathogenesis, we evaluated SET8 expression in AML patients and healthy individuals. We sampled peripheral blood from 20 AML patients and 20 healthy control participants and quantified SET8 expression in isolated peripheral blood leukocytes. RT-PCR and Western blotting revealed that SET8 was significantly upregulated in AML patients compared with the healthy controls (**Figures 3A, B**). Then, we analyzed RNA-seq data from the peripheral blood of AML patients using TCGA database and the RNA-seq data from peripheral blood of healthy people using the GTEx database (20) to verify our results. The analysis showed that the expression of *SET8* mRNA in AML patients was significantly higher in AML patients than in healthy people and was associated with a poor prognosis (**Figures 3C, D**).

HL-60 and NB4 cells were transfected with siRNAs or overexpression vectors to silence or overexpress SET8, and SET8 expression was quantified by RT-PCR and Western blotting (**Figures 3E, F**). Furthermore, apoptosis was assessed by flow cytometry after the transfections. The results showed that early apoptosis and late apoptosis were significantly increased after knockdown of SET8 in the AML cell lines. However, SET8 overexpression did not affect apoptosis, which may be explained by the low level of apoptosis in SET8-NC cells (**Figures 3G, H**). These results suggest that SET8 is involved in leukemia pathogenesis and may be a potential therapeutic target in AML.

### LukS-PV Induced Apoptosis in Acute Myeloid Leukemia Cells by Downregulating SET8 and H4K20me1

**Figure 4:**
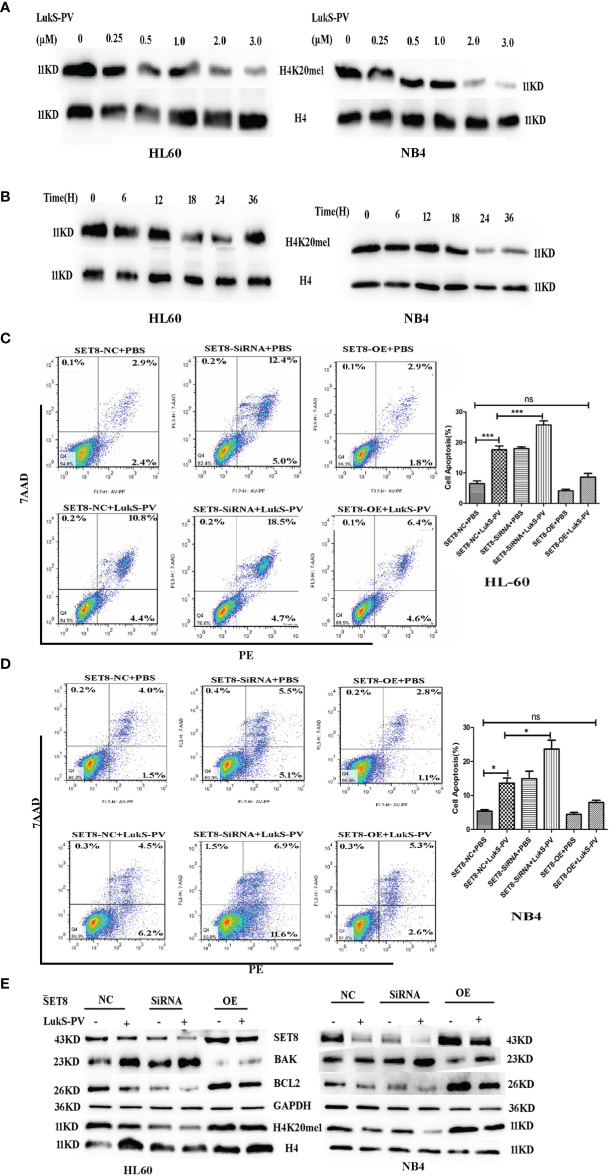
LukS-PV induces apoptosis in acute myeloid leukemia (AML) cells by downregulating SET8/H4K20me1. **(A)** HL-60 and NB4 cells were treated with LukS-PV at different concentrations for 24 h, and H4K20me1 expression was assessed by Western blotting. **(B)** HL-60 and NB4 cells were treated with 3.0 μM of LukS-PV at different timepoints, and H4K20me1 expression was assessed by Western blotting. **(C, D)** SET8 knockdown induced apoptosis and SET8 overexpression inhibited apoptosis in HL-60 **(C)** and NB4 **(D)** cells treated with LukS-PV. **(E)** Expression of SET8 and apoptosis‐associated proteins in HL-60 and NB4 cells was assessed by Western blotting. SET8-OE, SET8 overexpression; SET8-NC, SET8 negative control; SET8-siRNA, SET8 small interfering RNA; ns, not significant; *p < 0.05; ***p < 0.001.

SET8 is a member of the SET domain-containing methyltransferase family and the only modifying enzyme known to catalyze the monomethylation of histone H4 Lys-20 (H4K20me1). We used Western blotting to detect H4K20me1 levels in SET8-siRNA and SET8-overexpressing cells. The results demonstrated that the level of H4K20me1 was increased significantly in SET8-overexpressing cells and decreased in SET8-siRNA cells (**Figures 3E, F**). Furthermore, we treated HL-60 and NB4 cells with different concentrations of LukS-PV for different time periods and quantified H4K20me1 expression. The results showed that LukS-PV reduced H4K20me1 levels in a dose- and time-dependent manner, which was consistent with the results from the SET8 expression experiments (**Figures 4A, B**). These results collectively indicated that LukS-PV downregulated H4K20me1 by regulating SET8 in a dose- and time-dependent manner.

To further determine whether LukS-PV exerted antileukemia effects by downregulating SET8 and H4K20me1, we overexpressed or knocked down SET8 in AML cell lines treated in the cells with 3.0 μM of LukS-PV, and apoptosis was assessed by flow cytometry. The LukS-PV-treated cells displayed significantly greater apoptosis than the PBS-treated cells. Apoptosis was further enhanced in SET8-knockdown cells but markedly alleviated in SET8-overexpressing cells, indicating that the effect of LukS-PV on apoptosis was inhibited by SET8 expression (**Figures 4C, D**). Interestingly, we found that the level of apoptosis was the highest in the SET8-siRNA + LukS-PV group, likely because Luks-PV also induced apoptosis through other pathways, and there was an added apoptotic effect after knocking down SET8. These results indicated that SET8 downregulation is one of the mechanisms by which LukS-PV induced apoptosis in AML cells. Additionally, LukS-PV treatment decreased the protein levels of SET8, H4K20me1, and the anti-apoptotic protein BCL2 and increased the pro-apoptotic protein BAK, and this effect was further enhanced by SET8 knockdown and alleviated by SET8 overexpression (**Figure 4E**).

### *PIK3CB* Is a Downstream Target Gene of SET8-H4K20me1

Studies have shown that SET8 is involved in tumor pathogenesis by catalyzing the monomethylation of H4K20 in target gene promoter regions and promoting gene transcription. To further explore the molecular mechanism of LukS-PV-induced apoptosis and downregulation of SET8 in AML cells, we hypothesized that SET8 regulated downstream target genes through H4K20me1. To verify this hypothesis, target genes regulated by SET8/H4K20me1 were determined by ChIP sequencing. ChIP experiments were first performed with HL-60 cells using antibodies against H4K20me1 after LukS-PV treatment. H4K20me1-associated DNA sequences in LukS-PV-treated cells were then amplified under non-biased conditions, labeled, and sequenced. Through HiSeq2000 with a p-value cutoff of 10^−5^, we identified 2,450 H4K20me1-specific binding peaks of which 731 were upregulated and 1,719 were downregulated (**Supplementary Table S1**).

**Figure 5:**
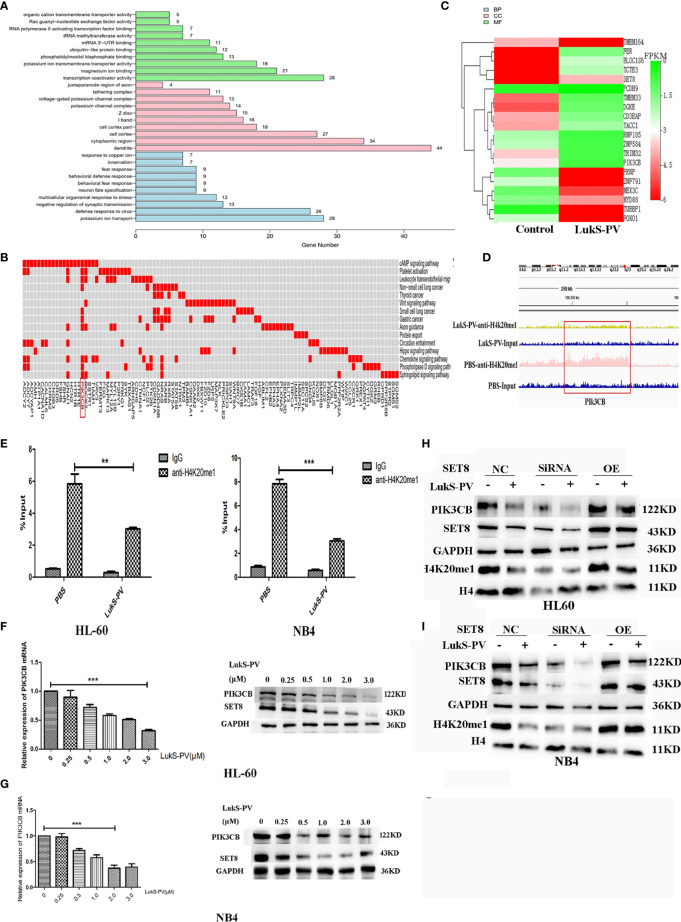
PIK3CB is the target gene for LukS-PV-SET8/H4K20me1. **(A)** Gene Ontology (GO) analysis of downregulated peak related gene binding by LukS-PV-mediated H4K20me1 through ChIP-seq. **(B)** Functional groups in downregulated peak-related genes binding by LukS-PV-mediated H4K20me1. **(C)** Heatmap of different expression genes upon LukS-PV or phosphate-buffered saline (PBS) treatment. **(D)** The binding of LukS-PV and PBS on target gene PIK3CB. **(E)** The binding of H4K20me1 at the PIK3CB promoter was significantly reduced upon LukS-PV treatment *via* quantitative chromatin immunoprecipitation (ChIP)–PCR analysis. Data are presented as fold-change relative to the control with PBS as a negative control. **(F, G)** HL-60 and NB4 cells were treated with LukS-PV at different concentrations for 24 h, and PIK3CB gene and protein expression levels were assessed *via* quantitative real-time PCR and Western blotting. **(H, I)** Western blotting showed that LukS-PV downregulated PIK3CB *via* SET8/H4K20me1 in HL-60 **(H)** and NB4 **(I)** cells. **p < 0.01; ***p < 0.001.

Because LukS-PV inhibits downstream gene transcription *via* downregulation of SET8/H4K20me1, we focused on the genes with reduced H4K20 monomethylation enrichment in the promoter region after LukS-PV treatment. Gene Ontology-based analysis showed that these reduced genes were significantly enriched for transcription coactivator activity and magnesium ion binding, were mainly located in dendrites and cytoplasmic regions, and participated in potassium ion transport and viral defense responses (**Figure 5A**). Kyoto Encyclopedia of Genes and Genomes-based functional enrichment analysis demonstrated that the reduced genes were enriched in cAMP signaling, Wnt signaling, and tumor-related pathways (**Figure 5B**). Data analysis showed that H4K20me1 enrichment in the *PIK3CB*, *ROCK2*, and *GNAI1* promoter regions decreased significantly. Furthermore, PIK3CB is involved in tumor-related signaling pathways, and the decrease in H4K20 methylation in the *PIK3CB* promoter region was the most obvious (**Figure 5D**). Similarly, RNA-seq results showed that *PIK3CB* mRNA was downregulated after LukS-PV treatment (**Figure 5C**). Moreover, Maeda et al. reported that PIK3CB plays a crucial role in apoptosis in renal cell carcinoma (24). Collectively, our data showed that *PIK3CB* was a potential downstream target gene of LukS-PV, and regulation of *PIK3CB* was mediated by SET8/H4K20me1. We verified this finding through ChIP-PCR in HL-60 and NB4 cells. Accordingly, upon LukS-PV treatment, the binding of H4K20me1 to the *PIK3CB* promoter was significantly reduced (**Figure 5E**). We treated HL-60 and NB4 with different concentrations of LukS-PV, and *PIK3CB* mRNA and protein expression levels were reduced in a dose-dependent manner (**Figures 5F, G**). Additionally, knockdown of SET8 reduced the expression of PIK3CB, while overexpression of SET8 promoted the expression of PIK3CB (**Figures 5H, I**). Collectively, LukS-PV inhibited the expression of PIK3CB *via* downregulation of SET8/H4K20me1.

### LukS-PV Induced Apoptosis in Acute Myeloid Leukemia Cells by Downregulating PIK3CB *via* SET8/H4K20me1

**Figure 6:**
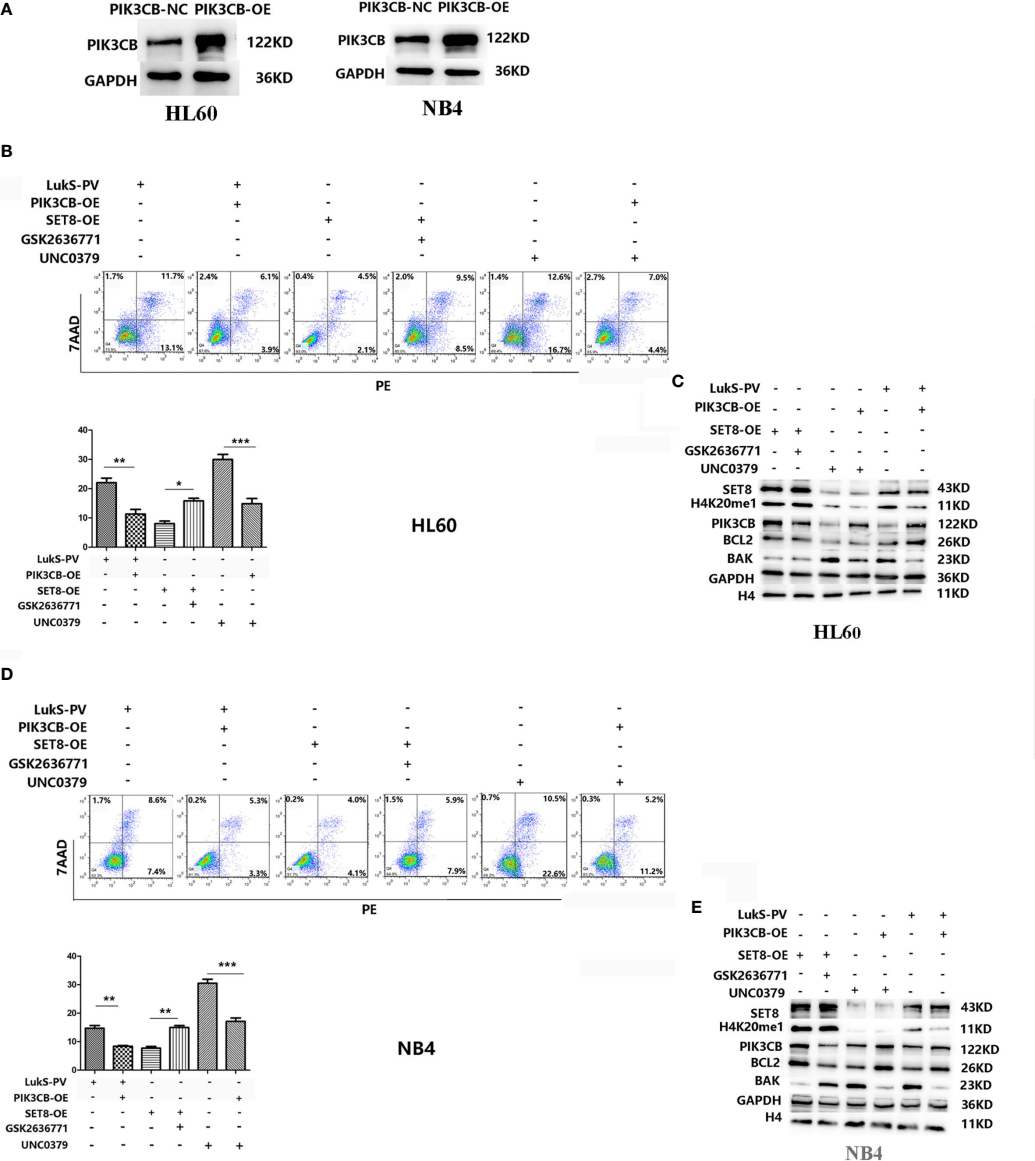
LukS-PV induces apoptosis in acute myeloid leukemia (AML) cells by downregulating PIK3CB *via* SET8/H4K20me1. **(A)** PIK3CB overexpression in HL-60 (left) and NB4 (right) cells. **(B)** Apoptosis was determined in HL-60 cells *via* flow cytometry. **(C)** Apoptosis-related proteins were determined by Western blotting in HL-60 cells. **(D)** Apoptosis was determined in NB4 cells *via* flow cytometry. **(E)** Apoptosis-related proteins were determined by Western blotting in NB4 cells. ns, not significant; *p < 0.05; **p < 0.01; ***p < 0.001.

Because PIK3CB was the downstream target gene of LukS-PV, we investigated whether PIK3CB played a role in apoptosis induced by LukS-PV. PIK3CB was overexpressed in AML cell lines (**Figure 6A**), which were then exposed to 3.0 μM of LukS-PV for 24 h. The flow cytometry results showed that overexpression of PIK3CB inhibited apoptosis induced by LukS-PV (**Figures 6B, D**). Furthermore, we examined the effect of GSK2636771 (a PIK3CB inhibitor) on apoptosis in SET8-overexpressing cells and UNC0379 (a SET8 inhibitor) on apoptosis in PIK3CB-overexpressing cells. The flow cytometry results demonstrated that inhibition of PIK3CB induced apoptosis in SET8-overexpressing cells; however, overexpression of PIK3CB prevented apoptosis induced by SET8 inhibition (**Figures 6B, D**). Finally, the levels of apoptosis-associated proteins were in accordance with the degree of apoptosis (**Figures 6C, E**). Together, our results indicated that LukS-PV induced apoptosis by downregulating the expression of target gene *PIK3CB*, and this downregulation was mediated by SET8/H4K20me1 in AML cells.

### LukS-PV Induced Apoptosis *via* the PIK3CB/AKT/FOXO1 Signaling Pathway by Targeting SET8

**Figure 7:**
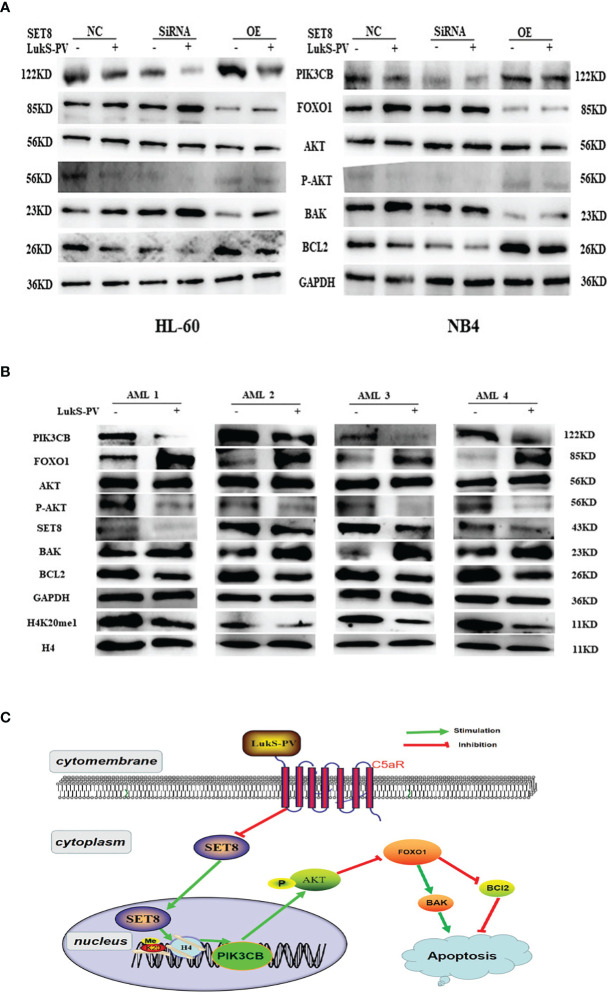
LukS-PV induces apoptosis *via* the PIK3CB signal pathway by targeting SET8. **(A)** Levels of PIK3CB, FOXO1, AKT, apoptosis‐associated (BAK/BCL2) proteins, and AKT phosphorylation in HL-60 and NB4 cells were assessed through Western blotting. **(B)** Expression levels of SET8, H4K20me1, PIK3CB, FOXO1, AKT, apoptosis‐associated (BAK/BCL2), proteins, and AKT phosphorylation in primary acute myeloid leukemia (AML) blasts were assessed through Western blotting. **(C)** Proposed mechanism of action of LukS-PV in acute myeloid leukemia cells. SET8-OE, SET8 overexpression; SET8-NC, SET8 negative control; SET8-SiRNA, SET8 small interfering RNA.

It was reported that PIK3CB inhibits transcription factor FOXO1 by regulating AKT phosphorylation and inhibits apoptosis by regulating the expression of BAK and BCL2. Furthermore, our RNA sequencing results showed that *FOXO1* mRNA was upregulated after LukS-PV treatment (**Figure 5C**). Therefore, we hypothesized that LukS-PV may induce apoptosis *via* the PIK3CB/AKT/FOXO1 signaling pathway by targeting SET8. We verified this molecular mechanism by Western blotting, and the results were in line with our expectations. We found that LukS-PV-treated HL-60 and NB4 cells had lower levels of PIK3CB, pAKT (Ser 473), and anti-apoptotic BCL2 but higher levels of FOXO1 and pro-apoptotic BAK than PBS-treated control cells. These effects were further enhanced after SET8 knockdown with siRNA and markedly alleviated in SET8-overexpressing cells (**Figure 7A**).

Similarly, we determined the levels of associated proteins in primary AML blasts *via* Western blotting. In accordance, the results showed that treatment with 3.0 μM of LukS-PV significantly decreased the levels of SET8, H4K20me1, PIK3CB, pAKT (Ser 473), and anti-apoptotic BCL2 but increased the levels of FOXO1 and pro-apoptotic BAK as compared with the PBS-treated control group (**Figure 7B**). Together, our results indicated that LukS-PV induced apoptosis *via* the PIK3CB/AKT/FOXO1 signaling pathway by targeting SET8 in primary AML blasts (**Figure 7C**).

## Discussion

AML is a complex disease with a diverse genetic landscape, and the current approaches for AML treatment are still far from satisfactory. Target cell specificity and cytotoxicity of bacterial toxins have gained importance in the development of new antitumor drugs (3, 4). In this study with AML cells, we demonstrated that LukS-PV induced apoptosis *in vitro* and inhibited cell invasion *in vivo.* Moreover, we found that SET8 expression was decreased significantly after LukS-PV treatment, and SET8 is highly expressed in AML and is associated with poor prognosis. Furthermore, we confirmed that LukS-PV induced AML apoptosis *via* SET8 and identified *PIK3CB* as a downstream target gene for apoptosis mediated by SET8/H4K20me1. Finally, our results indicated that LukS-PV induced apoptosis *via* the PIK3CB-AKT-FOXO1 signaling pathway by targeting SET8.

Recent studies have revealed that changes in histone modification play an important role in leukemia pathogenesis (25, 26). For example, histone methylation has been reported to regulate stem cell differentiation and leukemia pathogenesis (27). This phenomenon is precisely based on the reversibility of epigenetic modifications that may facilitate targeted leukemia therapy (28). For instance, azacitidine and decitabine are DNA methyltransferase inhibitors approved for clinical treatment of AML (29, 30). Histone methyltransferase (EZH2) and demethylase (LSD1) targeting drugs have entered clinical trials (31, 32). In summary, histone modifications are potentially promising for targeted therapy for leukemia. SET8 is a member of the SET domain-containing methyltransferase family and specifically targets H4K20me1 (33). SET8 is involved in vital cellular processes, including transcriptional regulation (34), S-phase cell cycle progression (35), genomic replication and stability (36), and DNA repair (37). Aberrant SET8 expression has been linked to numerous solid tumors. High SET8 levels are also associated with poor survival in cancer patients (38–40). However, SET8 has so far been poorly studied in leukemia. In this study, we found that SET8 was overexpressed in AML patients and associated with a poor prognosis, and knockdown of SET8 expression induced apoptosis in AML cells. These results suggest that SET8 may be a potential therapeutic target for AML.

Bacterial toxins reportedly have specific cytotoxic effects on target cells, including tumor cells, and they have received increasing attention in the development of antitumor drugs. As a new anti-AML drug, diphtheria toxin has entered the stage of clinical experimentation (41, 42). LukS-PV is the S component of PVL secreted by *S. aureus*. Our previous research has shown that LukS-PV has antileukemia activity *in vivo* and *in vitro* by inhibiting proliferation and inducing apoptosis and differentiation (12–15). Furthermore, we previously demonstrated that LukS-PV inhibited proliferation and induced apoptosis by downregulating histone acetylation in HCC cells (19), suggesting that LukS-PV maybe exert antileukemia activity by targeting histone epigenetic modifiers. In the current study, we found that LukS-PV induced apoptosis by downregulating SET8 and H4K20me1 and identified *PIK3CB* as a potential target gene. Our study indicates that SET8-PIK3CB signaling is one of the mechanisms by which LukS-PV induced apoptosis in AML cells.

The phosphatidylinositol 3-kinase (PI3K) pathway plays a pivotal role in cell growth, proliferation, and survival by integrating extracellular growth signals (43). PIK3CB is a member of the PI3K family, and hyperactivation of the PI3K pathway contributes to cancer progression in humans (44). AKT, a serine/threonine-protein kinase, is one of the most well-characterized targets of the PI3K pathway. Yutaka et al. reported that TGF-β selectively induces AKT phosphorylation at Ser 473 in a PIK3CB-dependent manner in CD4+ T cells, resulting in the inhibition of FOXO transcription factors (45). Furthermore, studies have shown that FOXO factors promote apoptosis by inducing the expression of multiple pro-apoptotic members of the BCL2 family of mitochondria-targeting proteins (46). The present research revealed that *PIK3CB* is a downstream target gene of LukS-PV signaling. LukS-PV decreased AKT Ser 473 phosphorylation and increased FOXO1 levels, thus inducing apoptosis by decreasing BCL2 and increasing BAK in HL-60 and NB4 cells.

Several limitations to this study need to be acknowledged. First, the number of AML patient samples was small, and further in-depth research will be required using a larger number of clinical samples. Second, we used a xenograft tumor model, and few leukemia cells were present in mouse peripheral blood, which made it difficult to isolate enough leukemia cells for apoptosis experiments. Third, we found that the levels of SET8 and H4K20me1 reduced by LukS-PV were maximally downregulated at the 24 h timepoint and then increased at the 36 h timepoint. Indeed, in previous research, we found that the effects of LukS-PV were time-dependent; apoptosis was induced at an early stage (<24 h), and cell differentiation was induced at a later stage (36–48 h) (15). Whether regulation of SET8 expression by LukS-PV is also time-dependent remains to be further studied.

In conclusion, these results demonstrate that LukS-PV induced apoptosis in AML cells *via* the PIK3CB/AKT/FOXO1 signal transduction pathway by targeting the methyltransferase SET8. Our data suggest that SET8 may be a potential therapeutic target for AML. Furthermore, LukS-PV may be a valuable drug candidate for treatment of AML that targets epigenetic modifications.

## Data Availability Statement

The sequence data presented in the study are deposited in the CBI SRA repository, accession number: PRJNA767433, https://www.ncbi.nlm.nih.gov/bioproject/PRJNA767433.

## Ethics Statement

The studies involving human participants were reviewed and approved by Ethics Committee and Institutional Review Board of University of Science and Technology of China, Anhui, China (Approval number: 2019-N(H)-101). The patients/participants provided their written informed consent to participate in this study. The animal study was reviewed and approved by Ethics Committee and Institutional Review Board of University of Science and Technology of China, Anhui, China (Approval number: 2019-N(H)-101).

## Author Contributions

LX: conceptualization, methodology, formal analysis, writing—original draft, data curation, and investigation. LS: conceptualization, investigation, writing—original draft, and funding acquisition. PD: resources and software. FM: methodology and software. KS: investigation and data curation. PQ: software and formal analysis. WC: visualization and supervision. YD: supervision and project administration. YM: writing—review and editing, and supervision. XM: writing—review and editing, supervision, project administration, and funding acquisition. All authors contributed to the article and approved the submitted version.

## Funding

This work was supported by the National Natural Science Foundation of China (Grant No. 81972001, No. 81572065), the Anhui Natural Science Foundation (Grant No. 1808085QH259), and Fundamental Research Funds for Central Universities (WK9110000007, WK9110000107).

## Conflict of Interest

The authors declare that the research was conducted in the absence of any commercial or financial relationships that could be construed as a potential conflict of interest.

## Publisher’s Note

All claims expressed in this article are solely those of the authors and do not necessarily represent those of their affiliated organizations, or those of the publisher, the editors and the reviewers. Any product that may be evaluated in this article, or claim that may be made by its manufacturer, is not guaranteed or endorsed by the publisher.

## Abbreviations

AML, acute myeloid leukemia; FCM, flow cytometry; ChIP, chromatin immunoprecipitation; SET-NC, SET8 negative control; SET8-OE, SET8 overexpression; SET8-SiRNA, SET8 small interfering RNA.
