# Supplementary material for: LukS-PV Induces Apoptosis via the SET8-H4K20me1-PIK3CB Axis in Human Acute Myeloid Leukemia Cells
Source: Front Oncol. 2021 Oct 20;11:718791. doi: 10.3389/fonc.2021.718791 (PMC8565356; doi:10.3389/fonc.2021.718791)
Supplement: Supplementary file 1 [file DataSheet_1.pdf]

peak	Chromoso	Start	End	Overlaped	Downstrea	Symbol1	Distance	Downstrea	Symbol2	Distance	annovar_r	annovar_g	signal	type	overlap1	overlap2
peak1	chr1	962985	963194	2	NR_03886	LINC0134	109307	NM_0011	HES4	27537	intronic	AGRN	15.86293	Up	luks_peak	NULL
peak2	chr1	1707002	1707152	3	NM_1387	CALML6	139189	NM_0011	NADK	16996	intronic	NADK	5.39316	Up	luks_peak	NULL
peak3	chr1	2178894	2179083	1	NM_0070	RER1	144225	NM_0012	FAAP20	34829	intronic	SKI	3.73192	Down	NULL	PBS_peak_65
peak4	chr1	2183700	2183861	1	NM_0070	RER1	139433	NM_0012	FAAP20	39621	intronic	SKI	6.69815	Down	NULL	NULL
peak5	chr1	2189642	2189840	1	NM_0070	RER1	133473	NM_0012	FAAP20	45582	intronic	SKI	8.77796	Down	NULL	PBS_peak_69
peak6	chr1	2190410	2190704	1	NM_0070	RER1	132657	NM_0012	FAAP20	46398	intronic	SKI	6.28449	Down	NULL	PBS_peak_70
peak7	chr1	2197965	2198163	1	NM_0070	RER1	125150	NM_0012	FAAP20	53905	intronic	SKI	11.59096	Down	NULL	PBS_peak_74
peak8	chr1	2198588	2198752	1	NM_0070	RER1	124544	NM_0012	FAAP20	54511	intronic	SKI	10.38343	Down	NULL	PBS_peak_74
peak9	chr1	2200058	2200227	1	NM_0070	RER1	123071	NM_0012	FAAP20	55983	intronic	SKI	3.82796	Down	NULL	PBS_peak_75
peak10	chr1	2200427	2201031	1	NM_0070	RER1	122485	NM_0012	FAAP20	56570	intronic	SKI	5.69347	Down	NULL	PBS_peak_76
peak11	chr1	2204552	2204813	1	NM_0070	RER1	118531	NM_0012	FAAP20	60523	intronic	SKI	7.21944	Down	NULL	PBS_peak_79
peak12	chr1	2206123	2206436	1	NM_0070	RER1	116934	NM_0012	FAAP20	62120	intronic	SKI	5.30317	Down	NULL	PBS_peak_81
peak13	chr1	2207196	2207409	1	NM_0070	RER1	115911	NM_0012	FAAP20	63143	intronic	SKI	6.01402	Down	NULL	PBS_peak_81
peak14	chr1	2208089	2208351	1	NM_0070	RER1	114994	NM_0012	FAAP20	64061	intronic	SKI	4.33089	Down	NULL	PBS_peak_82
peak15	chr1	2210433	2210597	1	NM_0070	RER1	112699	NM_0012	FAAP20	66356	intronic	SKI	5.37746	Down	NULL	PBS_peak_84
peak16	chr1	2226488	2226675	1	NM_0070	RER1	96632	NM_0012	FAAP20	82422	intronic	SKI	4.81752	Down	NULL	PBS_peak_91
peak17	chr1	6090742	6091154	3	NM_0011	KCNAB2	3400	NR_11198	NPHP4	38415	intronic	KCNAB2	6.73808	Down	NULL	PBS_peak_97
peak18	chr1	6091240	6091394	3	NM_0011	KCNAB2	3031	NR_11198	NPHP4	38784	intronic	KCNAB2	3.56422	Down	NULL	PBS_peak_97
peak19	chr1	6093405	6093685	3	NM_0011	KCNAB2	803	NR_11198	NPHP4	41012	intronic	KCNAB2	4.77982	Down	NULL	PBS_peak_99
peak20	chr1	6093758	6093911	3	NM_0011	KCNAB2	513	NR_11198	NPHP4	41301	intronic	KCNAB2	9.16594	Down	NULL	PBS_peak_99
peak21	chr1	6094462	6094648	4	NM_0011	KCNAB2	11426	NR_11198	NPHP4	42022	UTR5	KCNAB2(	4.8893	Down	NULL	PBS_peak_100
peak22	chr1	6096019	6096187	4	NM_0011	KCNAB2	9878	NR_11198	NPHP4	43570	intronic	KCNAB2	4.66749	Down	NULL	PBS_peak_101
peak23	chr1	6096256	6096410	4	NM_0011	KCNAB2	9648	NR_11198	NPHP4	43800	intronic	KCNAB2	4.20072	Down	NULL	PBS_peak_101
peak24	chr1	6097244	6097590	4	NM_0011	KCNAB2	8564	NR_11198	NPHP4	44884	intronic	KCNAB2	14.33918	Down	NULL	PBS_peak_103
peak25	chr1	6098216	6098467	4	NM_0011	KCNAB2	7639	NR_11198	NPHP4	45808	intronic	KCNAB2	6.04391	Down	NULL	PBS_peak_103
peak26	chr1	6101025	6101274	4	NM_0011	KCNAB2	4831	NR_11198	NPHP4	48616	intronic	KCNAB2	7.89386	Down	NULL	PBS_peak_105
peak27	chr1	6102446	6102635	4	NM_0011	KCNAB2	3440	NR_11198	NPHP4	50007	intronic	KCNAB2	4.12154	Down	NULL	PBS_peak_106
peak28	chr1	6104771	6104933	4	NM_0011	KCNAB2	1129	NR_11198	NPHP4	52319	intronic	KCNAB2	3.47744	Down	NULL	PBS_peak_109
peak29	chr1	6105793	6105944	4	NM_0011	KCNAB2	112	NR_11198	NPHP4	53335	intronic	KCNAB2	4.22194	Down	NULL	PBS_peak_110
peak30	chr1	6129219	6129392	6	NM_2073	RNF207	136883	NR_11198	NPHP4	76772	intronic	KCNAB2	5.45382	Down	NULL	PBS_peak_116
peak31	chr1	6129669	6129842	6	NM_2073	RNF207	136433	NR_11198	NPHP4	77222	intronic	KCNAB2	5.47903	Down	NULL	PBS_peak_117
peak32	chr1	6130794	6130955	6	NM_2073	RNF207	135314	NR_11198	NPHP4	78341	intronic	KCNAB2	7.79861	Down	NULL	PBS_peak_118
peak33	chr1	9360157	9360326	1	NM_0323	SLC25A3	239286	NR_132742		117844	intronic	SPSB1	4.68661	Up	NULL	NULL
peak34	chr1	9361631	9361785	1	NM_0323	SLC25A3	237820	NR_132742		119311	intronic	SPSB1	8.16893	Up	NULL	NULL
peak35	chr1	9362101	9362349	1	NM_0323	SLC25A3	237303	NR_132742		119828	intronic	SPSB1	7.10191	Up	luks_peak	NULL
peak36	chr1	9363015	9363184	1	NM_0323	SLC25A3	236428	NR_132742		120702	intronic	SPSB1	8.97046	Up	luks_peak	NULL
peak37	chr1	9363888	9364044	1	NM_0323	SLC25A3	235562	NR_132742		121569	intronic	SPSB1	5.87542	Up	NULL	NULL
peak38	chr1	9364162	9364383	1	NM_0323	SLC25A3	235255	NR_132742		121875	intronic	SPSB1	12.18752	Up	NULL	NULL
peak39	chr1	9365018	9365229	1	NM_0323	SLC25A3	234404	NR_132742		122726	intronic	SPSB1	5.02176	Up	luks_peak	NULL
peak40	chr1	9365316	9365487	1	NM_0323	SLC25A3	234126	NR_132742		123004	intronic	SPSB1	6.75927	Up	luks_peak	NULL
peak41	chr1	9365581	9365742	1	NM_0323	SLC25A3	233866	NR_132742		123264	intronic	SPSB1	4.83934	Up	luks_peak	NULL
peak42	chr1	9367650	9368031	1	NM_0323	SLC25A3	231687	NR_132742		125443	intronic	SPSB1	6.72565	Up	luks_peak	NULL
peak43	chr1	9368179	9368377	1	NM_0323	SLC25A3	231250	NR_132742		125881	intronic	SPSB1	35.85557	Up	luks_peak	NULL
peak44	chr1	9371904	9372176	1	NM_0323	SLC25A3	227488	NR_132742		129643	intronic	SPSB1	3.84737	Up	NULL	NULL
peak45	chr1	9372514	9372696	1	NM_0323	SLC25A3	226923	NR_132742		130208	intronic	SPSB1	5.45474	Up	luks_peak	NULL
peak46	chr1	9373540	9374167	1	NM_0323	SLC25A3	225674	NR_132742		131456	intronic	SPSB1	21.14281	Up	luks_peak	NULL
peak47	chr1	9374931	9375341	1	NM_0323	SLC25A3	224392	NR_132742		132739	intronic	SPSB1	7.94701	Up	luks_peak	NULL
peak48	chr1	9376954	9377136	1	NM_0323	SLC25A3	222483	NR_132742		134648	intronic	SPSB1	33.2777	Up	luks_peak	NULL
peak49	chr1	9378644	9378965	1	NM_0323	SLC25A3	220723	NR_132742		136407	intronic	SPSB1	11.46174	Up	luks_peak	NULL
peak50	chr1	9379326	9379820	1	NM_0323	SLC25A3	219955	NR_132742		137176	intronic	SPSB1	9.46473	Up	luks_peak	NULL
peak51	chr1	9380076	9380248	1	NM_0323	SLC25A3	219366	NR_132742		137765	intronic	SPSB1	4.45975	Up	NULL	NULL
peak52	chr1	9380373	9380543	1	NM_0323	SLC25A3	219070	NR_132742		138061	intronic	SPSB1	4.28673	Up	luks_peak	NULL
peak53	chr1	9382469	9382801	1	NM_0323	SLC25A3	216893	NR_132742		140238	intronic	SPSB1	6.62604	Up	luks_peak	NULL
peak54	chr1	9383225	9383379	1	NM_0323	SLC25A3	216226	NR_132742		140905	intronic	SPSB1	17.88245	Up	NULL	NULL
peak55	chr1	9383563	9383780	1	NM_0323	SLC25A3	215856	NR_132742		141274	intronic	SPSB1	22.19157	Up	NULL	NULL
peak56	chr1	9384484	9384639	1	NM_0323	SLC25A3	214966	NR_132742		142164	intronic	SPSB1	9.94138	Up	NULL	NULL
peak57	chr1	9384769	9384926	1	NM_0323	SLC25A3	214680	NR_132742		142450	intronic	SPSB1	9.6059	Up	NULL	NULL
peak58	chr1	9385880	9386040	1	NM_0323	SLC25A3	213568	NR_132742		143563	intronic	SPSB1	12.11577	Up	NULL	NULL
peak59	chr1	9386699	9387026	1	NM_0323	SLC25A3	212665	NR_132742		144465	intronic	SPSB1	10.58141	Up	luks_peak	NULL
peak60	chr1	9387365	9387663	1	NM_0323	SLC25A3	212014	NR_132742		145117	intronic	SPSB1	12.83193	Up	luks_peak	NULL
peak61	chr1	9390113	9390331	1	NM_0323	SLC25A3	209306	NR_132742		147825	intronic	SPSB1	5.05748	Up	NULL	NULL
peak62	chr1	9390575	9390725	1	NM_0323	SLC25A3	208878	NR_132742		148253	intronic	SPSB1	5.44341	Up	NULL	NULL
peak63	chr1	9391627	9391789	1	NM_0323	SLC25A3	207820	NR_132742		149311	intronic	SPSB1	5.82803	Up	NULL	NULL
peak64	chr1	9392713	9393255	1	NM_0323	SLC25A3	206544	NR_132742		150587	intronic	SPSB1	9.36313	Up	luks_peak	NULL
peak65	chr1	9395601	9395881	1	NM_0323	SLC25A3	203787	NR_132742		153344	intronic	SPSB1	16.51142	Up	luks_peak	NULL
peak66	chr1	9397286	9397843	1	NM_0323	SLC25A3	201963	NR_132742		155167	intronic	SPSB1	24.43167	Up	luks_peak	NULL
peak67	chr1	9398095	9398367	1	NM_0323	SLC25A3	201297	NR_132742		155834	intronic	SPSB1	20.36413	Up	luks_peak	NULL
peak68	chr1	9398463	9398625	1	NM_0323	SLC25A3	200984	NR_132742		156147	intronic	SPSB1	10.57161	Up	NULL	NULL
peak69	chr1	9398777	9398993	1	NM_0323	SLC25A3	200643	NR_132742		156488	intronic	SPSB1	9.36524	Up	luks_peak	NULL
peak70	chr1	9399435	9399897	1	NM_0323	SLC25A3	199862	NR_132742		157269	intronic	SPSB1	14.08502	Up	luks_peak	NULL
peak71	chr1	9400108	9400356	1	NM_0323	SLC25A3	199296	NR_132742		157835	intronic	SPSB1	6.49723	Up	luks_peak	NULL
peak72	chr1	9401225	9401448	1	NM_0323	SLC25A3	198191	NR_132742		158939	intronic	SPSB1	15.92855	Up	luks_peak	NULL
peak73	chr1	9403221	9403450	1	NM_0323	SLC25A3	196192	NR_132742		160938	intronic	SPSB1	22.45831	Up	luks_peak	NULL
peak74	chr1	9403575	9403725	1	NM_0323	SLC25A3	195878	NR_132742		161253	intronic	SPSB1	18.01777	Up	NULL	NULL
peak75	chr1	9406198	9406354	1	NM_0323	SLC25A3	193252	NR_132742		163879	intronic	SPSB1	28.53096	Up	luks_peak	NULL
peak76	chr1	9406663	9406948	1	NM_0323	SLC25A3	192722	NR_132742		164408	intronic	SPSB1	6.22987	Up	luks_peak	NULL
peak77	chr1	9407010	9407314	1	NM_0323	SLC25A3	192366	NR_132742		164765	intronic	SPSB1	10.51575	Up	luks_peak	NULL
peak78	chr1	9409499	9409757	1	NM_0323	SLC25A3	189900	NR_132742		167231	intronic	SPSB1	11.3385	Up	luks_peak	NULL
peak79	chr1	9410217	9410534	1	NM_0323	SLC25A3	189152	NR_132742		167978	intronic	SPSB1	6.97168	Up	luks_peak	NULL
peak80	chr1	9411253	9411421	1	NM_0323	SLC25A3	188191	NR_132742		168940	intronic	SPSB1	32.24035	Up	NULL	NULL
peak81	chr1	9411626	9411977	1	NM_0323	SLC25A3	187726	NR_132742		169404	intronic	SPSB1	10.97274	Up	luks_peak	NULL
peak82	chr1	9412039	9412544	1	NM_0323	SLC25A3	187236	NR_132742		169894	intronic	SPSB1	18.73742	Up	luks_peak	NULL
peak83	chr1	9414018	9414200	1	NM_0323	SLC25A3	185419	NR_132742		171712	intronic	SPSB1	9.19416	Up	NULL	NULL
peak84	chr1	9415516	9415682	1	NM_0323	SLC25A3	183929	NR_132742		173202	intronic	SPSB1	22.41312	Up	NULL	NULL
peak85	chr1	9420250	9420450	1	NM_0323	SLC25A3	179178	NR_132742		177953	intronic	SPSB1	7.66711	Up	NULL	NULL
peak86	chr1	9422132	9422297	1	NM_0323	SLC25A3	177313	NR_132742		179817	intronic	SPSB1	28.84766	Up	NULL	NULL
peak87	chr1	9423077	9423254	1	NM_0323	SLC25A3	176362	NR_132742		180768	intronic	SPSB1	6.39639	Up	NULL	NULL
peak88	chr1	9427505	9427699	1	NM_0323	SLC25A3	171926	NR_132742		185205	exonic	SPSB1	27.32407	Up	NULL	NULL
peak89	chr1	9723343	9723538	1	NM_0012	NMNAT1	279540	NR_02704	PIK3CD-	8796	intronic	PIK3CD	18.3456	Down	NULL	PBS_peak_134
peak90	chr1	9724442	9724852	1	NM_0012	NMNAT1	278334	NR_02704	PIK3CD-	10003	intronic	PIK3CD	8.87284	Down	NULL	PBS_peak_135
peak91	chr1	9727614	9727799	1	NM_0012	NMNAT1	275274	NR_02704	PIK3CD-	13062	intronic	PIK3CD	11.74014	Down	NULL	PBS_peak_136
peak92	chr1	9731258	9731418	1	NM_0012	NMNAT1	271643	NR_02704	PIK3CD-	16694	intronic	PIK3CD	16.39896	Down	NULL	PBS_peak_138
peak93	chr1	9731747	9731955	1	NM_0012	NMNAT1	271130	NR_02704	PIK3CD-	17207	intronic	PIK3CD	5.04039	Down	NULL	PBS_peak_139
peak94	chr1	9736359	9736657	3	NM_0012	NMNAT1	266473	NR_02704	PIK3CD-	21864	ncRNA_in	PIK3CD-	5.96236	Down	NULL	PBS_peak_143
peak95	chr1	9737453	9737621	3	NM_0012	NMNAT1	265444	NR_02704	PIK3CD-	22893	ncRNA_in	PIK3CD-	18.02597	Down	NULL	PBS_peak_144
peak96	chr1	9738322	9738533	3	NM_0012	NMNAT1	264553	NR_02704	PIK3CD-	23783	ncRNA_in	PIK3CD-	4.21466	Down	NULL	PBS_peak_145
peak97	chr1	9740814	9740971	4	NM_0012	NMNAT1	262088	NR_02704	PIK3CD-	26248	ncRNA_ex	PIK3CD-	3.89517	Down	NULL	PBS_peak_147
peak98	chr1	9745820	9746015	4	NM_0012	NMNAT1	257063	NR_02704	PIK3CD-	31273	ncRNA_in	PIK3CD-	4.66837	Down	NULL	PBS_peak_150
peak99	chr1	9746593	9746945	4	NM_0012	NMNAT1	256212	NR_02704	PIK3CD-	32125	ncRNA_in	PIK3CD-	12.27579	Down	NULL	PBS_peak_151
peak100	chr1	9748784	9748984	1	NM_0012	NMNAT1	254097	NR_12636	PIK3CD-	1252	intronic	PIK3CD	7.28069	Down	NULL	PBS_peak_154
peak101	chr1	9749207	9749383	1	NM_0012	NMNAT1	253686	NR_12636	PIK3CD-	1663	intronic	PIK3CD	4.514	Down	NULL	PBS_peak_154
peak102	chr1	9751185	9751358	1	NM_0012	NMNAT1	251709	NR_12636	PIK3CD-	3639	intronic	PIK3CD	15.66045	Down	NULL	PBS_peak_156

peak103	chr1	16184562	16184722	1	NR_0399	MIR5096	13002	NR_0242	FLJ37453	10000	intronic	SPEN	8.58775	Down	NULL	PBS_peak_163
peak104	chr1	16666883	16667035	1	NM_0011	SZRD1	26566	NM_0309	RSG1	103300	intronic	FBXO42	3.65383	Down	NULL	PBS_peak_176
peak105	chr1	17754727	17754902	2	NM_0181	ARHGEF	111515	NM_0073	PADI2	308866	intronic	RCC2	4.44683	Down	NULL	PBS_peak_188
peak106	chr1	19718244	19718417	5	NM_0010	MINOS1	205140	NM_0036	AKR7A2	79690	intronic	CAPZB	4.25015	Down	NULL	PBS_peak_203
peak107	chr1	19722927	19723094	5	NM_0010	MINOS1	200460	NM_0036	AKR7A2	84370	intronic	CAPZB	5.69962	Down	NULL	PBS_peak_206
peak108	chr1	19730368	19730554	5	NM_0010	MINOS1	193010	NM_0036	AKR7A2	91821	intronic	CAPZB	4.55256	Down	NULL	PBS_peak_213
peak109	chr1	19747983	19748166	5	NM_0010	MINOS1	175396	NM_0036	AKR7A2	109434	intronic	CAPZB	4.0962	Down	NULL	PBS_peak_228
peak110	chr1	19750156	19750469	5	NM_0010	MINOS1	173158	NM_0036	AKR7A2	111672	intronic	CAPZB	4.36931	Down	NULL	PBS_peak_230
peak111	chr1	19751808	19751961	5	NM_0010	MINOS1	171586	NM_0036	AKR7A2	113244	intronic	CAPZB	4.53282	Down	NULL	PBS_peak_231
peak112	chr1	19757825	19757999	5	NM_0010	MINOS1	165559	NM_0036	AKR7A2	119272	intronic	CAPZB	11.41357	Down	NULL	PBS_peak_237
peak113	chr1	19763386	19763567	5	NM_0010	MINOS1	159994	NM_0036	AKR7A2	124836	intronic	CAPZB	15.5722	Down	NULL	PBS_peak_241
peak114	chr1	19764741	19764905	5	NM_0010	MINOS1	158648	NM_0036	AKR7A2	126183	intronic	CAPZB	4.81333	Down	NULL	PBS_peak_243
peak115	chr1	19766093	19766444	5	NM_0010	MINOS1	157202	NM_0036	AKR7A2	127628	intronic	CAPZB	4.73622	Down	NULL	PBS_peak_244
peak116	chr1	19773027	19773198	5	NM_0010	MINOS1	150358	NM_0036	AKR7A2	134472	intronic	CAPZB	8.21456	Down	NULL	PBS_peak_252
peak117	chr1	19774512	19774749	5	NM_0010	MINOS1	148840	NM_0036	AKR7A2	135990	intronic	CAPZB	4.16671	Down	NULL	PBS_peak_253
peak118	chr1	19775724	19775914	5	NM_0010	MINOS1	147652	NM_0036	AKR7A2	137179	UTR5	CAPZB(N	16.42089	Down	NULL	PBS_peak_254
peak119	chr1	19776491	19776661	5	NM_0010	MINOS1	146895	NM_0036	AKR7A2	137936	intronic	CAPZB	8.80742	Down	NULL	PBS_peak_255
peak120	chr1	19777497	19777655	5	NM_0010	MINOS1	145895	NM_0036	AKR7A2	138936	intronic	CAPZB	5.14943	Down	NULL	PBS_peak_256
peak121	chr1	19777923	19778296	5	NM_0010	MINOS1	145361	NM_0036	AKR7A2	139469	intronic	CAPZB	4.32578	Down	NULL	PBS_peak_256
peak122	chr1	19778479	19778737	5	NM_0010	MINOS1	144863	NM_0036	AKR7A2	139968	intronic	CAPZB	5.84019	Down	NULL	PBS_peak_257
peak123	chr1	19779549	19780091	5	NM_0010	MINOS1	143651	NM_0036	AKR7A2	141180	intronic	CAPZB	8.78616	Down	NULL	PBS_peak_258
peak124	chr1	19780498	19780797	5	NM_0010	MINOS1	142823	NM_0036	AKR7A2	142007	intronic	CAPZB	7.3384	Down	NULL	PBS_peak_259
peak125	chr1	19782950	19783263	5	NM_0010	MINOS1	140364	NM_0036	AKR7A2	144466	intronic	CAPZB	9.13761	Down	NULL	PBS_peak_262
peak126	chr1	19783830	19784038	5	NM_0010	MINOS1	139537	NM_0036	AKR7A2	145294	intronic	CAPZB	8.76654	Down	NULL	PBS_peak_262
peak127	chr1	19784854	19785084	5	NM_0010	MINOS1	138502	NM_0036	AKR7A2	146329	intronic	CAPZB	8.48983	Down	NULL	PBS_peak_263
peak128	chr1	19785487	19785660	5	NM_0010	MINOS1	137897	NM_0036	AKR7A2	146933	intronic	CAPZB	9.5561	Down	NULL	PBS_peak_263
peak129	chr1	19786114	19786426	5	NM_0010	MINOS1	137201	NM_0036	AKR7A2	147630	intronic	CAPZB	6.18313	Down	NULL	PBS_peak_264
peak130	chr1	19787659	19787872	5	NM_0010	MINOS1	135705	NM_0036	AKR7A2	149125	intronic	CAPZB	4.56659	Down	NULL	PBS_peak_267
peak131	chr1	19788978	19789179	5	NM_0010	MINOS1	134392	NM_0036	AKR7A2	150438	intronic	CAPZB	5.85652	Down	NULL	PBS_peak_268
peak132	chr1	19792080	19792246	5	NM_0010	MINOS1	131308	NM_0036	AKR7A2	153523	intronic	CAPZB	4.31045	Down	NULL	PBS_peak_270
peak133	chr1	19792698	19792988	5	NM_0010	MINOS1	130628	NM_0036	AKR7A2	154203	intronic	CAPZB	4.22487	Down	NULL	PBS_peak_270
peak134	chr1	19793463	19793651	5	NM_0010	MINOS1	129914	NM_0036	AKR7A2	154917	intronic	CAPZB	3.79028	Down	NULL	PBS_peak_271
peak135	chr1	19794996	19795220	5	NM_0010	MINOS1	128363	NM_0036	AKR7A2	156468	intronic	CAPZB	5.88677	Down	NULL	PBS_peak_272
peak136	chr1	19800723	19801134	5	NM_0010	MINOS1	122542	NM_0036	AKR7A2	162288	intronic	CAPZB	5.74606	Down	NULL	PBS_peak_277
peak137	chr1	19802744	19802930	5	NM_0010	MINOS1	120634	NM_0036	AKR7A2	164197	intronic	CAPZB	6.00825	Down	NULL	PBS_peak_279
peak138	chr1	19930744	19930923	7	NR_0772	RPS14P3	3467	NM_0013	13932	118698	intronic	MINOS1,I	8.12273	Down	NULL	PBS_peak_285
peak139	chr1	22389545	22389695	3	NR_0396	MIR4418	203112	NR_1106	LOC1019	37079	intronic	CDC42	5.79807	Down	NULL	PBS_peak_292
peak140	chr1	24022470	24022665	2	NM_0031	TCEB3	47288	NM_0021	ID3	136282	intronic	RPL11	4.68159	Down	NULL	PBS_peak_310
peak141	chr1	25250976	25251127	3	NR_0396	MIR4425	98942	NR_1067	MIR6731	5144	intronic	RUNX3	6.18267	Up	NULL	NULL
peak142	chr1	25670117	25670267	1	NM_0012	TMEM57	87157	NR_1351	44	96182	intronic	TMEM50.	17.96442	Up	NULL	NULL
peak143	chr1	27029155	27029306	2	NM_0178	PIGV	85223	NR_1259	LOC1019	235202	intronic	ARID1A	4.44687	Down	NULL	PBS_peak_316
peak144	chr1	27038753	27038903	2	NM_0178	PIGV	75626	NR_1259	LOC1019	244800	intronic	ARID1A	4.91141	Down	NULL	PBS_peak_324
peak145	chr1	27039451	27039624	2	NM_0178	PIGV	74916	NR_1259	LOC1019	245509	intronic	ARID1A	6.75963	Down	NULL	PBS_peak_325
peak146	chr1	27047078	27047301	2	NM_0178	PIGV	67264	NR_1259	LOC1019	253161	intronic	ARID1A	12.65991	Down	NULL	PBS_peak_330
peak147	chr1	27052700	27052864	2	NM_0178	PIGV	61672	NR_1259	LOC1019	258754	intronic	ARID1A	4.07366	Down	NULL	PBS_peak_332
peak148	chr1	27077843	27078018	2	NM_0178	PIGV	36523	NR_1259	LOC1019	283902	intronic	ARID1A	5.04685	Down	NULL	PBS_peak_336
peak149	chr1	29073281	29073607	3	NM_0009	OPRD1	65210	NM_0056	TAF12	103840	intronic	YTHDF2	6.04813	Down	NULL	PBS_peak_343
peak150	chr1	29078421	29078649	3	NM_0009	OPRD1	60119	NM_0056	TAF12	108931	intronic	YTHDF2	4.98286	Down	NULL	PBS_peak_345
peak151	chr1	29085502	29085660	3	NM_0009	OPRD1	53073	NM_0056	TAF12	115977	intronic	YTHDF2	3.75393	Down	NULL	PBS_peak_352
peak152	chr1	29492292	29492465	1	NM_0011	PTPRU	70649	NM_0010	TMEM20	41957	intronic	SRSF4	10.73497	Down	NULL	PBS_peak_359
peak153	chr1	29495092	29495265	1	NM_0011	PTPRU	67849	NM_0010	TMEM20	44757	intronic	SRSF4	19.49646	Down	NULL	PBS_peak_361
peak154	chr1	31216161	31216331	1	NM_0012	ZCCHC17	553583	NR_0396	MIR4420	4167	intronic	LAPTM5	5.43231	Down	NULL	PBS_peak_367
peak155	chr1	31482367	31482582	2	NM_0012	ZCCHC17	287354	NR_0030	SNORD8	41390	intronic	PUM1	4.10927	Down	NULL	PBS_peak_377
peak156	chr1	31484878	31485041	2	NM_0012	ZCCHC17	284869	NR_0030	SNORD8	43875	intronic	PUM1	6.76154	Down	NULL	PBS_peak_379
peak157	chr1	31493644	31493818	2	NM_0012	ZCCHC17	276098	NR_0030	SNORD8	52647	intronic	PUM1	3.89531	Down	NULL	NULL
peak158	chr1	31524165	31524377	2	NM_0012	ZCCHC17	245558	NR_0030	SNORD8	83187	intronic	PUM1	3.93878	Down	NULL	PBS_peak_391
peak159	chr1	32485350	32485512	4	NM_0013	19677	52201	NM_0803	PTP4A2	81443	intronic	KHDRBS	4.47095	Down	NULL	PBS_peak_412
peak160	chr1	32502163	32502332	4	NM_0013	19677	35384	NM_0803	PTP4A2	98259	intronic	KHDRBS	5.95282	Up	luks_peak	NULL
peak161	chr1	33422259	33422528	3	NM_0012	AZIN2	124320	NM_0335	TMEM54	55440	intronic	RNF19B	7.72967	Up	luks_peak	NULL
peak162	chr1	36693855	36694018	1	NM_0011	SH3D21	78057	NM_0012	TRAPPC3	72282	intronic	THRAP3	9.43824	Down	NULL	NULL
peak163	chr1	36702007	36702167	1	NM_0011	SH3D21	69907	NM_0012	TRAPPC3	80433	intronic	THRAP3	4.65291	Down	NULL	PBS_peak_439
peak164	chr1	36706523	36706677	1	NM_0011	SH3D21	65394	NM_0012	TRAPPC3	84946	intronic	THRAP3	4.63076	Down	NULL	PBS_peak_444
peak165	chr1	40330398	40330582	17	NM_0011	MFSD2A	90294	NM_0017	BMP8B	75957	intronic	TRIT1	8.17214	Down	NULL	NULL
peak166	chr1	40336369	40336527	17	NM_0011	MFSD2A	84336	NM_0017	BMP8B	81915	intronic	TRIT1	6.18954	Down	NULL	PBS_peak_468
peak167	chr1	40337591	40337777	17	NM_0011	MFSD2A	83100	NM_0017	BMP8B	83151	intronic	TRIT1	4.34213	Down	NULL	PBS_peak_469
peak168	chr1	40338992	40339180	17	NM_0011	MFSD2A	81698	NM_0017	BMP8B	84553	intronic	TRIT1	9.4896	Down	NULL	PBS_peak_470
peak169	chr1	40339337	40339532	17	NM_0011	MFSD2A	81349	NM_0017	BMP8B	84901	intronic	TRIT1	6.98121	Down	NULL	PBS_peak_471
peak170	chr1	40340875	40341059	17	NM_0011	MFSD2A	79817	NM_0017	BMP8B	86434	intronic	TRIT1	6.96821	Down	NULL	PBS_peak_472
peak171	chr1	40341660	40341818	17	NM_0011	MFSD2A	79045	NM_0017	BMP8B	87206	intronic	TRIT1	4.69093	Down	NULL	PBS_peak_473
peak172	chr1	40341904	40342091	17	NM_0011	MFSD2A	78786	NM_0017	BMP8B	87464	intronic	TRIT1	6.59701	Down	NULL	PBS_peak_473
peak173	chr1	40342746	40342936	17	NM_0011	MFSD2A	77943	NM_0017	BMP8B	88308	intronic	TRIT1	5.62583	Down	NULL	PBS_peak_474
peak174	chr1	40343186	40343504	17	NM_0011	MFSD2A	77439	NM_0017	BMP8B	88812	intronic	TRIT1	5.66606	Down	NULL	PBS_peak_475
peak175	chr1	40343978	40344166	17	NM_0011	MFSD2A	76712	NM_0017	BMP8B	89539	intronic	TRIT1	10.54994	Down	NULL	PBS_peak_476
peak176	chr1	40427885	40428052	5	NM_0011	CAP1	78286	NM_0053	MYCL	60281	intronic	MFSD2A	6.58865	Up	NULL	NULL
peak177	chr1	40428726	40428968	5	NM_0011	CAP1	77408	NM_0053	MYCL	61160	intronic	MFSD2A	15.29891	Up	luks_peak	NULL
peak178	chr1	40644370	40644539	1	NM_0010	TMCO2	69118	NM_0011	PPT1	81312	intronic	RLF	3.98919	Up	luks_peak	NULL
peak179	chr1	40654446	40654630	1	NM_0010	TMCO2	59035	NM_0011	PPT1	91396	intronic	RLF	5.64137	Up	luks_peak	NULL
peak180	chr1	41169780	41169966	5	NM_0013	NFYC	5122	NR_0245	NFYC-AS	11940	intronic	NFYC	4.72313	Down	NULL	PBS_peak_517
peak181	chr1	41197917	41198071	6	NM_0013	NFYC	6514	NR_0245	NFYC-AS	40061	intronic	NFYC	3.19413	Down	NULL	PBS_peak_524
peak182	chr1	41209825	41209985	7	NR_0298	MIR30E	10122	NR_0245	NFYC-AS	51972	intronic	NFYC	5.02621	Down	NULL	PBS_peak_530
peak183	chr1	41448804	41449181	2	NM_0013	CTPS1	5127	NM_1334	CITED4	120974	exonic	CTPS1	7.47675	Down	NULL	PBS_peak_531
peak184	chr1	41449340	41449545	2	NM_0013	CTPS1	4677	NM_1334	CITED4	121424	intronic	CTPS1	6.64494	Down	NULL	PBS_peak_532
peak185	chr1	41450946	41451134	2	NM_0013	CTPS1	3080	NM_1334	CITED4	123022	intronic	CTPS1	5.63724	Down	NULL	PBS_peak_533
peak186	chr1	41453064	41453343	2	NM_0013	CTPS1	916	NM_1334	CITED4	125185	exonic	CTPS1	11.73522	Down	NULL	PBS_peak_535
peak187	chr1	42777864	42778092	4	NM_1736	RIMKLA	68490	NM_0335	GUCA2A	147583	intronic	FOXJ3	5.0429	Down	NULL	PBS_peak_536
peak188	chr1	42778615	42778788	4	NM_1736	RIMKLA	67766	NM_0335	GUCA2A	148306	intronic	FOXJ3	6.37246	Down	NULL	PBS_peak_536
peak189	chr1	42790756	42790934	4	NM_1736	RIMKLA	55623	NM_0335	GUCA2A	160450	intronic	FOXJ3	7.40744	Down	NULL	NULL
peak190	chr1	42796774	42796948	4	NM_1736	RIMKLA	49607	NM_0335	GUCA2A	166466	intronic	FOXJ3	3.8101	Down	NULL	PBS_peak_544
peak191	chr1	43153020	43153173	2	NM_0240	C1orf50	79819	NM_0322	ZMYND1	231158	intronic	YBX1	3.61454	Down	luks_peak	PBS_peak_547
peak192	chr1	43155891	43156123	2	NM_0240	C1orf50	76909	NM_0322	ZMYND1	234069	intronic	YBX1	3.69681	Down	NULL	PBS_peak_547
peak193	chr1	43156284	43156589	2	NM_0240	C1orf50	76479	NM_0322	ZMYND1	234498	intronic	YBX1	5.71491	Down	NULL	PBS_peak_547
peak194	chr1	43156786	43156939	2	NM_0240	C1orf50	76053	NM_0322	ZMYND1	234924	intronic	YBX1	3.74106	Down	luks_peak	PBS_peak_547
peak195	chr1	43158539	43158691	2	NM_0240	C1orf50	74301	NM_0322	ZMYND1	236677	intronic	YBX1	4.21248	Down	luks_peak	PBS_peak_547
peak196	chr1	43158947	43159178	2	NM_0240	C1orf50	73853	NM_0322	ZMYND1	237124	exonic	YBX1	5.29391	Down	luks_peak	PBS_peak_547
peak197	chr1	43160122	43160308	2	NM_0240	C1orf50	72701	NM_0322	ZMYND1	238277	intronic	YBX1	5.0925	Down	NULL	PBS_peak_547
peak198	chr1	43160823	43161020	2	NM_0240	C1orf50	71994	NM_0322	ZMYND1	238983	intronic	YBX1	5.77857	Down	luks_peak	PBS_peak_547
peak199	chr1	43163493	43163738	2	NM_0240	C1orf50	69300	NM_0322	ZMYND1	241677	intronic	YBX1	3.48335	Down	NULL	PBS_peak_547
peak200	chr1	43173529	43173692	0	NM_0240	C1orf50	59305	NM_0322	ZMYND1	251672	intergenic	YBX1(dis	7.151	Down	NULL	PBS_peak_560
peak201	chr1	43413515	43413964	1	NR_0339	SLC2A1-A	10980	NM_1993	SVBP	130680	intronic	SLC2A1	4.67062	Up	luks_peak	NULL
peak202	chr1	43417428	43417677	1	NR_0339	SLC2A1-A	7167	NM_1993	SVBP	134493	intronic	SLC2A1	3.645	Up	luks_peak	NULL
peak203	chr1	44339132	44339328	25	NM_0011	ARTN	59762	NR_1259	LOC1019	146216	intronic	ST3GAL3	14.31574	Down	NULL	PBS_peak_562
peak204	chr1	46053782	46054160	3	NM_0164	TMEM69	99876	NM_0012	PRDX1	65409	intronic	NASP	9.49029	Down	NULL	PBS_peak_564
peak205	chr1	46054473	46055236	3	NM_0164	TMEM69	98992	NM_0012	PRDX1	66292	intronic	NASP	14.24237	Down	NULL	PBS_peak_565

peak206	chr1	46055333	46055522	3	NM_0164	TMEM69	98419	NM_0012	PRDX1	66865	intronic	NASP	5.08543	Down	NULL	PBS_peak_565
peak207	chr1	46055585	46055864	3	NM_0164	TMEM69	98122	NM_0012	PRDX1	67162	intronic	NASP	8.35377	Down	NULL	PBS_peak_565
peak208	chr1	46127296	46127483	1	NM_0164	TMEM69	26457	NR_02676	RPS15AP	15032	intronic	GPBP1L1	6.29816	Down	NULL	PBS_peak_567
peak209	chr1	49716312	49716488	1	NR_12598	LOC10192	6683	NM_0246	BEND5	473759	intronic	AGBL4	14.30885	Down	NULL	PBS_peak_588
peak210	chr1	51720154	51720305	1	NR_12373	TTC39A-A	75096	NM_0070	FAF1	294293	intronic	RNF11	4.6034	Up	NULL	NULL
peak211	chr1	53771477	53771633	4	NR_13192	LOC10537	22350	NM_0023	MAGOH	67273	intronic	LRP8	3.70501	Down	NULL	PBS_peak_617
peak212	chr1	57784551	57784716	1	NR_10436	DAB1-AS	541581	NM_0012	C8B	352820	intronic	DAB1	3.93553	Down	NULL	NULL
peak213	chr1	91421893	91422055	3	NM_0011	CDC7	544430	NR_135038		104779	intronic	ZNF644	3.93181	Down	NULL	NULL
peak214	chr1	91478059	91478313	3	NM_0011	CDC7	488218	NR_135038		160991	intronic	ZNF644	6.93322	Down	NULL	PBS_peak_676
peak215	chr1	91481539	91481718	3	NM_0011	CDC7	484775	NR_135038		164433	intronic	ZNF644	9.81774	Down	NULL	PBS_peak_677
peak216	chr1	93302369	93302519	2	NR_00000	SNORD21	402	NM_0056	EVI5	44483	intronic	FAM69A,	6.07806	Down	NULL	PBS_peak_682
peak217	chr1	93304955	93305116	2	NR_00244	SNORA66	1240	NM_0056	EVI5	47074	intronic	FAM69A,	5.17125	Down	NULL	PBS_peak_684
peak218	chr1	93549858	93550021	4	NM_0013	CCDC18	96333	NM_0012	FAM69A	122860	intronic	MTF2	6.42546	Down	NULL	NULL
peak219	chr1	99594680	99594862	1	NM_0011	LPPR4	135077	NM_0010	LPPR5	124322	ncRNA_in	LOC10012	3.58437	Down	NULL	PBS_peak_708
peak220	chr1	100511495	100511657	1	NM_0190	TRMT13	87130	NM_0010	FRRS1	280227	intronic	MFSD14A	4.92195	Down	NULL	PBS_peak_710
peak221	chr1	101892135	101892287	0	NR_03342	DNAJA1P	445357	NR_10462	LOC10192	190127	intergenic	LINC0130	4.74543	Down	NULL	NULL
peak222	chr1	102495102	102495253	0	NM_0176	RNPC3	1573400	NR_11021	OLFM3	32387	intergenic	OLFM3(d	5.01008	Up	NULL	NULL
peak223	chr1	106664023	106664223	0	NM_0181	PRMT6	935144	NR_12595	LOC10192	502566	intergenic	LINC0167	3.84743	Up	luks_peak	NULL
peak224	chr1	108372837	108372990	1	NR_10700	MIR7852	66931	NM_0010	VAV3	141787	intronic	VAV3	6.01556	Up	NULL	NULL
peak225	chr1	110106161	110106341	1	NR_02958	MIR197	35264	NM_0207	AMIGO1	53915	intronic	GNAI3	5.08511	Down	NULL	PBS_peak_724
peak226	chr1	110886354	110886609	2	NR_10269	LAMTOR	63949	NR_03659	LOC44060	4688	intronic	RBM15	4.31495	Down	NULL	PBS_peak_730
peak227	chr1	110889485	110889683	0	NR_10269	LAMTOR	60847	NR_03659	LOC44060	7791	downstrea	RBM15	4.90257	Down	NULL	PBS_peak_731
peak228	chr1	110892564	110892805	0	NR_10269	LAMTOR	57746	NR_03659	LOC44060	10891	intergenic	RBM15(di	4.19299	Down	NULL	PBS_peak_733
peak229	chr1	110895726	110895882	0	NR_10269	LAMTOR	54627	NR_03659	LOC44060	14011	intergenic	RBM15(di	5.18185	Down	NULL	NULL
peak230	chr1	110900096	110900262	0	NR_10269	LAMTOR	50252	NR_03659	LOC44060	18386	intergenic	RBM15(di	17.13497	Down	NULL	PBS_peak_735
peak231	chr1	113221752	113221916	3	NM_0010	FAM19A3	41355	NM_1387	ST7L	59794	intronic	MOV10	4.48292	Up	NULL	NULL
peak232	chr1	113222582	113222777	3	NM_0010	FAM19A3	40509	NM_1387	ST7L	60639	intronic	MOV10	4.17534	Up	luks_peak	NULL
peak233	chr1	113224905	113225303	3	NM_0010	FAM19A3	38085	NM_1387	ST7L	63064	intronic	MOV10	4.54071	Up	luks_peak	NULL
peak234	chr1	113228821	113228988	3	NM_0010	FAM19A3	34284	NM_1387	ST7L	66864	intronic	MOV10	8.20949	Up	NULL	NULL
peak235	chr1	113229195	113229557	3	NM_0010	FAM19A3	33813	NM_1387	ST7L	67336	intronic	MOV10	6.60132	Up	luks_peak	NULL
peak236	chr1	113230887	113231100	3	NM_0010	FAM19A3	32195	NM_1387	ST7L	68953	UTR5	MOV10(N	7.94763	Up	NULL	NULL
peak237	chr1	113232107	113232275	3	NM_0010	FAM19A3	30998	NM_1387	ST7L	70151	exonic	MOV10	5.22986	Up	NULL	NULL
peak238	chr1	115280854	115281014	6	NM_0012	SYCP1	116490	NM_0025	NRAS	21419	intronic	CSDE1	6.40797	Down	NULL	PBS_peak_751
peak239	chr1	115288786	115289073	6	NM_0012	SYCP1	108494	NM_0025	NRAS	29414	intronic	CSDE1	3.9731	Down	NULL	PBS_peak_756
peak240	chr1	115291315	115291576	6	NM_0012	SYCP1	105978	NM_0025	NRAS	31930	intronic	CSDE1	5.37592	Down	NULL	PBS_peak_757
peak241	chr1	115291963	115292236	6	NM_0012	SYCP1	105324	NM_0025	NRAS	32584	intronic	CSDE1	5.90344	Down	NULL	PBS_peak_757
peak242	chr1	115292614	115292874	6	NM_0012	SYCP1	104680	NM_0025	NRAS	33229	UTR5	CSDE1(N	4.99782	Down	NULL	PBS_peak_758
peak243	chr1	115292946	115293261	6	NM_0012	SYCP1	104320	NM_0025	NRAS	33588	intronic	CSDE1	8.53675	Down	NULL	PBS_peak_758
peak244	chr1	115293941	115294210	6	NM_0012	SYCP1	103348	NM_0025	NRAS	34560	intronic	CSDE1	7.04334	Down	NULL	PBS_peak_759
peak245	chr1	115294365	115294858	6	NM_0012	SYCP1	102812	NM_0025	NRAS	35096	intronic	CSDE1	9.26056	Down	NULL	PBS_peak_759
peak246	chr1	115295104	115295318	6	NM_0012	SYCP1	102213	NM_0025	NRAS	35696	intronic	CSDE1	14.67222	Down	NULL	PBS_peak_759
peak247	chr1	115295837	115296040	6	NM_0012	SYCP1	101485	NM_0025	NRAS	36423	intronic	CSDE1	4.1039	Down	NULL	PBS_peak_760
peak248	chr1	116919063	116919219	2	NM_0011	ATP1A1	6851	NR_12597	LOC10192	399244	intronic	ATP1A1	5.47669	Down	NULL	NULL
peak249	chr1	116921336	116921522	2	NM_0011	ATP1A1	4563	NR_12597	LOC10192	401532	intronic	ATP1A1	4.97275	Down	NULL	PBS_peak_762
peak250	chr1	116921679	116921877	2	NM_0011	ATP1A1	4214	NR_12597	LOC10192	401881	intronic	ATP1A1	5.49553	Down	NULL	PBS_peak_762
peak251	chr1	120175351	120175511	1	NM_0066	PHGDH	78988	NR_03654	LINC0062	33517	intronic	ZNF697	4.35417	Up	luks_peak	NULL
peak252	chr1	120175991	120176220	1	NM_0066	PHGDH	78313	NR_03654	LINC0062	34191	intronic	ZNF697	31.92681	Up	NULL	NULL
peak253	chr1	143288932	143289102	0	NR_10690	MIR6077	383904	NR_11070	LOC10272	86778	intergenic	LOC10272	7.09229	Down	NULL	NULL
peak254	chr1	154148009	154148259	10	NM_0011	UBAP2L	44514	NM_2073	NUP210L	20542	intronic	TPM3	3.75593	Down	NULL	PBS_peak_811
peak255	chr1	154151696	154151851	9	NM_0011	UBAP2L	40874	NM_0012	TPM3	1076	intronic	TPM3	3.61159	Down	NULL	PBS_peak_814
peak256	chr1	154152209	154152384	9	NM_0011	UBAP2L	40351	NM_0012	TPM3	1599	intronic	TPM3	4.63835	Down	NULL	PBS_peak_814
peak257	chr1	154552476	154552680	0	NM_0018	CKS1B	394540	NM_0175	UBE2Q1	21458	downstrea	ADAR,CH	4.80282	Up	luks_peak	NULL
peak258	chr1	154555596	154555793	5	NM_0018	CKS1B	391423	NM_0175	UBE2Q1	24574	UTR3	ADAR(NM	6.8093	Up	luks_peak	NULL
peak259	chr1	154557247	154557404	5	NM_0018	CKS1B	389792	NM_0175	UBE2Q1	26205	exonic	ADAR	4.00144	Up	luks_peak	NULL
peak260	chr1	154559187	154559395	5	NM_0018	CKS1B	387827	NM_0175	UBE2Q1	28171	intronic	ADAR	4.34917	Up	luks_peak	NULL
peak261	chr1	154562302	154562505	5	NM_0018	CKS1B	384714	NM_0175	UBE2Q1	31283	exonic	ADAR	5.54573	Up	luks_peak	NULL
peak262	chr1	155497571	155497732	1	NR_02702	ASH1L-A	34120	NR_03028	MIR555	181415	intronic	ASH1L	10.1923	Down	NULL	PBS_peak_831
peak263	chr1	155509444	155509667	1	NR_02702	ASH1L-A	22216	NR_03028	MIR555	193319	intronic	ASH1L	4.44976	Down	NULL	PBS_peak_840
peak264	chr1	155520657	155520826	1	NR_02702	ASH1L-A	11030	NR_03028	MIR555	204505	intronic	ASH1L	6.19178	Down	NULL	PBS_peak_847
peak265	chr1	155665126	155665337	5	NR_02411	MSTO2P	50327	NM_1391	YY1AP1	6408	intronic	DAP3	3.62715	Down	NULL	PBS_peak_860
peak266	chr1	155673463	155673716	5	NR_02411	MSTO2P	41969	NM_1391	YY1AP1	14766	intronic	DAP3	5.69212	Down	NULL	PBS_peak_863
peak267	chr1	155674563	155674913	5	NR_02411	MSTO2P	40821	NM_1391	YY1AP1	15915	intronic	DAP3	9.96605	Down	NULL	PBS_peak_864
peak268	chr1	156748267	156748472	1	NM_0010	NTRK1	37172	NR_135008		26129	intronic	PRCC	5.29728	Down	NULL	PBS_peak_876
peak269	chr1	158994915	158995101	2	NM_0211	CADM3	146369	NM_001320010		24906	intronic	IFI16	9.56325	Up	luks_peak	NULL
peak270	chr1	159890045	159890386	3	NR_03884	LINC0113	40798	NM_0123	CFAP45	20309	exonic	TAGLN2	5.59596	Down	NULL	PBS_peak_877
peak271	chr1	159891071	159891329	3	NR_03884	LINC0113	39814	NM_0123	CFAP45	21294	intronic	TAGLN2	8.66612	Down	NULL	PBS_peak_877
peak272	chr1	159891468	159891869	3	NR_03884	LINC0113	39345	NM_0123	CFAP45	21762	intronic	TAGLN2	5.42216	Down	NULL	PBS_peak_878
peak273	chr1	159892629	159892799	3	NR_03884	LINC0113	38300	NM_0123	CFAP45	22808	intronic	TAGLN2	4.7088	Down	NULL	PBS_peak_879
peak274	chr1	165802223	165802397	1	NR_03743	MIR3658	74848	NR_0458	TMCO1	64151	intronic	UCK2	7.59285	Down	NULL	PBS_peak_887
peak275	chr1	165804619	165804802	1	NR_03743	MIR3658	72447	NR_0458	TMCO1	66551	intronic	UCK2	12.25159	Down	NULL	NULL
peak276	chr1	165807884	165808098	1	NR_03743	MIR3658	69167	NR_0458	TMCO1	69832	intronic	UCK2	4.53269	Down	NULL	PBS_peak_891
peak277	chr1	165808490	165808671	1	NR_03743	MIR3658	68577	NR_0458	TMCO1	70421	intronic	UCK2	44.92245	Down	NULL	PBS_peak_892
peak278	chr1	165809686	165810009	1	NR_03743	MIR3658	67310	NR_0458	TMCO1	71688	intronic	UCK2	5.19112	Down	NULL	PBS_peak_893
peak279	chr1	165813434	165813611	1	NR_03743	MIR3658	63635	NR_0458	TMCO1	75363	intronic	UCK2	5.03642	Down	NULL	PBS_peak_894
peak280	chr1	165845130	165845287	1	NR_03743	MIR3658	31949	NR_0458	TMCO1	107049	intronic	UCK2	17.9039	Down	NULL	PBS_peak_897
peak281	chr1	165954200	165954353	0	NR_00292	FMO9P	618876	NR_0458	TMCO1	216117	intergenic	UCK2(dis	4.3619	Down	NULL	PBS_peak_900
peak282	chr1	165991186	165991409	0	NR_00292	FMO9P	581855	NR_0458	TMCO1	253138	intergenic	UCK2(dis	14.16298	Down	NULL	PBS_peak_903
peak283	chr1	166662247	166662407	0	NM_001314014		146314	NM_0010	FAM78B	526335	intergenic	FMO9P(di	3.97951	Down	NULL	PBS_peak_922
peak284	chr1	166669337	166669497	0	NM_001314014		139224	NM_0010	FAM78B	533425	intergenic	FMO9P(di	4.3978	Down	NULL	PBS_peak_923
peak285	chr1	167618894	167619078	1	NM_0011	MPZL1	72201	NM_0038	CREG1	95930	intronic	RCSD1	3.8318	Down	NULL	PBS_peak_929
peak286	chr1	167620202	167620362	1	NM_0011	MPZL1	70905	NM_0038	CREG1	97226	intronic	RCSD1	8.4854	Down	NULL	PBS_peak_930
peak287	chr1	167624313	167624519	1	NM_0011	MPZL1	66771	NM_0038	CREG1	101360	intronic	RCSD1	4.62285	Down	NULL	PBS_peak_932
peak288	chr1	167625774	167626085	1	NM_0011	MPZL1	65257	NM_0038	CREG1	102873	intronic	RCSD1	7.06364	Down	NULL	PBS_peak_934
peak289	chr1	167628097	167628285	1	NM_0011	MPZL1	62996	NM_0038	CREG1	105135	intronic	RCSD1	4.9109	Down	NULL	PBS_peak_935
peak290	chr1	167629356	167629755	1	NM_0011	MPZL1	61631	NM_0038	CREG1	106499	intronic	RCSD1	5.26878	Down	NULL	PBS_peak_936
peak291	chr1	167630019	167630340	1	NM_0011	MPZL1	61007	NM_0038	CREG1	107123	intronic	RCSD1	5.93471	Down	NULL	PBS_peak_936
peak292	chr1	167630662	167630947	1	NM_0011	MPZL1	60382	NM_0038	CREG1	107748	intronic	RCSD1	4.39286	Down	NULL	PBS_peak_937
peak293	chr1	167631103	167631365	1	NM_0011	MPZL1	59953	NM_0038	CREG1	108178	intronic	RCSD1	4.40721	Down	NULL	PBS_peak_937
peak294	chr1	167631900	167632072	1	NM_0011	MPZL1	59201	NM_0038	CREG1	108930	intronic	RCSD1	3.6773	Down	NULL	PBS_peak_937
peak295	chr1	167632937	167633157	1	NM_0011	MPZL1	58140	NM_0038	CREG1	109991	intronic	RCSD1	6.46848	Down	NULL	PBS_peak_938
peak296	chr1	167633785	167634213	1	NM_0011	MPZL1	57188	NM_0038	CREG1	110943	intronic	RCSD1	5.41382	Down	NULL	PBS_peak_938
peak297	chr1	167636456	167636650	1	NM_0011	MPZL1	54634	NM_0038	CREG1	113497	intronic	RCSD1	6.32153	Down	NULL	PBS_peak_940
peak298	chr1	170202492	170202680	0	NM_0011	GORAB	298677	NR_03600	MIR3119-	81983	ncRNA_in	LINC0168	11.41447	Down	NULL	PBS_peak_948
peak299	chr1	171462525	171462723	1	NM_0010	METTL13	288137	NR_03162	MIR1295A	391677	intronic	PRRC2C	4.82491	Down	NULL	PBS_peak_957
peak300	chr1	174973015	174973178	2	NM_0220	TNN	63897	NR_12119	LOC10192	49698	intronic	CACYBP	6.12526	Down	NULL	NULL
peak301	chr1	174975244	174975416	2	NM_0220	TNN	61664	NR_12119	LOC10192	51932	intronic	CACYBP	17.03235	Down	NULL	NULL
peak302	chr1	177108438	177108590	3	NM_0211	BRINP2	32010	NR_03016	MIR488	109933	intronic	ASTN1	5.5608	Down	NULL	NULL
peak303	chr1	182814499	182814666	2	NM_0022	LAMC1	178012	NM_0333	RGS8	172515	intronic	DHX9	10.27453	Down	NULL	PBS_peak_990
peak304	chr1	182818693	182818853	2	NM_0022	LAMC1	173822	NM_0333	RGS8	176706	intronic	DHX9	17.72867	Down	NULL	PBS_peak_993
peak305	chr1	183456282	183456443	4	NM_0012	RGL1	148819	NR_04000	SMG7-AS	15245	intronic	SMG7	6.06533	Down	NULL	PBS_peak_994
peak306	chr1	194921223	194921378	0	NM_0010	CFH	1699707	NM_0037	B3GALT2	1765557	intergenic	LINC0103	21.03702	Down	NULL	NULL
peak307	chr1	195616317	195616484	0	NM_0010	CFH	1004607	NM_0037	B3GALT2	2460657	intergenic	NONE(dis	5.25802	Down	NULL	NULL
peak308	chr1	195952054	195952210	0	NM_0010	CFH	668876	NM_0037	B3GALT2	2796389	intergenic	NONE(dis	6.10353	Down	NULL	PBS_peak_1019

peak309	chr1	204374552	204374747	1	NM_0012	MDM4	110857	NR_02702	LINC0062	35802	UTR3	PPP1R15E	4.31667	Down	NULL	PBS_peak_1046
peak310	chr1	205699629	205699814	1	NR_04609	LOC28458	131485	NM_0331	SLC45A3	50091	intronic	NUCKS1	13.95183	Down	NULL	PBS_peak_1050
peak311	chr1	205703844	205704009	1	NR_04609	LOC28458	127280	NM_0331	SLC45A3	54296	intronic	NUCKS1	5.34737	Down	NULL	PBS_peak_1051
peak312	chr1	205706507	205706661	1	NR_04609	LOC28458	124623	NM_0331	SLC45A3	56954	intronic	NUCKS1	4.63534	Down	NULL	PBS_peak_1052
peak313	chr1	205711640	205711798	1	NR_04609	LOC28458	119488	NM_0331	SLC45A3	62089	intronic	NUCKS1	5.44926	Down	NULL	PBS_peak_1056
peak314	chr1	205713067	205713316	1	NR_04609	LOC28458	118015	NM_0331	SLC45A3	63561	intronic	NUCKS1	22.55881	Down	NULL	PBS_peak_1057
peak315	chr1	205714293	205714554	1	NR_04609	LOC28458	116783	NM_0331	SLC45A3	64793	intronic	NUCKS1	4.56878	Down	NULL	PBS_peak_1058
peak316	chr1	208611669	208611910	0	NR_135085		887011	NM_0251	PLXNA2	194124	intergenic	PLXNA2(	5.66708	Down	NULL	PBS_peak_1100
peak317	chr1	208617489	208617647	0	NR_135085		881233	NM_0251	PLXNA2	199903	intergenic	PLXNA2(	5.60153	Down	NULL	NULL
peak318	chr1	208679876	208680066	0	NR_135085		818830	NM_0251	PLXNA2	262306	intergenic	PLXNA2(	14.84045	Down	NULL	PBS_peak_1108
peak319	chr1	208748747	208748927	0	NR_135085		749964	NM_0251	PLXNA2	331172	intergenic	PLXNA2(	3.78421	Down	NULL	PBS_peak_1122
peak320	chr1	208956728	208956878	0	NR_135085		541998	NM_0251	PLXNA2	539138	intergenic	LINC0171	12.87118	Down	NULL	NULL
peak321	chr1	209104869	209105049	0	NR_135085		393842	NM_0251	PLXNA2	687294	intergenic	LINC0171	23.26887	Down	NULL	PBS_peak_1148
peak322	chr1	210141418	210141578	6	NM_0196	SERTAD4	264697	NM_0061	IRF6	161978	intronic	SYT14	7.86302	Down	NULL	NULL
peak323	chr1	210719165	210719366	6	NM_0011	RCOR3	713442	NR_02433	SERTAD4	311799	intronic	HHAT	3.65164	Down	NULL	PBS_peak_1184
peak324	chr1	210990448	210990651	2	NM_0011	RCOR3	442158	NR_02433	SERTAD4	583083	intronic	KCNH1	4.9844	Down	NULL	PBS_peak_1213
peak325	chr1	211009285	211009446	2	NM_0011	RCOR3	423342	NR_02433	SERTAD4	601899	intronic	KCNH1	6.17599	Down	NULL	PBS_peak_1217
peak326	chr1	211011846	211012020	2	NM_0011	RCOR3	420775	NR_02433	SERTAD4	604467	intronic	KCNH1	6.06483	Down	NULL	PBS_peak_1218
peak327	chr1	211067550	211067703	2	NM_0011	RCOR3	365081	NR_02433	SERTAD4	660160	intronic	KCNH1	4.2426	Down	NULL	PBS_peak_1228
peak328	chr1	211085642	211085799	2	NM_0011	RCOR3	346987	NR_02433	SERTAD4	678254	intronic	KCNH1	3.40197	Down	NULL	PBS_peak_1233
peak329	chr1	213685028	213685205	0	NR_04618	LINC0053	412975	NR_12533	ANGEL2	495899	intergenic	RPS6KC1	6.83834	Down	NULL	PBS_peak_1275
peak330	chr1	213927875	213928028	0	NR_04618	LINC0053	170140	NR_12533	ANGEL2	738734	intergenic	RPS6KC1	4.43406	Down	NULL	NULL
peak331	chr1	214048955	214049191	1	NR_04618	LINC0053	49019	NR_12533	ANGEL2	859856	ncRNA_in	PROX1-A	10.86016	Down	NULL	PBS_peak_1318
peak332	chr1	214140381	214140565	1	NM_0012	PROX1	20805	NR_12533	ANGEL2	951256	ncRNA_in	PROX1-A	4.06321	Down	NULL	PBS_peak_1329
peak333	chr1	214231656	214231833	0	NM_0201	SMYD2	222820	NR_03785	PROX1-A	72248	intergenic	PROX1(di	4.29842	Down	NULL	PBS_peak_1334
peak334	chr1	214236192	214236372	0	NM_0201	SMYD2	218283	NR_03785	PROX1-A	76786	intergenic	PROX1(di	4.18889	Down	NULL	PBS_peak_1335
peak335	chr1	215507749	215507907	0	NM_001319294		232894	NM_0054	PTPN14	782804	intergenic	KCNK2(d	4.84779	Down	NULL	NULL
peak336	chr1	226254447	226254670	2	NM_0012	MIXL1	156760	NM_1526	SDE2	67492	ncRNA_in	H3F3AP4	5.37865	Down	NULL	PBS_peak_1365
peak337	chr1	228274412	228274678	4	NR_03741	MIR3620	10419	NM_0033	WNT9A	138869	intronic	ARF1	4.20597	Down	luks_peak	PBS_peak_1374
peak338	chr1	229755386	229755537	2	NM_001314021		6501	NM_0120	ABCB10	61019	intronic	TAF5L	4.88528	Down	NULL	PBS_peak_1387
peak339	chr1	229778839	229779002	2	NR_135116		359543	NM_0010	TAF5L	17126	intronic	URB2	8.01073	Down	NULL	NULL
peak340	chr1	231541288	231541439	1	NM_0059	TSNAX	123035	NM_1758	EXOC8	67745	intronic	EGLN1	4.10863	Down	NULL	NULL
peak341	chr1	235284364	235284761	1	NM_0010	GGPS1	207190	NR_12594	LOC10192	184816	intronic	TOMM20	9.92477	Down	NULL	PBS_peak_1389
peak342	chr1	235286517	235286881	1	NM_0010	GGPS1	205054	NR_12594	LOC10192	186953	intronic	TOMM20	13.65002	Down	NULL	PBS_peak_1390
peak343	chr1	235287169	235287522	1	NM_0010	GGPS1	204407	NR_12594	LOC10192	187599	intronic	TOMM20	7.77276	Down	NULL	PBS_peak_1391
peak344	chr1	235287768	235287932	1	NM_0010	GGPS1	203903	NR_12594	LOC10192	188104	intronic	TOMM20	3.90564	Down	NULL	PBS_peak_1391
peak345	chr1	235288441	235288608	1	NM_0010	GGPS1	203228	NR_12594	LOC10192	188778	intronic	TOMM20	4.49939	Down	NULL	PBS_peak_1392
peak346	chr1	235422587	235422765	4	NM_0010	GGPS1	69077	NR_03990	MIR4753	69245	intronic	ARID4B	4.94883	Up	luks_peak	NULL
peak347	chr1	235427739	235427905	4	NM_0010	GGPS1	63931	NR_03990	MIR4753	74391	intronic	ARID4B	4.26155	Up	luks_peak	NULL
peak348	chr1	235434512	235434728	4	NM_0010	GGPS1	57133	NR_03990	MIR4753	81189	intronic	ARID4B	6.60696	Up	luks_peak	NULL
peak349	chr1	235439119	235439367	4	NM_0010	GGPS1	52510	NR_03990	MIR4753	85812	intronic	ARID4B	6.01761	Up	luks_peak	NULL
peak350	chr1	235468281	235468681	4	NM_0010	GGPS1	23272	NR_03990	MIR4753	115050	intronic	ARID4B	4.25295	Up	luks_peak	NULL
peak351	chr1	235910400	235910561	2	NM_0032	GPR137B	395351	NM_0044	GNG4	96426	intronic	LYST	14.14943	Down	NULL	NULL
peak352	chr1	236000146	236000462	3	NM_0032	GPR137B	305528	NM_0044	GNG4	186250	intronic	LYST	8.42448	Down	NULL	PBS_peak_1402
peak353	chr1	236001397	236001570	3	NM_0032	GPR137B	304348	NM_0044	GNG4	187429	intronic	LYST	20.21258	Down	NULL	PBS_peak_1403
peak354	chr1	236002027	236002280	3	NM_0032	GPR137B	303678	NM_0044	GNG4	188099	intronic	LYST	9.89281	Down	NULL	PBS_peak_1404
peak355	chr1	236003697	236003916	3	NM_0032	GPR137B	302025	NM_0044	GNG4	189752	intronic	LYST	11.07706	Down	NULL	PBS_peak_1405
peak356	chr1	236004931	236005100	3	NM_0032	GPR137B	300816	NM_0044	GNG4	190961	intronic	LYST	6.20857	Down	NULL	PBS_peak_1407
peak357	chr1	236008315	236008508	3	NM_0032	GPR137B	297420	NM_0044	GNG4	194357	intronic	LYST	7.76995	Down	NULL	PBS_peak_1408
peak358	chr1	236008736	236009252	3	NM_0032	GPR137B	296838	NM_0044	GNG4	194940	intronic	LYST	5.24395	Down	NULL	PBS_peak_1409
peak359	chr1	236010725	236010877	3	NM_0032	GPR137B	295031	NM_0044	GNG4	196747	intronic	LYST	4.99565	Down	NULL	PBS_peak_1410
peak360	chr1	236017715	236018050	3	NM_0032	GPR137B	287949	NR_03171	MIR1537	1522	intronic	LYST	6.5733	Down	NULL	PBS_peak_1413
peak361	chr1	236822860	236823018	0	NM_0011	ACTN2	26815	NM_0180	HEATR1	55098	intergenic	HEATR1(	4.73996	Down	NULL	PBS_peak_1416
peak362	chr1	237306768	237306918	1	NR_03962	MIR4428	327576	NM_0012	MT1HL1	139125	intronic	RYR2	3.51331	Down	NULL	PBS_peak_1441
peak363	chr1	237491277	237491463	1	NR_03962	MIR4428	143049	NM_0012	MT1HL1	323652	intronic	RYR2	6.70843	Down	NULL	PBS_peak_1459
peak364	chr1	237790002	237790155	1	NR_02724	LOC1001	235396	NM_0012	MT1HL1	622360	intronic	RYR2	25.02516	Down	NULL	NULL
peak365	chr1	237803550	237803750	1	NR_02724	LOC1001	221825	NM_0012	MT1HL1	635932	intronic	RYR2	3.41499	Down	NULL	PBS_peak_1478
peak366	chr1	237923955	237924144	1	NR_02724	LOC1001	101425	NM_0012	MT1HL1	756331	intronic	RYR2	6.58414	Down	NULL	PBS_peak_1491
peak367	chr1	239668546	239668701	0	NM_0007	CHRM3	123749	NR_01540	LINC0113	1019306	intronic	CHRM3	7.52961	Down	NULL	PBS_peak_1496
peak368	chr1	240418705	240418857	2	NR_12870	MIR1273I	221628	NR_04658	CHRM3-A	355609	intronic	FMN2	4.11662	Down	NULL	PBS_peak_1515
peak369	chr1	240510458	240510756	2	NR_12870	MIR1273I	129802	NR_04658	CHRM3-A	447435	intronic	FMN2	4.57443	Down	NULL	PBS_peak_1523
peak370	chr1	241194787	241194949	4	NR_03606	MIR3123	100704	NM_0224	GREM2	419406	intronic	RGS7	4.41787	Down	NULL	PBS_peak_1557
peak371	chr1	241372294	241372467	4	NM_0036	KMO	323053	NM_0224	GREM2	596918	intronic	RGS7	20.80487	Down	NULL	PBS_peak_1565
peak372	chr1	242300221	242300410	4	NM_0066	SDCCAG8	1118991	NM_0010	MAP1LC3	137930	intronic	PLD5	5.74842	Down	NULL	PBS_peak_1615
peak373	chr1	242309483	242309649	4	NM_0066	SDCCAG8	1109741	NM_0010	MAP1LC3	147181	intronic	PLD5	10.8305	Down	NULL	PBS_peak_1621
peak374	chr1	242331491	242331682	4	NM_0066	SDCCAG8	1087720	NM_0010	MAP1LC3	169201	intronic	PLD5	5.37803	Down	NULL	PBS_peak_1628
peak375	chr1	242334211	242334407	4	NM_0066	SDCCAG8	1084998	NM_0010	MAP1LC3	171924	intronic	PLD5	7.31229	Down	NULL	PBS_peak_1629
peak376	chr1	242342058	242342220	4	NM_0066	SDCCAG8	1077168	NM_0010	MAP1LC3	179754	intronic	PLD5	5.55587	Down	NULL	PBS_peak_1632
peak377	chr1	242386674	242386835	4	NM_0066	SDCCAG8	1032552	NM_0010	MAP1LC3	224369	intronic	PLD5	5.99247	Down	NULL	NULL
peak378	chr1	242387825	242388034	4	NM_0066	SDCCAG8	1031377	NM_0010	MAP1LC3	225544	intronic	PLD5	5.07163	Down	NULL	PBS_peak_1648
peak379	chr1	242431691	242431868	4	NM_0066	SDCCAG8	987527	NM_0010	MAP1LC3	269394	intronic	PLD5	24.45599	Down	NULL	PBS_peak_1659
peak380	chr1	242587352	242587541	4	NM_0066	SDCCAG8	831860	NM_0010	MAP1LC3	425061	intronic	PLD5	19.3259	Down	NULL	PBS_peak_1670
peak381	chr1	242671034	242671208	2	NM_0066	SDCCAG8	748186	NM_0011	PLD5	58337	intronic	PLD5	5.39579	Down	NULL	PBS_peak_1675
peak382	chr1	242752414	242752602	0	NM_0066	SDCCAG8	666799	NM_1526	PLD5	64510	intergenic	PLD5(dist	4.74277	Down	NULL	PBS_peak_1690
peak383	chr1	242792219	242792387	0	NM_0066	SDCCAG8	627004	NM_1526	PLD5	104305	intergenic	PLD5(dist	6.84437	Down	NULL	PBS_peak_1704
peak384	chr1	247585070	247585326	6	NM_0010	OR2W5	69172	NM_0327	ZNF496	90153	intronic	NLRP3	4.44861	Down	NULL	PBS_peak_1749
peak385	chr1	247588362	247588519	6	NM_0010	OR2W5	65929	NM_0327	ZNF496	93395	exonic	NLRP3	5.30413	Down	NULL	PBS_peak_1751
peak386	chr1	247591462	247591647	6	NM_0010	OR2W5	62815	NM_0327	ZNF496	96509	intronic	NLRP3	4.8115	Down	NULL	PBS_peak_1752
peak387	chr1	247594257	247594446	6	NM_0010	OR2W5	60018	NM_0327	ZNF496	99306	intronic	NLRP3	4.15379	Down	NULL	PBS_peak_1755
peak388	chr1	247603304	247603645	6	NM_0010	OR2W5	50895	NM_0327	ZNF496	108429	intronic	NLRP3	4.70789	Down	NULL	PBS_peak_1760
peak1289	chr10	967781	967969	1	NR_12062	LOC10192	11091	NM_0149	DIP2C	232267	intronic	LARP4B	6.07603	Down	NULL	PBS_peak_1790
peak1290	chr10	973166	973322	1	NR_12062	LOC10192	5722	NM_0149	DIP2C	237636	intronic	LARP4B	4.98897	Down	NULL	PBS_peak_1794
peak1291	chr10	1041677	1041850	1	NR_02462	IDI2-AS1	26813	NM_0151	LARP4B	64118	intronic	GTPBP4	6.80246	Down	NULL	PBS_peak_1798
peak1292	chr10	2383571	2383725	0	NM_0026	PFKP	726064	NR_03888	LINC0070	26380	intergenic	LINC0070	5.95084	Down	NULL	NULL
peak1293	chr10	5814516	5814669	2	NM_0012	FBXO18	116942	NR_03710	ASB13	106034	intronic	GDI2	4.61122	Down	NULL	PBS_peak_1811
peak1294	chr10	5822789	5822952	2	NM_0012	FBXO18	108664	NR_03710	ASB13	114312	intronic	GDI2	12.30055	Down	NULL	NULL
peak1295	chr10	5842189	5842394	2	NM_0012	FBXO18	89243	NR_03710	ASB13	133733	intronic	GDI2	6.73349	Down	NULL	PBS_peak_1819
peak1296	chr10	6135564	6135740	2	NM_0011	PFKFB3	51189	NM_0013	IL2RA	31319	intronic	RBM17	5.26919	Down	NULL	PBS_peak_1830
peak1297	chr10	6214179	6214340	2	NM_001314063		30580	NR_03969	MIR3155	20034	intronic	PFKFB3	13.49426	Down	NULL	NULL
peak1298	chr10	6215826	6216086	2	NM_001314063		28884	NR_03969	MIR3155	21731	intronic	PFKFB3	6.67762	Down	NULL	PBS_peak_1833
peak1299	chr10	7329460	7329649	2	NM_0022	ITIH2	415681	NR_132972		100808	intronic	SFMBT2	3.9373	Down	NULL	PBS_peak_1840
peak1300	chr10	7336213	7336379	2	NM_0022	ITIH2	408940	NR_132972		107550	intronic	SFMBT2	5.78352	Down	NULL	PBS_peak_1844
peak1301	chr10	7342044	7342215	2	NM_0022	ITIH2	403106	NR_132972		113383	intronic	SFMBT2	4.23021	Down	NULL	PBS_peak_1849
peak1302	chr10	7350984	7351198	2	NM_0022	ITIH2	394145	NR_132972		122345	intronic	SFMBT2	4.1947	Down	NULL	PBS_peak_1854
peak1303	chr10	7351363	7351634	2	NM_0022	ITIH2	393737	NR_132972		122752	intronic	SFMBT2	4.37575	Down	NULL	PBS_peak_1855
peak1304	chr10	7355292	7355538	2	NM_0022	ITIH2	389821	NR_132972		126669	intronic	SFMBT2	5.94118	Down	NULL	PBS_peak_1858
peak1305	chr10	7357324	7357505	2	NM_0022	ITIH2	387821	NR_132972		128668	intronic	SFMBT2	4.71513	Down	NULL	PBS_peak_1859
peak1306	chr10	7438028	7438210	2	NM_0022	ITIH2	307117	NR_132972		209373	intronic	SFMBT2	13.37336	Down	NULL	PBS_peak_1880
peak1307	chr10	11232238	11232409	4	NM_0246	ECHDC3	552032	NR_04610	CELF2-AS	84872	intronic	CELF2	9.3458	Down	NULL	PBS_peak_1885
peak1308	chr10	11244483	11244638	4	NM_0246	ECHDC3	539795	NR_04610	CELF2-AS	97109	intronic	CELF2	7.02059	Down	NULL	PBS_peak_1887
peak1309	chr10	11249874	11250048	4	NM_0246	ECHDC3	534395	NR_04610	CELF2-AS	102510	intronic	CELF2	6.50271	Down	NULL	NULL
peak1310	chr10	11254495	11254654	4	NM_0246	ECHDC3	529781	NR_04610	CELF2-AS	107123	intronic	CELF2	5.98951	Down	NULL	PBS_peak_1890
peak1311	chr10	11254925	11255099	4	NM_0246	ECHDC3	529344	NR_04610	CELF2-AS	107561	intronic	CELF2	12.94625	Down	NULL	PBS_peak_1891

peak1312	chr10	11263030	11263386	4	NM_0246	ECHDC3	521148	NR_04610	CELF2-AS	115757	intronic	CELF2	17.8778	Down	NULL	PBS_peak_1892
peak1313	chr10	11289287	11289503	4	NM_0246	ECHDC3	494961	NR_04610	CELF2-AS	141944	intronic	CELF2	7.8993	Down	NULL	PBS_peak_1900
peak1314	chr10	14573390	14573551	17	NM_0012	HSPA14	306688	NR_03618	MIR4293	148194	intronic	FAM107B	7.04117	Down	NULL	PBS_peak_1905
peak1315	chr10	14573923	14574132	17	NM_0012	HSPA14	306131	NR_03618	MIR4293	148751	intronic	FAM107B	5.54369	Down	NULL	PBS_peak_1906
peak1316	chr10	14581792	14581984	15	NM_0012	HSPA14	298271	NM_001320740		1347	intronic	FAM107B	3.98479	Down	NULL	PBS_peak_1910
peak1317	chr10	14585072	14585236	15	NM_0012	HSPA14	295005	NM_001320740		4613	intronic	FAM107B	14.46272	Down	NULL	PBS_peak_1911
peak1318	chr10	14607735	14607894	13	NM_0012	HSPA14	272344	NM_001320738		9635	intronic	FAM107B	9.93713	Down	NULL	PBS_peak_1915
peak1319	chr10	14627852	14628105	4	NM_0012	HSPA14	252180	NM_001320735		13606	intronic	FAM107B	4.509	Down	NULL	PBS_peak_1923
peak1320	chr10	14635974	14636153	4	NM_0012	HSPA14	244095	NM_001320735		21691	intronic	FAM107B	5.48757	Down	NULL	NULL
peak1321	chr10	18953018	18953235	1	NM_0011	MALRD1	384573	NM_1825	NSUN6	12560	intronic	ARL5B	5.43442	Up	luks_peak	NULL
peak1322	chr10	18954337	18954492	1	NM_0011	MALRD1	383285	NM_1825	NSUN6	13848	intronic	ARL5B	14.29661	Up	NULL	NULL
peak1323	chr10	28887065	28887241	3	NM_0123	BAMBI	79271	NR_03380	WAC-AS1	65870	intronic	WAC	6.25849	Down	NULL	PBS_peak_1943
peak1324	chr10	36186406	36186568	0	NM_0529	ANKRD30	1228298	NM_0318	FZD8	256125	intergenic	PCAT5(di	16.98288	Down	NULL	PBS_peak_1952
peak1325	chr10	38800338	38800491	0	NR_04500	ACTR3BP	189312	NR_02720	SEPT7P9	108634	intergenic	LINC0099	12.89087	Up	NULL	NULL
peak1326	chr10	43888702	43888858	6	NR_02669	ZNF487	43794	NM_1453	RASGEF1	126413	intronic	HNRNPF	9.43039	Down	NULL	NULL
peak1327	chr10	52562435	52562586	6	NM_0010	PRKG1	188400	NM_1471	SGMS1	178773	UTR3	A1CF(NM	5.10819	Down	NULL	NULL
peak1328	chr10	52795230	52795384	1	NM_0062	PRKG1	38927	NM_1389	A1CF	149872	intronic	PRKG1	3.65332	Down	NULL	NULL
peak1329	chr10	61107325	61107482	4	NM_0011	CDK1	1430685	NR_13178	CCHE1	171179	intronic	FAM13C	3.86513	Down	NULL	NULL
peak1330	chr10	70719967	70720203	2	NM_0156	KIAA1279	28392	NM_1527	SLC25A16	432805	exonic	DDX21	12.15887	Down	NULL	PBS_peak_1993
peak1331	chr10	73524346	73524535	2	NR_10697	MIR7152	26063	NM_0011	C10orf105	26859	intronic	CDH23,V	5.1078	Down	NULL	PBS_peak_1998
peak1332	chr10	73593585	73593879	3	NM_0042	CHST3	130388	NM_0221	C10orf54	60395	intronic	PSAP	5.59703	Up	luks_peak	NULL
peak1333	chr10	76982270	76982450	4	NR_03822	ZNF503-A	73781	NM_001320843		113390	intronic	VDAC2	8.05553	Down	NULL	PBS_peak_2003
peak1334	chr10	80839866	80840022	1	NM_0057	PPIF	267276	NR_02443	ZMIZ1-AS	12739	intronic	ZMIZ1	7.09228	Up	NULL	NULL
peak1335	chr10	80842106	80842271	1	NM_0057	PPIF	265031	NR_02443	ZMIZ1-AS	14983	intronic	ZMIZ1	6.17458	Up	luks_peak	NULL
peak1336	chr10	80846814	80847315	1	NM_0057	PPIF	260155	NR_02443	ZMIZ1-AS	19859	intronic	ZMIZ1	5.05525	Up	luks_peak	NULL
peak1337	chr10	80847658	80847865	1	NM_0057	PPIF	259458	NR_02443	ZMIZ1-AS	20556	intronic	ZMIZ1	7.16361	Up	luks_peak	NULL
peak1338	chr10	80848082	80848428	1	NM_0057	PPIF	258965	NR_02443	ZMIZ1-AS	21050	intronic	ZMIZ1	6.89618	Up	luks_peak	NULL
peak1339	chr10	80848500	80849007	1	NM_0057	PPIF	258466	NR_02443	ZMIZ1-AS	21548	intronic	ZMIZ1	5.01897	Up	luks_peak	NULL
peak1340	chr10	80850717	80850886	1	NM_0057	PPIF	256418	NR_02443	ZMIZ1-AS	23596	intronic	ZMIZ1	6.70097	Up	NULL	NULL
peak1341	chr10	80851268	80851744	1	NM_0057	PPIF	255714	NR_02443	ZMIZ1-AS	24301	intronic	ZMIZ1	4.58637	Up	luks_peak	NULL
peak1342	chr10	80856666	80857216	1	NM_0057	PPIF	250279	NR_02443	ZMIZ1-AS	29736	intronic	ZMIZ1	6.28725	Up	luks_peak	NULL
peak1343	chr10	80857578	80858120	1	NM_0057	PPIF	249371	NR_02443	ZMIZ1-AS	30644	intronic	ZMIZ1	5.75584	Up	luks_peak	NULL
peak1344	chr10	80858758	80858950	1	NM_0057	PPIF	248366	NR_02443	ZMIZ1-AS	31649	intronic	ZMIZ1	4.4044	Up	luks_peak	NULL
peak1345	chr10	80859560	80859734	1	NM_0057	PPIF	247573	NR_02443	ZMIZ1-AS	32442	intronic	ZMIZ1	7.83896	Up	luks_peak	NULL
peak1346	chr10	80860180	80860422	1	NM_0057	PPIF	246919	NR_02443	ZMIZ1-AS	33096	intronic	ZMIZ1	10.82254	Up	luks_peak	NULL
peak1347	chr10	80860806	80860970	1	NM_0057	PPIF	246332	NR_02443	ZMIZ1-AS	33683	intronic	ZMIZ1	4.13526	Up	luks_peak	NULL
peak1348	chr10	80861275	80861552	1	NM_0057	PPIF	245806	NR_02443	ZMIZ1-AS	34208	intronic	ZMIZ1	5.72811	Up	luks_peak	NULL
peak1349	chr10	80864424	80864579	1	NM_0057	PPIF	242718	NR_02443	ZMIZ1-AS	37296	intronic	ZMIZ1	4.29754	Up	NULL	NULL
peak1350	chr10	80867198	80867353	1	NM_0057	PPIF	239944	NR_02443	ZMIZ1-AS	40070	intronic	ZMIZ1	4.88292	Up	luks_peak	NULL
peak1351	chr10	80867607	80867757	1	NM_0057	PPIF	239538	NR_02443	ZMIZ1-AS	40477	intronic	ZMIZ1	5.74017	Up	luks_peak	NULL
peak1352	chr10	80867949	80868105	1	NM_0057	PPIF	239193	NR_02443	ZMIZ1-AS	40822	intronic	ZMIZ1	3.64974	Up	luks_peak	NULL
peak1353	chr10	80869722	80869920	1	NM_0057	PPIF	237399	NR_02443	ZMIZ1-AS	42616	intronic	ZMIZ1	5.62248	Up	luks_peak	NULL
peak1354	chr10	80870334	80870488	1	NM_0057	PPIF	236809	NR_02443	ZMIZ1-AS	43206	intronic	ZMIZ1	4.06539	Up	luks_peak	NULL
peak1355	chr10	80902721	80902871	1	NM_0057	PPIF	204424	NR_02443	ZMIZ1-AS	75591	intronic	ZMIZ1	7.1962	Up	NULL	NULL
peak1356	chr10	86468760	86468917	0	NR_12067	LOC10192	723780	NM_0156	LRIT1	467621	intergenic	CCSER2(	4.58007	Down	NULL	PBS_peak_2014
peak1357	chr10	91097973	91098165	4	NM_0010	IFIT1B	39744	NM_0012	LIPA	86273	intronic	IFIT3	35.9356	Up	luks_peak	NULL
peak1358	chr10	91159447	91159663	5	NM_0124	IFIT5	14770	NM_0012	LIPA	147759	UTR5	IFIT1(NM	14.96826	Up	NULL	NULL
peak1359	chr10	91162101	91162364	5	NM_0124	IFIT5	12092	NM_0012	LIPA	150436	exonic	IFIT1	20.77346	Up	NULL	NULL
peak1360	chr10	91162438	91162696	5	NM_0124	IFIT5	11758	NM_0012	LIPA	150771	exonic	IFIT1	28.08947	Up	NULL	NULL
peak1361	chr10	98367222	98367402	1	NM_0011	LCOR	224705	NM_0201	TM9SF3	20503	intronic	PIK3AP1	4.23504	Up	NULL	NULL
peak1362	chr10	101923336	101923527	2	NM_0050	SCD	183340	NM_0013	CPN1	81789	intronic	ERLIN1	12.8652	Down	NULL	PBS_peak_2046
peak1363	chr10	103894261	103894412	3	NM_0012	NOLC1	17596	NM_0011	LDB1	14126	intronic	PPRC1	10.39074	Down	NULL	PBS_peak_2050
peak1364	chr10	104015899	104016076	3	NM_0012	NFKB2	137879	NM_0050	PITX3	14756	intronic	GBF1	13.05793	Down	NULL	PBS_peak_2055
peak1365	chr10	106766616	106766772	1	NR_134331		1133229	NR_10402	SORCS3-	340720	intronic	SORCS3	4.1013	Up	NULL	NULL
peak1366	chr10	107221938	107222103	0	NR_134331		677902	NR_10402	SORCS3-	796046	intergenic	SORCS3(	5.65241	Down	NULL	NULL
peak1367	chr10	110129598	110129756	0	NM_001320591		1626431	NR_12576	LINC0143	300626	intergenic	LINC0143	10.53984	Down	NULL	PBS_peak_2070
peak1368	chr10	111492552	111492712	0	NM_001320591		263476	NR_12576	LINC0143	1663581	intergenic	LINC0143	3.50986	Down	NULL	NULL
peak1369	chr10	114775600	114775768	13	NR_132769		29430	NM_0224	ZDHHC6	568967	intronic	TCF7L2	31.0602	Up	NULL	NULL
peak1370	chr10	116823009	116823174	0	NM_0012	ATRNL1	30032	NM_0010	ABLIM1	378677	intergenic	LOC1027	9.45676	Down	NULL	PBS_peak_2103
peak1371	chr10	116885838	116886002	3	NM_1985	CCDC172	1198020	NM_0010	ABLIM1	441506	intronic	ATRNL1	6.29478	Down	NULL	PBS_peak_2104
peak1372	chr10	117040210	117040365	1	NM_1985	CCDC172	1043652	NM_0010	ABLIM1	595873	intronic	ATRNL1	22.12601	Down	NULL	PBS_peak_2105
peak1373	chr10	117317776	117317930	1	NM_1985	CCDC172	766087	NM_0010	ABLIM1	873439	intronic	ATRNL1	21.88752	Down	NULL	NULL
peak1374	chr10	119387346	119387512	0	NR_02694	CASC2	418903	NR_00279	EMX2OS	82850	intergenic	EMX2(dis	3.97249	Down	NULL	NULL
peak1375	chr10	120806713	120806882	1	NM_0013	FAM45A	56779	NM_1538	CACUL1	292039	intronic	EIF3A	21.6842	Down	NULL	NULL
peak1376	chr10	120830672	120830831	1	NM_0013	FAM45A	32825	NR_00291	SNORA19	11101	intronic	EIF3A	5.68283	Down	NULL	PBS_peak_2132
peak1377	chr10	121627992	121628151	3	NM_0071	SEC23IP	24013	NM_0032	TIAL1	271530	intronic	MCMBP	16.7105	Down	NULL	PBS_peak_2138
peak1378	chr10	121829528	121829715	0	NM_0010	PPAPDC1	386844	NM_0012	MCMBP	196481	intergenic	MIR4682(	4.29722	Down	NULL	PBS_peak_2139
peak1379	chr10	124310167	124310346	0	NM_001320644		9924	NM_0176	NSMCE4	575513	intergenic	HTRA1(d	16.80239	Down	NULL	PBS_peak_2147
peak1380	chr10	126755404	126755566	4	NR_02330	TEX36-AS	507455	NR_03617	MIR4296	34046	intronic	CTBP2	4.8675	Down	NULL	PBS_peak_2154
peak1381	chr10	126805060	126805265	4	NR_02330	TEX36-AS	457777	NR_03617	MIR4296	83723	intronic	CTBP2	5.83429	Down	NULL	PBS_peak_2155
peak1382	chr10	130444124	130444310	0	NM_0024	MGMT	821231	NM_0024	MKI67	519749	intergenic	LINC0116	5.38246	Down	NULL	PBS_peak_2168
peak1383	chr10	133791069	133791276	1	NM_0011	JAKMIP3	127140	NR_03830	LINC0116	168637	intronic	BNIP3	12.87822	Up	luks_peak	NULL
peak1384	chr11	581785	581970	4	NM_0007	DRD4	55427	NR_02690	LOC14360	5992	exonic	PHRF1	4.30009	Down	NULL	PBS_peak_2190
peak1385	chr11	3005009	3005212	1	NR_02713	MRGPRG	234451	NR_00298	SNORA54	19987	intronic	NAP1L4	5.47976	Down	NULL	PBS_peak_2203
peak1386	chr11	4911051	4911201	0	NM_0010	OR51A7	17474	NM_0010	OR51S1	40688	intergenic	OR51T1(	16.88893	Down	NULL	PBS_peak_2214
peak1387	chr11	5375789	5375978	2	NM_0010	OR51M1	34723	NM_0331	OR51B2	30301	intronic	OR51B5	4.77526	Down	NULL	PBS_peak_2217
peak1388	chr11	5717358	5717757	2	NM_0010	OR56B1	40120	NM_0330	TRIM5	11218	exonic	TRIM22	6.4808	Up	NULL	NULL
peak1389	chr11	5718512	5718681	2	NM_0010	OR56B1	39081	NM_0330	TRIM5	12257	exonic	TRIM22	6.94861	Up	NULL	NULL
peak1390	chr11	9245879	9246066	2	NM_0063	IPO7	160196	NM_0209	SCUBE2	132822	intronic	DENND5	6.18697	Up	NULL	NULL
peak1391	chr11	9263728	9264123	2	NM_0063	IPO7	142243	NM_0209	SCUBE2	150775	intronic	DENND5	10.42934	Up	luks_peak	NULL
peak1392	chr11	9420441	9420796	1	NR_00290	SNORA23	29694	NM_0011	TMEM41	84303	intronic	IPO7	5.31564	Down	NULL	PBS_peak_2238
peak1393	chr11	16519792	16519945	0	NM_0142	C11orf58	240279	NM_0333	SOX6	21933	intergenic	SOX6(dist	4.16966	Down	NULL	NULL
peak1394	chr11	17659961	17660110	2	NM_0024	MYOD1	81109	NR_12373	USH1C	94037	exonic	OTOG	3.40417	Down	NULL	PBS_peak_2242
peak1395	chr11	19027178	19027362	0	NM_0010	ZDHHC13	111422	NM_1471	MRGPRX	70721	intergenic	MRGPRX	10.07058	Down	NULL	PBS_peak_2261
peak1396	chr11	20056465	20056615	5	NM_0064	HTATIP2	328691	NR_03984	MIR4694	274911	intronic	NAV2	24.50024	Down	NULL	PBS_peak_2269
peak1397	chr11	20763190	20763371	4	NM_0011	ANO5	1451441	NM_0010	DBX1	581410	intronic	NELL1	6.99742	Down	NULL	PBS_peak_2270
peak1398	chr11	21183788	21183948	4	NM_0011	ANO5	1030854	NM_0010	DBX1	1001998	intronic	NELL1	5.59551	Down	NULL	PBS_peak_2275
peak1399	chr11	29728141	29728323	0	NM_0005	FSHB	524331	NR_10703	MIR8068	1229137	intergenic	MIR8068(	12.6594	Down	NULL	PBS_peak_2283
peak1400	chr11	31591035	31591209	3	NR_03397	DKFZp686	246992	NM_1449	IMMP1L	59947	intronic	ELP4	5.97176	Down	NULL	PBS_peak_2286
peak1401	chr11	34078462	34078645	2	NM_0011	NAT10	48557	NM_0055	LMO2	164717	intronic	CAPRIN1	11.64982	Down	NULL	PBS_peak_2294
peak1402	chr11	34083878	34084055	2	NM_0011	NAT10	43144	NM_0055	LMO2	170130	intronic	CAPRIN1	6.30579	Down	NULL	PBS_peak_2297
peak1403	chr11	34092996	34093455	2	NM_0011	NAT10	33885	NM_0055	LMO2	179389	exonic	CAPRIN1	13.95143	Down	NULL	PBS_peak_2303
peak1404	chr11	34284967	34285137	1	NM_0017	CAT	175420	NM_0055	LMO2	371216	intronic	ABTB2	16.1709	Up	NULL	NULL
peak1405	chr11	46372827	46372987	6	NM_0011	DGKZ	10238	NM_0166	PHF21A	229922	intronic	DGKZ	26.61444	Down	NULL	PBS_peak_2313
peak1406	chr11	46398616	46398801	7	NM_0012	MDK	3625	NM_0166	PHF21A	255723	exonic	DGKZ	4.96564	Down	NULL	PBS_peak_2318
peak1407	chr11	47530987	47531162	2	NM_0011	PTPMT1	55907	NM_1987	CELF1	15001	intronic	CELF1	3.87148	Down	NULL	PBS_peak_2324
peak1408	chr11	47532497	47532743	2	NM_0011	PTPMT1	54362	NM_1987	CELF1	16547	intronic	CELF1	4.05209	Down	NULL	PBS_peak_2326
peak1409	chr11	47542135	47542312	2	NM_0011	PTPMT1	44758	NM_1987	CELF1	26150	intronic	CELF1	7.39057	Down	NULL	PBS_peak_2330
peak1410	chr11	47558121	47558275	1	NM_0011	PTPMT1	28784	NM_0011	CELF1	12658	intronic	CELF1	11.75318	Down	NULL	NULL
peak1411	chr11	47561088	47561326	1	NM_0011	PTPMT1	25775	NM_0011	CELF1	15667	intronic	CELF1	10.11182	Down	NULL	PBS_peak_2339
peak1412	chr11	47568920	47569114	1	NM_0011	PTPMT1	17965	NM_0011	CELF1	23477	intronic	CELF1	3.96054	Down	NULL	PBS_peak_2345
peak1413	chr11	48386120	48386308	0	NM_0010	OR4A47	124131	NM_0010	OR4C45	12215	downstrea	OR4C5	9.69824	Down	NULL	PBS_peak_2346
peak1414	chr11	51586817	51587002	0	NM_0241	TRIM48	3442748	NM_0010	OR4A5	174461	intergenic	OR4C46(	5.62128	Down	NULL	PBS_peak_2347

peak1415	chr11	55563267	55563431	1	NM_0010	OR5L1	15594	NM_0010	OR4C11	191475	exonic	OR5D14	14.54062	Down	NULL	NULL
peak1416	chr11	56046869	56047040	0	NM_0010	OR8K3	38828	NM_0010	OR5T2	46293	intergenic	OR5T1(di	5.1439	Down	NULL	NULL
peak1417	chr11	57439204	57439379	1	NM_0011	TMX2	40703	NM_1450	YPEL4	21874	intronic	ZDHHC5	4.29486	Down	NULL	PBS_peak_2351
peak1418	chr11	57448667	57448853	1	NM_0011	TMX2	31235	NM_1450	YPEL4	31343	intronic	ZDHHC5	16.91602	Down	NULL	PBS_peak_2354
peak1419	chr11	60904580	60904766	1	NM_0010	PGA3	66312	NR_02739	SLC15A3	185416	intronic	VPS37C	3.77702	Up	NULL	NULL
peak1420	chr11	60909334	60909531	1	NM_0010	PGA3	61552	NR_02739	SLC15A3	190175	intronic	VPS37C	4.09357	Up	luks_peak	NULL
peak1421	chr11	60912849	60913015	1	NM_0010	PGA3	58053	NR_02739	SLC15A3	193675	intronic	VPS37C	3.91093	Up	luks_peak	NULL
peak1422	chr11	63433950	63434232	2	NM_0012	RTN3	14831	NM_0011	PLA2G16	52150	intronic	ATL3	4.47176	Down	NULL	PBS_peak_2376
peak1423	chr11	63614444	63614633	4	NM_0174	MARK2	41448	NM_0011	C11orf95	78425	intronic	MARK2	8.04318	Down	NULL	PBS_peak_2380
peak1424	chr11	63616585	63616747	4	NM_0174	MARK2	39321	NM_0011	C11orf95	80553	intronic	MARK2	5.39727	Down	NULL	PBS_peak_2381
peak1425	chr11	63617111	63617265	4	NM_0174	MARK2	38799	NM_0011	C11orf95	81075	intronic	MARK2	6.14677	Down	NULL	PBS_peak_2382
peak1426	chr11	63632073	63632298	4	NM_0174	MARK2	23801	NM_0011	C11orf95	96072	intronic	MARK2	7.34635	Down	NULL	PBS_peak_2385
peak1427	chr11	63661457	63661648	5	NM_0247	NAA40	44889	NM_0011	C11orf95	125439	intronic	MARK2	5.58794	Down	NULL	PBS_peak_2397
peak1428	chr11	63962617	63962884	3	NM_0314	FERMT3	11401	NM_0140	MACROD	29165	intronic	STIP1	6.9211	Down	NULL	PBS_peak_2399
peak1429	chr11	64542940	64543114	8	NM_0062	PPP2R5B	149116	NM_0056	PYGM	14840	intronic	SF1	6.90068	Down	NULL	PBS_peak_2405
peak1430	chr11	65623294	65623448	1	NM_0251	MUS81	4501	NR_10808	OVOL1-A	65032	exonic	CFL1	5.31816	Down	NULL	PBS_peak_2407
peak1431	chr11	66389323	66389476	5	NM_0011	RBM4	16688	NM_0182	CCDC87	28845	intronic	RBM14,R	4.35091	Down	NULL	PBS_peak_2410
peak1432	chr11	66391084	66391401	5	NM_0011	RBM4	14845	NM_0182	CCDC87	30688	intronic	RBM14,R	6.10726	Down	NULL	PBS_peak_2411
peak1433	chr11	66391606	66391760	5	NM_0011	RBM4	14405	NM_0182	CCDC87	31129	exonic	RBM14	6.04625	Down	NULL	PBS_peak_2411
peak1434	chr11	66394655	66394930	5	NM_0011	RBM4	11295	NM_0182	CCDC87	34238	UTR3	RBM14(N	4.68915	Down	NULL	PBS_peak_2413
peak1435	chr11	66400704	66400894	2	NM_0011	RBM4	5289	NM_0182	CCDC87	40245	intronic	RBM14-R	13.15913	Down	NULL	PBS_peak_2419
peak1436	chr11	67867004	67867178	2	NM_0012	LRP5	212986	NM_0309	UNC93B1	95496	intronic	CHKA	10.56198	Down	NULL	NULL
peak1437	chr11	67872010	67872181	2	NM_0012	LRP5	207981	NM_0309	UNC93B1	100500	intronic	CHKA	4.58004	Down	NULL	NULL
peak1438	chr11	67976233	67976386	5	NM_0012	LRP5	103767	NM_2124	CHKA	87451	intronic	KMT5B	6.90226	Down	NULL	NULL
peak1439	chr11	68235804	68236012	6	NM_0159	GAL	216035	NM_0223	C11orf24	196439	intronic	PPP6R3	3.3175	Down	NULL	PBS_peak_2464
peak1440	chr11	68244646	68244828	6	NM_0159	GAL	207206	NM_0223	C11orf24	205268	intronic	PPP6R3	5.98685	Down	NULL	PBS_peak_2468
peak1441	chr11	68259426	68259626	6	NM_0159	GAL	192417	NM_0223	C11orf24	220057	intronic	PPP6R3	5.35634	Down	NULL	PBS_peak_2481
peak1442	chr11	68275895	68276127	6	NM_0159	GAL	175932	NM_0223	C11orf24	236542	intronic	PPP6R3	5.09761	Down	NULL	PBS_peak_2492
peak1443	chr11	68282531	68282687	6	NM_0159	GAL	169334	NM_0223	C11orf24	243140	intronic	PPP6R3	5.91472	Down	NULL	PBS_peak_2495
peak1444	chr11	70052414	70052584	1	NM_1774	PPFIA1	64307	NR_10383	ANO1-AS	131045	exonic	FADD	25.77403	Down	NULL	NULL
peak1445	chr11	72933803	72933991	3	NM_0012	P2RY6	41653	NM_0148	FCHSD2	80754	intronic	P2RY2	14.2545	Down	NULL	PBS_peak_2498
peak1446	chr11	72935455	72935687	3	NM_0012	P2RY6	39979	NM_0148	FCHSD2	82428	intronic	P2RY2	8.85137	Down	NULL	PBS_peak_2500
peak1447	chr11	72936556	72937012	3	NM_0012	P2RY6	38766	NM_0148	FCHSD2	83641	intronic	P2RY2	8.36397	Down	NULL	PBS_peak_2502
peak1448	chr11	72938641	72938869	3	NM_0012	P2RY6	36795	NM_0148	FCHSD2	85612	intronic	P2RY2	7.98829	Down	NULL	PBS_peak_2503
peak1449	chr11	72939873	72940065	3	NM_0012	P2RY6	35581	NM_0148	FCHSD2	86826	intronic	P2RY2	14.54532	Down	NULL	PBS_peak_2504
peak1450	chr11	72943171	72943332	3	NM_0012	P2RY6	32298	NM_0148	FCHSD2	90108	intronic	P2RY2	5.5121	Down	NULL	NULL
peak1451	chr11	72944905	72945094	3	NM_0012	P2RY6	30550	NM_0148	FCHSD2	91856	UTR5	P2RY2(NI	19.45435	Down	NULL	PBS_peak_2505
peak1452	chr11	74491879	74492092	1	NM_0147	SPCS2	168306	NM_0154	CHRDL2	49555	intronic	RNF169	13.71432	Down	NULL	PBS_peak_2508
peak1453	chr11	83850057	83850308	5	NM_0011	TMEM126	1489434	NM_0011	DLG2	456714	intronic	DLG2	11.01707	Down	NULL	PBS_peak_2518
peak1454	chr11	87849199	87849355	1	NR_03612	MIR3166	60393	NR_135090		418974	intronic	RAB38	8.85436	Down	NULL	PBS_peak_2524
peak1455	chr11	93895087	93895257	1	NM_0011	IZUMO1R	143631	NM_0011	VSTM5	311504	intronic	PANX1	7.10194	Up	NULL	NULL
peak1456	chr11	93899607	93899758	1	NM_0011	IZUMO1R	139120	NM_0011	VSTM5	316014	intronic	PANX1	3.99747	Up	NULL	NULL
peak1457	chr11	93902252	93902413	1	NM_0011	IZUMO1R	136470	NM_0011	VSTM5	318664	intronic	PANX1	6.96952	Up	luks_peak	NULL
peak1458	chr11	93903680	93903923	1	NM_0011	IZUMO1R	135001	NM_0011	VSTM5	320133	intronic	PANX1	9.49276	Up	luks_peak	NULL
peak1459	chr11	93909009	93909164	1	NM_0011	IZUMO1R	129716	NM_0011	VSTM5	325418	intronic	PANX1	5.01561	Up	NULL	NULL
peak1460	chr11	102290832	102291006	1	NR_135053		32731	NM_1781	ANGPTL5	503666	intronic	TMEM123	5.88876	Up	NULL	NULL
peak1461	chr11	102296337	102296600	1	NR_135053		27181	NM_1781	ANGPTL5	509215	intronic	TMEM123	5.13122	Up	luks_peak	NULL
peak1462	chr11	106475471	106475636	0	NM_0011	ELMOD1	986263	NR_135099		472710	intergenic	LOC10192	7.59058	Down	NULL	PBS_peak_2537
peak1463	chr11	112616317	112616469	0	NM_0010	NCAM1	215576	NR_10415	LOC3878	189868	intergenic	LOC3878	6.47649	Down	NULL	NULL
peak1464	chr11	114984150	114984313	0	NR_135108		220064	NM_0176	NXPE4	517747	intergenic	NXPE2(di	6.19357	Down	NULL	PBS_peak_2577
peak1465	chr11	117017166	117017373	8	NM_0010	SIDT2	32669	NM_0251	SIK3	48138	intronic	PAFAH1E	11.41222	Down	NULL	PBS_peak_2580
peak1466	chr11	119087816	119088031	1	NM_0320	RNF26	117286	NM_0011	CCDC153	21339	intronic	CBL	5.13839	Down	NULL	PBS_peak_2584
peak1467	chr11	121015719	121015891	1	NM_0069	SC5D	147583	NR_133004		187057	intronic	TECTA	4.69943	Down	NULL	PBS_peak_2590
peak1468	chr11	126668967	126669198	3	NR_04698	KIRREL3-	141559	NR_03383	ST3GAL4	443600	intronic	KIRREL3	4.76485	Down	NULL	PBS_peak_2602
peak1469	chr11	127051799	127051954	0	NR_12058	LOC10192	89088	NM_0011	KIRREL3	181110	intergenic	LOC10192	4.25895	Down	NULL	PBS_peak_2623
peak1470	chr11	127066746	127066926	0	NR_12058	LOC10192	74129	NM_0011	KIRREL3	196070	intergenic	LOC10192	10.28173	Down	NULL	PBS_peak_2627
peak1471	chr11	127279851	127280003	0	NR_10673	MIR6090	1112358	NM_0011	KIRREL3	409161	intergenic	LOC10192	14.56847	Down	NULL	PBS_peak_2640
peak1472	chr11	127283144	127283353	0	NR_10673	MIR6090	1109036	NM_0011	KIRREL3	412482	intergenic	LOC10192	10.13166	Down	NULL	PBS_peak_2641
peak1473	chr11	127616936	127617154	0	NR_10673	MIR6090	775240	NM_0011	KIRREL3	746279	intergenic	LOC10192	6.04826	Down	NULL	PBS_peak_2668
peak1474	chr11	128567061	128567294	4	NM_0008	KCNJ5	194135	NR_03890	SENCR	1259	intronic	FLI1	4.07265	Down	NULL	PBS_peak_2678
peak1475	chr11	128571514	128571664	4	NM_0008	KCNJ5	189724	NR_03890	SENCR	5671	intronic	FLI1	11.16585	Down	NULL	NULL
peak1476	chr11	129945608	129945799	7	NM_0219	ST14	83978	NM_1994	PRDM10	72973	intronic	APLP2	3.88628	Down	NULL	PBS_peak_2682
peak1477	chr11	129949173	129949340	7	NM_0219	ST14	80425	NM_1994	PRDM10	76526	intronic	APLP2	5.15404	Down	NULL	PBS_peak_2685
peak1478	chr11	129950215	129950369	7	NM_0219	ST14	79390	NM_1994	PRDM10	77562	intronic	APLP2	3.85507	Down	NULL	PBS_peak_2686
peak1479	chr11	129954504	129955290	7	NM_0219	ST14	74785	NM_1994	PRDM10	82167	intronic	APLP2	6.33919	Down	NULL	PBS_peak_2691
peak1480	chr11	129959002	129959582	7	NM_0219	ST14	70390	NM_1994	PRDM10	86562	intronic	APLP2	5.46076	Down	NULL	PBS_peak_2692
peak1481	chr11	129968025	129968191	7	NM_0219	ST14	61574	NM_1994	PRDM10	95378	intronic	APLP2	12.23544	Down	NULL	PBS_peak_2696
peak1482	chr11	129971680	129971919	7	NM_0219	ST14	57882	NM_1994	PRDM10	99069	intronic	APLP2	17.30289	Down	NULL	PBS_peak_2699
peak1483	chr11	131207082	131207327	0	NM_0010	NTM	33166	NM_0147	SNX19	420822	intergenic	SNX19(di	3.84747	Down	NULL	PBS_peak_2739
peak1484	chr11	131219612	131219787	0	NM_0010	NTM	20671	NM_0147	SNX19	433317	intergenic	SNX19(di	6.88579	Down	NULL	PBS_peak_2742
peak1485	chr11	131420068	131420229	1	NM_0011	NTM	360563	NR_11014	LOC10192	9387	intronic	NTM	7.31128	Down	NULL	NULL
peak1486	chr11	131484115	131484283	1	NM_0011	NTM	296513	NR_11014	LOC10192	73438	intronic	NTM	4.46399	Down	NULL	PBS_peak_2764
peak1487	chr11	131661526	131661679	1	NM_0011	NTM	119109	NR_12615	C11orf39	128152	intronic	NTM	4.27179	Down	NULL	PBS_peak_2766
peak1488	chr11	133379006	133379167	2	NR_02727	LOC10012	523080	NM_001319105		565423	intronic	OPCML	18.47917	Down	NULL	NULL
peak1489	chr11	133451185	133451367	0	NR_02727	LOC10012	450891	NM_001319104		48769	intergenic	OPCML(d	5.03283	Down	NULL	PBS_peak_2824
peak1490	chr12	31458912	31459075	3	NR_02680	FLJ13224	18256	NR_03892	DDX11-A	232212	intronic	FAM60A	8.7902	Down	NULL	NULL
peak1491	chr12	31459716	31460027	3	NR_02680	FLJ13224	17378	NR_03892	DDX11-A	233090	intronic	FAM60A	9.77469	Down	NULL	PBS_peak_2830
peak1492	chr12	31467633	31467826	3	NR_02680	FLJ13224	9520	NR_03892	DDX11-A	240948	intronic	FAM60A	5.19619	Down	NULL	PBS_peak_2835
peak1493	chr12	31472187	31472382	3	NR_02680	FLJ13224	4965	NR_03892	DDX11-A	245503	intronic	FAM60A	4.27174	Down	NULL	PBS_peak_2837
peak1494	chr12	31473208	31473397	3	NR_02680	FLJ13224	3947	NR_03892	DDX11-A	246521	intronic	FAM60A	4.95534	Down	NULL	PBS_peak_2838
peak1495	chr12	53409226	53409396	2	NM_1983	TNS2	31499	NR_04596	KRT8	65661	intronic	EIF4B	5.87429	Down	NULL	PBS_peak_2848
peak1496	chr12	60661639	60661800	0	NM_0012	USP15	1992401	NM_1533	LRIG3	1347400	intergenic	SLC16A7	24.08833	Up	NULL	NULL
peak1497	chr12	61921728	61921893	0	NM_0012	USP15	732310	NM_1533	LRIG3	2607491	intergenic	SLC16A7	4.59387	Down	NULL	PBS_peak_2878
peak1498	chr12	77971018	77971177	0	NM_0010	NAV3	253971	NM_2033	E2F7	511737	intergenic	E2F7(dist=	9.15548	Down	NULL	PBS_peak_2906
peak1499	chr12	93805503	93805677	1	NM_0140	MRPL42	55676	NR_04009	LOC64331	34078	intronic	UBE2N	5.02617	Down	NULL	NULL
peak1500	chr12	93818601	93818784	1	NM_0140	MRPL42	42573	NR_04009	LOC64331	47180	intronic	UBE2N	10.75974	Down	NULL	PBS_peak_2916
peak1501	chr12	93824181	93824394	1	NM_0140	MRPL42	36978	NR_04009	LOC64331	52775	intronic	UBE2N	4.93946	Down	NULL	PBS_peak_2920
peak1502	chr12	97515877	97516052	0	NR_02403	RMST	342834	NM_0025	CDK17	721598	intergenic	NEDD1(d	4.77442	Down	NULL	PBS_peak_2931
peak1503	chr12	97814584	97814738	0	NR_02403	RMST	44138	NM_0025	CDK17	1020295	intergenic	NEDD1(d	4.69822	Down	NULL	PBS_peak_2934
peak1504	chr12	99834364	99834537	1	NM_1533	FAM71C	207077	NM_0012	ANKS1B	285582	intronic	ANKS1B	4.86161	Down	NULL	NULL
peak1505	chr12	101271402	101271610	2	NM_0012	ANO4	23918	NM_001319310		610649	intronic	ANO4	3.47412	Down	NULL	PBS_peak_2960
peak1506	chr12	103442502	103442692	0	NR_12633	LOC10192	103023	NM_0002	PAH	131216	intergenic	ASCL1(di	5.15463	Down	NULL	PBS_peak_2971
peak1507	chr12	104701520	104701706	7	NM_0011	CHST11	149079	NM_0061	NFYB	169573	intronic	TXNRD1	5.05873	Up	luks_peak	NULL
peak1508	chr12	106163753	106163904	0	NM_0012	TCP11L2	532740	NR_13198	KCCAT19	443033	intergenic	CASC18(d	12.60512	Down	NULL	PBS_peak_2984
peak1509	chr12	106177311	106177468	0	NM_0012	TCP11L2	519179	NR_13198	KCCAT19	456594	intergenic	CASC18(d	20.43649	Down	NULL	NULL
peak1510	chr12	106458133	106458311	1	NM_0012	TCP11L2	238347	NR_13198	KCCAT19	737427	UTR3	NUAK1(N	11.33063	Down	NULL	PBS_peak_2998
peak1511	chr12	107047250	107047421	3	NM_0324	RFX4	31141	NM_0068	CKAP4	405622	ncRNA_in	LOC10028	5.87614	Down	NULL	PBS_peak_2999
peak1512	chr12	107101303	107101461	4	NM_0181	RIC8B	67017	NR_03891	LOC10050	22904	ncRNA_in	LOC10028	9.45758	Down	NULL	NULL
peak1513	chr12	108938352	108938531	1	NM_0013	ISCU	16797	NR_12048	LINC0149	70975	intronic	SART3	8.68909	Down	NULL	PBS_peak_3012
peak1514	chr12	108939503	108939677	1	NM_0013	ISCU	15649	NR_12048	LINC0149	72124	intronic	SART3	6.81255	Down	NULL	NULL
peak1515	chr12	108944004	108944161	1	NM_0013	ISCU	11156	NR_12048	LINC0149	76616	intronic	SART3	4.05618	Down	NULL	NULL
peak1516	chr12	108953219	108953391	1	NM_0013	ISCU	1934	NR_12048	LINC0149	85839	intronic	SART3	4.36909	Down	NULL	PBS_peak_3018
peak1517	chr12	111167134	111167290	2	NM_0012	CCDC63	117552	NM_0012	HVCN1	39595	intronic	PPP1CC	26.83797	Down	NULL	PBS_peak_3038

peak1518	chr12	111170236	111170454	2	NM_0012	CCDC63	114419	NM_0012	HVCN1	42728	intronic	PPP1CC	5.56096	Down	NULL	PBS_peak_3040
peak1519	chr12	111172596	111172760	2	NM_0012	CCDC63	112086	NM_0012	HVCN1	45061	intronic	PPP1CC	4.79336	Down	NULL	NULL
peak1520	chr12	111175374	111175771	2	NM_0012	CCDC63	109191	NM_0012	HVCN1	47955	intronic	PPP1CC	7.18996	Down	NULL	PBS_peak_3043
peak1521	chr12	111175910	111176070	2	NM_0012	CCDC63	108774	NM_0012	HVCN1	48373	intronic	PPP1CC	6.03764	Down	NULL	PBS_peak_3044
peak1522	chr12	112029608	112029765	4	NM_0011	ACAD10	94170	NM_1446	FAM109A	222761	intronic	ATXN2	7.91917	Down	NULL	PBS_peak_3075
peak1523	chr12	112458021	112458188	2	NM_0011	TRAFD1	105244	NR_1221	TMEM116	7081	intronic	ERP29	7.21211	Down	NULL	PBS_peak_3076
peak1524	chr12	112537942	112538105	1	NM_0011	TRAFD1	25325	NR_0374	MIR3657	62504	intronic	NAA25	6.57671	Down	NULL	PBS_peak_3079
peak1525	chr12	112540651	112540833	1	NM_0011	TRAFD1	22607	NR_0374	MIR3657	65223	intronic	NAA25	7.25226	Down	NULL	NULL
peak1526	chr12	113355705	113356338	4	NM_0061	OAS3	20216	NR_0316	MIR1302-	223040	UTR3	OAS1(NM	9.70391	Up	luks_peak	NULL
peak1527	chr12	113382149	113382347	1	NM_0010	OAS2	34026	NR_0316	MIR1302-	249267	exonic	OAS3	4.56586	Up	NULL	NULL
peak1528	chr12	113383265	113383491	1	NM_0010	OAS2	32896	NR_0316	MIR1302-	250397	intronic	OAS3	10.49179	Up	NULL	NULL
peak1529	chr12	113383898	113384835	1	NM_0010	OAS2	31907	NR_0316	MIR1302-	251385	exonic	OAS3	10.1028	Up	luks_peak	NULL
peak1530	chr12	113385923	113386265	1	NM_0010	OAS2	30180	NR_0316	MIR1302-	253113	intronic	OAS3	24.67723	Up	luks_peak	NULL
peak1531	chr12	113386407	113387124	1	NM_0010	OAS2	29508	NR_0316	MIR1302-	253784	exonic	OAS3	5.98061	Up	luks_peak	NULL
peak1532	chr12	113387245	113387744	1	NM_0010	OAS2	28779	NR_0316	MIR1302-	254513	intronic	OAS3	16.34002	Up	luks_peak	NULL
peak1533	chr12	113387996	113388600	1	NM_0010	OAS2	27976	NR_0316	MIR1302-	255317	exonic	OAS3	7.20563	Up	luks_peak	NULL
peak1534	chr12	113392357	113392528	1	NM_0010	OAS2	23831	NR_0316	MIR1302-	259461	intronic	OAS3	19.45982	Up	NULL	NULL
peak1535	chr12	113393601	113393776	1	NM_0010	OAS2	22585	NR_0316	MIR1302-	260707	intronic	OAS3	16.28617	Up	luks_peak	NULL
peak1536	chr12	113396775	113397179	1	NM_0010	OAS2	19297	NR_0316	MIR1302-	263996	intronic	OAS3	11.19075	Up	luks_peak	NULL
peak1537	chr12	113400493	113400647	1	NM_0010	OAS2	15704	NR_0316	MIR1302-	267589	exonic	OAS3	4.16892	Up	luks_peak	NULL
peak1538	chr12	113400951	113401137	1	NM_0010	OAS2	15230	NR_0316	MIR1302-	268063	exonic	OAS3	10.91078	Up	NULL	NULL
peak1539	chr12	113402678	113402970	1	NM_0010	OAS2	13450	NR_0316	MIR1302-	269843	intronic	OAS3	8.61325	Up	luks_peak	NULL
peak1540	chr12	113403697	113404232	1	NM_0010	OAS2	12309	NR_0316	MIR1302-	270983	exonic	OAS3	6.00923	Up	luks_peak	NULL
peak1541	chr12	113404754	113405081	1	NM_0010	OAS2	11356	NR_0316	MIR1302-	271936	intronic	OAS3	9.38971	Up	luks_peak	NULL
peak1542	chr12	113405158	113405386	1	NM_0010	OAS2	11002	NR_0316	MIR1302-	272291	exonic	OAS3	4.67058	Up	luks_peak	NULL
peak1543	chr12	113405715	113405923	1	NM_0010	OAS2	10455	NR_0316	MIR1302-	272838	exonic	OAS3	11.98423	Up	luks_peak	NULL
peak1544	chr12	113407102	113407504	1	NM_0010	OAS2	8971	NR_0316	MIR1302-	274322	exonic	OAS3	8.33448	Up	luks_peak	NULL
peak1545	chr12	113407785	113408196	1	NM_0010	OAS2	8283	NR_0316	MIR1302-	275009	UTR3	OAS3(NM	12.83406	Up	luks_peak	NULL
peak1546	chr12	113408294	113408451	1	NM_0010	OAS2	7901	NR_0316	MIR1302-	275391	UTR3	OAS3(NM	6.06635	Up	NULL	NULL
peak1547	chr12	113423931	113424252	3	NM_0044	DTX1	71570	NR_0316	MIR1302-	291110	intronic	OAS2	5.34684	Up	luks_peak	NULL
peak1548	chr12	113424733	113424933	3	NM_0044	DTX1	70829	NR_0316	MIR1302-	291852	exonic	OAS2	4.75242	Up	NULL	NULL
peak1549	chr12	113427934	113428339	2	NM_0044	DTX1	67525	NR_0316	MIR1302-	295155	intronic	OAS2	4.98792	Up	luks_peak	NULL
peak1550	chr12	113428597	113428948	2	NM_0044	DTX1	66889	NR_0316	MIR1302-	295791	intronic	OAS2	7.75475	Up	luks_peak	NULL
peak1551	chr12	113434206	113434449	2	NM_0044	DTX1	61334	NR_0316	MIR1302-	301346	intronic	OAS2	20.25918	Up	luks_peak	NULL
peak1552	chr12	113435400	113435773	2	NM_0044	DTX1	60075	NR_0316	MIR1302-	302605	exonic	OAS2	11.54099	Up	luks_peak	NULL
peak1553	chr12	113436433	113436585	2	NM_0044	DTX1	59153	NR_0316	MIR1302-	303528	intronic	OAS2	6.02627	Up	NULL	NULL
peak1554	chr12	113436784	113437247	2	NM_0044	DTX1	58646	NR_0316	MIR1302-	304034	intronic	OAS2	15.1428	Up	luks_peak	NULL
peak1555	chr12	113438054	113438319	2	NM_0044	DTX1	57475	NR_0316	MIR1302-	305205	intronic	OAS2	29.06758	Up	luks_peak	NULL
peak1556	chr12	113438486	113438656	2	NM_0044	DTX1	57091	NR_0316	MIR1302-	305590	intronic	OAS2	27.51388	Up	NULL	NULL
peak1557	chr12	113438939	113439145	2	NM_0044	DTX1	56620	NR_0316	MIR1302-	306061	intronic	OAS2	23.73953	Up	luks_peak	NULL
peak1558	chr12	113440973	113441129	2	NM_0044	DTX1	54611	NR_0316	MIR1302-	308070	intronic	OAS2	7.21572	Up	NULL	NULL
peak1559	chr12	114620402	114620560	0	NR_0384	TBX5-AS	225519	NM_0161	RBM19	216305	intergenic	RBM19(di	6.71126	Down	NULL	PBS_peak_3094
peak1560	chr12	114684850	114685010	0	NR_0384	TBX5-AS	161070	NM_0161	RBM19	280754	intergenic	LINC0245	10.11107	Down	NULL	NULL
peak1561	chr12	117834357	117834528	0	NM_0011	RFC5	620063	NM_0012	NOS1	34835	intergenic	NOS1(dist	23.66746	Down	NULL	PBS_peak_3120
peak1562	chr12	118106135	118106355	1	NM_0011	RFC5	348261	NM_0012	NOS1	306638	intronic	KSR2	12.06833	Down	NULL	PBS_peak_3136
peak1563	chr12	120903356	120903516	1	NM_0010	DYNLL1	4224	NM_0163	TRIAP1	19221	exonic	SRSF9	5.10656	Down	NULL	PBS_peak_3150
peak1564	chr12	121464715	121464875	3	NR_0339	P2RX7	105827	NR_1044	C12orf43	10490	intronic	OASL	26.5239	Up	NULL	NULL
peak1565	chr12	121468394	121468563	3	NR_0339	P2RX7	102143	NR_1044	C12orf43	14173	intronic	OASL	16.32573	Up	NULL	NULL
peak1566	chr12	121470204	121470392	3	NR_0339	P2RX7	100324	NR_1044	C12orf43	15993	intronic	OASL	15.25062	Up	NULL	NULL
peak1567	chr12	121473450	121473600	3	NR_0339	P2RX7	97097	NR_1044	C12orf43	19220	intronic	OASL	9.19259	Up	NULL	NULL
peak1568	chr12	124964597	124964796	3	NR_0498	MIR5188	435396	NR_1069	MIR6880	142908	intronic	NCOR2	5.34731	Down	NULL	PBS_peak_3158
peak1569	chr12	124973573	124973728	3	NR_0498	MIR5188	426442	NR_1069	MIR6880	151862	intronic	NCOR2	5.07703	Down	NULL	NULL
peak1570	chr12	124986606	124986761	3	NR_0498	MIR5188	413409	NR_1069	MIR6880	164895	intronic	NCOR2	5.18979	Down	NULL	PBS_peak_3170
peak1571	chr12	125000010	125000192	3	NR_0498	MIR5188	399992	NR_1069	MIR6880	178313	intronic	NCOR2	7.28395	Down	NULL	PBS_peak_3180
peak1572	chr12	125022891	125023063	3	NR_0498	MIR5188	377116	NR_1069	MIR6880	201189	intronic	NCOR2	3.65087	Down	NULL	PBS_peak_3188
peak1573	chr12	125023165	125023425	3	NR_0498	MIR5188	376798	NR_1069	MIR6880	201507	intronic	NCOR2	5.37246	Down	NULL	PBS_peak_3188
peak1574	chr12	125023507	125023830	3	NR_0498	MIR5188	376424	NR_1069	MIR6880	201880	intronic	NCOR2	4.57052	Down	NULL	PBS_peak_3188
peak1575	chr12	125031023	125031322	3	NR_0498	MIR5188	368920	NR_1069	MIR6880	209384	intronic	NCOR2	5.21475	Down	NULL	PBS_peak_3193
peak1576	chr12	125034515	125034729	3	NR_0498	MIR5188	365471	NR_1069	MIR6880	212834	intronic	NCOR2	7.01322	Down	NULL	PBS_peak_3194
peak1577	chr12	126789625	126789781	0	NR_1307	LOC1001	137324	NR_0341	LINC0093	321783	intergenic	LINC0235	17.63476	Down	NULL	NULL
peak1578	chr12	130035296	130035467	1	NM_0071	FZD10	611622	NM_1456	SLC15A4	726840	intronic	TMEM132	7.56679	Down	NULL	PBS_peak_3223
peak1579	chr12	133080510	133080683	1	NR_1068	MIR6763	77986	NM_0011	GALNT9	174691	intronic	FBRSL1	5.47251	Down	NULL	PBS_peak_3236
peak1580	chr13	26504685	26504840	2	NM_0012	CDK8	323478	NM_0046	MTMR6	643058	intronic	ATP8A2	4.07739	Up	NULL	NULL
peak1581	chr13	29699978	29700130	1	NM_0152	MTUS2	302723	NM_1817	SLC46A3	406904	intronic	MTUS2	9.851	Down	NULL	NULL
peak1582	chr13	30134749	30134911	1	NR_135320		87000	NR_0463	MTUS2-A	72943	intronic	SLC7A1	5.38977	Down	NULL	NULL
peak1583	chr13	30137686	30137979	1	NR_135320		83997	NR_0463	MTUS2-A	75945	intronic	SLC7A1	4.57736	Down	NULL	PBS_peak_3252
peak1584	chr13	30155345	30155651	1	NR_135320		66332	NR_0463	MTUS2-A	93611	intronic	SLC7A1	3.92942	Down	NULL	PBS_peak_3259
peak1585	chr13	30160643	30160807	1	NR_135320		61105	NR_0463	MTUS2-A	98838	intronic	SLC7A1	8.65174	Down	NULL	PBS_peak_3262
peak1586	chr13	30161597	30161986	1	NR_135320		60038	NR_0463	MTUS2-A	99904	intronic	SLC7A1	7.50726	Down	NULL	PBS_peak_3263
peak1587	chr13	30162440	30162633	1	NR_135320		59293	NR_0463	MTUS2-A	100649	intronic	SLC7A1	27.52415	Down	NULL	PBS_peak_3264
peak1588	chr13	30165062	30165251	1	NR_135320		56673	NR_0463	MTUS2-A	103269	intronic	SLC7A1	9.18721	Down	NULL	PBS_peak_3267
peak1589	chr13	31205346	31205530	1	NM_0012	ALOX5AI	82177	NM_001313893		13704	exonic	USPL1	25.28664	Down	NULL	PBS_peak_3270
peak1590	chr13	36562288	36562440	1	NR_0451	SPG20-AS	358204	NR_0316	MIR548F	46982	intronic	DCLK1	18.1571	Down	NULL	PBS_peak_3276
peak1591	chr13	38712644	38712809	1	NM_0012	UFM1	211181	NM_0161	TRPC4	268787	ncRNA_in	LINC0057	23.54432	Down	NULL	NULL
peak1592	chr13	40075196	40075352	1	NM_0207	COG6	154490	NM_0251	PROSER1	463061	intronic	LHFP	16.12135	Down	NULL	NULL
peak1593	chr13	43496508	43496678	2	NM_0132	DNAJC15	100769	NR_1263	LINC0042	49713	intronic	EPSTI1	8.17463	Up	NULL	NULL
peak1594	chr13	43497549	43497704	2	NM_0132	DNAJC15	99735	NR_1263	LINC0042	50746	intronic	EPSTI1	20.59507	Up	NULL	NULL
peak1595	chr13	43506462	43506616	2	NM_0132	DNAJC15	90823	NR_1263	LINC0042	59659	intronic	EPSTI1	13.86352	Up	NULL	NULL
peak1596	chr13	43518500	43518759	2	NM_0132	DNAJC15	78732	NR_1263	LINC0042	71749	intronic	EPSTI1	13.43176	Up	NULL	NULL
peak1597	chr13	43539529	43539716	2	NM_0132	DNAJC15	57739	NR_1263	LINC0042	92742	intronic	EPSTI1	13.10144	Up	NULL	NULL
peak1598	chr13	43541563	43541840	2	NM_0132	DNAJC15	55660	NR_1263	LINC0042	94821	intronic	EPSTI1	8.4592	Up	luks_peak	NULL
peak1599	chr13	43545806	43545995	2	NM_0132	DNAJC15	51461	NR_1263	LINC0042	99020	intronic	EPSTI1	13.60696	Up	NULL	NULL
peak1600	chr13	43546901	43547137	2	NM_0132	DNAJC15	50343	NR_1263	LINC0042	100139	intronic	EPSTI1	4.69092	Up	NULL	NULL
peak1601	chr13	43550291	43550443	2	NM_0132	DNAJC15	46995	NR_1263	LINC0042	103487	intronic	EPSTI1	5.41062	Up	NULL	NULL
peak1602	chr13	43552366	43552525	2	NM_0132	DNAJC15	44916	NR_1263	LINC0042	105565	intronic	EPSTI1	6.16535	Up	NULL	NULL
peak1603	chr13	43554510	43554724	2	NM_0132	DNAJC15	42745	NR_1263	LINC0042	107737	intronic	EPSTI1	7.93584	Up	NULL	NULL
peak1604	chr13	43556920	43557076	2	NM_0132	DNAJC15	40364	NR_1263	LINC0042	110118	intronic	EPSTI1	8.24175	Up	NULL	NULL
peak1605	chr13	43557805	43557965	2	NM_0132	DNAJC15	39477	NR_1263	LINC0042	111005	intronic	EPSTI1	8.77861	Up	NULL	NULL
peak1606	chr13	52221303	52221472	1	NM_0312	CCDC70	214729	NM_0010	INTS6	194112	intronic	WDFY2	18.8789	Down	NULL	PBS_peak_3299
peak1607	chr13	52222391	52222549	1	NM_0312	CCDC70	213647	NM_0010	INTS6	195195	intronic	WDFY2	6.66788	Down	NULL	NULL
peak1608	chr13	52248585	52248740	1	NM_0312	CCDC70	187454	NM_0010	INTS6	221387	intronic	WDFY2	4.87775	Down	NULL	PBS_peak_3301
peak1609	chr13	69037848	69038020	0	NR_1257	LINC0038	758544	NM_001318374		1233466	intergenic	LINC0036	6.35238	Down	NULL	NULL
peak1610	chr13	79226935	79227125	1	NR_0389	RBM26-A	753414	NM_0062	POU4F1	49335	intronic	RNF219	6.06192	Down	NULL	PBS_peak_3306
peak1611	chr13	95401674	95401863	0	NR_1107	LOC1019	11426	NM_0070	SOX21	36971	intergenic	SOX21-A	9.07709	Down	NULL	PBS_peak_3308
peak1612	chr13	99181304	99181471	2	NR_0466	DOCK9-A	302950	NM_0035	STK24	7008	intronic	STK24	4.46463	Up	NULL	NULL
peak1613	chr13	103252568	103252760	1	NM_0011	BIVM	198735	NM_1759	FGF14	198540	intronic	TPP2	8.21575	Down	NULL	PBS_peak_3340
peak1614	chr13	107198028	107198187	1	NR_0519	LINC0055	72050	NM_0040	EFNB2	10719	intronic	ARGLU1	5.09159	Down	NULL	NULL
peak1615	chr13	108269106	108269260	1	NM_0328	ABHD13	601580	NR_0316	MIR1267	85587	intronic	FAM155A	5.7458	Down	NULL	PBS_peak_3355
peak1616	chr13	111203668	111203880	1	NM_0012	CARKD	64033	NR_0465	COL4A2-	43248	intronic	RAB20	4.27248	Up	luks_peak	NULL
peak1617	chr13	111204245	111204480	1	NM_0012	CARKD	63444	NR_0465	COL4A2-	43836	intronic	RAB20	4.63234	Up	luks_peak	NULL
peak1618	chr13	111205349	111205518	1	NM_0012	CARKD	62373	NR_0465	COL4A2-	44907	intronic	RAB20	8.2317	Up	luks_peak	NULL
peak1619	chr13	113226710	113226891	4	NM_2074	ATP11AU	74557	NR_1263	LINC0104	241261	intronic	TUBGCP3	8.27027	Down	NULL	PBS_peak_3361
peak1620	chr13	113424057	113424394	2	NM_0011	MCF2L	199309	NR_1098	ATP11A-	15180	intronic	ATP11A	4.98593	Down	NULL	PBS_peak_3378

peak1621	chr13	113436475	113436630	2	NM_0011	MCF2L	186982	NR_1098	ATP11A-A	27507	intronic	ATP11A	4.30657	Down	NULL	PBS_peak_3379
peak1622	chr13	114243899	114244267	2	NM_0029	GRK1	77514	NM_0010	DCUN1D	99060	intronic	TFDP1	5.28596	Down	NULL	PBS_peak_3391
peak1623	chr13	114246072	114246284	2	NM_0029	GRK1	75419	NM_0010	DCUN1D	101155	intronic	TFDP1	21.25401	Down	NULL	PBS_peak_3392
peak1624	chr13	114256230	114256422	2	NM_0029	GRK1	65271	NM_0010	DCUN1D	111303	intronic	TFDP1	16.40334	Down	NULL	PBS_peak_3395
peak1625	chr13	114256842	114257058	2	NM_0029	GRK1	64647	NM_0010	DCUN1D	111927	intronic	TFDP1	6.47137	Down	NULL	PBS_peak_3396
peak1626	chr13	114263830	114264007	2	NM_0029	GRK1	57678	NM_0010	DCUN1D	118895	intronic	TFDP1	6.03321	Down	NULL	PBS_peak_3399
peak1627	chr13	114265819	114265985	2	NM_0029	GRK1	55695	NM_0010	DCUN1D	120879	intronic	TFDP1	15.49714	Down	NULL	PBS_peak_3401
peak1628	chr13	114282661	114282811	2	NM_0029	GRK1	38861	NM_0010	DCUN1D	137713	intronic	TFDP1	4.46561	Down	NULL	PBS_peak_3407
peak1629	chr14	26489580	26489743	0	NR_1105	LOC1027	788892	NM_0013	STXBP6	970158	intergenic	STXBP6(	5.38931	Down	NULL	NULL
peak1630	chr14	32279629	32279811	4	NM_0010	ARHGAP	266775	NR_0366	GPR33	322569	intronic	NUBPL	11.32915	Down	NULL	PBS_peak_3450
peak1631	chr14	33517863	33518024	4	NR_132771		660199	NR_1257	RNU6-2	845468	intronic	NPAS3	4.95949	Down	NULL	NULL
peak1632	chr14	33778766	33778917	4	NR_132771		399301	NR_1257	RNU6-2	1106366	intronic	NPAS3	6.96723	Down	NULL	NULL
peak1633	chr14	33791287	33791450	4	NR_132771		386774	NR_1257	RNU6-2	1118893	intronic	NPAS3	5.5927	Down	NULL	PBS_peak_3453
peak1634	chr14	34101726	34101889	4	NR_132771		76335	NR_1257	RNU6-2	1429332	intronic	NPAS3	5.17001	Down	NULL	NULL
peak1635	chr14	35263499	35263664	2	NR_0029	IGBP1P1	145546	NR_0281	CFL2	79552	intronic	BAZ1A	4.67158	Up	NULL	NULL
peak1636	chr14	36561374	36561542	1	NR_1037	NKX2-1-A	427025	NM_0012	RALGAPA	283026	ncRNA_in	LINC006	7.44615	Down	NULL	NULL
peak1637	chr14	38483061	38483221	0	NM_0010	SSTR1	194063	NR_135286		111812	intergenic	LINC0051	20.76606	Down	NULL	PBS_peak_3461
peak1638	chr14	38705756	38705970	0	NR_135256		645936	NR_135286		334534	intergenic	SSTR1(dis	6.17257	Down	NULL	PBS_peak_3462
peak1639	chr14	46129762	46129932	0	NR_1027	LINC0087	403515	NM_0183	MIS18BP	407242	intergenic	MIS18BP	5.33519	Down	NULL	NULL
peak1640	chr14	51912506	51912711	0	NM_0010	FRMD6	43230	NM_0529	TRIM9	350186	intergenic	LINC0231	15.67781	Down	NULL	PBS_peak_3472
peak1641	chr14	55343159	55343331	4	NM_0808	SOCS4	150599	NM_0041	GMFB	387501	intronic	GCH1	6.34145	Up	NULL	NULL
peak1642	chr14	62931060	62931239	0	NM_0206	RHOJ	739952	NR_1040	LINC0064	324458	intergenic	LINC0064	5.46953	Down	NULL	NULL
peak1643	chr14	66780253	66780426	0	NR_0243	LINC0023	172749	NR_0243	FUT8-AS	901004	intergenic	FUT8(dist	4.78295	Down	NULL	NULL
peak1644	chr14	69376684	69376867	3	NR_0734	ACTN1-A	69623	NM_0012	ZFP36L1	113815	exonic	ACTN1	4.80001	Down	NULL	PBS_peak_3496
peak1645	chr14	69399391	69399651	3	NR_0734	ACTN1-A	46878	NM_0012	ZFP36L1	136561	intronic	ACTN1	3.99294	Down	NULL	PBS_peak_3502
peak1646	chr14	69400289	69400444	3	NR_0734	ACTN1-A	46032	NM_0012	ZFP36L1	137406	intronic	ACTN1	5.07048	Down	NULL	PBS_peak_3503
peak1647	chr14	69402860	69403126	3	NR_0734	ACTN1-A	43406	NM_0012	ZFP36L1	140033	intronic	ACTN1	9.94838	Down	NULL	PBS_peak_3505
peak1648	chr14	69404205	69404580	3	NR_0734	ACTN1-A	42006	NM_0012	ZFP36L1	141432	intronic	ACTN1	4.72101	Down	NULL	PBS_peak_3506
peak1649	chr14	69404678	69404835	3	NR_0734	ACTN1-A	41642	NM_0012	ZFP36L1	141796	intronic	ACTN1	4.21988	Down	NULL	PBS_peak_3506
peak1650	chr14	69408334	69408598	3	NR_0734	ACTN1-A	37933	NM_0012	ZFP36L1	145506	intronic	ACTN1	6.35479	Down	NULL	PBS_peak_3510
peak1651	chr14	69409112	69409523	3	NR_0734	ACTN1-A	37081	NM_0012	ZFP36L1	146357	intronic	ACTN1	4.83383	Down	NULL	PBS_peak_3510
peak1652	chr14	69410557	69410876	3	NR_0734	ACTN1-A	35682	NM_0012	ZFP36L1	147756	intronic	ACTN1	5.25926	Down	NULL	PBS_peak_3511
peak1653	chr14	69413809	69414014	3	NR_0734	ACTN1-A	32487	NM_0012	ZFP36L1	150951	intronic	ACTN1	6.70139	Down	NULL	PBS_peak_3513
peak1654	chr14	69417693	69417862	3	NR_0734	ACTN1-A	28621	NM_0012	ZFP36L1	154817	intronic	ACTN1	5.64214	Down	NULL	PBS_peak_3516
peak1655	chr14	69420182	69420371	3	NR_0734	ACTN1-A	26122	NM_0012	ZFP36L1	157316	intronic	ACTN1	3.55099	Down	NULL	PBS_peak_3517
peak1656	chr14	69421701	69422023	3	NR_0734	ACTN1-A	24537	NM_0012	ZFP36L1	158902	intronic	ACTN1	4.30818	Down	NULL	PBS_peak_3518
peak1657	chr14	69422419	69422574	3	NR_0734	ACTN1-A	23902	NM_0012	ZFP36L1	159536	intronic	ACTN1	3.44938	Down	NULL	PBS_peak_3519
peak1658	chr14	69423704	69424185	3	NR_0734	ACTN1-A	22454	NM_0012	ZFP36L1	160984	intronic	ACTN1	7.59485	Down	NULL	PBS_peak_3520
peak1659	chr14	69424326	69424511	3	NR_0734	ACTN1-A	21980	NM_0012	ZFP36L1	161458	intronic	ACTN1	15.63735	Down	NULL	PBS_peak_3520
peak1660	chr14	69424643	69424840	3	NR_0734	ACTN1-A	21657	NM_0012	ZFP36L1	161781	intronic	ACTN1	21.8655	Down	NULL	PBS_peak_3520
peak1661	chr14	69425483	69425636	3	NR_0734	ACTN1-A	20839	NM_0012	ZFP36L1	162599	intronic	ACTN1	7.16201	Down	NULL	NULL
peak1662	chr14	69427622	69427787	3	NR_0734	ACTN1-A	18694	NM_0012	ZFP36L1	164744	intronic	ACTN1	4.34534	Down	NULL	PBS_peak_3522
peak1663	chr14	70144609	70144762	1	NM_001320214		89118	NM_0012	CCDC177	103085	intronic	SUSD6	5.24095	Up	NULL	NULL
peak1664	chr14	79105600	79105778	2	NR_135159		560636	NM_0122	SNW1	878192	intronic	NRXN3	6.40075	Down	NULL	NULL
peak1665	chr14	79542006	79542183	2	NR_135159		124230	NM_0122	SNW1	1314597	intronic	NRXN3	6.69991	Down	NULL	PBS_peak_3552
peak1666	chr14	91959425	91959593	2	NM_0174	CPSF2	628789	NM_0010	CCDC88	75321	intronic	PPP4R3A	5.47109	Down	NULL	PBS_peak_3572
peak1667	chr14	98741508	98741667	0	NM_1825	C14orf17	436362	NR_0154	LINC015	297126	intergenic	LINC015	4.02089	Down	NULL	PBS_peak_3599
peak1668	chr14	99932604	99932773	2	NM_0010	CCNK	15050	NM_1385	BCL11B	194638	intronic	SETD3	10.29221	Down	NULL	PBS_peak_3600
peak1669	chr14	99933913	99934098	2	NM_0010	CCNK	13733	NM_1385	BCL11B	195955	intronic	SETD3	4.05893	Down	NULL	PBS_peak_3601
peak1670	chr14	99936302	99936491	2	NM_0010	CCNK	11342	NM_1385	BCL11B	198346	intronic	SETD3	7.80032	Down	NULL	PBS_peak_3603
peak1671	chr14	99954316	99954491	1	NM_0324	HHIPL1	157076	NM_1991	SETD3	7177	intronic	CCNK	17.61887	Down	NULL	PBS_peak_3610
peak1672	chr14	100545770	100546010	1	NR_0298	MIR342	30102	NM_0011	CCDC85	475163	intronic	EVL	4.45323	Up	luks_peak	NULL
peak1673	chr14	100551017	100551211	1	NR_0298	MIR342	24878	NM_0011	CCDC85	480387	exonic	EVL	6.86162	Up	NULL	NULL
peak1674	chr14	100551400	100551635	1	NR_0298	MIR342	24474	NM_0011	CCDC85	480790	intronic	EVL	15.84432	Up	NULL	NULL
peak1675	chr14	100559240	100559394	1	NR_0298	MIR342	16675	NM_0011	CCDC85	488590	intronic	EVL	8.00807	Up	NULL	NULL
peak1676	chr14	100559830	100560089	1	NR_0298	MIR342	16032	NM_0011	CCDC85	489232	intronic	EVL	28.08852	Up	luks_peak	NULL
peak1677	chr14	100560399	100560583	1	NR_0298	MIR342	15501	NM_0011	CCDC85	489764	intronic	EVL	24.26804	Up	NULL	NULL
peak1678	chr14	100560943	100561181	1	NR_0298	MIR342	14930	NM_0011	CCDC85	490335	intronic	EVL	5.74873	Up	luks_peak	NULL
peak1679	chr14	100562481	100562631	1	NR_0298	MIR342	13436	NM_0011	CCDC85	491829	intronic	EVL	7.54897	Up	NULL	NULL
peak1680	chr14	100563434	100563604	1	NR_0298	MIR342	12473	NM_0011	CCDC85	492792	intronic	EVL	5.76542	Up	NULL	NULL
peak1681	chr14	100564013	100564177	1	NR_0298	MIR342	11897	NM_0011	CCDC85	493368	intronic	EVL	4.35178	Up	NULL	NULL
peak1682	chr14	100576002	100576268	2	NM_0034	YY1	128967	NR_0396	MIR151B	284	ncRNA_e	MIR342	15.673	Up	luks_peak	NULL
peak1683	chr14	100580033	100580194	1	NM_0034	YY1	124988	NR_0396	MIR151B	4262	intronic	EVL	9.00497	Up	NULL	NULL
peak1684	chr14	100812500	100812679	4	NM_0011	WDR25	30165	NM_1523	SLC25A2	39705	intronic	WARS	16.83571	Up	luks_peak	NULL
peak1685	chr14	100815978	100816128	4	NM_0011	WDR25	26702	NM_1523	SLC25A2	43169	intronic	WARS	4.51133	Up	NULL	NULL
peak1686	chr14	100816997	100817218	4	NM_0011	WDR25	25647	NM_1523	SLC25A2	44223	intronic	WARS	12.11512	Up	luks_peak	NULL
peak1687	chr14	100819724	100819947	4	NM_0011	WDR25	22919	NM_1523	SLC25A2	46951	intronic	WARS	27.1157	Up	NULL	NULL
peak1688	chr14	100820444	100820609	4	NM_0011	WDR25	22228	NM_1523	SLC25A2	47642	intronic	WARS	4.94771	Up	NULL	NULL
peak1689	chr14	100828009	100828173	4	NM_0011	WDR25	14664	NM_1523	SLC25A2	55207	exonic	WARS	4.09074	Up	NULL	NULL
peak1690	chr14	100832896	100833122	4	NM_0011	WDR25	9746	NM_1523	SLC25A2	60125	intronic	WARS	8.07471	Up	luks_peak	NULL
peak1691	chr14	100834285	100834447	4	NM_0011	WDR25	8389	NM_1523	SLC25A2	61482	intronic	WARS	6.11372	Up	NULL	NULL
peak1692	chr14	100837723	100837974	4	NM_0011	WDR25	4906	NM_1523	SLC25A2	64964	intronic	WARS	9.06618	Up	NULL	NULL
peak1693	chr14	103062940	103063109	1	NM_0011	TRAF3	180791	NM_1523	ANKRD9	86896	intronic	RCOR1	5.90228	Down	NULL	NULL
peak1694	chr14	103066088	103066284	1	NM_0011	TRAF3	177630	NM_1523	ANKRD9	90058	intronic	RCOR1	4.57728	Down	NULL	PBS_peak_3646
peak1695	chr14	103072444	103072607	1	NM_0011	TRAF3	171290	NM_1523	ANKRD9	96397	intronic	RCOR1	7.45981	Down	NULL	PBS_peak_3650
peak1696	chr14	103077374	103077551	1	NM_0011	TRAF3	166353	NM_1523	ANKRD9	101334	intronic	RCOR1	6.70389	Down	NULL	PBS_peak_3657
peak1697	chr14	103080767	103080964	1	NM_0011	TRAF3	162950	NM_1523	ANKRD9	104737	intronic	RCOR1	4.3951	Down	NULL	PBS_peak_3660
peak1698	chr14	103088608	103088815	1	NM_0011	TRAF3	155104	NM_1523	ANKRD9	112583	intronic	RCOR1	7.5873	Down	NULL	PBS_peak_3668
peak1699	chr14	103091305	103091569	1	NM_0011	TRAF3	152379	NM_1523	ANKRD9	115309	intronic	RCOR1	5.2876	Down	NULL	PBS_peak_3670
peak1700	chr14	103094292	103094462	1	NM_0011	TRAF3	149439	NM_1523	ANKRD9	118249	intronic	RCOR1	5.63899	Down	NULL	PBS_peak_3673
peak1701	chr14	103100300	103100471	1	NM_0011	TRAF3	143430	NM_1523	ANKRD9	124257	intronic	RCOR1	20.05247	Down	NULL	PBS_peak_3677
peak1702	chr14	103102554	103102806	1	NM_0011	TRAF3	141136	NM_1523	ANKRD9	126552	intronic	RCOR1	12.79115	Down	NULL	PBS_peak_3679
peak1703	chr14	103102968	103103184	1	NM_0011	TRAF3	140740	NM_1523	ANKRD9	126948	intronic	RCOR1	19.54744	Down	NULL	PBS_peak_3679
peak1704	chr14	103124322	103124504	1	NM_0011	TRAF3	119403	NM_1523	ANKRD9	148285	intronic	RCOR1	4.12396	Down	NULL	PBS_peak_3686
peak1705	chr14	103125980	103126130	1	NM_0011	TRAF3	117761	NM_1523	ANKRD9	149927	intronic	RCOR1	5.46436	Down	NULL	PBS_peak_3688
peak1706	chr14	103128919	103129171	1	NM_0011	TRAF3	114771	NM_1523	ANKRD9	152917	intronic	RCOR1	8.08506	Down	NULL	PBS_peak_3691
peak1707	chr14	103129798	103130016	1	NM_0011	TRAF3	113909	NM_1523	ANKRD9	153779	intronic	RCOR1	15.56495	Down	NULL	PBS_peak_3692
peak1708	chr14	103133087	103133254	1	NM_0011	TRAF3	110645	NM_1523	ANKRD9	157042	intronic	RCOR1	3.74211	Down	NULL	PBS_peak_3696
peak1709	chr14	103135756	103135965	1	NM_0011	TRAF3	107955	NM_1523	ANKRD9	159732	intronic	RCOR1	4.97974	Down	NULL	PBS_peak_3697
peak1710	chr14	103156808	103156964	1	NM_0011	TRAF3	86930	NM_1523	ANKRD9	180758	intronic	RCOR1	3.79346	Down	NULL	PBS_peak_3706
peak1711	chr14	103274511	103274792	4	NM_0309	AMN	114341	NM_1523	ANKRD9	298523	intronic	TRAF3	4.49366	Up	luks_peak	NULL
peak1712	chr14	103295151	103295305	4	NM_0309	AMN	93765	NM_1523	ANKRD9	319100	intronic	TRAF3	5.0204	Up	luks_peak	NULL
peak1713	chr14	103307630	103307799	4	NM_0309	AMN	81278	NM_1523	ANKRD9	331586	intronic	TRAF3	4.6741	Up	luks_peak	NULL
peak1714	chr14	103366826	103367114	4	NM_0309	AMN	22023	NM_1523	ANKRD9	390842	intronic	TRAF3	6.48109	Up	luks_peak	NULL
peak1715	chr14	103858526	103858691	5	NM_1523	TRMT61A	136900	NR_0339	LINC006	203243	intronic	MARK3	4.37484	Down	NULL	PBS_peak_3726
peak1716	chr14	103863714	103863873	5	NM_1523	TRMT61A	131715	NR_0339	LINC006	208428	intronic	MARK3	3.31024	Down	NULL	NULL
peak1717	chr14	105481201	105481364	2	NR_1105	LOC1027	79201	NM_1384	AHNAK2	36588	intronic	CDCA4	22.60886	Down	NULL	PBS_peak_3747
peak1718	chr14	105481679	105481880	2	NR_1105	LOC1027	78704	NM_1384	AHNAK2	37085	intronic	CDCA4	4.64006	Down	NULL	PBS_peak_3748
peak1719	chr14	105482311	105482544	2	NR_1105	LOC1027	78056	NM_1384	AHNAK2	37733	intronic	CDCA4	5.36747	Down	NULL	PBS_peak_3749
peak1720	chr15	24001176	24001355	0	NR_1263	PWRN4	219028	NM_0024	NDN	68815	intergenic	NDN(dist=	22.17373	Down	NULL	PBS_peak_3759
peak1721	chr15	24358202	24358378	0	NR_1307	PWRN3	327984	NM_0024	NDN	425840	intergenic	PWRN4(d	5.83989	Down	NULL	PBS_peak_3760
peak1722	chr15	26038191	26038372	1	NR_0400	LOC1001	109225	NM_1308	UBE3A	354106	intronic	ATP10A	19.37986	Up	NULL	NULL
peak1723	chr15	26039265	26039419	1	NR_0400	LOC1001	108165	NM_1308	UBE3A	355167	intronic	ATP10A	25.46897	Up	NULL	NULL

peak1724	chr15	26040422	26040573	1	NR_04008	LOC10012	107009	NM_1308	UBE3A	356322	intronic	ATP10A	3.4028	Up	NULL	NULL
peak1725	chr15	26042534	26042772	1	NR_04008	LOC10012	104854	NM_1308	UBE3A	358478	intronic	ATP10A	18.94323	Up	NULL	NULL
peak1726	chr15	26042855	26043032	1	NR_04008	LOC10012	104563	NM_1308	UBE3A	358768	intronic	ATP10A	4.37368	Up	NULL	NULL
peak1727	chr15	26043547	26043841	1	NR_04008	LOC10012	103813	NM_1308	UBE3A	359519	intronic	ATP10A	15.10772	Up	NULL	NULL
peak1728	chr15	26047394	26047612	1	NR_04008	LOC10012	100004	NM_1308	UBE3A	363328	intronic	ATP10A	15.5884	Up	NULL	NULL
peak1729	chr15	26047748	26048065	1	NR_04008	LOC10012	99600	NM_1308	UBE3A	363731	intronic	ATP10A	26.35933	Up	luks_peak	NULL
peak1730	chr15	26050183	26050350	1	NR_04008	LOC10012	97240	NM_1308	UBE3A	366091	intronic	ATP10A	4.86327	Up	NULL	NULL
peak1731	chr15	26054846	26055104	1	NR_04008	LOC10012	92532	NM_1308	UBE3A	370800	intronic	ATP10A	13.03777	Up	luks_peak	NULL
peak1732	chr15	26055249	26055405	1	NR_04008	LOC10012	92180	NM_1308	UBE3A	371152	intronic	ATP10A	23.44096	Up	NULL	NULL
peak1733	chr15	26058113	26058339	1	NR_04008	LOC10012	89281	NM_1308	UBE3A	374051	intronic	ATP10A	11.35687	Up	luks_peak	NULL
peak1734	chr15	26069382	26069540	1	NR_04008	LOC10012	78046	NM_1308	UBE3A	385286	intronic	ATP10A	23.78351	Up	NULL	NULL
peak1735	chr15	26080526	26080904	1	NR_04008	LOC10012	66792	NM_1308	UBE3A	396540	intronic	ATP10A	6.27386	Up	luks_peak	NULL
peak1736	chr15	26081889	26082053	1	NR_04008	LOC10012	65536	NM_1308	UBE3A	397796	intronic	ATP10A	5.28229	Up	luks_peak	NULL
peak1737	chr15	26082503	26083162	1	NR_04008	LOC10012	64674	NM_1308	UBE3A	398657	intronic	ATP10A	9.24628	Up	luks_peak	NULL
peak1738	chr15	26088541	26088835	1	NR_04008	LOC10012	58819	NM_1308	UBE3A	404513	intronic	ATP10A	22.92935	Up	luks_peak	NULL
peak1739	chr15	26089758	26089926	1	NR_04008	LOC10012	57665	NM_1308	UBE3A	405667	intronic	ATP10A	12.90749	Up	NULL	NULL
peak1740	chr15	26090807	26091188	1	NR_04008	LOC10012	56509	NM_1308	UBE3A	406822	intronic	ATP10A	4.4614	Up	luks_peak	NULL
peak1741	chr15	26093537	26093698	1	NR_04008	LOC10012	53889	NM_1308	UBE3A	409442	intronic	ATP10A	3.86979	Up	NULL	NULL
peak1742	chr15	26101657	26101817	1	NR_04008	LOC10012	45770	NR_03986	MIR4715	7765	intronic	ATP10A	24.0323	Up	NULL	NULL
peak1743	chr15	26597516	26597686	0	NM_0008	GABRA5	514265	NM_0244	ATP10A	489252	intergenic	LINC0092	5.05076	Down	NULL	PBS_peak_3761
peak1744	chr15	27771376	27771533	1	NR_03335	GOLGA8I	852329	NR_12034	GABRG3-	364988	intronic	GABRG3	6.34875	Down	NULL	NULL
peak1745	chr15	35480001	35480160	0	NR_00314	ANP32AP	49446	NM_0141	ZNF770	199583	intergenic	ZNF770(d	4.80797	Up	NULL	NULL
peak1746	chr15	42224670	42224978	1	NR_12033	PLA2G4E	40137	NM_0166	SPTBN5	38549	intronic	EHD4	13.5326	Up	luks_peak	NULL
peak1747	chr15	42226706	42226987	1	NR_12033	PLA2G4E	38114	NM_0166	SPTBN5	40571	intronic	EHD4	11.29122	Up	luks_peak	NULL
peak1748	chr15	42249245	42249507	1	NR_12033	PLA2G4E	15585	NM_0166	SPTBN5	63101	intronic	EHD4	11.71803	Up	luks_peak	NULL
peak1749	chr15	61865971	61866152	0	NM_2073	C2CD4A	493114	NM_1342	RORA	344559	intergenic	RORA(dis	11.01666	Down	NULL	PBS_peak_3820
peak1750	chr15	63498127	63498394	1	NM_0011	APH1B	71488	NM_0159	RPS27L	48519	intronic	RAB8B	7.11117	Up	luks_peak	NULL
peak1751	chr15	63498462	63498630	1	NM_0011	APH1B	71203	NM_0159	RPS27L	48805	intronic	RAB8B	14.59279	Up	luks_peak	NULL
peak1752	chr15	63532088	63532294	1	NM_0011	APH1B	37558	NM_0159	RPS27L	82450	intronic	RAB8B	10.72059	Up	NULL	NULL
peak1753	chr15	63811927	63812097	4	NM_2033	FBXL22	77540	NM_2069	CA12	137703	intronic	USP3	5.46077	Down	NULL	PBS_peak_3822
peak1754	chr15	65146088	65146315	2	NM_1827	ANKDD1	57899	NM_0250	PIF1	28334	intronic	PLEKHO2	4.85859	Up	luks_peak	NULL
peak1755	chr15	65149499	65149731	2	NM_1827	ANKDD1	54486	NM_0250	PIF1	31748	intronic	PLEKHO2	3.81779	Up	NULL	NULL
peak1756	chr15	69098080	69098335	2	NR_03168	MIR548H-	18095	NR_03619	MIR4312	3943	ncRNA_e	ANP32A-	8.29627	Down	NULL	PBS_peak_3837
peak1757	chr15	69099105	69099280	2	NR_03168	MIR548H-	17110	NR_03619	MIR4312	4928	ncRNA_e	ANP32A-	4.34178	Down	NULL	PBS_peak_3838
peak1758	chr15	69100171	69100377	1	NR_03168	MIR548H-	16029	NR_02680	ANP32A-	834	intronic	ANP32A	4.50033	Down	NULL	PBS_peak_3839
peak1759	chr15	72508205	72508367	8	NR_02726	HEXA-AS	160168	NM_0010	GRAMD2	18150	intronic	PKM	4.75	Up	luks_peak	NULL
peak1760	chr15	72517947	72518112	8	NR_02726	HEXA-AS	150424	NM_0010	GRAMD2	27893	intronic	PKM	4.30432	Down	NULL	PBS_peak_3853
peak1761	chr15	72518233	72518384	8	NR_02726	HEXA-AS	150145	NM_0010	GRAMD2	28172	intronic	PKM	4.97108	Down	NULL	PBS_peak_3853
peak1762	chr15	73995662	73995828	2	NR_12035	C15orf59-	64682	NM_0174	NPTN	69992	intronic	CD276	6.83469	Up	luks_peak	NULL
peak1763	chr15	74292851	74293009	9	NM_0011	ISLR2	128785	NM_0012	STOML1	5967	intronic	PML	5.06573	Up	luks_peak	NULL
peak1764	chr15	74295269	74295471	9	NM_0011	ISLR2	126345	NM_0012	STOML1	8407	intronic	PML	4.24283	Up	luks_peak	NULL
peak1765	chr15	74296483	74296694	9	NM_0011	ISLR2	125126	NM_0012	STOML1	9625	intronic	PML	5.55579	Up	luks_peak	NULL
peak1766	chr15	74297080	74297254	9	NM_0011	ISLR2	124548	NM_0012	STOML1	10204	intronic	PML	4.89857	Up	luks_peak	NULL
peak1767	chr15	74297989	74298161	9	NM_0011	ISLR2	123640	NM_0012	STOML1	11112	intronic	PML	3.69916	Up	luks_peak	NULL
peak1768	chr15	74298390	74298598	9	NM_0011	ISLR2	123221	NM_0012	STOML1	11531	intronic	PML	5.13962	Up	luks_peak	NULL
peak1769	chr15	74300387	74300806	9	NM_0011	ISLR2	121118	NM_0012	STOML1	13633	intronic	PML	3.8729	Up	luks_peak	NULL
peak1770	chr15	74301026	74301183	9	NM_0011	ISLR2	120610	NM_0012	STOML1	14141	intronic	PML	4.69418	Up	luks_peak	NULL
peak1771	chr15	74301656	74301958	9	NM_0011	ISLR2	119908	NM_0012	STOML1	14844	intronic	PML	9.64289	Up	luks_peak	NULL
peak1772	chr15	74303985	74304208	9	NM_0011	ISLR2	117618	NM_0012	STOML1	17133	intronic	PML	4.41205	Up	luks_peak	NULL
peak1773	chr15	74306417	74306570	9	NM_0011	ISLR2	115221	NM_0012	STOML1	19530	intronic	PML	6.49706	Up	NULL	NULL
peak1774	chr15	74307182	74307530	9	NM_0011	ISLR2	114359	NM_0012	STOML1	20393	intronic	PML	3.94	Up	luks_peak	NULL
peak1775	chr15	74309158	74309514	9	NM_0011	ISLR2	112379	NM_0012	STOML1	22373	intronic	PML	4.71013	Up	luks_peak	NULL
peak1776	chr15	74310123	74310304	9	NM_0011	ISLR2	111501	NM_0012	STOML1	23250	intronic	PML	7.2003	Up	luks_peak	NULL
peak1777	chr15	74311229	74311503	9	NM_0011	ISLR2	110349	NM_0012	STOML1	24403	intronic	PML	4.27495	Up	luks_peak	NULL
peak1778	chr15	74311584	74311749	9	NM_0011	ISLR2	110048	NM_0012	STOML1	24703	intronic	PML	7.68957	Up	luks_peak	NULL
peak1779	chr15	74312052	74312364	9	NM_0011	ISLR2	109507	NM_0012	STOML1	25245	intronic	PML	5.76715	Up	luks_peak	NULL
peak1780	chr15	74313148	74313464	9	NM_0011	ISLR2	108409	NM_0012	STOML1	26343	intronic	PML	10.13158	Up	luks_peak	NULL
peak1781	chr15	74314437	74314591	9	NM_0011	ISLR2	107201	NM_0012	STOML1	27551	intronic	PML	4.36795	Up	luks_peak	NULL
peak1782	chr15	74314804	74314980	9	NM_0011	ISLR2	106823	NM_0012	STOML1	27929	intronic	PML	6.24142	Up	luks_peak	NULL
peak1783	chr15	74315659	74315820	9	NM_0011	ISLR2	105975	NM_0012	STOML1	28776	exonic	PML	7.48715	Up	luks_peak	NULL
peak1784	chr15	74315972	74316413	9	NM_0011	ISLR2	105522	NM_0012	STOML1	29229	intronic	PML	4.1915	Up	luks_peak	NULL
peak1785	chr15	74316844	74317313	9	NM_0011	ISLR2	104636	NM_0012	STOML1	30115	exonic	PML	7.04418	Up	luks_peak	NULL
peak1786	chr15	74318707	74318860	9	NM_0011	ISLR2	102931	NM_0012	STOML1	31820	intronic	PML	3.43306	Up	NULL	NULL
peak1787	chr15	74319010	74319272	9	NM_0011	ISLR2	102574	NM_0012	STOML1	32178	intronic	PML	9.22617	Up	luks_peak	NULL
peak1788	chr15	74319367	74320071	9	NM_0011	ISLR2	101996	NM_0012	STOML1	32756	intronic	PML	8.11233	Up	luks_peak	NULL
peak1789	chr15	74320140	74320464	9	NM_0011	ISLR2	101413	NM_0012	STOML1	33339	intronic	PML	5.53867	Up	luks_peak	NULL
peak1790	chr15	74321706	74321870	9	NM_0011	ISLR2	99927	NM_0012	STOML1	34825	intronic	PML	4.82467	Up	luks_peak	NULL
peak1791	chr15	74322360	74322567	9	NM_0011	ISLR2	99251	NM_0012	STOML1	35500	intronic	PML	5.97248	Up	luks_peak	NULL
peak1792	chr15	74323648	74323866	9	NM_0011	ISLR2	97958	NM_0012	STOML1	36794	intronic	PML	6.28812	Up	luks_peak	NULL
peak1793	chr15	74325093	74325439	9	NM_0011	ISLR2	96449	NM_0012	STOML1	38303	intronic	PML	5.33244	Up	luks_peak	NULL
peak1794	chr15	74327389	74327632	9	NM_0011	ISLR2	94204	NM_0012	STOML1	40547	exonic	PML	12.30845	Up	luks_peak	NULL
peak1795	chr15	74328423	74328589	9	NM_0011	ISLR2	93209	NM_0012	STOML1	41543	UTR3	PML(NM_	14.99828	Up	NULL	NULL
peak1796	chr15	74329349	74329730	4	NM_0011	ISLR2	92175	NM_0012	STOML1	42576	intronic	PML	4.53062	Up	luks_peak	NULL
peak1797	chr15	74329977	74330140	4	NM_0011	ISLR2	91656	NM_0012	STOML1	43095	intronic	PML	22.64654	Up	NULL	NULL
peak1798	chr15	74331145	74331353	4	NM_0011	ISLR2	90466	NM_0012	STOML1	44286	intronic	PML	23.66447	Up	luks_peak	NULL
peak1799	chr15	74331438	74331608	4	NM_0011	ISLR2	90192	NM_0012	STOML1	44560	intronic	PML	14.34194	Up	luks_peak	NULL
peak1800	chr15	74910977	74911137	2	NM_0007	CYP1A2	130127	NM_2012	UBL7	157528	intronic	CLK3	4.19818	Down	NULL	PBS_peak_3857
peak1801	chr15	75224637	75224791	1	NM_0011	SCAMP5	63162	NM_0204	FAM219B	25252	intronic	COX5A	20.4706	Down	NULL	NULL
peak1802	chr15	75738551	75738820	3	NM_001318146		202662	NM_0067	MAN2C1	77717	intronic	SIN3A	4.42256	Down	NULL	PBS_peak_3866
peak1803	chr15	76593066	76593230	2	NM_1458	ISL2	35917	NR_02851	TYRO3P	40655	intronic	ETFA	3.90898	Down	NULL	PBS_peak_3869
peak1804	chr15	79172510	79172690	5	NR_02970	MIR184	329530	NM_0142	ADAMTS	68795	intronic	MORF4L1	4.36937	Down	NULL	PBS_peak_3872
peak1805	chr15	85298436	85298602	1	NM_0207	ALPK3	61392	NR_07351	SEC11A	38828	intronic	ZNF592	4.51366	Down	NULL	NULL
peak1806	chr15	85300695	85300947	1	NM_0207	ALPK3	59090	NR_07351	SEC11A	41130	intronic	ZNF592	19.16914	Down	NULL	PBS_peak_3884
peak1807	chr15	85301869	85302070	1	NM_0207	ALPK3	57941	NR_07351	SEC11A	42278	intronic	ZNF592	11.71777	Down	NULL	PBS_peak_3885
peak1808	chr15	88391720	88391876	0	NR_03822	NTRK3-A	404163	NR_04601	AGBL1-A	1531599	intergenic	LINC0005	11.68759	Down	NULL	NULL
peak1809	chr15	89167830	89168012	1	NM_0013	ISG20	10947	NR_03986	MIR3529	12766	intronic	AEN	16.75011	Down	NULL	NULL
peak1810	chr15	89169483	89169676	1	NM_0013	ISG20	9288	NR_03986	MIR3529	14424	exonic	AEN	4.37753	Down	NULL	PBS_peak_3908
peak1811	chr15	101828178	101828344	1	NR_132373		46381	NM_0184	VIMP	10536	intronic	SNRPA1	9.4404	Down	NULL	PBS_peak_3915
peak1812	chr16	2057189	2057349	1	NM_0010	NPW	12252	NM_1721	NOXO1	25719	intronic	ZNF598	6.18746	Down	NULL	PBS_peak_3928
peak1813	chr16	3812083	3812246	2	NM_001318918		552597	NM_0162	TRAP1	44566	intronic	CREBBP	6.16945	Up	NULL	NULL
peak1814	chr16	4462485	4462637	4	NM_0011	DNAJA3	13245	NM_0160	PAM16	61188	intronic	CORO7,C	4.75988	Down	NULL	PBS_peak_3960
peak1815	chr16	8931312	8931475	1	NM_0141	C16orf72	254143	NM_0154	TMEM18	39888	intronic	PMM2	4.43068	Down	NULL	PBS_peak_3972
peak1816	chr16	8932770	8933014	1	NM_0141	C16orf72	252645	NM_0154	TMEM18	41387	intronic	PMM2	4.71316	Down	NULL	PBS_peak_3973
peak1817	chr16	8936471	8936657	1	NM_0141	C16orf72	248973	NM_0154	TMEM18	45059	intronic	PMM2	4.01501	Down	NULL	PBS_peak_3975
peak1818	chr16	8940422	8940691	1	NM_0141	C16orf72	244980	NM_0154	TMEM18	49051	intronic	PMM2	4.12426	Down	NULL	PBS_peak_3979
peak1819	chr16	11651758	11651932	4	NM_0034	SNN	110444	NR_135174		212776	intronic	LITAF	4.55443	Up	luks_peak	NULL
peak1820	chr16	11653929	11654253	4	NM_0034	SNN	108198	NR_135174		215022	intronic	LITAF	5.52605	Up	luks_peak	NULL
peak1821	chr16	11656229	11656616	4	NM_0034	SNN	105866	NR_135174		217353	intronic	LITAF	4.03998	Up	luks_peak	NULL
peak1822	chr16	11657942	11658104	4	NM_0034	SNN	104266	NR_135174		218954	intronic	LITAF	4.13222	Up	NULL	NULL
peak1823	chr16	11667121	11667290	4	NM_0034	SNN	95083	NR_135174		228136	intronic	LITAF	6.26891	Up	luks_peak	NULL
peak1824	chr16	11992384	11992565	3	NM_0011	TNFRSF1	66489	NM_0156	RSL1D1	47032	intronic	GSPT1	5.27328	Down	NULL	PBS_peak_4022
peak1825	chr16	12001558	12001742	3	NM_0011	TNFRSF1	57314	NM_0156	RSL1D1	56208	intronic	GSPT1	3.66293	Down	NULL	PBS_peak_4028
peak1826	chr16	18796526	18796726	2	NM_0013	TMC7	198630	NM_1736	NOMO2	223192	intronic	RPS15A	9.83846	Down	NULL	PBS_peak_4039

peak1827	chr16	20871929	20872081	1	NM_0204	LYRM1	39185	NM_0011	ERI2	54210	intronic	DCUN1D	6.20901	Up	NULL	NULL	
peak1828	chr16	20881368	20881555	1	NM_0204	LYRM1	29728	NM_0011	ERI2	63666	intronic	DCUN1D	6.70367	Up	luks_peak	NULL	
peak1829	chr16	20883105	20883310	1	NM_0204	LYRM1	27982	NM_0011	ERI2	65412	intronic	DCUN1D	4.04636	Up	luks_peak	NULL	
peak1830	chr16	20890140	20890352	1	NM_0204	LYRM1	20944	NM_0011	ERI2	72451	intronic	DCUN1D	4.73872	Up	NULL	NULL	
peak1831	chr16	20901158	20901320	1	NM_0204	LYRM1	9951	NM_0011	ERI2	83444	intronic	DCUN1D	5.07967	Up	luks_peak	NULL	
peak1832	chr16	20902834	20903132	1	NM_0204	LYRM1	8207	NM_0011	ERI2	85188	intronic	DCUN1D	8.30189	Up	luks_peak	NULL	
peak1833	chr16	23509786	23510083	1	NM_0191	UBFD1	58927	NM_1536	COG7	45422	intronic	GGA2	7.09634	Down	NULL	PBS_peak_4060	
peak1834	chr16	23511171	23511338	1	NM_0191	UBFD1	57607	NM_1536	COG7	46742	intronic	GGA2	5.7414	Down	NULL	PBS_peak_4062	
peak1835	chr16	23511605	23511778	1	NM_0191	UBFD1	57170	NM_1536	COG7	47179	intronic	GGA2	6.87098	Down	NULL	PBS_peak_4063	
peak1836	chr16	23513234	23513429	1	NM_0191	UBFD1	55530	NM_1536	COG7	48819	intronic	GGA2	12.39676	Down	NULL	PBS_peak_4064	
peak1837	chr16	23513868	23514142	1	NM_0191	UBFD1	54857	NM_1536	COG7	49493	intronic	GGA2	6.36548	Down	NULL	PBS_peak_4065	
peak1838	chr16	23515869	23516067	1	NM_0191	UBFD1	52894	NM_1536	COG7	51456	intronic	GGA2	4.96196	Down	NULL	PBS_peak_4066	
peak1839	chr16	28201952	28202108	2	NM_0010	SBK1	101810	NM_0011	GSG1L	127200	intronic	XPO6	7.25075	Down	NULL	NULL	
peak1840	chr16	28208218	28208386	2	NM_0010	SBK1	95538	NM_0011	GSG1L	133472	intronic	XPO6	3.92622	Down	NULL	NULL	
peak1841	chr16	28836802	28836963	7	NM_0013	SH2B1	21038	NM_1775	SULT1A1	201975	exonic	ATXN2L	5.75119	Down	NULL	PBS_peak_4082	
peak1842	chr16	33492627	33492781	0	NR_1307	ENPP7P1	79140	NR_0338	LOC3907	194002	intergenic	LOC3907	6.04346	Down	NULL	NULL	
peak1843	chr16	34539160	34539315	0	NR_0270	LINC0156	58549	NR_0028	UBE2MP	134475	intergenic	UBE2MP	10.6094	Down	NULL	PBS_peak_4100	
peak1844	chr16	48413543	48413709	3	NM_1446	C16orf78	994093	NM_0010	SIAH1	13842	ncRNA_in	MIR5095	6.43133	Down	NULL	NULL	
peak1845	chr16	48581614	48581764	2	NM_1446	C16orf78	826030	NM_0030	SIAH1	162460	ncRNA_in	MIR5095	4.02752	Up	NULL	NULL	
peak1846	chr16	48584788	48584965	2	NM_1446	C16orf78	822842	NM_0030	SIAH1	165647	ncRNA_in	MIR5095	16.61786	Up	NULL	NULL	
peak1847	chr16	48585749	48585918	2	NM_1446	C16orf78	821885	NM_0030	SIAH1	166604	ncRNA_in	MIR5095	15.56962	Up	NULL	NULL	
peak1848	chr16	48593958	48594274	2	NM_1446	C16orf78	813603	NM_0030	SIAH1	174887	ncRNA_in	MIR5095	4.90891	Up	luks_peak	NULL	
peak1849	chr16	48603305	48603507	2	NM_1446	C16orf78	804313	NM_0030	SIAH1	184177	ncRNA_in	MIR5095	6.67593	Up	NULL	NULL	
peak1850	chr16	48604335	48604582	2	NM_1446	C16orf78	803260	NM_0030	SIAH1	185229	ncRNA_in	MIR5095	3.99126	Up	luks_peak	NULL	
peak1851	chr16	48604868	48605070	2	NM_1446	C16orf78	802750	NM_0030	SIAH1	185740	ncRNA_in	MIR5095	7.697	Up	luks_peak	NULL	
peak1852	chr16	48606175	48606375	2	NM_1446	C16orf78	801444	NM_0030	SIAH1	187046	ncRNA_in	MIR5095	6.06712	Up	NULL	NULL	
peak1853	chr16	48616658	48616828	2	NM_1446	C16orf78	790976	NM_0030	SIAH1	197514	ncRNA_in	MIR5095	5.96166	Up	luks_peak	NULL	
peak1854	chr16	48616898	48617097	2	NM_1446	C16orf78	790721	NM_0030	SIAH1	197768	ncRNA_in	MIR5095	10.83874	Up	luks_peak	NULL	
peak1855	chr16	48620095	48620256	2	NM_1446	C16orf78	787543	NM_0030	SIAH1	200946	ncRNA_in	MIR5095	5.89639	Up	NULL	NULL	
peak1856	chr16	48629616	48629793	2	NM_1446	C16orf78	778014	NM_0030	SIAH1	210475	ncRNA_in	MIR5095	8.24568	Up	NULL	NULL	
peak1857	chr16	48637261	48637555	2	NM_1446	C16orf78	770311	NM_0030	SIAH1	218179	ncRNA_in	MIR5095	4.81399	Up	luks_peak	NULL	
peak1858	chr16	49060181	49060353	0	NM_1446	C16orf78	347452	NM_1530	N4BP1	416147	intergenic	MIR5095(	14.02754	Down	NULL	NULL	
peak1859	chr16	49210269	49210453	0	NM_1446	C16orf78	197358	NM_1530	N4BP1	566241	intergenic	MIR5095(	5.02834	Down	NULL	PBS_peak_4108	
peak1860	chr16	49215695	49215846	0	NM_1446	C16orf78	191948	NM_1530	N4BP1	571650	intergenic	MIR5095(	4.84885	Down	NULL	PBS_peak_4109	
peak1861	chr16	50192857	50193077	2	NM_0012	ADCY7	107484	NM_0012	ZNF423	301137	intronic	PAPD5	3.9921	Down	NULL	PBS_peak_4113	
peak1862	chr16	51312223	51312390	0	NR_1109	LINC0157	484123	NM_0029	SALL1	127123	intergenic	SALL1(di	21.15688	Down	NULL	NULL	
peak1863	chr16	57039573	57039774	0	NM_0322	NLRC5	11312	NM_0059	MT1G	337696	intronic	NLRC5	8.41741	Up	NULL	NULL	
peak1864	chr16	57050714	57050880	0	NM_0322	NLRC5	189	NM_0059	MT1G	348820	intronic	NLRC5	9.29483	Up	NULL	NULL	
peak1865	chr16	58635042	58635337	4	NR_1109	LOC1019	1254067	NR_0029	SNORA7	41354	intronic	CNOT1	8.18158	Down	NULL	PBS_peak_4134	
peak1866	chr16	63632810	63632992	0	NM_0017	CDH5	2767609	NM_0017	CDH8	1562162	intergenic	CDH8(dis	3.96018	Down	NULL	PBS_peak_4154	
peak1867	chr16	64407104	64407293	0	NM_0017	CDH5	1993311	NM_0017	CDH8	2336459	intergenic	NONE(dis	5.64759	Down	NULL	PBS_peak_4155	
peak1868	chr16	64475714	64475872	0	NM_0017	CDH5	1924717	NM_0017	CDH8	2405054	intergenic	NONE(dis	6.49464	Down	NULL	NULL	
peak1869	chr16	64866296	64866458	0	NM_0017	CDH5	1534133	NM_0017	CDH8	2795638	intergenic	NONE(dis	9.00894	Down	NULL	NULL	
peak1870	chr16	67079272	67079425	2	NM_001320540		64447	NR_0461	FAM96B	111022	intronic	CBFB	17.36283	Down	NULL	NULL	
peak1871	chr16	67887567	67887750	1	NM_0143	EDC4	19267	NM_0250	CENPT	6297	intronic	NUTF2	26.73366	Down	NULL	PBS_peak_4181	
peak1872	chr16	67889048	67889216	1	NM_0143	EDC4	17794	NM_0250	CENPT	7771	intronic	NUTF2	5.25889	Down	NULL	PBS_peak_4183	
peak1873	chr16	70568782	70569002	1	NR_0030	SNORD11	3016	NM_0153	COG4	11435	intronic	SF3B3	6.18149	Down	NULL	PBS_peak_4185	
peak1874	chr16	73760726	73760885	0	NM_0028	PSMD7	569867	NM_2073	C16orf47	582459	intergenic	LINC0156	3.68865	Down	NULL	PBS_peak_4191	
peak1875	chr16	74686926	74687111	1	NM_0322	ZNRF1	345896	NR_0272	GLG1	45976	intronic	RFWD3	5.91464	Down	NULL	PBS_peak_4194	
peak1876	chr16	74692700	74692868	1	NM_0322	ZNRF1	340131	NR_0272	GLG1	51742	intronic	RFWD3	7.23947	Down	NULL	PBS_peak_4197	
peak1877	chr16	74694743	74694950	1	NM_0322	ZNRF1	338068	NR_0272	GLG1	53804	exonic	RFWD3	3.72454	Down	NULL	PBS_peak_4199	
peak1878	chr16	80163060	80163235	0	NM_0013	DYNLRB	411483	NR_1046	MAFTRR	358718	intergenic	LINC0122	10.82578	Down	NULL	NULL	
peak1879	chr16	82770907	82771065	6	NR_1109	LOC1019	35938	NM_0057	MPHOSP	567157	intronic	CDH13	6.90321	Down	NULL	PBS_peak_4251	
peak1880	chr16	84626705	84626873	1	NM_0013	KLHL36	55328	NM_0209	TLDC1	88501	intronic	COTL1	6.86215	Down	NULL	PBS_peak_4256	
peak1881	chr16	84737896	84738128	4	NM_0314	CRISPLD	115575	NM_0211	COTL1	86310	intronic	USP10	11.89936	Down	NULL	PBS_peak_4258	
peak1882	chr16	84744560	84744743	4	NM_0314	CRISPLD	108935	NM_0211	COTL1	92949	intronic	USP10	5.31277	Down	NULL	PBS_peak_4263	
peak1883	chr16	84745387	84745543	4	NM_0314	CRISPLD	108122	NM_0211	COTL1	93763	intronic	USP10	3.92158	Down	NULL	PBS_peak_4265	
peak1884	chr16	85653019	85653183	3	NR_135193		172920	NR_0498	MIR5093	313170	intronic	GSE1	14.19285	Down	NULL	PBS_peak_4273	
peak1885	chr16	85654389	85654635	3	NR_135193		171509	NR_0498	MIR5093	314581	intronic	GSE1	5.22943	Down	NULL	PBS_peak_4275	
peak1886	chr16	85655824	85656003	3	NR_135193		170107	NR_0498	MIR5093	315982	intronic	GSE1	7.19095	Down	NULL	PBS_peak_4276	
peak1887	chr16	85656077	85656549	3	NR_135193		169708	NR_0498	MIR5093	316382	intronic	GSE1	5.97429	Down	NULL	PBS_peak_4276	
peak1888	chr16	85659927	85660107	3	NR_135193		166004	NR_0498	MIR5093	320086	intronic	GSE1	4.75019	Down	NULL	PBS_peak_4280	
peak1889	chr16	85661554	85661729	3	NR_135193		164379	NR_0498	MIR5093	321710	intronic	GSE1	6.2918	Down	NULL	PBS_peak_4282	
peak1890	chr16	85665499	85665831	3	NR_135193		160356	NR_0498	MIR5093	325734	intronic	GSE1	9.42996	Down	NULL	PBS_peak_4286	
peak1891	chr16	85677210	85677394	3	NR_135193		148719	NR_0498	MIR5093	337371	intronic	GSE1	4.48173	Down	NULL	PBS_peak_4295	
peak1892	chr16	85680716	85680873	3	NR_135193		145226	NR_0498	MIR5093	340863	intronic	GSE1	16.17505	Down	NULL	PBS_peak_4296	
peak1893	chr16	85827994	85828159	2	NM_001318786		5096	NM_2069	C16orf74	43387	intronic	EMC8	12.71125	Down	NULL	NULL	
peak1894	chr16	87400030	87400186	2	NM_0228	MAP1LC	25693	NM_0012	C16orf95	49082	intronic	FBXO31	6.08108	Down	NULL	NULL	
peak1895	chr16	89360380	89360535	4	NM_0012	LOC1002	27083	NM_0012	SLC22A3	93928	intronic	ANKRD1	3.50287	Down	NULL	PBS_peak_4307	
peak1896	chr16	89380751	89380922	4	NM_0012	LOC1002	6704	NM_0012	SLC22A3	114307	intronic	ANKRD1	5.84923	Down	NULL	PBS_peak_4319	
peak1897	chr16	89383400	89383581	4	NM_0012	LOC1002	4050	NM_0012	SLC22A3	116961	exonic	ANKRD1	5.5006	Down	NULL	PBS_peak_4321	
peak1898	chr16	89386239	89386450	4	NM_0012	LOC1002	1196	NM_0012	SLC22A3	119815	intronic	ANKRD1	20.99633	Down	NULL	PBS_peak_4323	
peak1899	chr16	89394384	89394553	4	NR_1109	LOC1019	102857	NM_0012	SLC22A3	127939	intronic	ANKRD1	5.59491	Down	NULL	PBS_peak_4326	
peak1900	chr16	89397519	89397674	4	NR_1109	LOC1019	99729	NM_0012	SLC22A3	131067	intronic	ANKRD1	8.46456	Down	NULL	PBS_peak_4327	
peak1901	chr16	89398054	89398366	4	NR_1109	LOC1019	99116	NM_0012	SLC22A3	131681	intronic	ANKRD1	5.87708	Down	NULL	PBS_peak_4328	
peak1902	chr16	89399021	89399200	4	NR_1109	LOC1019	98215	NM_0012	SLC22A3	132581	intronic	ANKRD1	8.89528	Down	NULL	PBS_peak_4329	
peak1903	chr16	89401777	89402028	4	NR_1109	LOC1019	95423	NM_0012	SLC22A3	135373	intronic	ANKRD1	5.08619	Down	NULL	PBS_peak_4332	
peak1904	chr16	89406853	89407050	4	NR_1109	LOC1019	90374	NM_0012	SLC22A3	140422	intronic	ANKRD1	12.42643	Down	NULL	PBS_peak_4335	
peak1905	chr16	89408997	89409172	4	NR_1109	LOC1019	88241	NM_0012	SLC22A3	142555	intronic	ANKRD1	5.04114	Down	NULL	PBS_peak_4337	
peak1906	chr16	89412126	89412346	4	NR_1109	LOC1019	85090	NM_0012	SLC22A3	145707	intronic	ANKRD1	8.31969	Down	NULL	PBS_peak_4339	
peak1907	chr16	89412517	89412680	4	NR_1109	LOC1019	84727	NM_0012	SLC22A3	146069	intronic	ANKRD1	3.45688	Down	NULL	PBS_peak_4339	
peak1908	chr16	89412745	89413318	4	NR_1109	LOC1019	84294	NM_0012	SLC22A3	146502	intronic	ANKRD1	4.70632	Down	NULL	PBS_peak_4339	
peak1909	chr16	89413660	89413892	4	NR_1109	LOC1019	83550	NM_0012	SLC22A3	147247	intronic	ANKRD1	8.86381	Down	NULL	PBS_peak_4339	
peak1910	chr16	89416676	89416827	4	NR_1109	LOC1019	80574	NM_0012	SLC22A3	150222	intronic	ANKRD1	4.78774	Down	NULL	PBS_peak_4342	
peak1911	chr16	89422773	89423015	4	NR_1109	LOC1019	74432	NM_0012	SLC22A3	156365	intronic	ANKRD1	6.32732	Down	NULL	PBS_peak_4344	
peak1912	chr16	89424091	89424241	4	NR_1109	LOC1019	73160	NM_0012	SLC22A3	157637	intronic	ANKRD1	3.95158	Down	NULL	PBS_peak_4345	
peak1913	chr16	89427761	89428115	4	NR_1109	LOC1019	69388	NM_0012	SLC22A3	161409	intronic	ANKRD1	6.59763	Down	NULL	PBS_peak_4347	
peak1914	chr16	89428661	89428909	4	NR_1109	LOC1019	68541	NM_0012	SLC22A3	162256	intronic	ANKRD1	6.62189	Down	NULL	PBS_peak_4348	
peak1915	chr16	89430901	89431135	4	NR_1109	LOC1019	66308	NM_0012	SLC22A3	164489	intronic	ANKRD1	4.8143	Down	NULL	PBS_peak_4349	
peak1916	chr16	89440345	89440508	4	NR_1109	LOC1019	56899	NM_0012	SLC22A3	173897	intronic	ANKRD1	3.98551	Down	NULL	PBS_peak_4355	
peak1917	chr16	89442959	89443114	4	NR_1109	LOC1019	54289	NM_0012	SLC22A3	176507	intronic	ANKRD1	5.90853	Down	NULL	PBS_peak_4358	
peak1918	chr16	89443914	89444154	4	NR_1109	LOC1019	53292	NM_0012	SLC22A3	177505	intronic	ANKRD1	3.81035	Down	NULL	PBS_peak_4358	
peak1919	chr16	89450010	89450199	4	NR_1109	LOC1019	47221	NM_0012	SLC22A3	183575	intronic	ANKRD1	5.63133	Down	NULL	PBS_peak_4363	
peak1920	chr16	89453433	89453595	4	NR_1109	LOC1019	43812	NM_0012	SLC22A3	186985	intronic	ANKRD1	4.39108	Down	NULL	PBS_peak_4365	
peak1921	chr16	89457150	89457366	4	NR_1109	LOC1019	40068	NM_0012	SLC22A3	190729	intronic	ANKRD1	6.14526	Down	NULL	PBS_peak_4369	
peak1922	chr16	89459272	89459497	4	NR_1109	LOC1019	37941	NM_0012	SLC22A3	192855	intronic	ANKRD1	5.19497	Down	NULL	PBS_peak_4372	
peak1923	chr16	89462322	89462602	4	NR_1109	LOC1019	34864	NM_0012	SLC22A3	195933	intronic	ANKRD1	3.95369	Down	NULL	PBS_peak_4374	
peak1924	chr16	89462819	89463130	4	NR_1109	LOC1019	34351	NM_0012	SLC22A3	196445	intronic	ANKRD1	4.56293	Down	NULL	PBS_peak_4374	
peak1925	chr16	89463871	89464065	4	NR_1109	LOC1019	33358	NM_0012	SLC22A3	197439	intronic	ANKRD1	5.27231	Down	NULL	PBS_peak_4375	
peak1926	chr16	89467544	89467818	4	NR_1109	LOC1019	29645	NM_0012	SLC22A3	201152	intronic	ANKRD1	7.85689	Down	NULL	PBS_peak_4377	
peak1927	chr16	89469556	89469792	4	NR_1109	LOC1019	27652	NM_0012	SLC22A3	203145	intronic	ANKRD1	12.58066	Down	NULL	PBS_peak_4378	
peak1928	chr16	89469963	89470128	4	NR_1109	LOC1019	27280	NM_0012	SLC22A3	203516	intronic	ANKRD1	5.57338	Down	NULL	PBS_peak_4378	
peak1929	chr16	89475489	89475835	4	NR_1109	LOC1019	21664	NM_0012	SLC22A3	209133	intronic	ANKRD1	3.96904	Down	NULL	PBS_peak_4382	

peak1930	chr16	89481296	89481557	4	NR_11093	LOC10192	15899	NM_0012	SLC22A3	214897	intronic	ANKRD1	11.46504	Down	NULL	PBS_peak_4384
peak1931	chr16	89482260	89482486	4	NR_11093	LOC10192	14953	NM_0012	SLC22A3	215844	intronic	ANKRD1	3.45922	Down	NULL	PBS_peak_4384
peak1932	chr16	89483103	89483257	4	NR_11093	LOC10192	14146	NM_0012	SLC22A3	216651	intronic	ANKRD1	4.83297	Down	NULL	PBS_peak_4384
peak1933	chr16	89483798	89484000	4	NR_11093	LOC10192	13427	NM_0012	SLC22A3	217370	intronic	ANKRD1	5.61029	Down	NULL	PBS_peak_4384
peak1934	chr16	89484108	89484533	4	NR_11093	LOC10192	13005	NM_0012	SLC22A3	217791	intronic	ANKRD1	8.25067	Down	NULL	PBS_peak_4384
peak1935	chr16	89484742	89484959	4	NR_11093	LOC10192	12475	NM_0012	SLC22A3	218321	UTR5	ANKRD1	8.03133	Down	NULL	PBS_peak_4384
peak1936	chr16	89485168	89485479	4	NR_11093	LOC10192	12002	NM_0012	SLC22A3	218794	intronic	ANKRD1	7.90041	Down	NULL	PBS_peak_4384
peak1937	chr16	89485546	89485794	4	NR_11093	LOC10192	11656	NM_0012	SLC22A3	219141	intronic	ANKRD1	5.92272	Down	NULL	PBS_peak_4384
peak1938	chr16	89486639	89486838	4	NR_11093	LOC10192	10587	NM_0012	SLC22A3	220209	intronic	ANKRD1	7.05229	Down	NULL	PBS_peak_4385
peak1939	chr16	89487128	89487347	4	NR_11093	LOC10192	10088	NM_0012	SLC22A3	220708	intronic	ANKRD1	6.15999	Down	NULL	PBS_peak_4386
peak1940	chr16	89488322	89488543	4	NR_11093	LOC10192	8893	NM_0012	SLC22A3	221903	intronic	ANKRD1	4.84913	Down	NULL	PBS_peak_4387
peak1941	chr16	89488761	89489019	4	NR_11093	LOC10192	8436	NM_0012	SLC22A3	222361	intronic	ANKRD1	6.64961	Down	NULL	PBS_peak_4387
peak1942	chr16	89489462	89489905	4	NR_11093	LOC10192	7642	NM_0012	SLC22A3	223154	intronic	ANKRD1	5.85624	Down	NULL	PBS_peak_4388
peak1943	chr16	89489972	89490541	4	NR_11093	LOC10192	7069	NM_0012	SLC22A3	223727	intronic	ANKRD1	9.00919	Down	NULL	PBS_peak_4388
peak1944	chr16	89490828	89491029	4	NR_11093	LOC10192	6397	NM_0012	SLC22A3	224399	intronic	ANKRD1	4.71671	Down	NULL	PBS_peak_4388
peak1945	chr16	89491252	89491458	4	NR_11093	LOC10192	5971	NM_0012	SLC22A3	224826	intronic	ANKRD1	4.972	Down	NULL	PBS_peak_4388
peak1946	chr16	89491628	89491800	4	NR_11093	LOC10192	5612	NM_0012	SLC22A3	225185	intronic	ANKRD1	6.30315	Down	NULL	PBS_peak_4389
peak1947	chr16	89491892	89492059	4	NR_11093	LOC10192	5350	NM_0012	SLC22A3	225446	intronic	ANKRD1	4.57426	Down	NULL	PBS_peak_4389
peak1948	chr16	89492429	89492626	4	NR_11093	LOC10192	4798	NM_0012	SLC22A3	225998	intronic	ANKRD1	7.49724	Down	NULL	PBS_peak_4390
peak1949	chr16	89492688	89493300	4	NR_11093	LOC10192	4332	NM_0012	SLC22A3	226465	intronic	ANKRD1	6.53589	Down	NULL	PBS_peak_4390
peak1950	chr16	89494405	89494621	4	NR_11093	LOC10192	2813	NM_0012	SLC22A3	227984	intronic	ANKRD1	4.55594	Down	NULL	PBS_peak_4392
peak1951	chr16	89496742	89496969	4	NR_11093	LOC10192	470	NM_0012	SLC22A3	230326	intronic	ANKRD1	14.75054	Down	NULL	PBS_peak_4393
peak1952	chr16	89497474	89497862	6	NM_1993	SPG7	77128	NM_0012	SLC22A3	231139	ncRNA_ex	LOC10192	6.69597	Down	NULL	PBS_peak_4393
peak1953	chr16	89498324	89498517	6	NM_1993	SPG7	76375	NM_0012	SLC22A3	231891	ncRNA_in	LOC10192	5.50028	Down	NULL	PBS_peak_4393
peak1954	chr16	89499484	89499648	6	NM_1993	SPG7	75230	NM_0012	SLC22A3	233037	ncRNA_in	LOC10192	4.52679	Down	NULL	PBS_peak_4394
peak1955	chr16	89500155	89500401	6	NM_1993	SPG7	74518	NM_0012	SLC22A3	233749	ncRNA_in	LOC10192	4.2058	Down	NULL	PBS_peak_4394
peak1956	chr16	89501491	89501745	6	NM_1993	SPG7	73178	NM_0012	SLC22A3	235089	ncRNA_in	LOC10192	4.10991	Down	NULL	PBS_peak_4394
peak1957	chr16	89503452	89503681	6	NM_1993	SPG7	71229	NM_0012	SLC22A3	237037	ncRNA_in	LOC10192	6.60711	Down	NULL	PBS_peak_4395
peak1958	chr16	89507522	89507685	6	NM_1993	SPG7	67192	NM_0012	SLC22A3	241074	ncRNA_in	LOC10192	6.83707	Down	NULL	PBS_peak_4397
peak1959	chr16	89510205	89510380	6	NM_1993	SPG7	64503	NM_0012	SLC22A3	243763	ncRNA_in	LOC10192	4.32196	Down	NULL	PBS_peak_4398
peak1960	chr16	89510621	89510895	6	NM_1993	SPG7	64038	NM_0012	SLC22A3	244229	ncRNA_ex	LOC10192	6.21798	Down	NULL	PBS_peak_4398
peak1961	chr16	89512029	89512271	6	NM_1993	SPG7	62646	NM_0012	SLC22A3	245621	ncRNA_in	LOC10192	3.61741	Down	NULL	PBS_peak_4398
peak1962	chr16	89513305	89513601	6	NM_1993	SPG7	61343	NM_0012	SLC22A3	246924	ncRNA_in	LOC10192	4.80698	Down	NULL	PBS_peak_4399
peak1963	chr16	89514347	89514511	6	NM_1993	SPG7	60367	NM_0012	SLC22A3	247900	ncRNA_in	LOC10192	3.70488	Down	NULL	PBS_peak_4400
peak1964	chr16	89518502	89518800	6	NM_1993	SPG7	56145	NM_0012	SLC22A3	252122	ncRNA_in	LOC10192	4.3988	Down	NULL	PBS_peak_4402
peak1965	chr16	89522445	89522608	4	NM_1993	SPG7	52269	NM_0012	SLC22A3	255997	intronic	ANKRD1	3.86185	Down	NULL	PBS_peak_4405
peak1966	chr16	89538159	89538348	4	NM_1993	SPG7	36542	NM_0012	SLC22A3	271724	intronic	ANKRD1	17.13477	Down	NULL	PBS_peak_4415
peak1967	chr16	89542682	89543050	4	NM_1993	SPG7	31930	NM_0012	SLC22A3	276337	intronic	ANKRD1	5.12033	Down	NULL	PBS_peak_4416
peak1968	chr17	18090921	18091082	1	NM_0041	LLGL1	37934	NM_1456	ATPAF2	148521	intronic	ALKBH5	10.70488	Down	NULL	PBS_peak_4456
peak1969	chr17	21889185	21889372	0	NR_02708	FLJ36000	14783	NM_0011	C17orf51	434337	intergenic	FAM27E5	7.05395	Up	NULL	NULL
peak1970	chr17	25961149	25961303	3	NM_0162	NLK	408462	NR_03974	MIR4522	340204	intronic	LGALS9	3.96987	Up	luks_peak	NULL
peak1971	chr17	27256796	27256962	4	NM_0165	PIPOX	113039	NM_1446	DHRS13	26790	ncRNA_in	LOC10192	8.35844	Down	NULL	NULL
peak1972	chr17	27257716	27258083	4	NM_0165	PIPOX	112018	NM_1446	DHRS13	27810	ncRNA_in	LOC10192	11.80622	Down	NULL	PBS_peak_4466
peak1973	chr17	27261194	27261528	4	NM_0165	PIPOX	108557	NM_1446	DHRS13	31272	ncRNA_in	LOC10192	11.84479	Down	NULL	PBS_peak_4468
peak1974	chr17	27262318	27262583	4	NM_0165	PIPOX	107467	NM_1446	DHRS13	32361	ncRNA_in	LOC10192	28.66179	Down	NULL	PBS_peak_4469
peak1975	chr17	27262769	27262961	4	NM_0165	PIPOX	107053	NM_1446	DHRS13	32776	ncRNA_in	LOC10192	7.3875	Down	NULL	PBS_peak_4470
peak1976	chr17	27266738	27267000	4	NM_0165	PIPOX	103049	NM_1446	DHRS13	36780	ncRNA_in	LOC10192	12.25391	Down	NULL	PBS_peak_4472
peak1977	chr17	27268679	27269017	4	NM_0165	PIPOX	101070	NM_1446	DHRS13	38759	ncRNA_in	LOC10192	5.38856	Down	NULL	PBS_peak_4473
peak1978	chr17	27270959	27271109	4	NM_0165	PIPOX	98884	NM_1446	DHRS13	40945	ncRNA_in	LOC10192	4.73025	Down	NULL	PBS_peak_4475
peak1979	chr17	30684587	30684746	3	NM_0012	PSMD11	86814	NM_0223	C17orf75	15438	intronic	ZNF207	4.46778	Down	NULL	PBS_peak_4499
peak1980	chr17	30689208	30689367	3	NM_0012	PSMD11	82193	NM_0223	C17orf75	20059	intronic	ZNF207	6.56899	Down	NULL	PBS_peak_4503
peak1981	chr17	34933294	34933509	1	NM_0243	DHRS11	14824	NM_0010	MYO19	42096	intronic	GGNBP2	4.68405	Down	NULL	PBS_peak_4513
peak1982	chr17	40287555	40287735	3	NM_0031	STAT5A	151920	NM_0210	KAT2A	14263	intronic	RAB5C	4.31936	Down	NULL	PBS_peak_4521
peak1983	chr17	40299753	40299937	3	NM_0031	STAT5A	139720	NM_0210	KAT2A	26463	intronic	RAB5C	3.76307	Down	NULL	PBS_peak_4532
peak1984	chr17	40532073	40532237	3	NM_0011	ATP6V0A	78707	NM_0124	STAT5B	103731	intronic	STAT3	4.23519	Up	NULL	NULL
peak1985	chr17	41370854	41371024	4	NM_0016	ARL4D	105414	NR_11086	LOC10192	48519	UTR3	TMEM10	10.21206	Up	NULL	NULL
peak1986	chr17	41372275	41372452	0	NM_0016	ARL4D	103989	NR_11086	LOC10192	49943	downstrea	LINC0085	28.02042	Up	NULL	NULL
peak1987	chr17	47869268	47869592	5	NM_0012	FLJ45513	53842	NM_0308	FAM117A	27912	exonic	KAT7	6.42885	Down	NULL	PBS_peak_4548
peak1988	chr17	48804126	48804308	2	NR_10702	MIR8059	41794	NM_0528	ANKRD4	18947	intronic	LUC7L3	10.57724	Down	NULL	PBS_peak_4554
peak1989	chr17	48810064	48810298	2	NR_10702	MIR8059	35830	NM_0528	ANKRD4	24911	intronic	LUC7L3	6.57808	Down	NULL	PBS_peak_4559
peak1990	chr17	52668856	52669039	0	NM_0054	TOM1L1	309104	NM_0010	CA10	2431570	intergenic	KIF2B(dis	17.16892	Down	NULL	PBS_peak_4567
peak1991	chr17	54970818	54971014	1	NM_0216	SCPEP1	84552	NR_03740	MIR3614	2200	intronic	TRIM25	6.75252	Up	luks_peak	NULL
peak1992	chr17	54973439	54973627	1	NM_0216	SCPEP1	81935	NR_03740	MIR3614	4817	intronic	TRIM25	4.2305	Up	NULL	NULL
peak1993	chr17	54974743	54974894	1	NM_0216	SCPEP1	80649	NR_03740	MIR3614	6102	intronic	TRIM25	5.19223	Up	NULL	NULL
peak1994	chr17	54977417	54977620	1	NM_0216	SCPEP1	77949	NR_03740	MIR3614	8802	intronic	TRIM25	3.80514	Up	luks_peak	NULL
peak1995	chr17	54979691	54979891	1	NM_0216	SCPEP1	75677	NR_03740	MIR3614	11075	intronic	TRIM25	8.20893	Up	luks_peak	NULL
peak1996	chr17	55181379	55181682	3	NM_1389	MSI2	152400	NM_0046	COIL	143119	intronic	AKAP1	3.88746	Down	NULL	PBS_peak_4577
peak1997	chr17	57775538	57775716	2	NM_0309	VMP1	9236	NR_1108	LINC0147	171409	intronic	PTRH2	4.54676	Down	NULL	PBS_peak_4591
peak1998	chr17	57777771	57777928	2	NM_0309	VMP1	7013	NR_1108	LINC0147	173631	intronic	PTRH2	5.13866	Down	NULL	PBS_peak_4593
peak1999	chr17	57778160	57778319	2	NM_0309	VMP1	6623	NR_1108	LINC0147	174021	intronic	PTRH2	4.0311	Down	NULL	PBS_peak_4594
peak2000	chr17	61853617	61853789	2	NM_0028	PSMC5	51067	NM_0201	CCDC47	2615	intronic	DDX42	5.05644	Down	NULL	PBS_peak_4599
peak2001	chr17	61860319	61860471	2	NM_0028	PSMC5	44375	NM_0201	CCDC47	9307	intronic	DDX42	4.05325	Down	NULL	PBS_peak_4602
peak2002	chr17	72203845	72204011	2	NM_0326	TTYH2	5768	NR_0271	LINC0046	379252	intronic	RPL38	4.33279	Down	NULL	PBS_peak_4610
peak2003	chr17	73362792	73363110	2	NR_0374	MIR3678	39199	NM_0217	SLC25A1	77421	intronic	GRB2	7.04917	Down	NULL	PBS_peak_4632
peak2004	chr17	74439565	74439720	1	NM_0011	AANAT	9790	NM_0027	PRPSAP1	89412	intronic	UBE2O	21.02504	Down	NULL	PBS_peak_4665
peak2005	chr17	76718520	76718760	3	NM_1533	C1QTNF1	300376	NM_0012	CYTH1	5526	intronic	CYTH1	13.22471	Up	luks_peak	NULL
peak2006	chr17	76720836	76720986	2	NM_1533	C1QTNF1	298105	NM_0012	CYTH1	1079	intronic	CYTH1	4.38404	Up	NULL	NULL
peak2007	chr17	76723738	76723979	2	NM_1533	C1QTNF1	295157	NM_0012	CYTH1	4026	intronic	CYTH1	5.40764	Up	luks_peak	NULL
peak2008	chr17	76725409	76725578	2	NM_1533	C1QTNF1	293522	NM_0012	CYTH1	5661	intronic	CYTH1	5.61406	Up	NULL	NULL
peak2009	chr17	76755292	76755462	2	NM_1533	C1QTNF1	263639	NM_0012	CYTH1	35545	intronic	CYTH1	3.72328	Up	NULL	NULL
peak2010	chr17	76760307	76760547	2	NM_1533	C1QTNF1	258589	NM_0012	CYTH1	40595	intronic	CYTH1	4.04591	Up	luks_peak	NULL
peak2011	chr17	76764287	76764514	2	NM_1533	C1QTNF1	254615	NM_0012	CYTH1	44568	intronic	CYTH1	15.28838	Up	NULL	NULL
peak2012	chr17	76764947	76765099	2	NM_1533	C1QTNF1	253993	NM_0012	CYTH1	45191	intronic	CYTH1	4.90986	Up	NULL	NULL
peak2013	chr17	76765534	76765688	2	NM_1533	C1QTNF1	253405	NM_0012	CYTH1	45779	intronic	CYTH1	5.12704	Up	NULL	NULL
peak2014	chr17	78240330	78240480	2	NM_0011	ENDOV	148562	NM_0001	SGSH	46206	intronic	RNF213	4.10291	Up	luks_peak	NULL
peak2015	chr17	78240592	78240800	2	NM_0011	ENDOV	148271	NM_0001	SGSH	46497	intronic	RNF213	3.79628	Up	luks_peak	NULL
peak2016	chr17	78241675	78242068	2	NM_0011	ENDOV	147095	NM_0001	SGSH	47672	intronic	RNF213	10.68625	Up	luks_peak	NULL
peak2017	chr17	78242906	78243417	2	NM_0011	ENDOV	145805	NM_0001	SGSH	48962	intronic	RNF213	5.33282	Up	luks_peak	NULL
peak2018	chr17	78243518	78243676	2	NM_0011	ENDOV	145370	NM_0001	SGSH	49398	intronic	RNF213	7.25125	Up	luks_peak	NULL
peak2019	chr17	78243742	78244015	2	NM_0011	ENDOV	145088	NM_0001	SGSH	49679	intronic	RNF213	8.60247	Up	luks_peak	NULL
peak2020	chr17	78245149	78245460	2	NM_0011	ENDOV	143662	NM_0001	SGSH	51105	intronic	RNF213	6.62828	Up	luks_peak	NULL
peak2021	chr17	78245545	78245769	2	NM_0011	ENDOV	143310	NM_0001	SGSH	51458	intronic	RNF213	5.54562	Up	NULL	NULL
peak2022	chr17	78246977	78247148	2	NM_0011	ENDOV	141904	NM_0001	SGSH	52863	exonic	RNF213	3.91263	Up	luks_peak	NULL
peak2023	chr17	78248071	78248305	2	NM_0011	ENDOV	140779	NM_0001	SGSH	53989	intronic	RNF213	6.22533	Up	luks_peak	NULL
peak2024	chr17	78248808	78249057	2	NM_0011	ENDOV	140034	NM_0001	SGSH	54733	intronic	RNF213	5.06693	Up	luks_peak	NULL
peak2025	chr17	78249528	78249693	2	NM_0011	ENDOV	139356	NM_0001	SGSH	55411	intronic	RNF213	4.73878	Up	luks_peak	NULL
peak2026	chr17	78251483	78251642	2	NM_0011	ENDOV	137404	NM_0001	SGSH	57363	intronic	RNF213	4.65589	Up	NULL	NULL
peak2027	chr17	78252320	78252492	2	NM_0011	ENDOV	136561	NM_0001	SGSH	58207	intronic	RNF213	8.46002	Up	NULL	NULL
peak2028	chr17	78253707	78254393	2	NM_0011	ENDOV	134917	NM_0001	SGSH	59851	intronic	RNF213	7.00451	Up	luks_peak	NULL
peak2029	chr17	78254651	78254820	2	NM_0011	ENDOV	134231	NM_0001	SGSH	60536	intronic	RNF213	4.92442	Up	NULL	NULL
peak2030	chr17	78255459	78255740	2	NM_0011	ENDOV	133367	NM_0001	SGSH	61400	intronic	RNF213	6.39315	Up	NULL	NULL
peak2031	chr17	78257245	78257815	2	NM_0011	ENDOV	131437	NM_0001	SGSH	63331	intronic	RNF213	24.9004	Up	luks_peak	NULL
peak2032	chr17	78258521	78258879	2	NM_0011	ENDOV	130267	NM_0001	SGSH	64501	intronic	RNF213	14.48451	Up	luks_peak	NULL

peak2033	chr17	78259290		78259523	2	NM_0011	ENDOV	129560	NM_0001	SGSH	65207	intronic	RNF213	7.61419	Up	luks_peak	NULL
peak2034	chr17	78263961		78264129	2	NM_0011	ENDOV	124922	NM_0001	SGSH	69846	intronic	RNF213	6.53852	Up	NULL	NULL
peak2035	chr17	78264291		78264456	2	NM_0011	ENDOV	124593	NM_0001	SGSH	70174	exonic	RNF213	12.22754	Up	NULL	NULL
peak2036	chr17	78264636		78265006	2	NM_0011	ENDOV	124146	NM_0001	SGSH	70622	intronic	RNF213	23.96749	Up	luks_peak	NULL
peak2037	chr17	78265535		78265863	2	NM_0011	ENDOV	123268	NM_0001	SGSH	71500	exonic	RNF213	12.55899	Up	luks_peak	NULL
peak2038	chr17	78266071		78266365	2	NM_0011	ENDOV	122749	NM_0001	SGSH	72019	intronic	RNF213	5.32933	Up	luks_peak	NULL
peak2039	chr17	78266542		78266716	2	NM_0011	ENDOV	122338	NM_0001	SGSH	72430	intronic	RNF213	5.76429	Up	luks_peak	NULL
peak2040	chr17	78266895		78267089	2	NM_0011	ENDOV	121975	NM_0001	SGSH	72793	intronic	RNF213	6.45937	Up	NULL	NULL
peak2041	chr17	78268981		78269254	2	NM_0011	ENDOV	119849	NM_0001	SGSH	74918	intronic	RNF213	8.12076	Up	luks_peak	NULL
peak2042	chr17	78269713		78270222	2	NM_0011	ENDOV	118999	NM_0001	SGSH	75768	intronic	RNF213	12.69513	Up	luks_peak	NULL
peak2043	chr17	78270501		78270772	2	NM_0011	ENDOV	118330	NM_0001	SGSH	76437	intronic	RNF213	16.79707	Up	luks_peak	NULL
peak2044	chr17	78270915		78271372	2	NM_0011	ENDOV	117823	NM_0001	SGSH	76944	intronic	RNF213	7.58338	Up	luks_peak	NULL
peak2045	chr17	78271598		78271768	2	NM_0011	ENDOV	117284	NM_0001	SGSH	77484	intronic	RNF213	5.38468	Up	luks_peak	NULL
peak2046	chr17	78272007		78272648	2	NM_0011	ENDOV	116639	NM_0001	SGSH	78128	exonic	RNF213	4.93859	Up	luks_peak	NULL
peak2047	chr17	78274516		78274898	2	NM_0011	ENDOV	114260	NM_0001	SGSH	80508	intronic	RNF213	10.10058	Up	luks_peak	NULL
peak2048	chr17	78275107		78275549	2	NM_0011	ENDOV	113639	NM_0001	SGSH	81129	intronic	RNF213	12.95574	Up	luks_peak	NULL
peak2049	chr17	78275711		78276004	2	NM_0011	ENDOV	113109	NM_0001	SGSH	81658	intronic	RNF213	5.44785	Up	luks_peak	NULL
peak2050	chr17	78276753		78277341	2	NM_0011	ENDOV	111920	NM_0001	SGSH	82848	intronic	RNF213	7.35999	Up	luks_peak	NULL
peak2051	chr17	78278267		78278434	2	NM_0011	ENDOV	110616	NM_0001	SGSH	84151	intronic	RNF213	17.97222	Up	luks_peak	NULL
peak2052	chr17	78278925		78279340	2	NM_0011	ENDOV	109834	NM_0001	SGSH	84933	intronic	RNF213	23.99835	Up	luks_peak	NULL
peak2053	chr17	78279510		78279702	2	NM_0011	ENDOV	109361	NM_0001	SGSH	85407	intronic	RNF213	5.1538	Up	luks_peak	NULL
peak2054	chr17	78279856		78280151	2	NM_0011	ENDOV	108963	NM_0001	SGSH	85804	exonic	RNF213	17.56131	Up	luks_peak	NULL
peak2055	chr17	78282015		78282524	2	NM_0011	ENDOV	106697	NM_0001	SGSH	88070	intronic	RNF213	9.82794	Up	luks_peak	NULL
peak2056	chr17	78283058		78283261	2	NM_0011	ENDOV	105807	NM_0001	SGSH	88960	intronic	RNF213	23.78371	Up	luks_peak	NULL
peak2057	chr17	78284047		78284200	2	NM_0011	ENDOV	104843	NM_0001	SGSH	89924	intronic	RNF213	8.95243	Up	luks_peak	NULL
peak2058	chr17	78285003		78285346	2	NM_0011	ENDOV	103792	NM_0001	SGSH	90975	intronic	RNF213	11.67557	Up	luks_peak	NULL
peak2059	chr17	78286571		78286992	2	NM_0011	ENDOV	102185	NM_0001	SGSH	92582	exonic	RNF213	10.14766	Up	luks_peak	NULL
peak2060	chr17	78290791		78291105	2	NM_0011	ENDOV	98019	NM_0001	SGSH	96749	exonic	RNF213	6.3823	Up	luks_peak	NULL
peak2061	chr17	78291676		78291922	2	NM_0011	ENDOV	97168	NM_0001	SGSH	97600	intronic	RNF213	4.08501	Up	luks_peak	NULL
peak2062	chr17	78292025		78292239	2	NM_0011	ENDOV	96835	NM_0001	SGSH	97933	intronic	RNF213	7.66112	Up	luks_peak	NULL
peak2063	chr17	78293597		78293767	2	NM_0011	ENDOV	95285	NM_0001	SGSH	99483	UTR3	RNF213(N	7.01813	Up	NULL	NULL
peak2064	chr17	78295228		78295451	2	NM_0011	ENDOV	93627	NM_0001	SGSH	101140	UTR3	RNF213(N	26.27185	Up	luks_peak	NULL
peak2065	chr17	78295664		78295815	1	NM_0011	ENDOV	93227	NM_0001	SGSH	101540	intronic	RNF213	9.19274	Up	NULL	NULL
peak2066	chr17	78296196		78296544	1	NM_0011	ENDOV	92597	NM_0001	SGSH	102171	intronic	RNF213	7.7636	Up	luks_peak	NULL
peak2067	chr17	78296912		78297231	1	NM_0011	ENDOV	91895	NM_0001	SGSH	102872	intronic	RNF213	6.65687	Up	luks_peak	NULL
peak2068	chr17	78298522		78298672	1	NM_0011	ENDOV	90370	NM_0001	SGSH	104398	intronic	RNF213	25.78241	Up	NULL	NULL
peak2069	chr17	78300580		78300738	1	NM_0011	ENDOV	88308	NM_0001	SGSH	106460	intronic	RNF213	5.57336	Up	NULL	NULL
peak2070	chr17	78302044		78302562	1	NM_0011	ENDOV	86664	NM_0001	SGSH	108104	exonic	RNF213	21.18535	Up	luks_peak	NULL
peak2071	chr17	78302637		78303042	1	NM_0011	ENDOV	86127	NM_0001	SGSH	108640	intronic	RNF213	9.59778	Up	luks_peak	NULL
peak2072	chr17	78303357		78303652	1	NM_0011	ENDOV	85462	NM_0001	SGSH	109305	intronic	RNF213	6.64841	Up	luks_peak	NULL
peak2073	chr17	78304498		78305198	1	NM_0011	ENDOV	84119	NM_0001	SGSH	110649	intronic	RNF213	20.9269	Up	luks_peak	NULL
peak2074	chr17	78305628		78306032	1	NM_0011	ENDOV	83137	NM_0001	SGSH	111631	exonic	RNF213	8.89058	Up	luks_peak	NULL
peak2075	chr17	78306148		78306464	1	NM_0011	ENDOV	82661	NM_0001	SGSH	112107	exonic	RNF213	9.26245	Up	luks_peak	NULL
peak2076	chr17	78307585		78307738	1	NM_0011	ENDOV	81305	NM_0001	SGSH	113462	intronic	RNF213	3.96398	Up	NULL	NULL
peak2077	chr17	78307936		78308144	1	NM_0011	ENDOV	80927	NM_0001	SGSH	113841	exonic	RNF213	7.98115	Up	luks_peak	NULL
peak2078	chr17	78308383		78308631	1	NM_0011	ENDOV	80460	NM_0001	SGSH	114308	intronic	RNF213	19.33331	Up	luks_peak	NULL
peak2079	chr17	78309837		78310297	1	NM_0011	ENDOV	78900	NM_0001	SGSH	115868	exonic	RNF213	22.94789	Up	luks_peak	NULL
peak2080	chr17	78311516		78311951	1	NM_0011	ENDOV	77233	NM_0001	SGSH	117534	exonic	RNF213	19.12605	Up	luks_peak	NULL
peak2081	chr17	78312276		78312433	1	NM_0011	ENDOV	76612	NM_0001	SGSH	118155	intronic	RNF213	8.53424	Up	NULL	NULL
peak2082	chr17	78312853		78313075	1	NM_0011	ENDOV	76003	NM_0001	SGSH	118765	exonic	RNF213	7.99065	Up	NULL	NULL
peak2083	chr17	78313379		78313684	1	NM_0011	ENDOV	75435	NM_0001	SGSH	119332	exonic	RNF213	8.55558	Up	luks_peak	NULL
peak2084	chr17	78313809		78313988	1	NM_0011	ENDOV	75068	NM_0001	SGSH	119699	exonic	RNF213	10.58843	Up	luks_peak	NULL
peak2085	chr17	78315225		78315555	1	NM_0011	ENDOV	73577	NM_0001	SGSH	121191	intronic	RNF213	8.23204	Up	luks_peak	NULL
peak2086	chr17	78316657		78316956	1	NM_0011	ENDOV	72160	NM_0001	SGSH	122607	exonic	RNF213	13.58766	Up	luks_peak	NULL
peak2087	chr17	78317032		78317321	1	NM_0011	ENDOV	71790	NM_0001	SGSH	122977	exonic	RNF213	5.14948	Up	luks_peak	NULL
peak2088	chr17	78317728		78318271	1	NM_0011	ENDOV	70967	NM_0001	SGSH	123800	exonic	RNF213	9.72603	Up	luks_peak	NULL
peak2089	chr17	78318536		78318688	1	NM_0011	ENDOV	70355	NM_0001	SGSH	124413	exonic	RNF213	3.92679	Up	luks_peak	NULL
peak2090	chr17	78318907		78319116	1	NM_0011	ENDOV	69955	NM_0001	SGSH	124812	exonic	RNF213	6.24701	Up	luks_peak	NULL
peak2091	chr17	78319835		78320009	1	NM_0011	ENDOV	69045	NM_0001	SGSH	125723	exonic	RNF213	5.30037	Up	luks_peak	NULL
peak2092	chr17	78320092		78320615	1	NM_0011	ENDOV	68613	NM_0001	SGSH	126154	exonic	RNF213	8.13763	Up	luks_peak	NULL
peak2093	chr17	78320767		78321141	1	NM_0011	ENDOV	68013	NM_0001	SGSH	126755	exonic	RNF213	4.09903	Up	luks_peak	NULL
peak2094	chr17	78321706		78321991	1	NM_0011	ENDOV	67118	NM_0001	SGSH	127649	exonic	RNF213	6.25511	Up	luks_peak	NULL
peak2095	chr17	78322442		78322996	1	NM_0011	ENDOV	66248	NM_0001	SGSH	128520	intronic	RNF213	6.92305	Up	luks_peak	NULL
peak2096	chr17	78323191		78323527	1	NM_0011	ENDOV	65608	NM_0001	SGSH	129160	intronic	RNF213	7.72207	Up	luks_peak	NULL
peak2097	chr17	78324461		78324753	1	NM_0011	ENDOV	64360	NM_0001	SGSH	130408	intronic	RNF213	7.25276	Up	luks_peak	NULL
peak2098	chr17	78324944		78325292	1	NM_0011	ENDOV	63849	NM_0001	SGSH	130919	intronic	RNF213	5.76637	Up	luks_peak	NULL
peak2099	chr17	78326069		78326273	2	NM_0011	ENDOV	62796	NM_0001	SGSH	131972	ncRNA_e	LOC10029	20.27416	Up	NULL	NULL
peak2100	chr17	78326480		78326709	2	NM_0011	ENDOV	62372	NM_0001	SGSH	132395	ncRNA_e	LOC10029	20.39948	Up	luks_peak	NULL
peak2101	chr17	78326788		78327020	2	NM_0011	ENDOV	62063	NM_0001	SGSH	132705	exonic	RNF213	4.99207	Up	luks_peak	NULL
peak2102	chr17	78327252		78327499	2	NM_0011	ENDOV	61591	NM_0001	SGSH	133176	exonic	RNF213	18.97662	Up	NULL	NULL
peak2103	chr17	78327570		78328109	2	NM_0011	ENDOV	61127	NM_0001	SGSH	133640	exonic	RNF213	12.53958	Up	luks_peak	NULL
peak2104	chr17	78328202		78328566	2	NM_0011	ENDOV	60583	NM_0001	SGSH	134185	exonic	RNF213	19.21736	Up	luks_peak	NULL
peak2105	chr17	78329710		78329990	2	NM_0011	ENDOV	59117	NM_0001	SGSH	135651	ncRNA_in	LOC10029	19.00024	Up	luks_peak	NULL
peak2106	chr17	78331840		78332194	2	NM_0011	ENDOV	56950	NM_0001	SGSH	137818	exonic	RNF213	6.88041	Up	luks_peak	NULL
peak2107	chr17	78332417		78332585	2	NM_0011	ENDOV	56466	NM_0001	SGSH	138302	ncRNA_in	LOC10029	7.05173	Up	NULL	NULL
peak2108	chr17	78333700		78333955	2	NM_0011	ENDOV	55139	NM_0001	SGSH	139628	exonic	RNF213	13.83023	Up	luks_peak	NULL
peak2109	chr17	78334179		78334334	2	NM_0011	ENDOV	54710	NM_0001	SGSH	140057	ncRNA_in	LOC10029	4.55499	Up	NULL	NULL
peak2110	chr17	78334434		78334596	2	NM_0011	ENDOV	54452	NM_0001	SGSH	140316	ncRNA_in	LOC10029	10.19336	Up	NULL	NULL
peak2111	chr17	78335789		78335973	2	NM_0011	ENDOV	53086	NM_0001	SGSH	141682	ncRNA_in	LOC10029	27.74664	Up	luks_peak	NULL
peak2112	chr17	78336287		78336490	2	NM_0011	ENDOV	52578	NM_0001	SGSH	142189	ncRNA_in	LOC10029	34.32036	Up	luks_peak	NULL
peak2113	chr17	78336603		78336809	2	NM_0011	ENDOV	52261	NM_0001	SGSH	142507	ncRNA_in	LOC10029	6.173	Up	luks_peak	NULL
peak2114	chr17	78336973		78337192	2	NM_0011	ENDOV	51884	NM_0001	SGSH	142883	exonic	RNF213	6.21521	Up	luks_peak	NULL
peak2115	chr17	78338462		78338623	2	NM_0011	ENDOV	50424	NM_0001	SGSH	144343	ncRNA_in	LOC10029	6.4272	Up	luks_peak	NULL
peak2116	chr17	78338832		78339493	2	NM_0011	ENDOV	49804	NM_0001	SGSH	144963	ncRNA_in	LOC10029	19.66556	Up	luks_peak	NULL
peak2117	chr17	78342761		78343064	2	NM_0011	ENDOV	46054	NM_0001	SGSH	148713	ncRNA_in	LOC10029	6.47572	Up	NULL	NULL
peak2118	chr17	78343354		78343540	2	NM_0011	ENDOV	45520	NM_0001	SGSH	149248	exonic	RNF213	6.54426	Up	NULL	NULL
peak2119	chr17	78343891		78344041	2	NM_0011	ENDOV	45001	NM_0001	SGSH	149767	ncRNA_in	LOC10029	6.08706	Up	NULL	NULL
peak2120	chr17	78344252		78344585	2	NM_0011	ENDOV	44548	NM_0001	SGSH	150219	ncRNA_in	LOC10029	20.4353	Up	luks_peak	NULL
peak2121	chr17	78347409		78347579	2	NM_0011	ENDOV	41473	NM_0001	SGSH	153295	ncRNA_in	LOC10029	16.54492	Up	NULL	NULL
peak2122	chr17	78348331		78348669	2	NM_0011	ENDOV	40467	NM_0001	SGSH	154301	exonic	RNF213	21.71022	Up	luks_peak	NULL
peak2123	chr17	78348942		78349112	2	NM_0011	ENDOV	39940	NM_0001	SGSH	154828	ncRNA_in	LOC10029	12.17981	Up	NULL	NULL
peak2124	chr17	78350158		78350459	2	NM_0011	ENDOV	38658	NM_0001	SGSH	156109	exonic	RNF213	22.2672	Up	luks_peak	NULL
peak2125	chr17	78350550		78350718	2	NM_0011	ENDOV	38333	NM_0001	SGSH	156435	exonic	RNF213	25.25359	Up	NULL	NULL
peak2126	chr17	78352063		78352233	2	NM_0011	ENDOV	36819	NM_0001	SGSH	157949	ncRNA_in	LOC10029	17.29513	Up	NULL	NULL
peak2127	chr17	78352396		78352605	2	NM_0011	ENDOV	36466	NM_0001	SGSH	158301	ncRNA_in	LOC10029	10.70049	Up	luks_peak	NULL
peak2128	chr17	78352717		78352994	2	NM_0011	ENDOV	36111	NM_0001	SGSH	158656	ncRNA_in	LOC10029	6.78163	Up	luks_peak	NULL
peak2129	chr17	78353374		78353544	2	NM_0011	ENDOV	35508	NM_0001	SGSH	159260	exonic	RNF213	11.54224	Up	NULL	NULL
peak2130	chr17	78353945		78354173	2	NM_0011	ENDOV	34908	NM_0001	SGSH	159860	ncRNA_in	LOC10029	12.87078	Up	luks_peak	NULL
peak2131	chr17	78354254		78354484	2	NM_0011	ENDOV	34598	NM_0001	SGSH	160170	ncRNA_in	LOC10029	8.79752	Up	luks_peak	NULL
peak2132	chr17	78355052		78355311	2	NM_0011	ENDOV	33785	NM_0001	SGSH	160982	ncRNA_in	LOC10029	14.21231	Up	luks_peak	NULL
peak2133	chr17	78356829		78356983	2	NM_0011	ENDOV	32061	NM_0001	SGSH	162707	exonic	RNF213	6.32889	Up	NULL	NULL
peak2134	chr17	78358417		78358578	2	NM_0011	ENDOV	30469	NM_0001	SGSH	164298	ncRNA_in	LOC10029	3.98836	Up	NULL	NULL
peak2135	chr17	78359367		78359571	2	NM_0011	ENDOV	29498	NM_0001	SGSH	165270	exonic	RNF213	7.78587	Up	NULL	NULL

peak2136	chr17	78359647	78359812	2	NM_0011	ENDOV	29237	NM_0001	SGSH	165530	ncRNA_in	LOC1002	17.77514	Up	NULL	NULL
peak2137	chr17	78360648	78360974	2	NM_0011	ENDOV	28156	NM_0001	SGSH	166612	exonic	RNF213	11.19643	Up	luks_peak	NULL
peak2138	chr17	78361326	78361561	2	NM_0011	ENDOV	27523	NM_0001	SGSH	167244	ncRNA_in	LOC1002	4.29069	Up	luks_peak	NULL
peak2139	chr17	78363610	78363832	2	NM_0011	ENDOV	25246	NM_0001	SGSH	169522	exonic	RNF213	7.35738	Up	luks_peak	NULL
peak2140	chr17	78363949	78364322	2	NM_0011	ENDOV	24831	NM_0001	SGSH	169936	exonic	RNF213	13.41102	Up	luks_peak	NULL
peak2141	chr17	78365056	78365281	2	NM_0011	ENDOV	23798	NM_0001	SGSH	170969	ncRNA_in	LOC1002	9.54188	Up	NULL	NULL
peak2142	chr17	78366222	78366392	2	NM_0011	ENDOV	22660	NM_0001	SGSH	172108	ncRNA_in	LOC1002	8.92012	Up	NULL	NULL
peak2143	chr17	78367380	78367538	2	NM_0011	ENDOV	21508	NM_0001	SGSH	173260	ncRNA_in	LOC1002	16.59378	Up	NULL	NULL
peak2144	chr17	78370808	78371079	2	NM_0011	ENDOV	18023	NM_0001	SGSH	176744	ncRNA_in	LOC1002	8.65802	Up	NULL	NULL
peak2145	chr17	78524138	78524306	2	NM_0245	CHMP6	441419	NM_0025	NPTX1	73818	intronic	RPTOR	5.02322	Down	NULL	PBS_peak_4670
peak2146	chr17	78538786	78538971	2	NM_0245	CHMP6	426762	NM_0025	NPTX1	88474	intronic	RPTOR	4.19283	Down	NULL	PBS_peak_4672
peak2147	chr17	79581762	79581929	1	NM_0012	TSPAN10	22351	NR_1301	NPLOC4	37386	intronic	NPLOC4	4.07827	Up	luks_peak	NULL
peak2148	chr17	80485152	80485391	1	NM_0246	FN3KRP	189310	NR_0365	C17orf62	76564	intronic	FOXK2	4.61119	Down	NULL	PBS_peak_4696
peak2149	chr17	80487971	80488237	1	NM_0246	FN3KRP	186478	NR_0365	C17orf62	79397	intronic	FOXK2	14.06969	Down	NULL	PBS_peak_4699
peak2150	chr17	80490500	80490748	1	NM_0246	FN3KRP	183958	NR_0365	C17orf62	81917	intronic	FOXK2	7.09493	Down	NULL	PBS_peak_4703
peak2151	chr17	80491537	80491843	1	NM_0246	FN3KRP	182892	NR_0365	C17orf62	82983	intronic	FOXK2	4.26567	Down	NULL	PBS_peak_4704
peak2152	chr17	80495311	80495596	1	NM_0246	FN3KRP	179128	NR_0365	C17orf62	86746	intronic	FOXK2	6.01172	Down	NULL	PBS_peak_4707
peak2153	chr17	80496384	80496620	1	NM_0246	FN3KRP	178080	NR_0365	C17orf62	87795	intronic	FOXK2	7.50889	Down	NULL	PBS_peak_4708
peak2154	chr17	81046909	81047079	1	NR_0294	RPL23AP	127672	NR_135465		37308	intronic	METRNL	24.39846	Up	NULL	NULL
peak2155	chr18	708595	708755	5	NM_0010	ADCYAP	196269	NM_0010	TYMSOS	50335	intronic	ENOSF1	11.72495	Down	NULL	NULL
peak2156	chr18	1058621	1058775	0	NM_0061	NDC80	1512812	NM_0054	YES1	246371	intergenic	LINC0190	4.25685	Down	NULL	NULL
peak2157	chr18	2895092	2895290	1	NM_0013	MYL12A	352289	NR_0337	CBX3P2	239797	intronic	EMILIN2	3.82723	Up	luks_peak	NULL
peak2158	chr18	3252796	3252981	4	NM_0335	MYL12B	9222	NM_0198	MYOM1	32782	intronic	MYL12A	3.97764	Down	NULL	PBS_peak_4726
peak2159	chr18	8023080	8023232	2	NM_0010	RAB12	586287	NM_0055	LAMA1	905343	intronic	PTPRM	9.98016	Down	NULL	NULL
peak2160	chr18	18683583	18683744	1	NM_0011	GREB1L	138539	NR_0274	LOC6446	3357745	intronic	ROCK1	4.72562	Down	NULL	NULL
peak2161	chr18	19199323	19199645	2	NR_0315	MIR320C	63987	NM_0529	ESCO1	18791	intronic	SNRPD1	4.53821	Down	NULL	PBS_peak_4763
peak2162	chr18	19200757	19201096	2	NR_0315	MIR320C	62544	NM_0529	ESCO1	20233	intronic	SNRPD1	8.05625	Down	NULL	PBS_peak_4765
peak2163	chr18	19204451	19204634	2	NR_0315	MIR320C	58928	NM_0529	ESCO1	23849	intronic	SNRPD1	8.92952	Down	NULL	PBS_peak_4766
peak2164	chr18	19205349	19205515	2	NR_0315	MIR320C	58039	NM_0529	ESCO1	24739	intronic	SNRPD1	3.491	Down	NULL	NULL
peak2165	chr18	36155010	36155172	0	NR_0498	MIR5583-	1101594	NM_0201	CELF4	1009091	intergenic	MIR4318	19.09435	Down	NULL	NULL
peak2166	chr18	43672401	43672554	5	NM_1384	HAUS1	11820	NM_0244	PSTPIP2	20227	intronic	ATP5A1	18.41619	Down	NULL	NULL
peak2167	chr18	52828737	52828905	0	NR_132985		290943	NR_1107	LOC1019	37284	intergenic	LINC0192	20.09473	Down	NULL	PBS_peak_4799
peak2168	chr18	60199424	60199583	2	NM_1944	PHLPP1	183168	NM_1767	PIGN	345214	intronic	ZCCHC2	4.1401	Up	luks_peak	NULL
peak2169	chr18	60204108	60204317	2	NM_1944	PHLPP1	178459	NM_1767	PIGN	349923	intronic	ZCCHC2	4.6647	Up	NULL	NULL
peak2170	chr18	60213697	60213957	2	NM_1944	PHLPP1	168845	NM_1767	PIGN	359538	intronic	ZCCHC2	23.97451	Up	luks_peak	NULL
peak2171	chr18	60216972	60217142	2	NM_1944	PHLPP1	165615	NM_1767	PIGN	362768	intronic	ZCCHC2	22.36295	Up	NULL	NULL
peak2172	chr18	60217992	60218173	2	NM_1944	PHLPP1	164589	NM_1767	PIGN	363793	intronic	ZCCHC2	24.60577	Up	NULL	NULL
peak2173	chr18	60219076	60219373	2	NM_1944	PHLPP1	163447	NM_1767	PIGN	364935	intronic	ZCCHC2	6.93145	Up	NULL	NULL
peak2174	chr18	60219467	60219632	2	NM_1944	PHLPP1	163122	NM_1767	PIGN	365260	intronic	ZCCHC2	10.02112	Up	NULL	NULL
peak2175	chr18	64571858	64572038	0	NR_0498	MIR5011	176873	NR_0731	CDH19	300573	intergenic	CDH19(di	13.67488	Down	NULL	NULL
peak2176	chr18	64744326	64744510	0	NR_0498	MIR5011	4403	NR_0731	CDH19	473043	intergenic	CDH19(di	5.95565	Down	NULL	PBS_peak_4813
peak2177	chr18	70478336	70478505	2	NR_134648		57202	NM_1825	CBLN2	266697	intronic	NETO1	5.59812	Down	NULL	NULL
peak2178	chr18	74111680	74111835	1	NM_0012	C18orf65	95719	NR_134646		687399	intronic	ZNF516	4.43041	Down	NULL	PBS_peak_4844
peak2179	chr18	74139218	74139383	1	NM_0012	C18orf65	68176	NR_134646		714942	intronic	ZNF516	6.8578	Down	NULL	PBS_peak_4865
peak2180	chr18	74140954	74141129	1	NM_0012	C18orf65	66435	NR_134646		716683	intronic	ZNF516	6.30702	Down	NULL	PBS_peak_4866
peak2181	chr18	74142163	74142315	1	NM_0012	C18orf65	65238	NR_134646		717881	intronic	ZNF516	16.10329	Down	NULL	PBS_peak_4867
peak2182	chr18	74145328	74145547	1	NM_0012	C18orf65	62039	NR_134646		721079	intronic	ZNF516	5.69356	Down	NULL	PBS_peak_4870
peak2183	chr18	76058917	76059083	0	NM_1719	SALL3	681275	NR_1041	LINC0102	353317	intergenic	LINC0102	4.17926	Down	NULL	PBS_peak_4889
peak2184	chr18	76673764	76673922	0	NM_1719	SALL3	66432	NR_1041	LINC0102	968160	intergenic	LINC0102	4.16076	Down	NULL	NULL
peak2185	chr18	77741363	77741711	8	NM_0011	RBFA	52809	NM_0250	PQLC1	29884	intronic	TXNL4A	6.85658	Down	NULL	PBS_peak_4898
peak2186	chr18	77744279	77744487	8	NM_0011	RBFA	49963	NM_0250	PQLC1	32730	intronic	TXNL4A	3.80597	Down	NULL	PBS_peak_4900
peak2188	chr19	575113	575284	4	NM_0011	HCN2	14694	NM_1825	ODF3L2	100215	intronic	BSG	5.44648	Down	NULL	PBS_peak_4904
peak2189	chr19	652113	652269	1	NM_0058	FSTL3	24198	NM_0050	POLRMT	18623	exonic	RNF126	5.05013	Down	NULL	PBS_peak_4907
peak2190	chr19	659959	660317	1	NM_0058	FSTL3	16251	NM_0050	POLRMT	26570	intronic	RNF126	8.83685	Down	NULL	PBS_peak_4914
peak2191	chr19	1412276	1412557	2	NM_0010	RPS15	25946	NM_1389	GAMT	10847	intronic	DAZAP1	4.31346	Down	NULL	PBS_peak_4934
peak2192	chr19	1419096	1419394	2	NM_0010	RPS15	19118	NM_1389	GAMT	17676	intronic	DAZAP1	3.91546	Down	NULL	PBS_peak_4939
peak2193	chr19	2175644	2175811	1	NM_0071	SF3A2	61088	NM_0039	AP3D1	24171	intronic	DOT1L	5.17857	Down	NULL	PBS_peak_4986
peak2194	chr19	2240960	2241119	1	NM_0004	AMH	8073	NM_0180	PLEKHJ1	4687	intronic	SF3A2	4.54235	Down	NULL	PBS_peak_4995
peak2195	chr19	2242569	2242736	1	NM_0004	AMH	6460	NM_0180	PLEKHJ1	6300	intronic	SF3A2	4.92426	Down	NULL	PBS_peak_4996
peak2196	chr19	2453966	2454125	1	NM_0156	GADD45E	22077	NR_1069	MIR7108	19047	intronic	LMNB2	5.9307	Down	NULL	PBS_peak_4998
peak2197	chr19	2772123	2772274	1	NM_0032	THOP1	13259	NM_2135	SLC39A3	32124	intronic	SGTA	12.79898	Down	NULL	NULL
peak2198	chr19	2774160	2774327	1	NM_0032	THOP1	11214	NM_2135	SLC39A3	34169	intronic	SGTA	25.69329	Down	NULL	PBS_peak_5000
peak2199	chr19	3377405	3377591	5	NM_0162	FZR1	128797	NR_1106	LOC1009	222325	intronic	NFIC	4.9017	Down	NULL	PBS_peak_5008
peak2200	chr19	3382976	3383130	5	NM_0162	FZR1	123242	NR_1106	LOC1009	227880	intronic	NFIC	4.05809	Down	NULL	PBS_peak_5012
peak2201	chr19	3385817	3386007	5	NM_0162	FZR1	120383	NR_1106	LOC1009	230739	intronic	NFIC	9.74631	Down	NULL	PBS_peak_5014
peak2202	chr19	3391079	3391249	5	NM_0162	FZR1	115131	NR_1106	LOC1009	235991	intronic	NFIC	4.15461	Down	NULL	PBS_peak_5020
peak2203	chr19	3399954	3400115	5	NM_0162	FZR1	106260	NR_1106	LOC1009	244861	intronic	NFIC	5.07804	Down	NULL	PBS_peak_5026
peak2204	chr19	3402284	3402602	5	NM_0162	FZR1	103852	NR_1106	LOC1009	247270	intronic	NFIC	6.44293	Down	NULL	PBS_peak_5028
peak2205	chr19	3408053	3408216	5	NM_0162	FZR1	98160	NR_1106	LOC1009	252961	intronic	NFIC	3.44185	Down	NULL	PBS_peak_5031
peak2206	chr19	3412428	3412593	5	NM_0162	FZR1	93784	NR_1106	LOC1009	257337	intronic	NFIC	10.89851	Down	NULL	PBS_peak_5034
peak2207	chr19	4386624	4386859	3	NM_0054	CHAF1A	15918	NM_0177	STAP2	47894	intronic	SH3GL1	5.66163	Up	luks_peak	NULL
peak2208	chr19	5635575	5635943	7	NM_0012	HSD11B1	45017	NM_0146	SAFB2	12821	intronic	SAFB	4.20474	Down	NULL	PBS_peak_5068
peak2209	chr19	5636316	5636474	7	NM_0012	HSD11B1	44381	NM_0146	SAFB2	13457	intronic	SAFB	5.01591	Down	NULL	PBS_peak_5068
peak2210	chr19	5637690	5637840	7	NM_0012	HSD11B1	43011	NM_0146	SAFB2	14827	intronic	SAFB	3.72091	Down	NULL	PBS_peak_5069
peak2211	chr19	5643299	5643580	7	NM_0012	HSD11B1	37336	NM_0146	SAFB2	20501	intronic	SAFB	5.95835	Down	NULL	PBS_peak_5076
peak2212	chr19	5654038	5654202	7	NM_0012	HSD11B1	26656	NM_0146	SAFB2	31182	exonic	SAFB	4.35046	Down	NULL	NULL
peak2213	chr19	5957053	5957240	4	NR_0463	LOC1001	21267	NR_0341	NDUFA11	53122	intronic	RANBP3	5.80434	Down	NULL	PBS_peak_5084
peak2214	chr19	5972612	5972848	4	NR_0463	LOC1001	5684	NR_0341	NDUFA11	68706	intronic	RANBP3	19.41315	Down	NULL	PBS_peak_5094
peak2215	chr19	8058638	8058814	1	NM_0012	CCL25	58920	NM_0063	TIMM44	50018	intronic	ELAVL1	4.38932	Down	NULL	PBS_peak_5106
peak2216	chr19	8059484	8059725	1	NM_0012	CCL25	58041	NM_0063	TIMM44	50896	intronic	ELAVL1	5.28127	Down	NULL	PBS_peak_5107
peak2217	chr19	8062164	8062339	1	NM_0012	CCL25	55394	NM_0063	TIMM44	53543	intronic	ELAVL1	3.96115	Down	NULL	PBS_peak_5109
peak2218	chr19	8064230	8064403	1	NM_0012	CCL25	53329	NM_0063	TIMM44	55608	intronic	ELAVL1	5.05308	Down	NULL	PBS_peak_5111
peak2219	chr19	8064639	8064901	1	NM_0012	CCL25	52876	NM_0063	TIMM44	56062	intronic	ELAVL1	5.99412	Down	NULL	PBS_peak_5111
peak2220	chr19	8521592	8521780	3	NM_0010	OR2Z1	319630	NR_0382	RAB11B-	66111	UTR5	HNRNPM	6.1966	Down	NULL	PBS_peak_5123
peak2221	chr19	8522790	8523066	3	NM_0010	OR2Z1	318388	NR_0382	RAB11B-	67353	intronic	HNRNPM	5.08088	Down	NULL	PBS_peak_5125
peak2222	chr19	8530235	8530400	3	NM_0010	OR2Z1	310998	NR_0382	RAB11B-	74742	exonic	HNRNPM	4.65684	Down	NULL	PBS_peak_5130
peak2223	chr19	10294676	10294869	4	NR_0362	MIR4322	46316	NM_0037	EIF3G	64173	intronic	DNMT1	7.71304	Down	NULL	PBS_peak_5134
peak2224	chr19	10295510	10295759	4	NR_0362	MIR4322	45454	NM_0037	EIF3G	65035	intronic	DNMT1	6.41564	Down	NULL	PBS_peak_5135
peak2225	chr19	10298615	10298909	4	NR_0362	MIR4322	42327	NM_0037	EIF3G	68163	intronic	DNMT1	12.68759	Down	NULL	PBS_peak_5136
peak2226	chr19	10299428	10299634	4	NR_0362	MIR4322	41558	NM_0037	EIF3G	68932	intronic	DNMT1	4.47871	Down	NULL	PBS_peak_5137
peak2227	chr19	10300467	10300663	4	NR_0362	MIR4322	40524	NM_0037	EIF3G	69966	intronic	DNMT1	4.44841	Down	NULL	PBS_peak_5138
peak2228	chr19	10387984	10388186	1	NM_0010	ICAM4	9558	NM_0042	S1PR2	46137	intronic	ICAM1	5.03699	Up	luks_peak	NULL
peak2229	chr19	10389547	10389709	1	NM_0010	ICAM4	8015	NM_0042	S1PR2	47680	intronic	ICAM1	5.25546	Up	NULL	NULL
peak2230	chr19	10390649	10390962	1	NM_0010	ICAM4	6837	NM_0042	S1PR2	48857	intronic	ICAM1	6.28433	Up	luks_peak	NULL
peak2231	chr19	10391955	10392125	1	NM_0010	ICAM4	5603	NM_0042	S1PR2	50092	intronic	ICAM1	16.766	Up	NULL	NULL
peak2232	chr19	10392600	10392852	1	NM_0010	ICAM4	4917	NM_0042	S1PR2	50778	intronic	ICAM1	15.46716	Up	luks_peak	NULL
peak2233	chr19	10766999	10767198	5	NM_0312	QTRT1	45013	NR_0243	ILF3-AS1	2550	intronic	ILF3	6.1549	Down	NULL	PBS_peak_5140
peak2234	chr19	10769216	10769471	5	NM_0312	QTRT1	42768	NR_0243	ILF3-AS1	4795	intronic	ILF3	4.48916	Down	NULL	PBS_peak_5142
peak2235	chr19	10770184	10770889	5	NM_0312	QTRT1	41575	NR_0243	ILF3-AS1	5988	intronic	ILF3	8.58461	Down	NULL	PBS_peak_5143
peak2236	chr19	10771767	10772054	5	NM_0312	QTRT1	40201	NR_0243	ILF3-AS1	7362	intronic	ILF3	5.78395	Down	NULL	PBS_peak_5144
peak2237	chr19	10772577	10772819	5	NM_0312	QTRT1	39414	NR_0243	ILF3-AS1	8150	intronic	ILF3	9.38766	Down	NULL	PBS_peak_5145
peak2238	chr19	10778822	10779018	5	NM_0312	QTRT1	33192	NR_0243	ILF3-AS1	14372	intronic	ILF3	3.65516	Down	NULL	PBS_peak_5150
peak2239	chr19	10782476	10782740	5	NM_0312	QTRT1	29504	NR_0243	ILF3-AS1	18060	intronic	ILF3	6.98625	Down	NULL	PBS_peak_5152

peak2240	chr19	10832615	10832781	5	NR_0399(	MIR4748	58232	NR_0243	ILF3-AS1	68150	intronic	DNM2	4.40059	Down	NULL	PBS_peak_5160
peak2241	chr19	10836606	10836841	5	NR_0399(	MIR4748	54206	NR_0243	ILF3-AS1	72175	intronic	DNM2	4.2209	Down	NULL	PBS_peak_5165
peak2242	chr19	11079263	11079436	3	NM_0011	SMARCA	15478	NM_0240	YIPF2	39661	intronic	SMARCA	4.56446	Down	NULL	PBS_peak_5174
peak2243	chr19	11080872	11081030	3	NM_0011	SMARCA	13877	NM_0240	YIPF2	41263	intronic	SMARCA	5.26392	Down	NULL	PBS_peak_5176
peak2244	chr19	11083430	11083593	3	NM_0011	SMARCA	11316	NM_0240	YIPF2	43823	intronic	SMARCA	6.686	Down	NULL	PBS_peak_5177
peak2245	chr19	13239633	13239820	1	NM_0049	IER2	21555	NM_0011	TRMT1	12163	intronic	NACC1	16.76805	Down	NULL	PBS_peak_5184
peak2246	chr19	15411473	15411709	0	NR_0317	MIR1470	148768	NM_0142	BRD4	20329	intronic	BRD4	3.59594	Down	NULL	PBS_peak_5224
peak2247	chr19	16562728	16562899	6	NM_0012	C19orf44	44308	NM_0541	CIB3	278477	intronic	EPS15L1	5.27547	Down	NULL	PBS_peak_5249
peak2248	chr19	16704901	16705056	1	NM_0240	TMEM38	66959	NM_0248	SLC35E1	21785	intronic	MED26	4.68714	Up	NULL	NULL
peak2249	chr19	16707579	16707806	1	NM_0240	TMEM38	64245	NM_0248	SLC35E1	24499	intronic	MED26	5.0066	Down	NULL	PBS_peak_5262
peak2250	chr19	16724208	16724415	1	NM_0240	TMEM38	47626	NM_0248	SLC35E1	41118	intronic	MED26	4.29611	Down	NULL	PBS_peak_5278
peak2251	chr19	17212248	17212415	2	NM_0184	USE1	113823	NM_0334	HAUS8	25988	intronic	MYO9B	8.17604	Up	NULL	NULL
peak2252	chr19	17510758	17510924	0	NR_1307	BISPR	5654	NM_0313	PLVAP	22683	intergenic	PLVAP(di	5.49417	Up	NULL	NULL
peak2253	chr19	18536556	18536709	2	NM_0011	KXD1	131939	NM_1452	LRRC25	28217	intronic	SSBP4	4.80666	Down	NULL	NULL
peak2254	chr19	18820213	18820383	2	NM_0029	UPF1	122446	NM_0047	CRLF1	102638	intronic	CRTC1	8.06662	Down	NULL	PBS_peak_5317
peak2255	chr19	18947371	18947600	2	NM_0190	DDX49	82998	NM_0000	COMP	45371	intronic	UPF1	5.50152	Down	NULL	PBS_peak_5320
peak2256	chr19	18948112	18948293	2	NM_0190	DDX49	82281	NM_0000	COMP	46088	intronic	UPF1	5.10712	Down	NULL	PBS_peak_5320
peak2257	chr19	18954387	18954558	2	NM_0190	DDX49	76011	NM_0000	COMP	52358	intronic	UPF1	4.36229	Down	NULL	PBS_peak_5325
peak2258	chr19	19119337	19119504	2	NM_0011	ARMC6	24966	NM_0048	HOMER3	67379	intronic	SUGP2	7.28602	Down	NULL	NULL
peak2259	chr19	19121727	19121911	2	NM_0011	ARMC6	22568	NM_0048	HOMER3	69778	intronic	SUGP2	18.77565	Down	NULL	PBS_peak_5328
peak2260	chr19	19125068	19125263	2	NM_0011	ARMC6	19221	NM_0048	HOMER3	73124	intronic	SUGP2	17.08073	Down	NULL	PBS_peak_5329
peak2261	chr19	19132708	19133102	2	NM_0011	ARMC6	11482	NM_0048	HOMER3	80864	intronic	SUGP2	4.90647	Down	NULL	PBS_peak_5330
peak2262	chr19	19133848	19134046	2	NM_0011	ARMC6	10440	NM_0048	HOMER3	81906	intronic	SUGP2	5.55146	Down	NULL	PBS_peak_5331
peak2263	chr19	19521255	19521427	1	NR_0303	MIR640	24531	NM_1722	SUGP1	90020	intronic	GATAD2	3.86899	Down	NULL	PBS_peak_5338
peak2264	chr19	19543648	19543852	1	NR_0303	MIR640	2122	NM_1722	SUGP1	112429	intronic	GATAD2	6.08202	Down	NULL	PBS_peak_5358
peak2265	chr19	30609210	30609391	0	NM_0147	ZNF536	253999	NM_0012	C19orf12	402604	intergenic	URI1(dist	22.0129	Down	NULL	PBS_peak_5382
peak2266	chr19	32165514	32165666	0	NR_1106	LINC0153	351178	NM_0208	TSHZ3	325400	intergenic	THEG5(di	4.53716	Down	NULL	NULL
peak2267	chr19	32845476	32845790	2	NM_0011	DPY19L3	51022	NR_1106	LOC1019	248811	exonic	ZNF507	7.7782	Down	NULL	PBS_peak_5388
peak2268	chr19	33187793	33187954	1	NM_0011	TDRD12	22805	NM_0321	ANKRD2	21771	intronic	NUDT19	22.73066	Down	NULL	NULL
peak2269	chr19	33189047	33189478	1	NM_0011	TDRD12	21416	NM_0321	ANKRD2	23160	intronic	NUDT19	6.69402	Down	NULL	PBS_peak_5389
peak2270	chr19	34922639	34922821	1	NM_0010	WTIP	50150	NM_0011	PEPD	909931	exonic	UBA2	7.58096	Down	NULL	PBS_peak_5395
peak2271	chr19	41262672	41262867	1	NM_0012	MIA	18312	NM_1984	C19orf54	6941	intronic	SNRPA	6.73603	Down	NULL	PBS_peak_5403
peak2272	chr19	41264300	41264452	1	NM_0012	MIA	16706	NM_1984	C19orf54	8548	intronic	SNRPA	3.84473	Down	NULL	PBS_peak_5404
peak2273	chr19	41265329	41265486	1	NM_0012	MIA	15674	NM_1984	C19orf54	9579	exonic	SNRPA	23.50349	Down	NULL	PBS_peak_5405
peak2274	chr19	42371927	42372134	1	NR_1068	MIR6797	1666	NM_1735	LYPD4	23294	intronic	RPS19	5.31629	Down	NULL	PBS_peak_5410
peak2275	chr19	47637795	47638043	4	NR_0361	MIR3190	92280	NM_0151	ZC3H4	20910	intronic	SAE1	5.21348	Down	NULL	PBS_peak_5431
peak2276	chr19	47996931	47997148	4	NM_0157	GLTSCR1	114413	NR_1119	KPTN	9518	ncRNA_in	NAPA-AS	5.67984	Up	luks_peak	NULL
peak2277	chr19	48000124	48000422	4	NM_0157	GLTSCR1	111180	NR_1119	KPTN	12752	ncRNA_in	NAPA-AS	3.9457	Up	luks_peak	NULL
peak2278	chr19	48000566	48001040	4	NM_0157	GLTSCR1	110650	NR_1119	KPTN	13282	ncRNA_in	NAPA-AS	5.87963	Up	luks_peak	NULL
peak2279	chr19	48001279	48001482	4	NM_0157	GLTSCR1	110072	NR_1119	KPTN	13859	ncRNA_in	NAPA-AS	4.16436	Up	luks_peak	NULL
peak2280	chr19	48005165	48005423	3	NM_0157	GLTSCR1	106159	NR_1119	KPTN	17773	intronic	NAPA	4.26552	Up	luks_peak	NULL
peak2281	chr19	48005945	48006209	3	NM_0157	GLTSCR1	105376	NR_1119	KPTN	18556	intronic	NAPA	8.41981	Up	luks_peak	NULL
peak2282	chr19	48012377	48012536	3	NM_0157	GLTSCR1	98996	NR_1119	KPTN	24935	intronic	NAPA	4.58113	Up	luks_peak	NULL
peak2283	chr19	49598812	49599007	2	NM_0013	LIN7B	18708	NM_0318	KCNA7	22711	intronic	SNRNP70	6.59281	Down	NULL	PBS_peak_5448
peak2284	chr19	55526894	55527048	3	NM_1331	EPS8L1	60250	NM_2068	NLRP7	68098	exonic	GP6	5.95077	Up	NULL	NULL
peak2285	chr19	55747358	55747510	1	NM_0324	BRSK1	48100	NR_1068	MIR6804	5114	intronic	PPP6R1	15.54342	Down	NULL	PBS_peak_5456
peak2286	chr19	55900804	55900958	3	NM_0331	ZNF628	86818	NM_0011	TMEM23	5254	UTR3	RPL28(NI	11.23167	Down	NULL	PBS_peak_5466
peak2287	chr19	56602629	56602807	1	NM_0012	ZNF444	49817	NM_1768	NLRP13	159016	intronic	ZNF787	6.21469	Down	NULL	PBS_peak_5478
peak2288	chr19	58902251	58902436	1	NM_0011	RNF225	5113	NR_0399	MIR4754	4118	intronic	RPS5	4.98963	Down	NULL	PBS_peak_5485
peak389	chr2	2972403	2972554	1	NM_0160	TRAPPC1	410967	NM_0150	MYT1L	637393	ncRNA_in	LINC012	5.08528	Down	NULL	PBS_peak_5503
peak390	chr2	3243360	3243544	1	NM_0160	TRAPPC1	139994	NR_1102	LINC012	113654	intronic	EIPR1	5.49128	Down	NULL	PBS_peak_5506
peak391	chr2	6822734	6822902	0	NM_0806	RSAD2	194978	NR_1104	MIR7515	32167	intergenic	MIR7515	19.84612	Down	NULL	NULL
peak392	chr2	7021330	7021501	1	NM_0147	RNF144A	36107	NR_0462	CMPK2	14649	intronic	RSAD2	4.75895	Up	NULL	NULL
peak393	chr2	7021847	7022077	1	NM_0147	RNF144A	35561	NR_0462	CMPK2	15196	intronic	RSAD2	14.8177	Up	luks_peak	NULL
peak394	chr2	9684965	9685346	1	NM_0056	TAF1B	298415	NR_134957		121457	intronic	ADAM17	4.64943	Down	NULL	PBS_peak_5521
peak395	chr2	9686876	9687048	1	NM_0056	TAF1B	296609	NR_134957		123264	intronic	ADAM17	5.973	Down	NULL	PBS_peak_5523
peak396	chr2	9687825	9688403	1	NM_0056	TAF1B	295457	NR_134957		124416	intronic	ADAM17	9.61464	Down	NULL	PBS_peak_5524
peak397	chr2	10468367	10468571	2	NM_0012	HPCAL1	1820	NR_0362	MIR4261	135672	intronic	HPCAL1	3.51152	Up	NULL	NULL
peak398	chr2	10511719	10511888	5	NR_1105	LOC1019	78050	NR_0362	MIR4261	179006	intronic	HPCAL1	4.32453	Up	luks_peak	NULL
peak399	chr2	11600794	11600973	7	NM_0146	GREB1	73358	NM_0048	ROCK2	116172	intronic	E2F6	6.21757	Down	NULL	PBS_peak_5533
peak400	chr2	15976862	15977016	0	NR_1257	MYCNUT	83582	NR_0520	NBAS	275467	intergenic	LINC018	4.09239	Down	NULL	NULL
peak401	chr2	17162997	17163156	0	NM_0033	VSNL1	558730	NM_0307	FAM49A	315942	intergenic	FAM49A(	11.0826	Down	NULL	PBS_peak_5541
peak402	chr2	17628527	17628710	0	NM_0033	VSNL1	93188	NM_0307	FAM49A	781484	intergenic	FAM49A(	4.92594	Down	NULL	PBS_peak_5547
peak403	chr2	20541694	20541853	2	NM_0040	RHOB	105058	NM_0012	PUM2	14626	intronic	PUM2	4.37658	Down	NULL	NULL
peak404	chr2	24135113	24135307	3	NM_1817	UBXN2A	28166	NR_0388	LOC6459	2201686	intronic	ATAD2B	4.90927	Down	NULL	PBS_peak_5567
peak405	chr2	26085503	26085662	1	NM_0161	RAB10	171146	NM_001320937		189066	intronic	ASXL2	3.2908	Down	NULL	PBS_peak_5569
peak406	chr2	26260139	26260314	1	NM_0011	GAREML	135733	NM_0022	KIF3C	54783	intronic	RAB10	4.12203	Down	NULL	PBS_peak_5570
peak407	chr2	26285630	26285792	1	NM_0011	GAREML	110249	NM_0022	KIF3C	80268	intronic	RAB10	21.88024	Down	NULL	NULL
peak408	chr2	27576399	27576583	3	NM_0012	SNX17	16872	NM_0024	MPV17	30522	intronic	GTF3C2	5.34841	Down	NULL	PBS_peak_5588
peak409	chr2	27614072	27614242	1	NM_0133	NRBP1	37316	NM_1446	ZNF513	10546	intronic	PPM1G	6.59711	Down	NULL	PBS_peak_5591
peak410	chr2	27614725	27614923	1	NM_0133	NRBP1	36649	NM_1446	ZNF513	11213	intronic	PPM1G	5.42686	Down	NULL	PBS_peak_5592
peak411	chr2	27616210	27616444	2	NM_0133	NRBP1	35146	NM_1446	ZNF513	12716	ncRNA_e	FTH1P3	21.62389	Down	NULL	PBS_peak_5594
peak412	chr2	27618415	27618661	1	NM_0133	NRBP1	32935	NR_0022	FTH1P3	2095	intronic	PPM1G	17.62613	Down	NULL	PBS_peak_5595
peak413	chr2	27619405	27619721	1	NM_0133	NRBP1	31910	NR_0022	FTH1P3	3120	intronic	PPM1G	7.66035	Down	NULL	PBS_peak_5596
peak414	chr2	27622277	27622449	1	NM_0133	NRBP1	29110	NR_0022	FTH1P3	5920	intronic	PPM1G	6.26726	Down	NULL	PBS_peak_5599
peak415	chr2	27624471	27624640	1	NM_0133	NRBP1	26917	NR_0022	FTH1P3	8112	intronic	PPM1G	5.60016	Down	NULL	PBS_peak_5602
peak416	chr2	27625387	27625553	1	NM_0133	NRBP1	26003	NR_0022	FTH1P3	9027	intronic	PPM1G	6.71023	Down	NULL	PBS_peak_5603
peak417	chr2	27628383	27628665	1	NM_0133	NRBP1	22949	NR_0022	FTH1P3	12081	intronic	PPM1G	6.62883	Down	NULL	PBS_peak_5606
peak418	chr2	27630015	27630180	1	NM_0133	NRBP1	21375	NR_0022	FTH1P3	13654	intronic	PPM1G	7.31383	Down	NULL	PBS_peak_5607
peak419	chr2	27630259	27630429	1	NM_0133	NRBP1	21129	NR_0022	FTH1P3	13901	intronic	PPM1G	25.65525	Down	NULL	PBS_peak_5607
peak420	chr2	28637934	28638109	0	NM_0011	PLB1	80916	NR_1038	FLJ31356	20482	downstrea	FOSL2	6.46126	Up	NULL	NULL
peak421	chr2	29121657	29121922	1	NR_0030	SNORD92	14738	NM_0179	TRMT61E	28614	intronic	WDR43	5.13249	Down	NULL	PBS_peak_5612
peak422	chr2	29122666	29122867	1	NR_0030	SNORD92	13761	NM_0179	TRMT61E	29591	intronic	WDR43	13.00292	Down	NULL	PBS_peak_5614
peak423	chr2	29136184	29136363	1	NR_0030	SNORD92	254	NM_0179	TRMT61E	43098	intronic	WDR43	10.19309	Down	NULL	NULL
peak424	chr2	30119082	30119247	1	NM_0011	YPEL5	250585	NM_0010	C2orf71	822037	intronic	ALK	4.28575	Down	NULL	NULL
peak425	chr2	37343035	37343292	3	NR_0378	CEBPZOS	80471	NM_0190	HEATR5E	31675	intronic	EIF2AK2	7.85295	Up	NULL	NULL
peak426	chr2	37344427	37344686	3	NR_0378	CEBPZOS	79078	NM_0190	HEATR5E	33068	intronic	EIF2AK2	12.75239	Up	NULL	NULL
peak427	chr2	37345175	37345343	3	NR_0378	CEBPZOS	78376	NM_0190	HEATR5E	33771	intronic	EIF2AK2	19.62088	Up	NULL	NULL
peak428	chr2	37350692	37350849	3	NR_0378	CEBPZOS	72864	NM_0190	HEATR5E	39282	intronic	EIF2AK2	20.30874	Up	NULL	NULL
peak429	chr2	37356142	37356375	3	NR_0378	CEBPZOS	67376	NM_0190	HEATR5E	44770	intronic	EIF2AK2	6.3597	Up	luks_peak	NULL
peak430	chr2	37358863	37359035	3	NR_0378	CEBPZOS	64686	NM_0190	HEATR5E	47461	intronic	EIF2AK2	7.56159	Up	luks_peak	NULL
peak431	chr2	37367974	37368211	3	NR_0378	CEBPZOS	55542	NM_0190	HEATR5E	56604	intronic	EIF2AK2	11.6978	Up	NULL	NULL
peak432	chr2	37374935	37375106	3	NR_0378	CEBPZOS	48614	NM_0190	HEATR5E	63532	exonic	EIF2AK2	8.81987	Up	luks_peak	NULL
peak433	chr2	44215785	44215963	1	NM_0010	PPM1B	180068	NM_0224	ABCG5	149916	intronic	LRPPRC	4.2375	Down	NULL	PBS_peak_5654
peak434	chr2	44216897	44217238	1	NM_0010	PPM1B	178874	NM_0224	ABCG5	151109	intronic	LRPPRC	6.45171	Down	NULL	PBS_peak_5656
peak435	chr2	44219475	44219664	1	NM_0010	PPM1B	176372	NM_0224	ABCG5	153611	intronic	LRPPRC	8.99067	Down	NULL	PBS_peak_5659
peak436	chr2	44219814	44220185	1	NM_0010	PPM1B	175942	NM_0224	ABCG5	154041	intronic	LRPPRC	8.37179	Down	NULL	PBS_peak_5659
peak437	chr2	49503370	49503584	0	NR_135237		1756262	NM_1814	FSHR	121811	intergenic	FSHR(dist	16.65231	Down	NULL	PBS_peak_5665
peak438	chr2	55229221	55229410	5	NM_0011	RPS27A	229723	NM_0010	TSPYL6	745906	intronic	RTN4	6.26922	Down	NULL	PBS_peak_5672
peak439	chr2	55903701	55903862	1	NM_0010	CCDC85A	507476	NM_0204	PPP4R3B	58921	intronic	PNPT1	4.9493	Up	NULL	NULL
peak440	chr2	55904335	55904486	1	NM_0010	CCDC85A	506847	NM_0204	PPP4R3B	59550	intronic	PNPT1	3.64213	Up	NULL	NULL
peak441	chr2	55915265	55915435	1	NM_0010	CCDC85A	495908	NM_0204	PPP4R3B	70490	intronic	PNPT1	5.40244	Up	NULL	NULL
peak442	chr2	61756378	61756549	1	NM_1525	COMMD1	376339	NM_0147	USP34	58614	intronic	XPO1	17.15772	Down	NULL	PBS_peak_5684

peak443	chr2	61761363	61761620	1	NM_1525	COMMD1	371311	NM_0147	USP34	63642	intronic	XPO1	19.70668	Down	NULL	PBS_peak_5686
peak444	chr2	62435940	62436320	1	NM_001319075		6526	NM_0064	CCT4	320324	intronic	B3GNT2	4.59686	Down	NULL	PBS_peak_5695
peak445	chr2	62437506	62437680	1	NM_001319075		5063	NM_0064	CCT4	321787	intronic	B3GNT2	5.36907	Down	NULL	PBS_peak_5695
peak446	chr2	65353048	65353222	2	NM_0010	ACTR2	101694	NR_03658	LOC4009	193554	intronic	RAB1A	3.60791	Down	NULL	PBS_peak_5717
peak447	chr2	65649483	65649636	1	NR_13093	LOC72934	960878	NM_0011	SPRED2	55628	intronic	SPRED2	16.04932	Down	NULL	PBS_peak_5734
peak448	chr2	68120803	68120989	0	NM_0201	PNO1	264109	NR_11027	LOC10192	68202	intergenic	LINC0181	6.87403	Down	NULL	PBS_peak_5750
peak449	chr2	68433416	68433566	1	NM_0026	PLEK	158831	NM_0012	WDR92	48799	intronic	PPP3R1	5.06894	Down	NULL	NULL
peak450	chr2	68603307	68603487	1	NM_1735	APLF	91294	NM_0154	CNRIP1	56214	intronic	PLEK	6.4134	Up	luks_peak	NULL
peak451	chr2	74233479	74233672	1	NR_04563	BOLA3-A	141532	NR_10403	DGUOK-A	25009	intronic	TET3	3.53392	Down	NULL	PBS_peak_5777
peak452	chr2	74244838	74245021	1	NR_04563	BOLA3-A	130178	NR_10403	DGUOK-A	36363	intronic	TET3	12.31351	Down	NULL	PBS_peak_5781
peak453	chr2	74433603	74433753	2	NR_02446	DCTN1-A	179167	NM_0182	MOB1A	27584	intronic	MTHFD2	4.01906	Down	NULL	PBS_peak_5794
peak454	chr2	96867858	96868096	1	NR_04632	STARD7-	6177	NM_0044	DUSP2	56798	intronic	STARD7	8.20855	Down	NULL	PBS_peak_5818
peak455	chr2	96868318	96868654	1	NR_04632	STARD7-	5668	NM_0044	DUSP2	57307	intronic	STARD7	7.6	Down	NULL	PBS_peak_5819
peak456	chr2	96869576	96869750	1	NR_04632	STARD7-	4491	NM_0044	DUSP2	58484	intronic	STARD7	4.65463	Down	NULL	PBS_peak_5820
peak457	chr2	96870099	96870269	1	NR_04632	STARD7-	3970	NM_0044	DUSP2	59005	intronic	STARD7	9.22792	Down	NULL	PBS_peak_5820
peak458	chr2	96870679	96870838	1	NR_04632	STARD7-	3395	NM_0044	DUSP2	59579	intronic	STARD7	6.40369	Down	NULL	PBS_peak_5821
peak459	chr2	96871214	96871378	1	NR_04632	STARD7-	2858	NM_0044	DUSP2	60117	intronic	STARD7	5.04733	Down	NULL	PBS_peak_5822
peak460	chr2	97213049	97213298	7	NM_0012	FER1L5	95300	NR_10434	NEURL3	39327	exonic	ARID5A	6.09958	Up	luks_peak	NULL
peak461	chr2	100093053	100093219	2	NR_10373	LINC0110	731580	NM_0057	TXNDC9	140276	intronic	REV1	6.63395	Down	NULL	PBS_peak_5827
peak462	chr2	100096004	100096315	2	NR_10373	LINC0110	728556	NM_0057	TXNDC9	143299	intronic	REV1	12.58576	Down	NULL	PBS_peak_5829
peak463	chr2	105261130	105261297	0	NM_0062	POU3F3	209312	NR_11011	LINC0110	134246	intergenic	LINC0110	24.32155	Down	NULL	NULL
peak464	chr2	105575694	105575847	1	NM_1826	MRPS9	78712	NR_11037	LINC0115	86717	ncRNA_in	LOC1027	4.13074	Down	NULL	PBS_peak_5835
peak465	chr2	114686833	114687000	3	NR_03412	LINC0119	50229	NR_11017	LOC1010	38347	intronic	ACTR3	4.00659	Up	NULL	NULL
peak466	chr2	119433251	119433405	0	NM_0067	MARCO	266417	NR_11027	LOC10192	72786	intergenic	LOC10192	6.24805	Down	NULL	PBS_peak_5868
peak467	chr2	122833597	122833772	0	NM_1307	CNTNAP5	1949179	NM_0323	NIFK	339181	intergenic	TSN(dist=	16.05068	Down	NULL	PBS_peak_5870
peak468	chr2	126332224	126332399	0	NM_0012	GYPC	1081199	NM_0323	NIFK	3837808	intergenic	CNTNAP5	14.57294	Down	NULL	NULL
peak469	chr2	128550847	128551014	3	NM_0201	UGGT1	297823	NM_0011	LIMS2	111570	intronic	WDR33	4.22001	Down	NULL	PBS_peak_5884
peak470	chr2	128560943	128561164	3	NM_0201	UGGT1	287700	NM_0011	LIMS2	121693	intronic	WDR33	5.00549	Down	NULL	PBS_peak_5888
peak471	chr2	129681522	129681675	0	NR_11028	LOC10192	999151	NM_0048	HS6ST1	605427	intergenic	LOC10192	12.3256	Down	NULL	NULL
peak472	chr2	131867094	131867270	13	NM_0010	POTEE	108742	NM_0010	FAM168B	16178	intronic	PLEKHB2	7.6464	Down	NULL	PBS_peak_5897
peak473	chr2	161999801	161999965	2	NM_0011	TANK	17056	NM_0168	RBMS1	649565	intronic	TANK	4.8208	Up	NULL	NULL
peak474	chr2	162003832	162004006	2	NM_0011	TANK	13020	NM_0168	RBMS1	653601	intronic	TANK	6.4218	Up	NULL	NULL
peak475	chr2	163139031	163139225	1	NM_0121	GCA	61455	NM_0044	FAP	39083	exonic	IFIH1	12.86811	Up	NULL	NULL
peak476	chr2	163155288	163155771	1	NM_0121	GCA	45053	NM_0044	FAP	55484	intronic	IFIH1	7.04023	Up	luks_peak	NULL
peak477	chr2	163164476	163164628	1	NM_0121	GCA	36031	NM_0044	FAP	64507	intronic	IFIH1	8.59835	Up	NULL	NULL
peak478	chr2	163583356	163583506	2	NR_11025	LOC10192	42015	NM_0221	IFIH1	408213	intronic	KCNH7	7.64651	Down	NULL	NULL
peak479	chr2	167780296	167780484	3	NM_0011	XIRP2	263403	NR_04562	SCN7A	436909	intronic	XIRP2	4.75426	Down	NULL	PBS_peak_5938
peak480	chr2	176445799	176445981	0	NM_0005	HOXD13	511642	NM_0016	ATP5G3	399400	intergenic	ATP5G3(d	4.01713	Down	NULL	PBS_peak_5952
peak481	chr2	176601186	176601354	0	NM_0005	HOXD13	356262	NM_0016	ATP5G3	554780	intergenic	ATP5G3(d	12.27298	Down	NULL	PBS_peak_5954
peak482	chr2	177650892	177651047	0	NM_1942	HNRNPA1	426452	NR_04000	LINC0111	148667	intergenic	LINC0111	3.56038	Down	NULL	NULL
peak483	chr2	177692055	177692236	0	NM_1942	HNRNPA1	385276	NR_04000	LINC0111	189843	intergenic	LINC0111	16.1389	Down	NULL	PBS_peak_5968
peak484	chr2	177909770	177909955	0	NM_1942	HNRNPA1	167559	NR_04000	LINC0111	407560	intergenic	LINC0111	3.46927	Down	NULL	PBS_peak_5975
peak485	chr2	178125621	178125797	8	NM_0036	AGPS	131762	NR_03607	MIR3128	4971	intronic	NFE2L2	5.03382	Down	NULL	PBS_peak_5982
peak486	chr2	191844640	191844899	2	NM_0011	MYO1B	265337	NM_0011	NEMP2	445301	intronic	STAT1	6.17327	Up	luks_peak	NULL
peak487	chr2	191845353	191845517	2	NM_0011	MYO1B	264672	NM_0011	NEMP2	445967	exonic	STAT1	6.192	Up	NULL	NULL
peak488	chr2	191868467	191868729	2	NM_0011	MYO1B	241509	NM_0011	NEMP2	469130	intronic	STAT1	6.61069	Up	luks_peak	NULL
peak489	chr2	191871206	191871389	2	NM_0011	MYO1B	238809	NM_0011	NEMP2	471829	intronic	STAT1	4.43241	Up	NULL	NULL
peak490	chr2	203135909	203136168	1	NR_00305	SNORD70	5115	NM_0033	SUMO1	32716	intronic	NOP58	13.68783	Down	NULL	PBS_peak_6006
peak491	chr2	203137081	203137260	1	NR_00305	SNORD70	3983	NM_0033	SUMO1	33848	intronic	NOP58	4.46139	Down	NULL	PBS_peak_6008
peak492	chr2	203247530	203247700	1	NM_1735	FAM117B	252286	NM_0033	SUMO1	144293	intronic	BMPR2	7.82403	Up	NULL	NULL
peak493	chr2	203262500	203262721	1	NM_1735	FAM117B	237290	NM_0033	SUMO1	159288	intronic	BMPR2	5.25715	Up	NULL	NULL
peak494	chr2	205553526	205553782	4	NM_2012	NRP2	993570	NM_2033	RAPH1	1153596	intronic	PARD3B	11.27887	Down	NULL	PBS_peak_6019
peak495	chr2	215175960	215176112	3	NM_0010	VWC2L	100425	NM_0012	LOC1001	1027107	intronic	SPAG16	4.27883	Down	NULL	NULL
peak496	chr2	219089566	219089729	2	NM_0010	GPBAR1	36090	NM_0006	CXCR1	57931	intronic	ARPC2	7.09715	Down	NULL	PBS_peak_6038
peak497	chr2	220911473	220911644	0	NM_1530	CCDC140	2251307	NR_03622	MIR4268	140272	intergenic	MIR4268(	9.54916	Down	NULL	PBS_peak_6045
peak498	chr2	220939645	220939814	0	NM_1530	CCDC140	2223136	NR_03622	MIR4268	168443	intergenic	MIR4268(	3.23644	Down	NULL	PBS_peak_6046
peak499	chr2	220996462	220996640	0	NM_1530	CCDC140	2166315	NR_03622	MIR4268	225265	intergenic	MIR4268(	4.15107	Down	NULL	PBS_peak_6047
peak500	chr2	221140089	221140295	0	NM_1530	CCDC140	2022674	NR_03622	MIR4268	368906	intergenic	MIR4268(	7.70876	Down	NULL	PBS_peak_6049
peak501	chr2	221469837	221470023	0	NM_1530	CCDC140	1692936	NR_03622	MIR4268	698644	intergenic	MIR4268(	11.67104	Down	NULL	PBS_peak_6056
peak502	chr2	221720790	221720952	0	NM_1530	CCDC140	1441995	NR_03622	MIR4268	949585	intergenic	MIR4268(	4.08223	Down	NULL	PBS_peak_6058
peak503	chr2	222486023	222486186	0	NM_1530	CCDC140	676761	NM_0013	EPHA4	47182	intergenic	EPHA4(di	11.16893	Down	NULL	NULL
peak504	chr2	229183638	229183800	0	NM_1748	FBXO36	1603488	NM_0306	SPHKAP	137358	intergenic	SPHKAP(	8.87327	Down	NULL	NULL
peak505	chr2	231626122	231626465	3	NM_0012	ITM2C	103019	NR_04003	LOC1514	61049	intronic	CAB39	15.34095	Down	NULL	PBS_peak_6095
peak506	chr2	240216377	240216545	1	NR_03622	MIR4269	10696	NR_02666	MGC1602	99308	intronic	HDAC4	21.30596	Down	NULL	NULL
peak507	chr2	240231906	240232072	1	NR_03780	LOC15093	452565	NR_02666	MGC1602	114836	intronic	HDAC4	7.21394	Down	NULL	NULL
peak508	chr2	242238133	242238288	2	NM_0010	2-Sep	16391	NM_2033	HDLBP	25909	intronic	HDLBP	4.62022	Down	NULL	PBS_peak_6126
peak2289	chr20	435660	435836	2	NM_0010	FAM110A	378592	NR_10999	NRSN2-A	129852	intronic	TBC1D20	4.70065	Down	NULL	NULL
peak2290	chr20	1359767	1359919	4	NR_11009	SIRPG-AS	254311	NM_0804	SDCBP2	49964	ncRNA_in	FKBP1A-S	5.06721	Down	NULL	PBS_peak_6139
peak2291	chr20	17655712	17655898	2	NM_0011	BANF2	18515	NM_0012	BFSP1	105940	intronic	RRBP1	5.2069	Up	NULL	NULL
peak2292	chr20	21293965	21294148	2	NR_10988	LOC10192	256605	NR_10995	KIZ-AS1	95129	intronic	XRN2	6.29475	Down	NULL	PBS_peak_6142
peak2293	chr20	21295504	21295712	2	NR_10988	LOC10192	255054	NR_10995	KIZ-AS1	96681	intronic	XRN2	4.23274	Down	NULL	PBS_peak_6143
peak2294	chr20	21646867	21647027	1	NM_0012	PAX1	39350	NM_0025	NKX2-2	152283	ncRNA_in	LINC0172	3.34875	Down	NULL	NULL
peak2295	chr20	21720258	21720456	0	NR_03839	LINC0143	314371	NR_10987	LOC10192	36134	intergenic	PAX1(dist	3.95939	Down	NULL	PBS_peak_6155
peak2296	chr20	21900764	21900924	0	NR_03839	LINC0143	133884	NR_10987	LOC10192	216621	intergenic	PAX1(dist	6.1054	Down	NULL	PBS_peak_6192
peak2297	chr20	22043358	22043534	1	NM_0010	SSTR4	972611	NR_10987	LOC10192	359223	ncRNA_in	LINC0143	4.55006	Down	NULL	PBS_peak_6216
peak2298	chr20	22409454	22409611	0	NM_0010	SSTR4	606524	NR_02708	LOC28478	8251	intergenic	LOC28478	3.66206	Down	NULL	PBS_peak_6253
peak2299	chr20	22424702	22424872	0	NM_0010	SSTR4	591270	NR_02708	LOC28478	23506	intergenic	LOC28478	13.36409	Down	NULL	PBS_peak_6255
peak2300	chr20	22536653	22536817	0	NM_0010	SSTR4	479322	NR_02708	LOC28478	135454	intergenic	LOC28478	5.99484	Down	NULL	PBS_peak_6268
peak2301	chr20	22545384	22545546	1	NM_0010	SSTR4	470592	NR_02708	LOC28478	144184	ncRNA_ex	LINC0026	6.12681	Down	NULL	PBS_peak_6269
peak2302	chr20	24465095	24465268	1	NM_0036	CST7	464684	NM_1783	GGTLC1	495765	intronic	SYNDIG1	8.01711	Down	NULL	NULL
peak2303	chr20	30704193	30704350	1	NM_1722	POFUT1	91424	NM_0308	PDRG1	164388	intronic	TM9SF4	13.73529	Down	NULL	NULL
peak2304	chr20	30951464	30951701	2	NR_11061	LOC10192	146179	NM_0026	PLAGL2	156036	intronic	ASXL1	3.9158	Down	NULL	PBS_peak_6291
peak2305	chr20	31409573	31409727	1	NM_0252	BPIFB2	185734	NM_0530	COMMD7	77836	intronic	MAPRE1	4.03071	Down	NULL	NULL
peak2306	chr20	32417330	32417482	1	NM_0073	RALY	164052	NR_11062	ZNF341-A	18501	intronic	CHMP4B	4.92336	Up	luks_peak	NULL
peak2307	chr20	32419641	32419791	1	NM_0073	RALY	161742	NR_11062	ZNF341-A	20811	intronic	CHMP4B	4.79946	Up	luks_peak	NULL
peak2308	chr20	32420053	32420207	1	NM_0073	RALY	161328	NR_11062	ZNF341-A	21225	intronic	CHMP4B	4.59078	Up	NULL	NULL
peak2309	chr20	32429253	32429465	1	NM_0073	RALY	152099	NR_11062	ZNF341-A	30454	intronic	CHMP4B	5.02059	Up	luks_peak	NULL
peak2310	chr20	32435288	32435461	1	NM_0073	RALY	146083	NR_11062	ZNF341-A	36469	intronic	CHMP4B	20.63325	Up	luks_peak	NULL
peak2311	chr20	32436006	32436238	1	NM_0073	RALY	145336	NR_11062	ZNF341-A	37217	intronic	CHMP4B	7.05386	Up	luks_peak	NULL
peak2312	chr20	32437697	32437891	1	NM_0073	RALY	143664	NR_11062	ZNF341-A	38889	intronic	CHMP4B	5.45747	Up	NULL	NULL
peak2313	chr20	32595006	32595197	2	NR_03991	MIR4755	41823	NR_10988	RALY-AS	12928	intronic	RALY	5.47099	Down	NULL	PBS_peak_6314
peak2314	chr20	32597188	32597405	2	NR_03991	MIR4755	39628	NR_10988	RALY-AS	15123	intronic	RALY	5.81025	Down	NULL	PBS_peak_6315
peak2315	chr20	32600534	32600723	2	NR_03991	MIR4755	36296	NR_10988	RALY-AS	18455	intronic	RALY	4.33332	Down	NULL	PBS_peak_6317
peak2316	chr20	32607182	32607348	2	NR_03991	MIR4755	29660	NR_10988	RALY-AS	25092	intronic	RALY	10.51152	Down	NULL	PBS_peak_6322
peak2317	chr20	34242824	34243080	10	NM_0807	ROMO1	44280	NM_0039	CPNE1	1121	exonic	RBM12	4.14253	Down	NULL	PBS_peak_6346
peak2318	chr20	35825662	35825819	2	NM_0010	MANBAL	92310	NM_2136	MROH8	17749	intronic	RPN2	3.70676	Down	NULL	PBS_peak_6366
peak2319	chr20	37053887	37054063	3	NR_00323	SNHG11	21322	NM_0010	KIAA175	164801	ncRNA_ex	SNORA71	9.69493	Down	NULL	PBS_peak_6370
peak2320	chr20	37055341	37055615	2	NR_00323	SNHG11	19819	NR_00291	SNORA71	1500	ncRNA_in	SNHG17	5.38522	Down	NULL	PBS_peak_6371
peak2321	chr20	37055752	37055918	2	NR_00323	SNHG11	19462	NR_00291	SNORA71	1857	ncRNA_in	SNHG17	13.66764	Down	NULL	PBS_peak_6371
peak2322	chr20	37059555	37059737	2	NR_00323	SNHG11	15651	NR_00301	SNORA71	1199	ncRNA_ex	SNHG17	4.65507	Down	NULL	PBS_peak_6374
peak2323	chr20	39674560	39674750	1	NM_0026	PLCG1	91506	NM_0054	MAFB	356775	intronic	TOP1	4.89597	Down	NULL	PBS_peak_6380
peak2324	chr20	39676366	39676533	1	NM_0026	PLCG1	89711	NM_0054	MAFB	358569	intronic	TOP1	31.03999	Down	NULL	NULL
peak2325	chr20	47549325	47549478	1	NM_0012	CSE1L	113381	NM_0208	PREX1	104981	intronic	ARFGEF2	8.03217	Down	NULL	PBS_peak_6404

peak2326	chr20	47672684	47672840	3	NM_0178	DDX27	163070	NR_11062	CSE1L-A	10169	intronic	CSE1L	4.15769	Down	NULL	NULL
peak2327	chr20	47673993	47674173	3	NM_0178	DDX27	161749	NR_11062	CSE1L-A	11490	intronic	CSE1L	6.79934	Down	NULL	PBS_peak_6411
peak2328	chr20	47862656	47862912	1	NR_00360	ZFAS1	31931	NM_001319135		26952	UTR3	ZNFX1(N	8.57002	Up	luks_peak	NULL
peak2329	chr20	47866836	47867009	1	NR_00360	ZFAS1	27792	NM_001319135		31090	intronic	ZNFX1	7.00149	Up	luks_peak	NULL
peak2330	chr20	47871192	47871376	1	NR_00360	ZFAS1	23431	NM_001319135		35452	intronic	ZNFX1	4.06512	Up	luks_peak	NULL
peak2331	chr20	47871715	47871931	1	NR_00360	ZFAS1	22892	NM_001319135		35991	intronic	ZNFX1	12.12284	Up	NULL	NULL
peak2332	chr20	47877080	47877239	1	NR_00360	ZFAS1	17555	NM_001319135		41327	exonic	ZNFX1	5.25885	Up	NULL	NULL
peak2333	chr20	47879774	47879956	1	NR_00360	ZFAS1	14850	NM_001319135		44033	exonic	ZNFX1	4.67072	Up	luks_peak	NULL
peak2334	chr20	47880402	47880632	1	NR_00360	ZFAS1	14198	NM_001319135		44685	intronic	ZNFX1	4.06883	Up	luks_peak	NULL
peak2335	chr20	48313709	48313869	1	NM_0012	SLC9A8	115461	NM_0009	PTGIS	129082	intronic	B4GALT5	5.52392	Down	NULL	PBS_peak_6425
peak2336	chr20	48316171	48316413	1	NM_0012	SLC9A8	112958	NM_0009	PTGIS	131585	intronic	B4GALT5	21.40568	Down	NULL	PBS_peak_6427
peak2337	chr20	49143310	49143494	2	NR_03037	MIR645	58921	NR_10995	LINC0127	205523	intronic	PTPN1	10.68653	Down	NULL	PBS_peak_6435
peak2338	chr20	49526883	49527054	3	NR_11000	ADNP-AS	20552	NM_0012	ADNP	640	intronic	ADNP	7.17703	Down	NULL	PBS_peak_6438
peak2339	chr20	53646915	53647073	0	NR_11062	LINC0144	392587	NM_0011	CYP24A1	856478	intergenic	DOK5(dis	10.10682	Down	NULL	NULL
peak2340	chr20	54036668	54036832	0	NR_11062	LINC0144	2831	NM_0011	CYP24A1	1246234	downstrea	LINC0144	13.86092	Up	NULL	NULL
peak2341	chr20	60622605	60622794	1	NR_13046	MIR3195	17158	NR_03165	MIR1257	93981	intronic	TAF4	5.39868	Down	NULL	PBS_peak_6461
peak2342	chr20	60623337	60623538	1	NR_13046	MIR3195	16420	NR_03165	MIR1257	94719	intronic	TAF4	7.3402	Down	NULL	PBS_peak_6462
peak2343	chr20	60628029	60628200	1	NR_13046	MIR3195	11743	NR_03165	MIR1257	99396	intronic	TAF4	4.46622	Down	NULL	PBS_peak_6466
peak2344	chr20	60632486	60632638	1	NR_13046	MIR3195	7296	NR_03165	MIR1257	103844	intronic	TAF4	20.06384	Down	NULL	PBS_peak_6468
peak2345	chr20	61444924	61445092	1	NM_0018	COL9A3	3406	NR_10243	OGFR-AS	8069	exonic	OGFR	6.73912	Up	NULL	NULL
peak2346	chr20	61565403	61565554	3	NM_0178	GID8	3962	NM_0807	DIDO1	7575	intronic	DIDO1	4.10362	Down	NULL	PBS_peak_6486
peak2347	chr20	61840906	61841269	1	NM_0221	BIRC7	26147	NR_00324	HAR1B	107416	intronic	YTHDF1	5.15639	Down	NULL	PBS_peak_6500
peak2348	chr20	62188764	62188934	0	NR_132418		69739	NM_0808	SRMS	9992	downstrea	FNDC11,	4.9459	Up	NULL	NULL
peak2349	chr20	62193778	62193937	2	NR_132418		64730	NM_0808	SRMS	15000	exonic	HELZ2	23.76963	Up	NULL	NULL
peak2350	chr20	62194504	62194674	2	NR_132418		63999	NM_0808	SRMS	15732	exonic	HELZ2	8.06688	Up	luks_peak	NULL
peak2351	chr20	62196454	62196675	2	NR_132418		62023	NM_0808	SRMS	17707	exonic	HELZ2	12.112	Up	luks_peak	NULL
peak2352	chr20	62196775	62196947	2	NR_132418		61727	NM_0808	SRMS	18004	exonic	HELZ2	6.03251	Up	luks_peak	NULL
peak2353	chr20	62197789	62198013	2	NR_132418		60687	NM_0808	SRMS	19044	exonic	HELZ2	5.52833	Up	luks_peak	NULL
peak2354	chr21	10834253	10834489	0	NR_02691	ANKRD3	3576116	NR_03832	TEKT4P2	865777	intergenic	TEKT4P2	4.24824	Down	NULL	PBS_peak_6590
peak2355	chr21	18471252	18471405	0	NR_03758	LINC0154	339879	NM_0034	NRIP1	2034202	intergenic	MIR99AH	8.64608	Down	NULL	PBS_peak_6621
peak2356	chr21	23559658	23559816	0	NR_04619	LOC33962	2653127	NR_10995	LOC1019	88959	intergenic	LINC0030	3.89347	Up	NULL	NULL
peak2357	chr21	33101223	33101408	3	NM_0145	HUNK	144312	NM_0032	TIAM1	170025	intronic	SCAF4	7.53908	Down	NULL	PBS_peak_6638
peak2358	chr21	34789255	34789432	1	NM_0012	SON	126000	NR_03897	IL10RB-A	150778	intronic	IFNGR2	6.20008	Up	NULL	NULL
peak2359	chr21	38756513	38756726	1	NM_1304	DYRK1A	34587	NM_0060	DSCR3	116786	intronic	DYRK1A	3.75642	Down	NULL	PBS_peak_6671
peak2360	chr21	42739983	42740212	1	NM_0011	MX1	52387	NM_1828	PLAC4	182931	intronic	MX2	18.79384	Up	luks_peak	NULL
peak2361	chr21	42740445	42740659	1	NM_0011	MX1	51933	NM_1828	PLAC4	183386	intronic	MX2	15.51898	Up	luks_peak	NULL
peak2362	chr21	42743651	42743890	1	NM_0011	MX1	48714	NM_1828	PLAC4	186604	intronic	MX2	25.74346	Up	luks_peak	NULL
peak2363	chr21	42745208	42745372	1	NM_0011	MX1	47195	NM_1828	PLAC4	188124	intronic	MX2	20.07876	Up	luks_peak	NULL
peak2364	chr21	42745467	42745929	1	NM_0011	MX1	46787	NM_1828	PLAC4	188532	intronic	MX2	16.37003	Up	luks_peak	NULL
peak2365	chr21	42746034	42746200	1	NM_0011	MX1	46368	NM_1828	PLAC4	188951	intronic	MX2	5.73985	Up	luks_peak	NULL
peak2366	chr21	42746686	42746857	1	NM_0011	MX1	45713	NM_1828	PLAC4	189605	intronic	MX2	4.64481	Up	NULL	NULL
peak2367	chr21	42746925	42747132	1	NM_0011	MX1	45456	NM_1828	PLAC4	189862	intronic	MX2	6.74404	Up	NULL	NULL
peak2368	chr21	42747243	42747409	1	NM_0011	MX1	45159	NM_1828	PLAC4	190160	intronic	MX2	7.36511	Up	luks_peak	NULL
peak2369	chr21	42747762	42748156	1	NM_0011	MX1	44526	NM_1828	PLAC4	190793	intronic	MX2	10.84231	Up	luks_peak	NULL
peak2370	chr21	42748527	42748705	1	NM_0011	MX1	43869	NM_1828	PLAC4	191450	intronic	MX2	12.51349	Up	NULL	NULL
peak2371	chr21	42750865	42751122	1	NM_0011	MX1	41491	NM_1828	PLAC4	193827	intronic	MX2	18.83931	Up	luks_peak	NULL
peak2372	chr21	42751529	42752150	1	NM_0011	MX1	40645	NM_1828	PLAC4	194673	exonic	MX2	14.14675	Up	luks_peak	NULL
peak2373	chr21	42754019	42754187	1	NM_0011	MX1	38382	NM_1828	PLAC4	196937	intronic	MX2	8.07017	Up	NULL	NULL
peak2374	chr21	42757268	42757470	1	NM_0011	MX1	35116	NM_1828	PLAC4	200203	intronic	MX2	22.58492	Up	NULL	NULL
peak2375	chr21	42759389	42759548	1	NM_0011	MX1	33016	NM_1828	PLAC4	202302	intronic	MX2	4.80526	Up	NULL	NULL
peak2376	chr21	42759899	42760075	1	NM_0011	MX1	32498	NM_1828	PLAC4	202821	intronic	MX2	15.20371	Up	NULL	NULL
peak2377	chr21	42762421	42762661	1	NM_0011	MX1	29944	NM_1828	PLAC4	205375	exonic	MX2	14.76194	Up	NULL	NULL
peak2378	chr21	42818160	42818326	4	NR_02436	LINC0011	281219	NM_1828	PLAC4	261077	intronic	MX1	4.69442	Up	NULL	NULL
peak2379	chr21	42827097	42827318	4	NR_02436	LINC0011	272254	NM_1828	PLAC4	270041	intronic	MX1	16.22642	Up	NULL	NULL
peak2380	chr21	44267894	44268080	1	NM_0010	NDUFV3	45391	NR_13119	LOC1019	232782	intronic	WDR4	6.49557	Down	NULL	PBS_peak_6694
peak2381	chr21	44288850	44289019	7	NM_0010	NDUFV3	24443	NR_13119	LOC1019	253729	intronic	WDR4	4.62192	Down	NULL	PBS_peak_6698
peak2382	chr21	44316611	44316769	2	NM_001320694		77930	NR_04853	WDR4	16991	intronic	NDUFV3	4.05356	Down	NULL	PBS_peak_6699
peak2383	chr21	45364222	45364383	2	NM_0032	TRAPPC1	67903	NR_02696	AATBC	131854	intronic	AGPAT3	5.60921	Up	luks_peak	NULL
peak2384	chr21	45439594	45439881	1	NM_0050	PWP2	87470	NR_02696	AATBC	207289	intronic	TRAPPC1	4.38175	Down	NULL	PBS_peak_6705
peak2385	chr21	46378597	46378779	7	NM_0011	ADARB1	115805	NR_02712	LINC0154	18860	intronic	FAM207A	7.49254	Down	NULL	PBS_peak_6718
peak2386	chr22	18637516	18637961	1	NM_0056	DGCR6	255997	NM_0152	MICAL3	130413	intronic	USP18	7.25977	Up	luks_peak	NULL
peak2387	chr22	18638135	18638420	1	NM_0056	DGCR6	255458	NM_0152	MICAL3	130952	intronic	USP18	5.23905	Up	luks_peak	NULL
peak2388	chr22	18638509	18638662	1	NM_0056	DGCR6	255150	NM_0152	MICAL3	131260	intronic	USP18	20.82199	Up	NULL	NULL
peak2389	chr22	18638824	18639303	1	NM_0056	DGCR6	254672	NM_0152	MICAL3	131738	intronic	USP18	6.16918	Up	luks_peak	NULL
peak2390	chr22	18642741	18642980	1	NM_0056	DGCR6	250875	NM_0152	MICAL3	135535	exonic	USP18	7.52929	Up	NULL	NULL
peak2391	chr22	18646643	18646865	1	NM_0056	DGCR6	246982	NM_0152	MICAL3	139429	intronic	USP18	6.17971	Up	NULL	NULL
peak2392	chr22	18652314	18652475	1	NM_0056	DGCR6	241341	NM_0152	MICAL3	145069	intronic	USP18	7.6386	Up	NULL	NULL
peak2393	chr22	19104220	19104409	5	NM_0530	TSSK2	14006	NR_02415	DGCR11	68426	intronic	DGCR2	17.10838	Down	NULL	PBS_peak_6728
peak2394	chr22	21276555	21276750	1	NR_11053	LOC1019	34727	NM_0580	PI4KA	63552	intronic	CRKL	5.66521	Down	NULL	PBS_peak_6755
peak2395	chr22	21295300	21295461	1	NR_11053	LOC1019	15999	NM_0580	PI4KA	82280	intronic	CRKL	4.42196	Down	NULL	PBS_peak_6768
peak2396	chr22	22175837	22176004	2	NM_0013	VPREB1	423271	NR_13091	YPEL1	85797	intronic	MAPK1	6.6989	Down	NULL	PBS_peak_6813
peak2397	chr22	24671089	24671255	4	NM_0012	SPECC1L	66904	NM_0041	GGT5	30062	ncRNA_in	SPECC1L	6.20471	Down	NULL	PBS_peak_6824
peak2398	chr22	26980119	26980322	1	NM_0018	CRYBA4	37707	NM_0010	TPST2	18850	intronic	TPST2	4.19105	Up	luks_peak	NULL
peak2399	chr22	29778799	29778962	3	NM_0210	RFPL1	55691	NR_03742	MIR3653	49624	intronic	AP1B1	5.46901	Down	NULL	PBS_peak_6828
peak2400	chr22	35710254	35710411	6	NR_03747	MIR3909	21300	NR_12635	LINC0139	83283	intronic	TOM1	6.6129	Up	NULL	NULL
peak2401	chr22	36050154	36050495	1	NM_0306	APOL5	63594	NM_2033	MB	30923	intronic	APOL6	12.61668	Up	luks_peak	NULL
peak2402	chr22	36739903	36740064	1	NR_134623		359937	NR_10687	MIR6819	57030	intronic	MYH9	5.29623	Down	NULL	PBS_peak_6842
peak2403	chr22	36746354	36746522	1	NR_134623		353483	NR_10687	MIR6819	63485	intronic	MYH9	3.76699	Down	NULL	PBS_peak_6844
peak2404	chr22	36747226	36747423	1	NR_134623		352596	NR_10687	MIR6819	64371	intronic	MYH9	6.08737	Down	NULL	PBS_peak_6845
peak2405	chr22	36752773	36752934	1	NR_134623		347067	NR_10687	MIR6819	69900	intronic	MYH9	21.00759	Down	NULL	PBS_peak_6847
peak2406	chr22	36753415	36753683	1	NR_134623		346372	NR_10687	MIR6819	70596	intronic	MYH9	9.21284	Down	NULL	PBS_peak_6847
peak2407	chr22	36754039	36754207	1	NR_134623		345798	NR_10687	MIR6819	71170	intronic	MYH9	11.44719	Down	NULL	PBS_peak_6848
peak2408	chr22	36755022	36755278	1	NR_134623		344771	NR_10687	MIR6819	72197	intronic	MYH9	21.69778	Down	NULL	PBS_peak_6849
peak2409	chr22	36757065	36757241	1	NR_134623		342768	NR_10687	MIR6819	74200	intronic	MYH9	5.85071	Down	NULL	PBS_peak_6850
peak2410	chr22	36758609	36758782	1	NR_134623		341225	NR_10687	MIR6819	75742	intronic	MYH9	5.21952	Down	NULL	PBS_peak_6853
peak2411	chr22	36759032	36759321	1	NR_134623		340744	NR_10687	MIR6819	76223	intronic	MYH9	8.57848	Down	NULL	PBS_peak_6854
peak2412	chr22	36759449	36759645	1	NR_134623		340374	NR_10687	MIR6819	76594	intronic	MYH9	4.33768	Down	NULL	PBS_peak_6854
peak2413	chr22	36760214	36761005	1	NR_134623		339311	NR_10687	MIR6819	77656	intronic	MYH9	7.10698	Down	NULL	PBS_peak_6855
peak2414	chr22	36761309	36761537	1	NR_134623		338498	NR_10687	MIR6819	78470	intronic	MYH9	8.89264	Down	NULL	PBS_peak_6855
peak2415	chr22	36761711	36762178	1	NR_134623		337976	NR_10687	MIR6819	78991	intronic	MYH9	20.4612	Down	NULL	PBS_peak_6855
peak2416	chr22	36762304	36762552	1	NR_134623		337493	NR_10687	MIR6819	79475	intronic	MYH9	6.71966	Down	NULL	PBS_peak_6855
peak2417	chr22	36763187	36763338	1	NR_134623		336658	NR_10687	MIR6819	80309	intronic	MYH9	3.69646	Down	NULL	PBS_peak_6855
peak2418	chr22	36763851	36764028	1	NR_134623		335981	NR_10687	MIR6819	80986	intronic	MYH9	4.76412	Down	NULL	PBS_peak_6855
peak2419	chr22	36764720	36764904	1	NR_134623		335109	NR_10687	MIR6819	81859	intronic	MYH9	3.95746	Down	NULL	PBS_peak_6856
peak2420	chr22	36765372	36765957	1	NR_134623		334256	NR_10687	MIR6819	82711	intronic	MYH9	6.58001	Down	NULL	PBS_peak_6856
peak2421	chr22	36768696	36769033	1	NR_134623		331056	NR_10687	MIR6819	85911	intronic	MYH9	8.72212	Down	NULL	PBS_peak_6860
peak2422	chr22	36771714	36771903	1	NR_134623		328112	NR_10687	MIR6819	88855	intronic	MYH9	3.58122	Down	NULL	PBS_peak_6861
peak2423	chr22	36772488	36772943	1	NR_134623		327205	NR_10687	MIR6819	89762	intronic	MYH9	4.53154	Down	NULL	PBS_peak_6862
peak2424	chr22	36773464	36773927	1	NR_134623		326225	NR_10687	MIR6819	90742	intronic	MYH9	3.93647	Down	NULL	PBS_peak_6863
peak2425	chr22	36774629	36774961	1	NR_134623		325126	NR_10687	MIR6819	91842	intronic	MYH9	5.58183	Down	NULL	PBS_peak_6864
peak2426	chr22	36776410	36776589	1	NR_134623		323421	NR_10687	MIR6819	93546	intronic	MYH9	3.56992	Down	NULL	PBS_peak_6866
peak2427	chr22	36777216	36777453	1	NR_134623		322586	NR_10687	MIR6819	94381	intronic	MYH9	4.95706	Down	NULL	PBS_peak_6867
peak2428	chr22	38056296	38056468	1	NM_0023	LGALS1	15231	NR_10995	LOC1019	1998	intronic	PDXP	11.2576	Down	NULL	PBS_peak_6869

peak2429	chr22	39110148	39110405	1	NM_0012	APOBEC3	243250	NM_0148	JOSD1	13817	intronic	GTPBP1	4.67774	Up	luks_peak	NULL
peak2430	chr22	39110567	39110737	1	NM_0012	APOBEC3	242875	NM_0148	JOSD1	14193	intronic	GTPBP1	18.7812	Up	luks_peak	NULL
peak2431	chr22	39112200	39112501	1	NM_0012	APOBEC3	241176	NM_0148	JOSD1	15891	intronic	GTPBP1	8.60341	Up	luks_peak	NULL
peak2432	chr22	39112898	39113084	1	NM_0012	APOBEC3	240536	NM_0148	JOSD1	16532	exonic	GTPBP1	5.30733	Up	luks_peak	NULL
peak2433	chr22	39113378	39113548	1	NM_0012	APOBEC3	240064	NM_0148	JOSD1	17004	intronic	GTPBP1	4.08181	Up	luks_peak	NULL
peak2434	chr22	39113699	39113888	1	NM_0012	APOBEC3	239733	NM_0148	JOSD1	17334	intronic	GTPBP1	4.22599	Up	NULL	NULL
peak2435	chr22	39124040	39124266	1	NM_0012	APOBEC3	229374	NM_0148	JOSD1	27694	exonic	GTPBP1	7.18307	Up	luks_peak	NULL
peak2436	chr22	41873178	41873363	1	NM_0144	CSDC2	83743	NM_0327	PHF5A	8562	intronic	ACO2	28.56655	Down	NULL	PBS_peak_6887
peak2437	chr22	41874166	41874403	1	NM_0144	CSDC2	82729	NM_0327	PHF5A	9576	intronic	ACO2	4.43779	Down	NULL	PBS_peak_6888
peak2438	chr22	41878306	41878472	1	NM_0144	CSDC2	78625	NM_0327	PHF5A	13681	intronic	ACO2	6.40437	Down	NULL	PBS_peak_6890
peak2439	chr22	41889215	41889405	1	NM_0144	CSDC2	67704	NM_0327	PHF5A	24602	intronic	ACO2	6.51431	Down	NULL	PBS_peak_6898
peak2440	chr22	41890976	41891193	1	NM_0144	CSDC2	65929	NM_0327	PHF5A	26376	intronic	ACO2	5.47642	Down	NULL	PBS_peak_6899
peak2441	chr22	42076719	42076879	2	NM_0011	C22orf46	9748	NM_0157	DESI1	59738	intronic	SNU13	14.76184	Down	NULL	NULL
peak2442	chr22	43002812	43002972	4	NR_02942	RNU12	8359	NR_00218	RRP7BP	24875	intronic	POLDIP3	6.37441	Down	NULL	PBS_peak_6906
peak2443	chr22	43333375	43333534	3	NR_12530	LOC10050	101136	NM_0145	ARFGAP3	80046	intronic	PACSIN2	3.55454	Down	NULL	NULL
peak2444	chr22	45594856	45595019	2	NM_0011	UPK3A	85930	NR_03895	NUP50-A	35275	intronic	KIAA0930	5.8845	Down	NULL	NULL
peak2445	chr22	46075853	46076010	2	NR_03991	MIR4762	80472	NR_13124	LINC0158	74404	intronic	ATXN10	16.3216	Down	NULL	NULL
peak2446	chr22	47030286	47030454	1	NM_0012	TBC1D22	128144	NM_0142	CELSR1	97303	intronic	GRAMD4	5.13541	Down	NULL	PBS_peak_6923
peak2447	chr22	47042371	47042701	1	NM_0012	TBC1D22	115978	NM_0142	CELSR1	109469	intronic	GRAMD4	4.63766	Down	NULL	PBS_peak_6928
peak2448	chr22	47047823	47048019	1	NM_0012	TBC1D22	110593	NM_0142	CELSR1	114854	intronic	GRAMD4	3.90677	Down	NULL	PBS_peak_6933
peak2449	chr22	47049364	47049602	1	NM_0012	TBC1D22	109031	NM_0142	CELSR1	116416	intronic	GRAMD4	9.67904	Down	NULL	PBS_peak_6935
peak2450	chr22	50206451	50206750	2	NM_0148	ZBED4	40896	NM_0012	C22orf34	155410	intronic	BRD1	5.57767	Down	NULL	PBS_peak_6944
peak509	chr3	8246440	8246661	0	NM_0012	LMCD1	296942	NR_11013	LOC10192	188556	intergenic	LOC10192	4.49793	Up	NULL	NULL
peak510	chr3	25315857	25316068	1	NM_0012	RARB	66330	NR_03995	MIR4792	753036	intronic	RARB	11.15756	Down	NULL	PBS_peak_7015
peak511	chr3	41246280	41246441	3	NM_0012	TRAK1	886385	NR_04010	ENTPD3-	751561	intronic	CTNNB1	4.61396	Down	NULL	PBS_peak_7020
peak512	chr3	41260236	41260393	3	NM_0012	TRAK1	872431	NR_04010	ENTPD3-	765515	intronic	CTNNB1	4.8842	Down	NULL	PBS_peak_7026
peak513	chr3	46987536	46987727	3	NM_0151	NBEAL2	33541	NM_0002	MYL3	82658	intronic	CCDC12	5.68987	Down	NULL	PBS_peak_7035
peak514	chr3	46988474	46988774	3	NM_0151	NBEAL2	32549	NM_0002	MYL3	83651	intronic	CCDC12	5.33064	Down	NULL	PBS_peak_7037
peak515	chr3	46994449	46994604	3	NM_0151	NBEAL2	26646	NM_0002	MYL3	89553	intronic	CCDC12	8.54347	Down	NULL	PBS_peak_7043
peak516	chr3	47001904	47002118	3	NM_0151	NBEAL2	19162	NM_0002	MYL3	97038	intronic	CCDC12	5.06387	Down	NULL	PBS_peak_7048
peak517	chr3	47012965	47013146	3	NM_0151	NBEAL2	8117	NM_0002	MYL3	108082	intronic	CCDC12	5.9197	Down	NULL	PBS_peak_7052
peak518	chr3	47178797	47178952	1	NR_03337	KIF9-AS1	26985	NM_1447	CCDC12	155374	intronic	SETD2	5.4052	Down	NULL	PBS_peak_7054
peak519	chr3	47180697	47180847	1	NR_03337	KIF9-AS1	25088	NM_1447	CCDC12	157272	intronic	SETD2	6.40176	Down	NULL	PBS_peak_7055
peak520	chr3	49426512	49426684	7	NM_0221	TCTA	23041	NM_2013	GPX1	30807	intronic	RHOA	3.53176	Down	NULL	PBS_peak_7075
peak521	chr3	52296494	52296671	1	NM_0011	GLYCTK	25253	NM_0072	TWF2	23399	intronic	WDR82	8.02548	Down	NULL	NULL
peak522	chr3	52301311	52301513	1	NM_0011	GLYCTK	20424	NM_0072	TWF2	28229	intronic	WDR82	4.53347	Down	NULL	PBS_peak_7097
peak523	chr3	52305510	52305674	1	NM_0011	GLYCTK	16244	NR_02966	MIRLET7	3215	intronic	WDR82	12.32045	Down	NULL	PBS_peak_7100
peak524	chr3	52309693	52309844	1	NM_0011	GLYCTK	12067	NR_02966	MIRLET7	7391	intronic	WDR82	8.67361	Down	NULL	NULL
peak525	chr3	53279992	53280154	4	NM_0007	CACNA1I	249003	NM_0528	RFT1	115603	intronic	TKT	9.27014	Down	NULL	NULL
peak526	chr3	57251083	57251248	0	NM_0120	APPL1	10599	NM_0038	HESX1	16885	intergenic	HESX1(di	25.20052	Up	NULL	NULL
peak527	chr3	57251627	57251968	0	NM_0120	APPL1	9967	NM_0038	HESX1	17517	intergenic	HESX1(di	9.43388	Up	NULL	NULL
peak528	chr3	62492297	62492459	3	NR_02710	LINC0069	595986	NM_0180	FEZF2	133188	intronic	CADPS	11.57775	Down	NULL	NULL
peak529	chr3	63064794	63064986	0	NR_02710	LINC0069	23474	NM_1833	CADPS	203826	intergenic	CADPS(di	5.39557	Down	NULL	PBS_peak_7114
peak530	chr3	64240334	64240500	0	NR_11015	ADAMTS	306605	NM_1988	PRICKLE	29286	intergenic	PRICKLE	5.63992	Down	NULL	PBS_peak_7118
peak531	chr3	72436210	72436373	1	NM_0010	GXYLT2	501093	NR_1041	LINC0087	286685	intronic	RYBP	17.94906	Up	NULL	NULL
peak532	chr3	73097164	73097322	4	NM_0180	EBLN2	13567	NM_0181	SHQ1	199645	intronic	PPP4R2	5.98926	Down	NULL	PBS_peak_7159
peak533	chr3	86653660	86653830	0	NR_04740	LINC0050	484685	NR_04675	CADM2-A	776545	intergenic	LINC0207	4.30929	Down	NULL	NULL
peak534	chr3	88558052	88558214	0	NM_1826	EPHA3	598541	NM_0011	CGGBP1	359117	intergenic	C3orf38(d	3.57272	Down	NULL	PBS_peak_7165
peak535	chr3	94307785	94307938	0	NR_01540	LINC0087	349245	NM_0011	DHFRL1	525794	downstrea	MIR6730	3.30909	Up	NULL	NULL
peak536	chr3	98134184	98134351	0	NM_0010	OR5K1	54056	NR_04768	GABRR3	380119	intergenic	OR5K3(di	4.75233	Up	NULL	NULL
peak537	chr3	116183459	116183613	0	NR_01539	TUSC7	245099	NM_0023	LSAMP	19151	intergenic	LSAMP(d	11.58873	Down	NULL	NULL
peak538	chr3	122256558	122256728	6	NM_1382	DTX3L	26542	NR_02669	KPNA1	22857	intronic	PARP9	6.23273	Up	NULL	NULL
peak539	chr3	122269691	122269845	6	NM_1382	DTX3L	13417	NR_02669	KPNA1	35982	intronic	PARP9	4.81403	Up	NULL	NULL
peak540	chr3	122405013	122405168	1	NM_0328	DIRC2	108810	NM_0314	PARP9	121567	intronic	PARP14	4.83948	Up	NULL	NULL
peak541	chr3	122415820	122415980	1	NM_0328	DIRC2	98001	NM_0314	PARP9	132377	intronic	PARP14	6.33513	Up	NULL	NULL
peak542	chr3	122417210	122417431	1	NM_0328	DIRC2	96580	NM_0314	PARP9	133797	intronic	PARP14	12.71021	Up	NULL	NULL
peak543	chr3	122419871	122420191	1	NM_0328	DIRC2	93870	NM_0314	PARP9	136508	exonic	PARP14	6.68202	Up	luks_peak	NULL
peak544	chr3	122420701	122421105	1	NM_0328	DIRC2	92998	NM_0314	PARP9	137380	intronic	PARP14	15.36516	Up	luks_peak	NULL
peak545	chr3	122422624	122422813	1	NM_0328	DIRC2	91182	NM_0314	PARP9	139195	exonic	PARP14	5.90326	Up	NULL	NULL
peak546	chr3	122426373	122426647	1	NM_0328	DIRC2	87391	NM_0314	PARP9	142987	intronic	PARP14	26.75265	Up	luks_peak	NULL
peak547	chr3	122433187	122433508	1	NM_0328	DIRC2	80553	NM_0314	PARP9	149824	exonic	PARP14	12.09084	Up	luks_peak	NULL
peak548	chr3	122435625	122435794	1	NM_0328	DIRC2	78191	NM_0314	PARP9	152186	intronic	PARP14	16.69191	Up	NULL	NULL
peak549	chr3	122436062	122436234	1	NM_0328	DIRC2	77753	NM_0314	PARP9	152625	intronic	PARP14	14.84797	Up	NULL	NULL
peak550	chr3	122437456	122437622	1	NM_0328	DIRC2	76362	NM_0314	PARP9	154016	exonic	PARP14	8.14796	Up	NULL	NULL
peak551	chr3	122438255	122438417	1	NM_0328	DIRC2	75565	NM_0314	PARP9	154813	intronic	PARP14	5.53735	Up	NULL	NULL
peak552	chr3	125081723	125081906	1	NR_02425	FAM86JP	553629	NM_0246	SLC12A8	150205	intronic	ZNF148	19.45558	Down	NULL	PBS_peak_7186
peak553	chr3	128889797	128890114	6	NM_0161	COPG1	78497	NM_0207	ISY1	9882	exonic	CNBP	5.40398	Down	NULL	PBS_peak_7223
peak554	chr3	128894398	128894648	6	NM_0161	COPG1	73930	NM_0207	ISY1	14450	intronic	CNBP	4.28953	Down	NULL	PBS_peak_7224
peak555	chr3	135876065	135876260	2	NM_0005	PCCB	93004	NM_1785	KY	1506298	intronic	MSL2	6.57311	Down	NULL	PBS_peak_7238
peak556	chr3	135880250	135880422	2	NM_0005	PCCB	88831	NM_1785	KY	1510472	intronic	MSL2	7.4553	Down	NULL	PBS_peak_7241
peak557	chr3	135883204	135883393	2	NM_0005	PCCB	85868	NM_1785	KY	1513434	intronic	MSL2	4.78882	Down	NULL	PBS_peak_7244
peak558	chr3	135890746	135890935	2	NM_0005	PCCB	78326	NM_1785	KY	1520976	intronic	MSL2	5.21193	Down	NULL	PBS_peak_7247
peak559	chr3	135891833	135891997	2	NM_0005	PCCB	77252	NM_1785	KY	1522051	intronic	MSL2	8.00878	Down	NULL	PBS_peak_7249
peak560	chr3	135894065	135894259	2	NM_0005	PCCB	75005	NM_1785	KY	1524298	intronic	MSL2	9.656	Down	NULL	PBS_peak_7252
peak561	chr3	135896717	135896910	2	NM_0005	PCCB	72353	NM_1785	KY	1526949	intronic	MSL2	5.56377	Down	NULL	PBS_peak_7255
peak562	chr3	137194998	137195208	0	NM_0041	SOX14	288031	NR_11017	NCK1-AS	614198	intergenic	IL20RB(d	10.32351	Down	NULL	PBS_peak_7263
peak563	chr3	138495920	138496183	0	NM_0010	FOXL2NE	170024	NM_0062	PIK3CB	17850	intergenic	PIK3CB(d	18.03117	Down	NULL	PBS_peak_7264
peak564	chr3	138510831	138511005	0	NM_0010	FOXL2NE	155158	NM_0062	PIK3CB	32717	intergenic	PIK3CB(d	15.07298	Down	NULL	NULL
peak565	chr3	138521951	138522106	0	NM_0010	FOXL2NE	144047	NM_0062	PIK3CB	43827	intergenic	PIK3CB(d	11.51382	Down	NULL	NULL
peak566	chr3	138524252	138524423	0	NM_0010	FOXL2NE	141738	NM_0062	PIK3CB	46136	intergenic	PIK3CB(d	27.63181	Down	NULL	PBS_peak_7268
peak567	chr3	138525057	138525225	0	NM_0010	FOXL2NE	140935	NM_0062	PIK3CB	46940	intergenic	PIK3CB(d	29.50319	Down	NULL	PBS_peak_7269
peak568	chr3	138545606	138545768	0	NM_0010	FOXL2NE	120389	NM_0062	PIK3CB	67486	intergenic	PIK3CB(d	6.63806	Down	NULL	PBS_peak_7276
peak569	chr3	138550149	138550319	0	NM_0010	FOXL2NE	115842	NM_0062	PIK3CB	72033	intergenic	PIK3CB(d	8.84625	Down	NULL	NULL
peak570	chr3	145651782	145651948	0	NM_0034	ZIC1	1475316	NM_1736	SLC9A9	2084492	intergenic	C3orf58(d	6.19963	Down	NULL	NULL
peak571	chr3	152087215	152087409	8	NM_0025	P2RY1	465170	NR_132656		28533	intronic	MBNL1	14.19068	Down	NULL	PBS_peak_7281
peak572	chr3	152090114	152090275	8	NM_0025	P2RY1	462287	NR_132656		31415	intronic	MBNL1	32.60538	Down	NULL	PBS_peak_7284
peak573	chr3	152113334	152113553	8	NM_0025	P2RY1	439038	NR_132656		54664	intronic	MBNL1	6.53768	Down	NULL	PBS_peak_7287
peak574	chr3	152117403	152117569	8	NM_0025	P2RY1	434996	NR_132656		58707	intronic	MBNL1	6.6278	Down	NULL	PBS_peak_7288
peak575	chr3	152162987	152163296	8	NM_0025	P2RY1	389340	NR_132656		104362	exonic	MBNL1	6.17671	Down	NULL	PBS_peak_7289
peak576	chr3	155599572	155599758	1	NM_0013	KCNAB1	238672	NM_0047	SLC33A1	27417	intronic	GMPS	15.64055	Down	NULL	PBS_peak_7291
peak577	chr3	162320326	162320555	0	NR_12570	LINC0132	2604307	NM_001320679		1229702	intergenic	OTOL1(di	8.86404	Down	NULL	PBS_peak_7299
peak578	chr3	165894618	165894771	0	NR_12635	LINC0132	1215889	NM_0000	BCHE	339441	intergenic	BCHE(dis	17.19131	Down	NULL	PBS_peak_7302
peak579	chr3	169331339	169331516	2	NM_0011	MYNN	159425	NM_0011	MECOM	465905	intronic	MECOM	4.21668	Down	NULL	PBS_peak_7312
peak580	chr3	176850048	176850198	1	NR_04740	LINC0050	162107	NR_1108	LINC0120	315334	intronic	TBL1XR1	4.18268	Down	NULL	PBS_peak_7327
peak581	chr3	176879423	176879607	1	NR_04740	LINC0050	132715	NR_1108	LINC0120	344726	intronic	TBL1XR1	3.89045	Down	NULL	PBS_peak_7329
peak582	chr3	176890793	176890953	1	NR_04740	LINC0050	121357	NR_1108	LINC0120	356084	intronic	TBL1XR1	4.99248	Down	NULL	NULL
peak583	chr3	176899794	176899960	1	NR_04740	LINC0050	112353	NR_1108	LINC0120	365088	intronic	TBL1XR1	3.83075	Down	NULL	PBS_peak_7334
peak584	chr3	176902883	176903101	1	NR_04740	LINC0050	109238	NR_1108	LINC0120	368203	intronic	TBL1XR1	8.34636	Down	NULL	PBS_peak_7337
peak585	chr3	179146773	179147049	1	NM_0043	ACTL6A	133757	NM_1718	KCNMB3	162073	intronic	GNB4	7.00972	Up	luks_peak	NULL
peak586	chr3	182163585	182163751	0	NM_0146	ATP11B	347623	NR_12540	LOC1027	1003394	downstrea	LINC0199	4.42581	Down	NULL	PBS_peak_7353
peak587	chr3	185646592	185646774	2	NR_03375	LOC34488	31075	NM_0065	IGF2BP2	103856	intronic	TRA2B	5.83298	Down	NULL	PBS_peak_7363
peak588	chr3	191989075	191989236	3	NM_0203	HRASLS	969761	NM_1981	UTS2B	940830	ncRNA_in	FGF12-AS	8.77945	Down	NULL	PBS_peak_7365
peak589	chr3	192979794	192979971	1	NR_04675	ATP13A5	45150	NM_1784	MB21D2	343932	intronic	HRASLS	6.67552	Down	NULL	PBS_peak_7368

peak590	chr3	193016923	193017077	1	NR_04675	ATP13A5	8033	NM_1784	MB21D2	381050	exonic	ATP13A5	6.6968	Down	NULL	NULL
peak591	chr3	193023586	193023763	1	NR_04675	ATP13A5	1358	NM_1784	MB21D2	387724	intronic	ATP13A5	5.83443	Down	NULL	PBS_peak_7370
peak592	chr3	193110345	193110561	0	NR_12166	ATP13A4	160549	NM_1985	ATP13A5	13939	intergenic	ATP13A5	4.67136	Down	NULL	PBS_peak_7372
peak593	chr3	195146979	195147139	1	NR_00326	SDHAP2	237851	NM_1525	XXYLT1	155164	intronic	ACAP2	28.21969	Down	NULL	PBS_peak_7381
peak594	chr3	195154745	195154931	1	NR_00326	SDHAP2	230072	NM_1525	XXYLT1	162943	intronic	ACAP2	4.00376	Down	NULL	PBS_peak_7383
peak595	chr4	919664	919901	2	NM_0012	TMEM175	6392	NM_0066	CPLX1	99837	intronic	GAK	3.66346	Down	NULL	PBS_peak_7397
peak596	chr4	1706796	1706982	3	NM_0063	TACC3	16328	NM_0011	FAM53A	20849	intronic	SLBP	12.02111	Down	NULL	PBS_peak_7401
peak597	chr4	1707340	1707533	3	NM_0063	TACC3	15780	NM_0011	FAM53A	21396	intronic	SLBP	9.61713	Down	NULL	PBS_peak_7402
peak598	chr4	1707700	1707950	3	NM_0063	TACC3	15392	NM_0011	FAM53A	21785	intronic	SLBP	5.80649	Down	NULL	PBS_peak_7402
peak599	chr4	2473566	2473760	3	NM_0012	FAM193A	153496	NM_0011	ZFYVE28	53293	intronic	RNF4	6.75486	Down	NULL	PBS_peak_7405
peak600	chr4	2480299	2480508	3	NM_0012	FAM193A	146755	NM_0011	ZFYVE28	60033	intronic	RNF4	4.8801	Down	NULL	PBS_peak_7411
peak601	chr4	2481482	2481652	3	NM_0012	FAM193A	145592	NM_0011	ZFYVE28	61197	intronic	RNF4	7.63866	Down	NULL	PBS_peak_7411
peak602	chr4	2485229	2485394	3	NM_0012	FAM193A	141847	NM_0011	ZFYVE28	64941	intronic	RNF4	4.07189	Down	NULL	PBS_peak_7414
peak603	chr4	2485890	2486122	3	NM_0012	FAM193A	141153	NM_0011	ZFYVE28	65636	intronic	RNF4	4.7713	Down	NULL	PBS_peak_7415
peak604	chr4	2489528	2489716	3	NM_0012	FAM193A	137537	NM_0011	ZFYVE28	69252	intronic	RNF4	4.80271	Down	NULL	PBS_peak_7418
peak605	chr4	2491228	2491418	3	NM_0012	FAM193A	135836	NM_0011	ZFYVE28	70953	intronic	RNF4	3.29076	Down	NULL	PBS_peak_7419
peak606	chr4	2543765	2543929	0	NM_0012	FAM193A	83312	NM_0011	ZFYVE28	123477	intergenic	RNF4(dist	9.05414	Down	NULL	PBS_peak_7427
peak607	chr4	2752941	2753099	3	NM_0011	SH3BP2	41730	NM_0011	ZFYVE28	332650	intronic	TNIP2	4.20635	Down	NULL	NULL
peak608	chr4	2809624	2809783	1	NM_0011	SH3BP2	4242	NM_0243	TNIP2	51600	intronic	SH3BP2	7.66319	Up	NULL	NULL
peak609	chr4	9628217	9628408	0	NM_0007	DRD5	154945	NR_03168	MIR548I2	70375	intergenic	MIR548I2	13.96213	Down	NULL	PBS_peak_7470
peak610	chr4	10960235	10960424	0	NR_03029	MIR572	410121	NM_0529	CLNK	273943	intergenic	CLNK(dis	26.9273	Down	NULL	PBS_peak_7502
peak611	chr4	17585331	17585499	1	NM_0252	MED28	30836	NM_0013	QDPR	71558	intronic	LAP3	3.83356	Up	luks_peak	NULL
peak612	chr4	17588191	17588342	1	NM_0252	MED28	27984	NM_0013	QDPR	74409	intronic	LAP3	3.86259	Up	luks_peak	NULL
peak613	chr4	17600778	17601112	1	NM_0252	MED28	15306	NM_0013	QDPR	87088	intronic	LAP3	4.82968	Up	luks_peak	NULL
peak614	chr4	17603742	17603912	1	NM_0252	MED28	12424	NM_0013	QDPR	89970	intronic	LAP3	5.73705	Up	NULL	NULL
peak615	chr4	17605303	17605483	1	NM_0252	MED28	10858	NM_0013	QDPR	91536	intronic	LAP3	4.45313	Up	luks_peak	NULL
peak616	chr4	17607981	17608161	1	NM_0252	MED28	8180	NM_0013	QDPR	94214	intronic	LAP3	13.30158	Up	NULL	NULL
peak617	chr4	24558028	24558178	1	NM_0031	SOD3	238982	NR_03029	MIR573	36190	exonic	DHX15	4.45609	Down	NULL	PBS_peak_7518
peak618	chr4	24567129	24567376	1	NM_0031	SOD3	229832	NR_03029	MIR573	45339	intronic	DHX15	11.92513	Down	NULL	PBS_peak_7524
peak619	chr4	26341110	26341299	4	NM_0012	TBC1D19	244341	NM_0012	SEL1L3	475987	intronic	RBPJ	8.04684	Down	NULL	PBS_peak_7544
peak620	chr4	26342171	26342329	4	NM_0012	TBC1D19	243296	NM_0012	SEL1L3	477033	intronic	RBPJ	6.11428	Down	NULL	PBS_peak_7545
peak621	chr4	26346651	26346835	4	NM_0012	TBC1D19	238803	NM_0012	SEL1L3	481526	intronic	RBPJ	8.75684	Down	NULL	PBS_peak_7548
peak622	chr4	26346971	26347199	4	NM_0012	TBC1D19	238461	NM_0012	SEL1L3	481868	intronic	RBPJ	8.66076	Down	NULL	PBS_peak_7548
peak623	chr4	26348718	26348868	4	NM_0012	TBC1D19	236753	NM_0012	SEL1L3	483576	intronic	RBPJ	5.18036	Down	NULL	PBS_peak_7550
peak624	chr4	26366452	26366621	4	NM_0012	TBC1D19	219009	NM_0012	SEL1L3	501319	intronic	RBPJ	5.73766	Down	NULL	PBS_peak_7559
peak625	chr4	26368792	26368968	4	NM_0012	TBC1D19	216666	NM_0012	SEL1L3	503663	intronic	RBPJ	5.34404	Down	NULL	NULL
peak626	chr4	26370393	26370608	4	NM_0012	TBC1D19	215045	NM_0012	SEL1L3	505283	intronic	RBPJ	7.13449	Down	NULL	PBS_peak_7562
peak627	chr4	26373268	26373428	4	NM_0012	TBC1D19	212198	NM_0012	SEL1L3	508131	intronic	RBPJ	15.37566	Down	NULL	PBS_peak_7563
peak628	chr4	26373797	26373956	4	NM_0012	TBC1D19	211669	NM_0012	SEL1L3	508659	intronic	RBPJ	4.3129	Down	NULL	PBS_peak_7563
peak629	chr4	26376334	26376553	4	NM_0012	TBC1D19	209102	NM_0012	SEL1L3	511226	intronic	RBPJ	7.05454	Down	NULL	PBS_peak_7565
peak630	chr4	26378923	26379104	4	NM_0012	TBC1D19	206532	NM_0012	SEL1L3	513796	intronic	RBPJ	7.32282	Down	NULL	PBS_peak_7568
peak631	chr4	26380818	26381038	4	NM_0012	TBC1D19	204618	NM_0012	SEL1L3	515711	intronic	RBPJ	5.21582	Down	NULL	PBS_peak_7569
peak632	chr4	39719986	39720180	6	NM_0013	18359	338441	NM_1749	SMIM14	79465	intronic	UBE2K	12.29653	Down	NULL	PBS_peak_7575
peak633	chr4	39975685	39975839	2	NM_0013	18359	82762	NM_1749	SMIM14	335144	intronic	PDS5A	10.58969	Down	NULL	PBS_peak_7594
peak634	chr4	49093571	49093746	0	NM_0010	DCUN1D4	3615507	NR_10458	OCIAD2	184813	intergenic	CWH43(d	3.67686	Down	NULL	PBS_peak_7603
peak635	chr4	49656354	49656577	0	NM_0010	DCUN1D4	3052700	NR_10458	OCIAD2	747620	intergenic	CWH43(d	4.84555	Down	NULL	PBS_peak_7615
peak636	chr4	59181734	59181885	0	NR_1339	41	668190	NM_0012	IGFBP7	1205258	intergenic	LINC0238	10.09794	Down	NULL	NULL
peak637	chr4	66323586	66323773	6	NR_03413	EPHA5-A	211999	NR_03397	LOC40111	453461	intronic	EPHA5	6.35515	Down	NULL	PBS_peak_7621
peak638	chr4	70319125	70319305	0	NM_0010	CSN1S1	477584	NM_0010	UGT2B11	238766	intergenic	UGT2B28	8.96582	Down	NULL	PBS_peak_7625
peak639	chr4	70327318	70327470	0	NM_0010	CSN1S1	469405	NM_0010	UGT2B11	246945	intergenic	UGT2B28	22.75573	Down	NULL	NULL
peak640	chr4	81842950	81843166	2	NM_0012	BMP3	109061	NM_0012	ANTXR2	848432	intronic	C4orf22	7.19893	Down	NULL	PBS_peak_7630
peak641	chr4	83289263	83289436	4	NM_0012	ENOPH1	62283	NM_1525	RASGEF1	896267	intronic	HNRNPD	4.5888	Down	NULL	PBS_peak_7637
peak642	chr4	83291527	83291704	4	NM_0012	ENOPH1	60017	NM_1525	RASGEF1	898533	intronic	HNRNPD	9.89604	Down	NULL	PBS_peak_7640
peak643	chr4	83347428	83347808	3	NM_0012	ENOPH1	4015	NM_0313	HNRNPD	52469	exonic	HNRNPD	4.36572	Down	NULL	PBS_peak_7641
peak644	chr4	92016685	92016862	1	NM_0012	GRID2	1208776	NM_0011	SNCA	1257326	intronic	CCSER1	4.59307	Down	NULL	PBS_peak_7645
peak645	chr4	94387364	94387542	2	NM_0051	ATOH1	362625	NR_12592	LOC10192	1189000	intronic	GRID2	7.88549	Down	NULL	PBS_peak_7646
peak646	chr4	108399737	108399899	0	NM_0011	SGMS2	345903	NM_0144	DKK2	442365	intergenic	DKK2(dis	3.50819	Up	NULL	NULL
peak647	chr4	110549951	110550110	1	NM_0189	GAR1	186635	NR_03997	SEC24B-A	195057	intronic	MCUB	23.21506	Up	NULL	NULL
peak648	chr4	126962185	126962350	0	NM_0156	INTU	1591819	NM_0011	ANKRD50	1328380	intergenic	MIR2054(	21.4413	Down	NULL	NULL
peak649	chr4	139954605	139954779	1	NM_0571	NAA15	267984	NR_1339	45	20892	intronic	NOCT	4.59408	Down	NULL	PBS_peak_7686
peak650	chr4	139955348	139955556	1	NM_0571	NAA15	267224	NR_1339	45	21652	intronic	NOCT	6.19305	Down	NULL	PBS_peak_7687
peak651	chr4	139957504	139957659	1	NM_0571	NAA15	265094	NR_1339	45	23781	intronic	NOCT	3.91812	Down	NULL	NULL
peak652	chr4	139959218	139959369	1	NM_0571	NAA15	263382	NR_1339	45	25493	intronic	NOCT	3.32515	Down	NULL	PBS_peak_7690
peak653	chr4	139966358	139966528	1	NM_0571	NAA15	256233	NR_1339	45	32643	exonic	NOCT	4.81458	Down	NULL	PBS_peak_7692
peak654	chr4	140236387	140236564	1	NM_0312	RAB33B	138485	NM_0011	NDUFC1	12770	intronic	NAA15	4.18343	Down	NULL	PBS_peak_7698
peak655	chr4	140244020	140244194	1	NM_0312	RAB33B	130854	NM_0011	NDUFC1	20402	intronic	NAA15	4.41584	Down	NULL	PBS_peak_7705
peak656	chr4	140251555	140251728	1	NM_0312	RAB33B	123319	NM_0011	NDUFC1	27936	intronic	NAA15	6.90386	Down	NULL	NULL
peak657	chr4	140253274	140253564	1	NM_0312	RAB33B	121542	NM_0011	NDUFC1	29714	intronic	NAA15	4.68808	Down	NULL	PBS_peak_7708
peak658	chr4	156027769	156027921	0	NM_0009	NPY2R	101936	NM_0005	FGG	493885	intergenic	RBM46(di	11.18808	Down	NULL	PBS_peak_7714
peak659	chr4	167241159	167241334	0	NM_0071	ANXA10	1772441	NR_12167	LOC10192	577023	intergenic	TLL1(dist	6.04372	Down	NULL	PBS_peak_7722
peak660	chr4	167817659	167817848	9	NM_0071	ANXA10	1195934	NR_12167	LOC10192	1153530	intronic	SPOCK3	12.09429	Down	NULL	PBS_peak_7723
peak661	chr4	169319374	169319549	2	NM_0011	PALLD	98755	NM_0176	DDX60	79503	intronic	DDX60L	17.9891	Up	NULL	NULL
peak662	chr4	169320257	169320566	2	NM_0011	PALLD	97805	NM_0176	DDX60	80453	intronic	DDX60L	21.19111	Up	luks_peak	NULL
peak663	chr4	186908732	186908919	0	NM_0032	TLR3	81483	NM_0210	SORBS2	30955	intergenic	SORBS2(	24.29106	Down	NULL	PBS_peak_7741
peak664	chr5	2379890	2380040	0	NM_0013	C5orf38	372280	NR_10461	LOC10050	67650	intergenic	LOC10050	3.698	Down	NULL	PBS_peak_7756
peak665	chr5	2416277	2416456	0	NM_0013	C5orf38	335878	NR_10461	LOC10050	104051	intergenic	LOC10050	4.68577	Down	NULL	NULL
peak666	chr5	6748232	6748475	3	NM_0205	ADCY2	647989	NR_10377	LOC10050	40529	intronic	PAPD7	5.24165	Up	luks_peak	NULL
peak667	chr5	6750521	6750811	3	NM_0205	ADCY2	645677	NR_10377	LOC10050	42842	exonic	PAPD7	15.97086	Up	luks_peak	NULL
peak668	chr5	6903172	6903358	0	NM_0205	ADCY2	493078	NR_03624	MIR4278	75231	intergenic	MIR4278(	5.47787	Down	NULL	PBS_peak_7782
peak669	chr5	7089666	7089833	0	NM_0205	ADCY2	306593	NR_03624	MIR4278	261715	intergenic	MIR4278(	12.8973	Down	NULL	PBS_peak_7786
peak670	chr5	7242841	7243008	0	NM_0205	ADCY2	153418	NR_03624	MIR4278	414890	intergenic	MIR4278(	6.19967	Down	NULL	NULL
peak671	chr5	7625723	7625898	1	NM_0024	MTRR	243406	NR_1342	77	277720	intronic	ADCY2	10.88761	Down	NULL	PBS_peak_7794
peak672	chr5	8662180	8662344	0	NR_10993	LOC10192	177582	NR_03998	LOC72950	204585	intergenic	MIR4458H	4.50623	Down	NULL	PBS_peak_7809
peak673	chr5	8671046	8671239	0	NR_10993	LOC10192	168701	NR_03998	LOC72950	213465	intergenic	MIR4458H	10.72108	Down	NULL	PBS_peak_7810
peak674	chr5	8726660	8726884	0	NR_10993	LOC10192	113072	NR_03998	LOC72950	269095	intergenic	MIR4458H	10.57176	Down	NULL	PBS_peak_7813
peak675	chr5	8789333	8789486	0	NR_10993	LOC10192	50434	NR_03998	LOC72950	331732	intergenic	MIR4458H	4.99122	Down	NULL	NULL
peak676	chr5	8920877	8921040	0	NR_10993	CTD-2201	590486	NR_03998	LOC72950	463281	intergenic	LINC0219	5.02018	Down	NULL	PBS_peak_7817
peak677	chr5	9024297	9024465	0	NR_10993	CTD-2201	487064	NR_03998	LOC72950	566704	intergenic	LINC0219	4.91806	Down	NULL	NULL
peak678	chr5	9257391	9257541	1	NR_10993	CTD-2201	253979	NR_03977	MIR4636	203459	intronic	SEMA5A	17.5612	Down	NULL	NULL
peak679	chr5	9731331	9731482	1	NM_0013	CCT5	518626	NM_0195	TAS2R1	100943	ncRNA_in	LINC0211	5.00477	Down	NULL	PBS_peak_7839
peak680	chr5	9965914	9966090	0	NM_0013	CCT5	284031	NR_02711	LOC28569	62066	intergenic	LINC0211	5.26296	Down	NULL	PBS_peak_7844
peak681	chr5	10253200	10253460	5	NM_0012	6-Mar	100421	NR_04767	FAM173B	3309	intronic	CCT5	9.30036	Down	NULL	PBS_peak_7848
peak682	chr5	11512882	11513035	5	NR_03338	LINC0119	1062010	NM_0043	DAP	751571	intronic	CTNND2	5.32586	Down	NULL	NULL
peak683	chr5	12049890	12050074	0	NR_03338	LINC0119	524987	NR_10998	CTNND2	145827	intergenic	CTNND2(	5.1369	Down	NULL	PBS_peak_7866
peak684	chr5	13397050	13397262	0	NM_0071	TRIO	746655	NR_10998	CTNND2	1493001	intergenic	LINC0222	3.8874	Down	NULL	PBS_peak_7902
peak685	chr5	13631567	13631734	0	NM_0071	TRIO	512160	NR_10998	CTNND2	1727495	intergenic	LINC0222	4.13325	Down	NULL	NULL
peak686	chr5	13666012	13666178	0	NM_0071	TRIO	477716	NR_10998	CTNND2	1761940	intergenic	LINC0222	4.59109	Down	NULL	NULL
peak687	chr5	13908472	13908642	1	NM_0071	TRIO	235254	NR_10998	CTNND2	2004402	intronic	DNAH5	18.171	Down	NULL	NULL
peak688	chr5	13962966	13963163	0	NM_0071	TRIO	180746	NM_0013	DNAH5	18475	intergenic	DNAH5(d	5.03249	Down	NULL	PBS_peak_7937
peak689	chr5	14667463	14667664	1	NR_04628	LOC10013	45239	NR_1329	81	15072	intronic	OTULIN	5.46971	Down	NULL	PBS_peak_7948
peak690	chr5	14671608	14671767	1	NR_04628	LOC10013	41115	NR_1329	81	19196	intronic	OTULIN	4.23909	Down	NULL	NULL
peak691	chr5	15279954	15280153	0	NM_0123	FBXL7	220251	NR_10994	LOC10192	13384	intergenic	LINC0214	5.49274	Down	NULL	PBS_peak_7966
peak692	chr5	16005956	16006132	0	NR_10994	LOC10192	609991	NR_10994	CTD-2350	390933	intergenic	FBXL7(di	3.90331	Down	NULL	PBS_peak_8003

peak693	chr5	16072519	16072670	1	NR_10994	LOC10192	543440	NR_10994	CTD-2350	457483	intronic	11-Mar	4.06807	Down	NULL	PBS_peak_8013
peak694	chr5	16092654	16092822	1	NR_10994	LOC10192	523297	NR_10994	CTD-2350	477627	intronic	11-Mar	7.74179	Down	NULL	NULL
peak695	chr5	16461125	16461286	1	NR_10994	LOC10192	154829	NR_10462	LOC10192	19994	intronic	ZNF622	5.8712	Down	NULL	NULL
peak696	chr5	16829204	16829390	1	NM_0012	BASP1	387635	NM_0010	FAM134B	212130	intronic	MYO10	4.80686	Up	NULL	NULL
peak697	chr5	16893943	16894097	1	NM_0012	BASP1	322912	NM_0010	FAM134B	276853	intronic	MYO10	5.06528	Up	NULL	NULL
peak698	chr5	17228702	17228886	2	NR_134275		175334	NR_02725	LOC28569	11263	intronic	BASP1	4.54978	Down	NULL	PBS_peak_8023
peak699	chr5	28122733	28122915	0	NR_03396	LSP1P3	804153	NM_0162	CDH9	1084135	intergenic	LINC0102	8.95632	Down	NULL	NULL
peak700	chr5	29764813	29764984	0	NR_134264		115768	NR_10462	LOC10192	368815	intergenic	LINC0206	7.00915	Down	NULL	PBS_peak_8039
peak701	chr5	29967266	29967423	0	NM_0049	CDH6	1226417	NR_10462	LOC10192	571261	intergenic	LOC10537	3.82859	Down	NULL	PBS_peak_8040
peak702	chr5	32162791	32163005	1	NM_0067	SUB1	422707	NR_03624	MIR4279	226633	intronic	GOLPH3	6.5398	Down	NULL	PBS_peak_8050
peak703	chr5	32163286	32163451	1	NM_0067	SUB1	422236	NR_03624	MIR4279	227103	intronic	GOLPH3	7.55349	Down	NULL	NULL
peak704	chr5	32165337	32165510	1	NM_0067	SUB1	420181	NR_03624	MIR4279	229158	intronic	GOLPH3	5.46786	Down	NULL	NULL
peak705	chr5	50598799	50599046	0	NM_0022	ISL1	80035	NR_10465	LOC10028	332901	intergenic	LINC0210	8.75204	Down	NULL	PBS_peak_8058
peak706	chr5	56148019	56148170	1	NM_1537	SETD9	56992	NM_0012	C5orf67	246035	intronic	MAP3K1	4.47688	Down	NULL	PBS_peak_8062
peak707	chr5	60686612	60686937	1	NM_1736	C5orf64	246833	NM_0010	SMIM15	228472	intronic	ZSWIM6	6.92599	Up	luks_peak	NULL
peak708	chr5	60695553	60695747	1	NM_1736	C5orf64	237958	NM_0010	SMIM15	237348	intronic	ZSWIM6	5.2081	Up	NULL	NULL
peak709	chr5	65272062	65272265	6	NM_0012	SREK1	167882	NR_02847	LOC10030	30762	intronic	ERBIN	4.21539	Up	luks_peak	NULL
peak710	chr5	128069418	128069644	0	NM_0010	SLC27A6	231289	NM_0019	FBN2	195796	intergenic	FBN2(dist	9.52452	Down	NULL	PBS_peak_8088
peak711	chr5	131819907	131820058	1	NM_0057	RAD50	72633	NR_11099	LOC55310	114374	exonic	IRF1	5.09066	Up	NULL	NULL
peak712	chr5	131822331	131822539	1	NM_0057	RAD50	70181	NR_11099	LOC55310	116827	exonic	IRF1	22.40814	Up	luks_peak	NULL
peak713	chr5	132284130	132284358	1	NM_0021	HSPA4	103418	NM_0012	GDF9	81668	intronic	AFF4	6.41475	Up	luks_peak	NULL
peak714	chr5	136335287	136335460	1	NR_134246		128512	NM_0203	TRPC7	634209	intronic	SPOCK1	10.1507	Down	NULL	PBS_peak_8144
peak715	chr5	137344161	137344313	3	NR_037900		24226	NM_0068	HNRNPA	254198	intronic	FAM13B	21.58546	Down	NULL	NULL
peak716	chr5	137861997	137862163	5	NM_0012	CTNNA1	227005	NM_001318098		188036	intronic	ETF1	5.44062	Down	NULL	PBS_peak_8169
peak717	chr5	138636539	138636697	6	NM_0164	PAIP2	40903	NM_0224	SIL1	102553	intronic	MATR3	4.08676	Down	NULL	PBS_peak_8184
peak718	chr5	138637749	138638022	6	NM_0164	PAIP2	39635	NM_0224	SIL1	103820	intronic	MATR3	6.20881	Down	NULL	PBS_peak_8186
peak719	chr5	138645376	138645534	6	NM_0164	PAIP2	32066	NM_0224	SIL1	111390	intronic	MATR3	4.97422	Down	NULL	PBS_peak_8192
peak720	chr5	138652653	138652839	6	NM_0164	PAIP2	24775	NM_0224	SIL1	118681	exonic	MATR3	4.6996	Down	NULL	PBS_peak_8195
peak721	chr5	138952632	138952799	2	NM_001317201		74168	NM_1982	TMEM17	90340	intronic	UBE2D2	5.00473	Down	NULL	PBS_peak_8203
peak722	chr5	138954287	138954470	2	NM_001317201		72505	NM_1982	TMEM17	92003	intronic	UBE2D2	4.39635	Down	NULL	PBS_peak_8204
peak723	chr5	145832778	145832954	2	NM_1450	STK32A	781713	NM_0201	LARS	270516	intronic	TCERG1	4.32195	Down	NULL	PBS_peak_8225
peak724	chr5	149120948	149121105	2	NM_0011	PPARGC1	30476	NM_0018	CSNK1A1	189911	intronic	PPARGC1	6.44323	Down	NULL	NULL
peak725	chr5	149121601	149121807	2	NM_0011	PPARGC1	29799	NM_0018	CSNK1A1	190589	intronic	PPARGC1	17.49422	Down	NULL	PBS_peak_8253
peak726	chr5	149123733	149124030	2	NM_0011	PPARGC1	27621	NM_0018	CSNK1A1	192766	intronic	PPARGC1	12.53751	Down	NULL	PBS_peak_8255
peak727	chr5	149124231	149124448	2	NM_0011	PPARGC1	27163	NM_0018	CSNK1A1	193224	intronic	PPARGC1	19.34382	Down	NULL	PBS_peak_8256
peak728	chr5	149124997	149125172	2	NM_0011	PPARGC1	26418	NM_0018	CSNK1A1	193969	intronic	PPARGC1	12.44445	Down	NULL	PBS_peak_8256
peak729	chr5	149128614	149128784	2	NM_0011	PPARGC1	22804	NM_0018	CSNK1A1	197584	intronic	PPARGC1	6.12803	Down	NULL	PBS_peak_8258
peak730	chr5	149129633	149129789	2	NM_0011	PPARGC1	21792	NM_0018	CSNK1A1	198596	intronic	PPARGC1	18.42168	Down	NULL	NULL
peak731	chr5	149136105	149136287	2	NM_0011	PPARGC1	15307	NM_0018	CSNK1A1	205081	intronic	PPARGC1	7.42987	Down	NULL	PBS_peak_8260
peak732	chr5	149136505	149136795	2	NM_0011	PPARGC1	14853	NM_0018	CSNK1A1	205535	intronic	PPARGC1	8.49475	Down	NULL	PBS_peak_8260
peak733	chr5	149143780	149143945	2	NM_0011	PPARGC1	7640	NM_0018	CSNK1A1	212747	intronic	PPARGC1	7.9293	Down	NULL	PBS_peak_8261
peak734	chr5	149151603	149151785	3	NM_0001	SLC26A2	188606	NM_0018	CSNK1A1	220579	exonic	PPARGC1	18.16153	Down	NULL	PBS_peak_8263
peak735	chr5	149159682	149159871	3	NM_0001	SLC26A2	180523	NM_0018	CSNK1A1	228661	intronic	PPARGC1	14.35109	Down	NULL	PBS_peak_8264
peak736	chr5	149160352	149160553	3	NM_0001	SLC26A2	179847	NM_0018	CSNK1A1	229337	intronic	PPARGC1	6.43605	Down	NULL	PBS_peak_8265
peak737	chr5	149174186	149174374	3	NM_0001	SLC26A2	166020	NM_0018	CSNK1A1	243165	intronic	PPARGC1	25.3475	Down	NULL	PBS_peak_8267
peak738	chr5	154149342	154149719	1	NR_03966	MIR378H	59487	NR_03609	MIR3141	173898	intronic	LARP1	3.44202	Down	NULL	PBS_peak_8318
peak739	chr5	154155327	154155561	1	NR_03966	MIR378H	53574	NR_03609	MIR3141	179812	intronic	LARP1	5.68923	Down	NULL	PBS_peak_8326
peak740	chr5	154160026	154160353	1	NR_03966	MIR378H	48828	NR_03609	MIR3141	184557	intronic	LARP1	9.37138	Down	NULL	PBS_peak_8331
peak741	chr5	154163484	154163926	1	NR_03966	MIR378H	45313	NR_03609	MIR3141	188073	intronic	LARP1	9.19188	Down	NULL	PBS_peak_8335
peak742	chr5	154164705	154164868	1	NR_03966	MIR378H	44231	NR_03609	MIR3141	189154	intronic	LARP1	4.10364	Down	NULL	PBS_peak_8336
peak743	chr5	154166556	154166745	1	NR_03966	MIR378H	42367	NR_03609	MIR3141	191018	intronic	LARP1	6.14734	Down	NULL	PBS_peak_8338
peak744	chr5	157261181	157261360	3	NR_10989	LOC10192	1266220	NM_0013	SOX30	162782	intronic	CLINT1	20.04163	Down	NULL	PBS_peak_8361
peak745	chr5	159904531	159904682	1	NR_02970	MIR146A	7752	NM_0064	SLU7	58438	ncRNA_in	MIR3142I	16.68252	Up	NULL	NULL
peak746	chr5	159911567	159911748	1	NR_02970	MIR146A	701	NM_0064	SLU7	65489	ncRNA_in	MIR3142I	9.01989	Up	NULL	NULL
peak747	chr5	167445884	167446062	2	NM_0011	WWC1	273092	NR_134280		850567	intronic	TENM2	5.12942	Down	NULL	PBS_peak_8368
peak748	chr5	168187112	168187290	2	NR_10989	LOC72809	253031	NM_0245	PANK3	180587	intronic	SLIT3	7.34463	Down	NULL	PBS_peak_8370
peak749	chr5	168472636	168472824	2	NM_0177	SPDL1	537908	NR_02963	MIR218-2	277470	intronic	SLIT3	4.88785	Down	NULL	PBS_peak_8376
peak750	chr5	168779949	168780136	0	NM_0177	SPDL1	230595	NM_0030	SLIT3	51909	intergenic	SLIT3(dis	7.49933	Down	NULL	PBS_peak_8383
peak751	chr5	170821114	170821478	3	NM_0038	FGF18	25371	NR_03747	MIR3912	7532	intronic	NPM1	7.30932	Down	NULL	PBS_peak_8394
peak752	chr5	170834458	170834626	2	NM_0038	FGF18	12125	NR_03747	MIR3912	20778	intronic	NPM1	4.79784	Down	NULL	PBS_peak_8402
peak753	chr5	170838145	170838321	0	NM_0038	FGF18	8434	NR_03747	MIR3912	24469	downstrea	NPM1	13.4433	Down	NULL	PBS_peak_8404
peak754	chr5	170839691	170839842	0	NM_0038	FGF18	6900	NR_03747	MIR3912	26002	downstrea	NPM1	3.55231	Down	NULL	NULL
peak755	chr5	171594635	171594805	1	NM_0011	EFCAB9	26456	NM_0336	FBXW11	160843	intronic	STK10	4.2968	Up	luks_peak	NULL
peak756	chr5	174610892	174611073	0	NM_0227	SFXN1	294531	NR_04611	FLJ16171	188248	intergenic	LINC0195	5.98982	Down	NULL	PBS_peak_8420
peak757	chr5	179047484	179047650	2	NR_134260		37196	NR_11056	LOC10192	16825	intronic	HNRNPH	9.14529	Down	NULL	NULL
peak758	chr6	34309578	34309751	2	NM_0208	PACSIN1	124173	NM_1785	C6orf1	92779	exonic	NUDT3,R	9.72867	Down	NULL	NULL
peak759	chr6	34316957	34317338	2	NM_0208	PACSIN1	116690	NM_1785	C6orf1	100262	intronic	NUDT3,R	7.39053	Down	NULL	PBS_peak_8444
peak760	chr6	34323721	34323997	2	NM_0208	PACSIN1	109979	NM_1785	C6orf1	106974	intronic	NUDT3,R	10.14672	Down	NULL	PBS_peak_8445
peak761	chr6	34327603	34327792	2	NM_0208	PACSIN1	106140	NM_1785	C6orf1	110812	intronic	NUDT3,R	4.39902	Down	NULL	PBS_peak_8446
peak762	chr6	34331318	34331472	2	NM_0208	PACSIN1	102443	NM_1785	C6orf1	114510	intronic	NUDT3,R	31.77113	Down	NULL	NULL
peak763	chr6	34331665	34331854	2	NM_0208	PACSIN1	102078	NM_1785	C6orf1	114874	intronic	NUDT3,R	5.52487	Down	NULL	PBS_peak_8451
peak764	chr6	34332907	34333087	2	NM_0208	PACSIN1	100841	NM_1785	C6orf1	116112	intronic	NUDT3,R	5.53834	Down	NULL	PBS_peak_8452
peak765	chr6	34333385	34333574	2	NM_0208	PACSIN1	100358	NM_1785	C6orf1	116594	intronic	NUDT3,R	5.05739	Down	NULL	PBS_peak_8453
peak766	chr6	34334687	34334860	2	NM_0208	PACSIN1	99064	NM_1785	C6orf1	117888	intronic	NUDT3,R	5.00713	Down	NULL	PBS_peak_8454
peak767	chr6	34340280	34340521	2	NM_0208	PACSIN1	93437	NM_1785	C6orf1	123515	intronic	NUDT3,R	5.50322	Down	NULL	PBS_peak_8456
peak768	chr6	34344618	34344772	2	NM_0208	PACSIN1	89143	NM_1785	C6orf1	127810	intronic	NUDT3,R	4.08535	Down	NULL	PBS_peak_8458
peak769	chr6	34346429	34346700	2	NM_0208	PACSIN1	87273	NM_1785	C6orf1	129679	intronic	NUDT3,R	5.86417	Down	NULL	PBS_peak_8459
peak770	chr6	34347943	34348256	2	NM_0208	PACSIN1	85738	NM_1785	C6orf1	131214	intronic	NUDT3,R	10.96083	Down	NULL	PBS_peak_8461
peak771	chr6	34348574	34348733	2	NM_0208	PACSIN1	85184	NM_1785	C6orf1	131768	intronic	NUDT3,R	3.72546	Down	NULL	PBS_peak_8461
peak772	chr6	34349654	34349824	2	NM_0208	PACSIN1	84099	NM_1785	C6orf1	132854	intronic	NUDT3,R	4.55201	Down	NULL	PBS_peak_8462
peak773	chr6	34352620	34352812	2	NM_0208	PACSIN1	81122	NM_1785	C6orf1	135831	intronic	NUDT3,R	6.74355	Down	NULL	PBS_peak_8466
peak774	chr6	34353813	34354048	2	NM_0208	PACSIN1	79907	NM_1785	C6orf1	137045	intronic	NUDT3,R	6.0357	Down	NULL	PBS_peak_8467
peak775	chr6	36001802	36002159	4	NM_0027	MAPK13	96280	NM_0529	SLC26A8	9567	intronic	MAPK14	14.20409	Down	NULL	PBS_peak_8472
peak776	chr6	36002252	36002460	4	NM_0027	MAPK13	95905	NM_0529	SLC26A8	9943	intronic	MAPK14	5.24418	Down	NULL	PBS_peak_8472
peak777	chr6	36003023	36003269	4	NM_0027	MAPK13	95115	NM_0529	SLC26A8	10733	intronic	MAPK14	8.16658	Down	NULL	PBS_peak_8473
peak778	chr6	36005244	36005559	4	NM_0027	MAPK13	92859	NM_0529	SLC26A8	12988	intronic	MAPK14	4.66356	Down	NULL	PBS_peak_8475
peak779	chr6	36005645	36005818	4	NM_0027	MAPK13	92529	NM_0529	SLC26A8	13318	intronic	MAPK14	4.922	Down	NULL	PBS_peak_8475
peak780	chr6	36005910	36006643	4	NM_0027	MAPK13	91984	NM_0529	SLC26A8	13863	intronic	MAPK14	9.5111	Down	NULL	PBS_peak_8475
peak781	chr6	36007548	36007808	4	NM_0027	MAPK13	90583	NM_0529	SLC26A8	15265	intronic	MAPK14	5.67686	Down	NULL	PBS_peak_8476
peak782	chr6	36008266	36008578	4	NM_0027	MAPK13	89839	NM_0529	SLC26A8	16009	intronic	MAPK14	8.46617	Down	NULL	PBS_peak_8477
peak783	chr6	36010386	36010564	4	NM_0027	MAPK13	87786	NM_0529	SLC26A8	18062	intronic	MAPK14	7.12262	Down	NULL	PBS_peak_8478
peak784	chr6	36012647	36012851	4	NM_0027	MAPK13	85512	NM_0529	SLC26A8	20336	intronic	MAPK14	4.54744	Down	NULL	PBS_peak_8480
peak785	chr6	36018338	36018513	4	NM_0027	MAPK13	79835	NM_0529	SLC26A8	26012	intronic	MAPK14	6.68558	Down	NULL	PBS_peak_8482
peak786	chr6	36033897	36034066	4	NM_0027	MAPK13	64279	NM_0529	SLC26A8	41568	intronic	MAPK14	31.84516	Down	NULL	PBS_peak_8484
peak787	chr6	36035024	36035318	4	NM_0027	MAPK13	63090	NM_0529	SLC26A8	42758	intronic	MAPK14	12.91661	Down	NULL	PBS_peak_8485
peak788	chr6	36572472	36572673	0	NM_0012	CDKN1A	71664	NM_0072	STK38	57279	downstrea	SRSF3	6.56551	Down	NULL	PBS_peak_8491
peak789	chr6	37944037	37944201	1	NM_0012	DNAH8	738998	NM_1534	MDGA1	278353	intronic	ZFAND3	6.09363	Up	NULL	NULL
peak790	chr6	37981493	37981662	1	NM_0012	DNAH8	701539	NM_1534	MDGA1	315811	intronic	ZFAND3	8.72882	Up	NULL	NULL
peak791	chr6	39458052	39458236	3	NM_0012	DAAM2	302015	NM_0012	KIF6	58994	intronic	KIF6	4.1662	Down	NULL	PBS_peak_8533
peak792	chr6	39590274	39590435	3	NM_0012	DAAM2	169804	NM_0012	KIF6	191204	intronic	KIF6	4.65274	Down	NULL	NULL
peak793	chr6	39676197	39676362	3	NM_0012	DAAM2	83879	NM_0012	KIF6	277129	intronic	KIF6	3.71166	Down	NULL	PBS_peak_8538
peak794	chr6	40064821	40065023	0	NR_02401	TDRG1	281241	NR_03323	MOCS1	162632	intergenic	MOCS1(d	10.57885	Down	NULL	PBS_peak_8554
peak795	chr6	41893807	41894054	1	NM_1385	TAF8	124320	NR_13116	MED20	5045	intronic	BYSL	13.25713	Down	NULL	PBS_peak_8566

peak796	chr6	44086204	44086368	6	NM_001318792		8365	NR_12586	LOC10192	43897	intronic	MRPL14	4.06298	Down	NULL	PBS_peak_8569
peak797	chr6	44088042	44088229	6	NM_001318792		6515	NR_12586	LOC10192	45746	intronic	MRPL14	3.87181	Down	NULL	PBS_peak_8571
peak798	chr6	44089183	44089334	6	NM_001318792		5392	NR_12586	LOC10192	46869	intronic	MRPL14	3.71207	Down	NULL	PBS_peak_8572
peak799	chr6	44219816	44219986	5	NM_0011	TMEM151	18579	NM_0321	MRPL14	124673	exonic	HSP90AB	6.04021	Down	NULL	PBS_peak_8580
peak800	chr6	50324923	50325081	0	NM_1722	TFAP2D	356255	NM_0010	DEFB112	308638	intergenic	DEFB112(	6.13503	Down	NULL	PBS_peak_8593
peak801	chr6	50451975	50452128	0	NM_1722	TFAP2D	229205	NM_0010	DEFB112	435687	intergenic	DEFB112(	5.62228	Down	NULL	PBS_peak_8598
peak802	chr6	55237284	55237476	1	NR_12586	LOC10193	1471404	NM_0010	KLHL31	1706874	intronic	GFRAL	4.00627	Down	NULL	PBS_peak_8647
peak803	chr6	63658865	63659038	0	NM_0034	PTP4A1	622965	NM_1526	KHDRBS2	662819	intergenic	KHDRBS2	5.12742	Down	NULL	NULL
peak804	chr6	71627987	71628176	1	NM_0245	OGFRL1	370395	NM_0018	COL9A1	615295	intronic	B3GAT2	15.87011	Down	NULL	PBS_peak_8711
peak805	chr6	73858481	73858647	5	NM_0010	KHDC3L	213836	NR_04662	KCNQ5-A	5327	intronic	KCNQ5	6.39433	Down	NULL	PBS_peak_8721
peak806	chr6	77911405	77911570	0	NM_0012	MEI4	488885	NM_0015	IMPG1	1129092	intergenic	IMPG1(di	16.59179	Down	NULL	PBS_peak_8750
peak807	chr6	78848936	78849101	0	NM_0010	IRAK1BP	728242	NM_0008	HTR1B	675279	intergenic	MEI4(dist	4.47095	Down	NULL	PBS_peak_8762
peak808	chr6	80971654	80971818	4	NM_0066	TPBG	2101187	NM_0227	ELOVL4	314421	intronic	BCKDHB	3.68088	Down	NULL	PBS_peak_8763
peak809	chr6	83732107	83732290	2	NM_0011	DOPEY1	45186	NM_0155	IBTK	774727	exonic	UBE3D	4.61386	Down	NULL	PBS_peak_8819
peak810	chr6	85855945	85856096	0	NM_0012	NT5E	303281	NM_0010	TBX18	382066	intergenic	TBX18(di	5.65514	Down	NULL	NULL
peak811	chr6	86335213	86335410	7	NM_0008	HTR1E	1311712	NM_1538	SNX14	31437	intronic	SYNCRIP	3.92631	Down	NULL	PBS_peak_8823
peak812	chr6	86340258	86340442	7	NM_0008	HTR1E	1306674	NM_1538	SNX14	36476	intronic	SYNCRIP	3.57113	Down	NULL	PBS_peak_8826
peak813	chr6	86344682	86344843	7	NM_0008	HTR1E	1302261	NM_1538	SNX14	40888	intronic	SYNCRIP	3.56015	Down	NULL	PBS_peak_8831
peak814	chr6	86345208	86345700	7	NM_0008	HTR1E	1301570	NM_1538	SNX14	41580	intronic	SYNCRIP	5.68748	Down	NULL	PBS_peak_8832
peak815	chr6	87435216	87435404	0	NM_0008	HTR1E	211714	NR_00303	SNHG5	1046859	intergenic	SNHG5(d	5.71133	Down	NULL	PBS_peak_8841
peak816	chr6	90524402	90524588	1	NM_0011	CASP8AP	15124	NM_0204	LYRM2	176021	intronic	MDN1	9.2787	Down	NULL	PBS_peak_8851
peak817	chr6	92055226	92055381	0	NR_03978	MIR4643	176074	NM_1453	MAP3K7	758283	intergenic	MAP3K7(	5.87045	Down	NULL	PBS_peak_8856
peak818	chr6	93804209	93804385	0	NR_01536	TSG1	612504	NR_10415	CASC6	1404151	intergenic	CASC6(di	7.54021	Down	NULL	NULL
peak819	chr6	94036690	94036846	2	NR_01536	TSG1	380033	NR_10415	CASC6	1636622	intronic	EPHA7	13.43735	Down	NULL	NULL
peak820	chr6	96188617	96188794	0	NM_0065	FUT9	275139	NR_04750	MANEA-A	163378	intergenic	MANEA(d	4.96693	Down	NULL	PBS_peak_8881
peak821	chr6	96389921	96390091	0	NM_0065	FUT9	73839	NR_04750	MANEA-A	364679	intergenic	MANEA(d	4.35342	Down	NULL	PBS_peak_8886
peak822	chr6	96638551	96638703	1	NM_0153	UFL1	331075	NR_04750	MANEA-A	613300	intronic	FUT9	4.74626	Down	NULL	PBS_peak_8894
peak823	chr6	98226776	98226932	0	NR_03157	MIR2113	245553	NR_03167	MIR548H	364571	intergenic	LOC10192	10.02678	Down	NULL	NULL
peak824	chr6	98245616	98245770	0	NR_03157	MIR2113	226714	NR_03167	MIR548H	383410	intergenic	LOC10192	4.48896	Down	NULL	PBS_peak_8923
peak825	chr6	98356192	98356374	0	NR_03157	MIR2113	116124	NR_03167	MIR548H	494000	intergenic	LOC10192	3.83967	Down	NULL	NULL
peak826	chr6	98708682	98708851	0	NM_0056	POU3F2	573813	NR_03167	MIR548H	846483	intergenic	MIR2113(	6.5839	Down	NULL	PBS_peak_8937
peak827	chr6	99124116	99124269	0	NM_0056	POU3F2	158387	NR_03167	MIR548H	1261909	intergenic	MIR2113(	4.56739	Down	NULL	NULL
peak828	chr6	101808701	101808860	0	NM_0011	GRIK2	38080	NM_0220	ASCC3	479532	intergenic	ASCC3(di	4.88212	Down	NULL	PBS_peak_8987
peak829	chr6	108265752	108265924	1	NM_0032	NR2E1	221424	NM_1980	SCML4	120317	intronic	SEC63	3.77294	Down	NULL	PBS_peak_9014
peak830	chr6	108268880	108269044	1	NM_0032	NR2E1	218300	NM_1980	SCML4	123441	intronic	SEC63	4.14575	Down	NULL	PBS_peak_9016
peak831	chr6	108965617	108965785	2	NR_03337	LINC0022	107156	NM_1528	SNX3	383237	intronic	FOXO3	19.92362	Up	NULL	NULL
peak832	chr6	111202276	111202636	5	NM_1384	GTF3C6	77307	NM_0013	CDK19	65368	intronic	AMD1	5.04968	Down	NULL	PBS_peak_9025
peak833	chr6	111204986	111205177	5	NM_1384	GTF3C6	74681	NM_0013	CDK19	67993	intronic	AMD1	4.61411	Down	NULL	PBS_peak_9029
peak834	chr6	111209481	111209688	5	NM_1384	GTF3C6	70178	NM_0013	CDK19	72496	intronic	AMD1	3.79897	Down	NULL	PBS_peak_9033
peak835	chr6	114286797	114287063	3	NR_12584	LOC10192	3935	NR_03886	LINC0126	92418	intronic	HDAC2	8.985	Down	NULL	PBS_peak_9037
peak836	chr6	114912434	114912584	0	NR_02733	TPI1P3	1447385	NM_1536	HS3ST5	528468	intergenic	HDAC2-A	19.04637	Down	NULL	NULL
peak837	chr6	116137382	116137545	0	NR_02733	TPI1P3	222430	NR_134602		172464	intergenic	LOC10537	6.11628	Down	NULL	PBS_peak_9062
peak838	chr6	116240087	116240270	0	NR_02733	TPI1P3	119715	NR_134602		275179	intergenic	LOC10537	28.3376	Down	NULL	PBS_peak_9063
peak839	chr6	120368575	120368726	0	NM_0001	GJA1	1388072	NR_04610	LOC28576	556183	intergenic	MIR3144(	4.03038	Down	NULL	NULL
peak840	chr6	120865932	120866121	0	NM_0001	GJA1	890696	NR_04610	LOC28576	1053559	intergenic	MIR3144(	8.14497	Down	NULL	PBS_peak_9089
peak841	chr6	121513927	121514107	2	NM_0001	GJA1	242706	NR_04610	LOC28576	1701550	intronic	TBC1D32	5.18184	Down	NULL	PBS_peak_9105
peak842	chr6	123994673	123994841	0	NM_0010	NKAIN2	130234	NM_0012	TRDN	36519	intergenic	TRDN(dis	3.86098	Down	NULL	PBS_peak_9136
peak843	chr6	124149307	124149488	4	NM_0013	NKAIN2	454753	NM_0012	TRDN	191159	intronic	NKAIN2	12.27096	Down	NULL	PBS_peak_9143
peak844	chr6	124404926	124405105	4	NM_0013	NKAIN2	199135	NM_0012	TRDN	446777	intronic	NKAIN2	4.1619	Down	NULL	PBS_peak_9161
peak845	chr6	124447488	124447673	4	NM_0013	NKAIN2	156570	NM_0012	TRDN	489342	intronic	NKAIN2	4.20006	Down	NULL	PBS_peak_9164
peak846	chr6	124528998	124529183	4	NM_0013	NKAIN2	75060	NM_0012	TRDN	570852	intronic	NKAIN2	21.14731	Down	NULL	PBS_peak_9167
peak847	chr6	124665975	124666161	5	NM_0012	RNF217	617623	NM_0012	TRDN	707830	intronic	NKAIN2	9.51344	Down	NULL	PBS_peak_9175
peak848	chr6	124706244	124706408	5	NM_0012	RNF217	577365	NM_0012	TRDN	748088	intronic	NKAIN2	3.9941	Down	NULL	PBS_peak_9176
peak849	chr6	126245867	126246034	6	NM_1385	HINT3	31910	NR_12638	NCOA7-A	105946	intronic	NCOA7	4.37238	Up	NULL	NULL
peak850	chr6	126840830	126841012	0	NM_0327	RSPO3	599127	NR_04988	MIR5695	397159	intergenic	MIR588(d	4.92645	Down	NULL	PBS_peak_9200
peak851	chr6	126916222	126916378	0	NM_0327	RSPO3	523748	NR_04988	MIR5695	472538	intergenic	MIR588(d	4.76949	Down	NULL	PBS_peak_9202
peak852	chr6	129314164	129314316	2	NM_0010	L3MBTL3	1025488	NM_0012	PTPRK	472421	intronic	LAMA2	4.77954	Down	NULL	NULL
peak853	chr6	132931821	132931975	0	NM_0010	RPS12	203810	NR_02851	TAAR3	1457	upstream	TAAR3P	4.45171	Down	NULL	PBS_peak_9231
peak854	chr6	133321357	133321516	0	NR_02696	LINC0032	87782	NM_0528	SLC18B1	201689	intergenic	RPS12(dis	3.67408	Down	NULL	NULL
peak855	chr6	133446021	133446217	0	NM_0041	EYA4	116376	NM_0528	SLC18B1	326372	intergenic	LINC0032	5.3036	Down	NULL	PBS_peak_9262
peak856	chr6	133543438	133543596	0	NM_0041	EYA4	18978	NM_0528	SLC18B1	423770	intergenic	LINC0032	4.48237	Down	NULL	PBS_peak_9271
peak857	chr6	134041056	134041227	1	NR_02703	LINC0131	101143	NM_0528	SLC18B1	921394	ncRNA_in	TARID	7.49325	Down	NULL	NULL
peak858	chr6	134049418	134049615	1	NR_02703	LINC0131	92768	NM_0528	SLC18B1	929769	ncRNA_in	TARID	7.30534	Down	NULL	PBS_peak_9319
peak859	chr6	134090747	134090906	1	NR_02703	LINC0131	51458	NM_0528	SLC18B1	971079	ncRNA_in	TARID	4.99551	Down	NULL	PBS_peak_9323
peak860	chr6	139291734	139291899	4	NM_0212	ABRACL	58002	NM_0012	LOC10050	197000	intronic	REPS1	4.33029	Down	NULL	PBS_peak_9334
peak861	chr6	144490315	144490535	1	NM_0071	UTRN	122448	NM_0312	SF3B5	73671	intronic	STX11	15.64674	Up	luks_peak	NULL
peak862	chr6	144490933	144491092	1	NM_0071	UTRN	121860	NM_0312	SF3B5	74258	intronic	STX11	7.30373	Up	NULL	NULL
peak863	chr6	144495167	144495426	1	NM_0071	UTRN	117576	NM_0312	SF3B5	78542	intronic	STX11	18.04474	Up	luks_peak	NULL
peak864	chr6	144503452	144503740	1	NM_0071	UTRN	109277	NM_0312	SF3B5	86842	intronic	STX11	6.04527	Up	NULL	NULL
peak865	chr6	153119157	153119352	0	NR_10698	MIR7641-	622357	NM_1829	SYNE1	160720	intergenic	VIP(dist=1	10.52474	Down	NULL	PBS_peak_9346
peak866	chr6	154493914	154494295	4	NM_0012	SCAF8	560407	NM_0124	RGS17	1041715	intronic	IPCEF1,O	7.22147	Down	NULL	PBS_peak_9350
peak867	chr6	154637633	154637837	3	NM_0012	SCAF8	416777	NM_0124	RGS17	1185346	intronic	IPCEF1	5.40219	Down	NULL	PBS_peak_9357
peak868	chr6	154643374	154643543	3	NM_0012	SCAF8	411053	NM_0124	RGS17	1191069	intronic	IPCEF1	14.24514	Down	NULL	NULL
peak869	chr6	154644211	154644435	3	NM_0012	SCAF8	410189	NM_0124	RGS17	1191934	intronic	IPCEF1	5.64027	Down	NULL	PBS_peak_9360
peak870	chr6	154645277	154645479	3	NM_0012	SCAF8	409134	NM_0124	RGS17	1192989	intronic	IPCEF1	25.22086	Down	NULL	PBS_peak_9361
peak871	chr6	154648887	154649077	3	NM_0012	SCAF8	405530	NM_0124	RGS17	1196593	intronic	IPCEF1	10.25588	Down	NULL	PBS_peak_9363
peak872	chr6	154649457	154649617	3	NM_0012	SCAF8	404975	NM_0124	RGS17	1197148	intronic	IPCEF1	4.44726	Down	NULL	PBS_peak_9364
peak873	chr6	154651499	154651757	2	NM_0012	SCAF8	402884	NM_0011	IPCEF1	413	intronic	IPCEF1	5.39982	Down	NULL	PBS_peak_9366
peak874	chr6	154664635	154664803	2	NM_0012	SCAF8	389793	NM_0011	IPCEF1	13504	intronic	IPCEF1	8.8594	Down	NULL	PBS_peak_9369
peak875	chr6	154664879	154665038	2	NM_0012	SCAF8	389553	NM_0011	IPCEF1	13743	intronic	IPCEF1	10.00195	Down	NULL	PBS_peak_9369
peak876	chr6	154672144	154672294	2	NM_0012	SCAF8	382293	NM_0011	IPCEF1	21004	intronic	IPCEF1	6.66256	Down	NULL	NULL
peak877	chr6	155070858	155071137	5	NR_03610	MIR1273C	103496	NM_1735	CNKSR3	239244	intronic	SCAF8	6.35251	Down	NULL	PBS_peak_9379
peak878	chr6	155079695	155079855	5	NR_03610	MIR1273C	94719	NM_1735	CNKSR3	248022	intronic	SCAF8	5.32977	Down	NULL	PBS_peak_9383
peak879	chr6	158266751	158266910	1	NM_0038	SYNJ2	136057	NM_0184	TMEM242	521539	intronic	SNX9	20.28096	Up	NULL	NULL
peak880	chr6	159813849	159814038	1	NM_0012	WTAP	334086	NR_12166	LOC10192	222904	ncRNA_in	LOC10272	11.56173	Down	NULL	PBS_peak_9404
peak881	chr6	160076567	160076722	0	NM_0012	WTAP	71385	NR_12166	LOC10192	485605	intergenic	LOC10272	8.94047	Up	NULL	NULL
peak882	chr6	160078451	160078617	0	NM_0012	WTAP	69496	NR_12166	LOC10192	487495	intergenic	LOC10272	31.23919	Up	NULL	NULL
peak883	chr6	160625777	160625969	0	NM_0219	SLC22A3	143532	NR_04751	AIRN	197177	intergenic	SLC22A1(	7.98998	Down	NULL	PBS_peak_9424
peak884	chr6	160761141	160761307	0	NM_0219	SLC22A3	8181	NM_0030	SLC22A2	81261	intergenic	SLC22A2(	6.03036	Down	NULL	PBS_peak_9441
peak885	chr6	161011717	161011909	1	NM_0011	PLG	111412	NR_02809	LPAL2	79657	intronic	LPA	7.49865	Down	NULL	PBS_peak_9459
peak886	chr6	161019706	161019943	1	NM_0011	PLG	103400	NR_02809	LPAL2	87668	intronic	LPA	15.07929	Down	NULL	PBS_peak_9460
peak887	chr6	161026494	161026645	1	NM_0011	PLG	96655	NR_02809	LPAL2	94413	intronic	LPA	4.30149	Down	NULL	PBS_peak_9465
peak888	chr6	161266517	161266676	0	NM_0012	MAP3K4	146162	NM_0055	LPA	179189	intergenic	PLG(dist=	3.88047	Down	NULL	PBS_peak_9474
peak889	chr6	161279353	161279651	0	NM_0012	MAP3K4	133257	NM_0055	LPA	192095	intergenic	PLG(dist=	18.66294	Down	NULL	PBS_peak_9476
peak890	chr6	162190732	162190893	3	NM_0010	PACRG	957351	NM_0201	AGPAT4	495705	intronic	PRKN	4.28597	Down	NULL	PBS_peak_9490
peak891	chr6	162197213	162197388	3	NM_0010	PACRG	950863	NM_0201	AGPAT4	502193	intronic	PRKN	10.23997	Down	NULL	PBS_peak_9492
peak892	chr6	163868445	163868599	5	NR_10698	MIR7641-	1954568	NR_03759	CAHM	33540	intronic	QKI	3.55135	Up	luks_peak	NULL
peak893	chr6	163915666	163915839	5	NR_10698	MIR7641-	1907337	NR_03759	CAHM	80770	intronic	QKI	3.81419	Down	NULL	PBS_peak_9545
peak894	chr6	166027979	166028143	2	NR_02728	LINC0060	372978	NM_1449	C6orf118	304950	intronic	PDE10A	5.62065	Down	NULL	PBS_peak_9570
peak895	chr6	166478111	166478271	0	NR_12586	LOC10192	172266	NR_02686	LINC0047	76664	intergenic	LINC0060	5.64414	Down	NULL	PBS_peak_9600
peak896	chr6	167028359	167028580	4	NR_04664	RPS6KA2	288716	NM_001318938		70291	intronic	RPS6KA2	8.87642	Up	luks_peak	NULL
peak897	chr6	167032314	167032487	4	NR_04664	RPS6KA2	284785	NM_001318938		74222	intronic	RPS6KA2	6.75638	Up	NULL	NULL
peak898	chr6	167736887	167737037	0	NM_0319	TTLL2	1612	NR_134592		153494	upstream	TTLL2	5.41849	Down	NULL	PBS_peak_9604

peak899	chr6	167865094	167865253	0	NM_0010	MLLT4	362497	NM_0046	TCP10	67175	intergenic	TCP10(dis	4.69421	Down	NULL	PBS_peak_9607
peak900	chr7	4726327	4726481	1	NM_0148	AP5Z1	88858	NR_12602	LOC10012	1528198	intronic	FOXK1	6.99663	Down	NULL	PBS_peak_9633
peak901	chr7	4736957	4737111	1	NM_0148	AP5Z1	78228	NR_12602	LOC10012	1538828	intronic	FOXK1	4.87598	Down	NULL	PBS_peak_9636
peak902	chr7	4739060	4739232	1	NM_0148	AP5Z1	76116	NR_12602	LOC10012	1540940	intronic	FOXK1	4.53911	Down	NULL	PBS_peak_9637
peak903	chr7	4739614	4739773	1	NM_0148	AP5Z1	75568	NR_12602	LOC10012	1541487	intronic	FOXK1	15.66495	Down	NULL	NULL
peak904	chr7	4741069	4741257	1	NM_0148	AP5Z1	74099	NR_12602	LOC10012	1542957	intronic	FOXK1	7.20931	Down	NULL	PBS_peak_9638
peak905	chr7	4741561	4741725	1	NM_0148	AP5Z1	73619	NR_12602	LOC10012	1543437	intronic	FOXK1	25.95701	Down	NULL	NULL
peak906	chr7	12463647	12463856	0	NM_0011	SCIN	146451	NM_0011	VWDE	19899	intergenic	VWDE(di	4.80732	Up	NULL	NULL
peak907	chr7	22302396	22302555	1	NR_03839	LOC10050	300480	NM_0187	CDCA7L	316933	intronic	RAPGEF5	4.88068	Down	NULL	NULL
peak908	chr7	44840596	44840904	2	NR_03077	CCM2	198595	NM_0013	TMED4	218856	exonic	PPIA	14.58191	Down	NULL	PBS_peak_9707
peak909	chr7	45148598	45148811	4	NM_0058	RAMP3	48662	NR_00299	SNORA5I	3006	exonic	TBRG4	21.17226	Down	NULL	PBS_peak_9708
peak910	chr7	47401767	47401941	1	NR_10809	LINC0144	259683	NR_134575		665134	intronic	TNS3	3.58621	Up	luks_peak	NULL
peak911	chr7	66395377	66395547	1	NR_134540		66330	NR_00393	GTF2IRD	85649	intronic	TMEM248	5.11837	Down	NULL	PBS_peak_9720
peak912	chr7	67035234	67035391	0	NR_12051	LOC10272	449927	NR_02200	PMS2P4	267883	intergenic	LINC0137	9.92965	Down	NULL	NULL
peak913	chr7	69288427	69288598	3	NM_0224	WBSCR17	1309010	NR_10810	LOC10050	226031	intronic	AUTS2	3.787	Down	NULL	PBS_peak_9725
peak914	chr7	71735783	71735952	2	NR_03979	MIR4650-	427006	NR_03747	MIR3914-	963111	intronic	CALN1	10.49003	Down	NULL	PBS_peak_9729
peak915	chr7	73592569	73592721	2	NR_03032	MIR590	12883	NM_1525	WBSCR27	335790	intronic	EIF4H	3.7191	Down	NULL	PBS_peak_9736
peak916	chr7	74088838	74089004	6	NM_0002	NCF1	99388	NM_1814	RFC2	420133	intronic	GTF2I	6.95628	Down	NULL	PBS_peak_9759
peak917	chr7	74103033	74103212	6	NM_0002	NCF1	85186	NM_1814	RFC2	434334	intronic	GTF2I	3.77923	Down	NULL	PBS_peak_9762
peak918	chr7	75959490	75959765	1	NM_0071	ZP3	67213	NR_135079		221547	exonic	YWHAG	4.58197	Down	NULL	PBS_peak_9768
peak919	chr7	77254600	77254750	3	NM_1984	RSBN1L	71068	NM_0174	GSAP	208958	intronic	PTPN12	5.3086	Up	NULL	NULL
peak920	chr7	77340129	77340281	1	NM_0011	PHTF2	87904	NR_03830	APTR	13543	intronic	RSBN1L	4.27955	Down	NULL	PBS_peak_9795
peak921	chr7	79178310	79178472	0	NR_03166	MIR548M	222181	NM_0123	MAGI2	95501	intergenic	MAGI2-A	8.30376	Down	NULL	PBS_peak_9802
peak922	chr7	79617462	79617638	0	NM_0020	GNAI1	146590	NM_0123	MAGI2	534660	intergenic	MAGI2-A	15.71692	Down	NULL	PBS_peak_9803
peak923	chr7	81961407	81961584	2	NR_11007	LOC10192	2200296	NM_0010	HGF	561981	intronic	CACNA2I	4.22543	Down	NULL	PBS_peak_9805
peak924	chr7	85721949	85722100	0	NM_0008	GRM3	551205	NM_1527	SEMA3D	970777	intergenic	LINC0097	8.47707	Down	NULL	NULL
peak925	chr7	86515945	86516124	4	NM_0011	DMTF1	265642	NM_1527	SEMA3D	1764787	intronic	KIAA1324	6.12786	Down	NULL	PBS_peak_9809
peak926	chr7	89570497	89570655	1	NR_00355	DPY19L2	178138	NM_1527	C7orf62	1145545	ncRNA_in	STEAP2-A	5.20688	Up	NULL	NULL
peak927	chr7	94947989	94948151	1	NM_1458	ASB4	167143	NM_0039	SGCE	662549	intronic	PON1	17.42964	Down	NULL	NULL
peak928	chr7	96118008	96118171	2	NM_0052	DLX6	517200	NR_02766	SLC25A13	166630	intronic	SEM1	4.90604	Down	NULL	NULL
peak929	chr7	103421993	103422165	2	NM_1990	LHFPL3	547025	NM_1989	SLC26A5	335455	intronic	RELN	5.76063	Down	NULL	PBS_peak_9827
peak930	chr7	111808749	111808926	1	NM_0219	ZNF277	37805	NM_0012	IMMP2L	606264	intronic	DOCK4	6.91794	Up	NULL	NULL
peak931	chr7	130585242	130585409	3	NR_10978	LOC10050	12897	NR_0295	MIR29B1	23027	ncRNA_in	LINC-PIN	6.736	Up	NULL	NULL
peak932	chr7	136264750	136264958	0	NM_0010	CHRM2	288545	NM_1458	MTPN	602650	intergenic	MTPN(dis	4.55403	Down	NULL	PBS_peak_9881
peak933	chr7	138748936	138749215	2	NM_0011	TTC26	69414	NM_0806	ZC3HAV	28300	intronic	ZC3HAV1	5.07052	Up	luks_peak	NULL
peak934	chr7	138750398	138750598	2	NM_0011	TTC26	67992	NM_0806	ZC3HAV	29723	intronic	ZC3HAV1	4.75121	Up	luks_peak	NULL
peak935	chr7	138752819	138752987	2	NM_0011	TTC26	65587	NM_0806	ZC3HAV	32128	intronic	ZC3HAV1	11.30804	Up	NULL	NULL
peak936	chr7	138756407	138756575	2	NM_0011	TTC26	61999	NM_0806	ZC3HAV	35716	intronic	ZC3HAV1	3.83268	Up	luks_peak	NULL
peak937	chr7	138765181	138765535	2	NM_0011	TTC26	53132	NM_0806	ZC3HAV	44583	intronic	ZC3HAV1	3.99385	Up	luks_peak	NULL
peak938	chr7	138766718	138766935	2	NM_0011	TTC26	51663	NM_0806	ZC3HAV	46051	intronic	ZC3HAV1	7.45132	Up	luks_peak	NULL
peak939	chr7	138768894	138769238	2	NM_0011	TTC26	49424	NM_0806	ZC3HAV	48291	intronic	ZC3HAV1	7.55575	Up	luks_peak	NULL
peak940	chr7	138771062	138771233	2	NM_0011	TTC26	47342	NM_0806	ZC3HAV	50372	intronic	ZC3HAV1	5.28278	Up	luks_peak	NULL
peak941	chr7	138771876	138772075	2	NM_0011	TTC26	46514	NM_0806	ZC3HAV	51200	intronic	ZC3HAV1	8.3671	Up	NULL	NULL
peak942	chr7	138773970	138774279	2	NM_0011	TTC26	44365	NM_0806	ZC3HAV	53349	intronic	ZC3HAV1	5.2342	Up	luks_peak	NULL
peak943	chr7	138778405	138778555	2	NM_0011	TTC26	40010	NM_0806	ZC3HAV	57705	intronic	ZC3HAV1	5.1728	Up	luks_peak	NULL
peak944	chr7	138779258	138779425	2	NM_0011	TTC26	39148	NM_0806	ZC3HAV	58566	intronic	ZC3HAV1	4.66049	Up	luks_peak	NULL
peak945	chr7	138782470	138782646	2	NM_0011	TTC26	35932	NM_0806	ZC3HAV	61783	intronic	ZC3HAV1	8.62541	Up	NULL	NULL
peak946	chr7	139055277	139055457	4	NM_0010	CLEC2L	153307	NM_0246	ZC3HAV	260902	intronic	C7orf55-L	3.53564	Down	NULL	PBS_peak_9890
peak947	chr7	139055548	139055720	4	NM_0010	CLEC2L	153040	NM_0246	ZC3HAV	261169	intronic	C7orf55-L	3.79548	Down	NULL	PBS_peak_9890
peak948	chr7	139442490	139442651	2	NM_0011	TBXAS1	35476	NM_1985	KLRG2	274113	intronic	HIPK2	8.04724	Up	NULL	NULL
peak949	chr7	139452881	139453064	2	NM_0011	TBXAS1	25074	NM_1985	KLRG2	284515	intronic	HIPK2	4.13113	Up	luks_peak	NULL
peak950	chr7	139726205	139726386	2	NR_02445	JHDM1D-	150765	NM_0227	HIPK2	248602	intronic	PARP12	5.53339	Up	NULL	NULL
peak951	chr7	139737913	139738090	2	NR_02445	JHDM1D-	139059	NM_0227	HIPK2	260308	intronic	PARP12	5.28629	Up	luks_peak	NULL
peak952	chr7	139739969	139740125	2	NR_02445	JHDM1D-	137014	NM_0227	HIPK2	262354	intronic	PARP12	7.03744	Up	luks_peak	NULL
peak953	chr7	139740196	139740366	2	NR_02445	JHDM1D-	136780	NM_0227	HIPK2	262588	intronic	PARP12	4.00014	Up	luks_peak	NULL
peak954	chr7	139747152	139747315	2	NR_02445	JHDM1D-	129827	NM_0227	HIPK2	269540	intronic	PARP12	19.73786	Up	NULL	NULL
peak955	chr7	139747747	139747917	2	NR_02445	JHDM1D-	129229	NM_0227	HIPK2	270139	intronic	PARP12	16.89553	Up	NULL	NULL
peak956	chr7	139757433	139757589	2	NR_02445	JHDM1D-	119550	NM_0227	HIPK2	279818	intronic	PARP12	4.67699	Up	NULL	NULL
peak957	chr7	144757723	144757902	0	NM_0141	CNTNAP2	1055640	NM_0224	TPK1	224666	intergenic	TPK1(dist	6.09811	Down	NULL	PBS_peak_9897
peak958	chr7	148412186	148412488	1	NR_13010	GHET1	272282	NR_03169	MIR548I4	1157375	intronic	CUL1	10.9314	Down	NULL	PBS_peak_9911
peak959	chr7	148558513	148558699	5	NR_13010	GHET1	126013	NR_03169	MIR548I4	1303644	intronic	EZH2	5.65397	Down	NULL	PBS_peak_9926
peak960	chr7	150797447	150797659	4	NM_0013	AGAP3	14212	NM_0011	TMUB1	16933	intronic	AGAP3	12.20655	Up	luks_peak	NULL
peak961	chr7	151211506	151211657	1	NR_03892	PRKAG2-	362545	NR_13177	CRYGN	73682	intronic	RHEB	9.59997	Down	NULL	NULL
peak962	chr7	151288045	151288367	5	NR_03892	PRKAG2-	285921	NM_0056	RHEB	71196	intronic	PRKAG2	4.51607	Up	luks_peak	NULL
peak963	chr7	151288673	151288855	5	NR_03892	PRKAG2-	285363	NM_0056	RHEB	71754	intronic	PRKAG2	7.24613	Up	NULL	NULL
peak964	chr7	151289018	151289188	5	NR_03892	PRKAG2-	285024	NM_0056	RHEB	72093	intronic	PRKAG2	24.24989	Up	NULL	NULL
peak965	chr7	151290001	151290151	5	NR_03892	PRKAG2-	284051	NM_0056	RHEB	73066	intronic	PRKAG2	5.49954	Up	NULL	NULL
peak966	chr7	151296339	151296501	5	NR_03892	PRKAG2-	277707	NM_0056	RHEB	79410	intronic	PRKAG2	4.56118	Up	NULL	NULL
peak967	chr7	151305379	151305556	5	NR_03892	PRKAG2-	268659	NM_0056	RHEB	88457	intronic	PRKAG2	4.76335	Up	luks_peak	NULL
peak968	chr7	151309717	151309896	5	NR_03892	PRKAG2-	264320	NM_0056	RHEB	92796	intronic	PRKAG2	5.01093	Up	luks_peak	NULL
peak969	chr7	151311000	151311180	5	NR_03892	PRKAG2-	263037	NM_0056	RHEB	94080	intronic	PRKAG2	4.64735	Up	luks_peak	NULL
peak970	chr7	151311260	151311444	5	NR_03892	PRKAG2-	262775	NM_0056	RHEB	94342	intronic	PRKAG2	16.0069	Up	luks_peak	NULL
peak971	chr7	151312895	151313168	5	NR_03892	PRKAG2-	261095	NM_0056	RHEB	96021	intronic	PRKAG2	29.51212	Up	luks_peak	NULL
peak972	chr7	151313571	151313760	5	NR_03892	PRKAG2-	260461	NM_0056	RHEB	96655	intronic	PRKAG2	6.94914	Up	NULL	NULL
peak973	chr7	151313959	151314113	5	NR_03892	PRKAG2-	260091	NM_0056	RHEB	97026	intronic	PRKAG2	21.65033	Up	NULL	NULL
peak974	chr7	152092411	152092576	1	NR_00293	FABP5P3	41486	NM_0162	PRKAG2	518177	intronic	KMT2C	3.64366	Down	NULL	PBS_peak_9954
peak975	chr7	152953247	152953399	0	NM_0010	DPP6	630859	NM_0054	XRCC2	580073	intergenic	ACTR3B(	4.27447	Down	NULL	PBS_peak_9969
peak976	chr7	153278917	153279083	0	NM_0010	DPP6	305182	NR_12577	LINC0128	169681	intergenic	LINC0128	12.88218	Down	NULL	NULL
peak977	chr7	153801869	153802043	3	NM_0012	DPP6	200341	NR_12577	LINC0128	692637	intronic	DPP6	4.64094	Down	NULL	PBS_peak_9977
peak978	chr7	155507759	155507933	1	NM_0012	LOC38960	247480	NR_12634	LOC10050	70746	intronic	RBM33	4.65836	Down	NULL	PBS_peak_9986
peak979	chr7	156936273	156936459	1	NM_0054	DNAJB6	193344	NM_0055	MNX1	133019	intronic	UBE3C	5.15003	Down	NULL	PBS_peak_9990
peak980	chr7	156949182	156949357	1	NM_0054	DNAJB6	180440	NM_0055	MNX1	145922	intronic	UBE3C	4.31833	Down	NULL	PBS_peak_9998
peak981	chr7	157166479	157166643	2	NR_11015	LOC10192	92364	NM_0055	MNX1	363214	intronic	DNAJB6	6.90573	Up	luks_peak	NULL
peak982	chr7	157192019	157192271	1	NR_11015	LOC10192	66780	NM_0055	MNX1	388798	intronic	DNAJB6	4.50556	Up	luks_peak	NULL
peak983	chr7	157192604	157192754	1	NR_11015	LOC10192	66246	NM_0055	MNX1	389332	intronic	DNAJB6	4.90197	Up	luks_peak	NULL
peak984	chr7	157207418	157207600	1	NR_11015	LOC10192	51416	NM_0055	MNX1	404162	intronic	DNAJB6	10.2579	Up	luks_peak	NULL
peak985	chr8	529914	530094	0	NR_07339	ERICH1-A	157601	NM_1750	TDRP	34223	intergenic	TDRP(dist	6.54364	Down	NULL	PBS_peak_10024
peak986	chr8	773466	773641	1	NM_0012	DLGAP2	675978	NM_2073	ERICH1	92314	intronic	DLGAP2	6.40779	Down	NULL	NULL
peak987	chr8	809516	809695	1	NM_0012	DLGAP2	639926	NM_2073	ERICH1	128366	intronic	DLGAP2	7.48861	Down	NULL	NULL
peak988	chr8	1465226	1465434	2	NM_0189	CLN8	246540	NR_11194	LOC28608	214507	intronic	DLGAP2	3.93679	Down	NULL	PBS_peak_10040
peak989	chr8	2168593	2168810	0	NM_0011	MCPH1	4095411	NR_134303		455951	intergenic	MYOM2(	9.87834	Down	NULL	PBS_peak_10043
peak990	chr8	2245298	2245452	0	NM_0011	MCPH1	4018738	NR_134303		532625	intergenic	MYOM2(	4.05405	Down	NULL	PBS_peak_10051
peak991	chr8	3017111	3017318	1	NM_0011	MCPH1	3246898	NR_12542	LOC10192	431223	intronic	CSMD1	21.78594	Down	NULL	PBS_peak_10079
peak992	chr8	3458938	3459147	1	NM_0011	MCPH1	2805070	NR_12542	LOC10192	873051	intronic	CSMD1	9.9266	Down	NULL	PBS_peak_10086
peak993	chr8	5210978	5211153	0	NM_0011	MCPH1	1053047	NM_0332	CSMD1	358737	intergenic	CSMD1(d	7.16683	Down	NULL	PBS_peak_10115
peak994	chr8	5340857	5341007	0	NM_0011	MCPH1	923181	NM_0332	CSMD1	488604	intergenic	CSMD1(d	6.01356	Down	NULL	PBS_peak_10125
peak995	chr8	5423195	5423352	0	NM_0011	MCPH1	840839	NM_0332	CSMD1	570945	intergenic	CSMD1(d	4.39745	Down	NULL	PBS_peak_10139
peak996	chr8	5710148	5710314	0	NM_0011	MCPH1	553882	NM_0332	CSMD1	857903	intergenic	CSMD1(d	7.24234	Down	NULL	PBS_peak_10174
peak997	chr8	5905166	5905444	0	NM_0011	MCPH1	358808	NM_0332	CSMD1	1052977	intergenic	CSMD1(d	3.97892	Down	NULL	PBS_peak_10198
peak998	chr8	11704009	11704272	6	NM_0010	DEFB135	135689	NM_0532	FAM167A	379864	intronic	CTSB	3.74772	Up	luks_peak	NULL
peak999	chr8	12752853	12753044	0	NM_0208	KIAA1456	50234	NR_01538	LOC34032	84038	intergenic	LINC0068	5.68173	Down	NULL	PBS_peak_10208
peak1000	chr8	12969284	12969480	4	NM_0010	C8orf48	454970	NR_01538	LOC34032	300472	intronic	DLC1	11.9844	Down	NULL	PBS_peak_10232
peak1001	chr8	13018427	13018593	2	NM_0010	C8orf48	405842	NM_0060	DLC1	27701	intronic	DLC1	4.03547	Up	NULL	NULL

peak1002	chr8	13036731	13036912	2	NM_0010	C8orf48	387530	NM_0060	DLC1	46012	intronic	DLC1	9.60976	Down	NULL	NULL
peak1003	chr8	13047748	13047912	2	NM_0010	C8orf48	376522	NM_0060	DLC1	57021	intronic	DLC1	3.28419	Down	NULL	PBS_peak_10241
peak1004	chr8	13292817	13292988	2	NM_0010	C8orf48	131449	NM_00131	6668	158845	intronic	DLC1	4.57969	Down	NULL	PBS_peak_10264
peak1005	chr8	13386156	13386342	0	NM_0010	C8orf48	38103	NM_0247	DLC1	13820	intronic	DLC1	17.03146	Down	NULL	PBS_peak_10273
peak1006	chr8	13436550	13436726	0	NR_134450		50561	NM_0247	DLC1	64209	intronic	DLC1	4.3722	Down	NULL	PBS_peak_10276
peak1007	chr8	13466519	13466713	0	NR_134450		20583	NM_0247	DLC1	94187	intergenic	DLC1(dist	19.36326	Down	NULL	PBS_peak_10277
peak1008	chr8	13645105	13645260	0	NM_0067	TUSC3	1752413	NM_0247	DLC1	272753	intergenic	LOC1027	6.79508	Down	NULL	PBS_peak_10284
peak1009	chr8	13654408	13654572	0	NM_0067	TUSC3	1743106	NM_0247	DLC1	282061	intergenic	LOC1027	5.42983	Down	NULL	PBS_peak_10285
peak1010	chr8	13759463	13759646	0	NM_0067	TUSC3	1638041	NM_0247	DLC1	387125	intergenic	LOC1027	4.11398	Down	NULL	PBS_peak_10291
peak1011	chr8	13949392	13949558	1	NM_0067	TUSC3	1448121	NM_0247	DLC1	577046	intronic	SGCZ	3.89644	Down	NULL	PBS_peak_10296
peak1012	chr8	13965948	13966118	1	NM_0067	TUSC3	1431563	NM_0247	DLC1	593604	intronic	SGCZ	3.51396	Down	NULL	PBS_peak_10299
peak1013	chr8	14335199	14335354	1	NM_0067	TUSC3	1062319	NM_0247	DLC1	962847	intronic	SGCZ	4.54941	Down	NULL	NULL
peak1014	chr8	14824809	14824967	1	NM_0067	TUSC3	572708	NR_0298	MIR383	113869	intronic	SGCZ	4.84554	Down	NULL	PBS_peak_10305
peak1015	chr8	21824085	21824244	1	NM_0012	NPM2	57456	NM_201349		52945	intronic	XPO7	9.65589	Down	NULL	NULL
peak1016	chr8	24411177	24411339	0	NM_0053	NEFM	360016	NR_1258	LOC1019	5127	intergenic	LOC1019	10.08438	Down	NULL	PBS_peak_10318
peak1017	chr8	24543310	24543484	0	NM_0053	NEFM	227877	NR_1258	LOC1019	137266	intergenic	LOC1019	4.46349	Down	NULL	PBS_peak_10325
peak1018	chr8	25586070	25586265	0	NM_0027	PPP2R2A	562839	NM_0176	KCTD9	270183	intergenic	CDCA2(d	8.77802	Down	NULL	PBS_peak_10344
peak1019	chr8	25750313	25750480	1	NM_0027	PPP2R2A	398610	NM_0176	KCTD9	434412	intronic	EBF2	4.71323	Down	NULL	PBS_peak_10352
peak1020	chr8	26186832	26186989	2	NM_0043	BNIP3L	53612	NM_0226	EBF2	284270	intronic	PPP2R2A	5.89462	Down	NULL	PBS_peak_10368
peak1021	chr8	26206787	26206939	2	NM_0043	BNIP3L	33660	NM_0226	EBF2	304223	intronic	PPP2R2A	4.49945	Down	NULL	PBS_peak_10379
peak1022	chr8	26691996	26692158	4	NM_1731	PTK2B	476922	NM_0072	PNMA2	320594	intronic	ADRA1A	5.83464	Down	NULL	PBS_peak_10382
peak1023	chr8	35355928	35356079	1	NM_0010	KCNU1	1285838	NM_0240	DUSP26	1898379	intronic	UNC5D	5.2935	Up	NULL	NULL
peak1024	chr8	41894046	41894208	2	NM_0011	AP3M2	116337	NM_0011	ANK1	139847	intronic	KAT6A	4.80266	Down	NULL	PBS_peak_10414
peak1025	chr8	42390007	42390243	2	NM_0011	SMIM19	6173	NM_0012	SLC20A2	31147	intronic	SLC20A2	10.19143	Down	NULL	PBS_peak_10421
peak1026	chr8	48859483	48859758	2	NM_0059	MCM4	13142	NM_0051	CEBPD	208894	intronic	PRKDC	7.4888	Down	NULL	PBS_peak_10433
peak1027	chr8	56835047	56835305	2	NM_0010	CHCHD7	289021	NM_0012	TMEM68	149210	intronic	LYN	4.36323	Down	NULL	PBS_peak_10465
peak1028	chr8	56838068	56838242	2	NM_0010	CHCHD7	286042	NM_0012	TMEM68	152189	intronic	LYN	6.25896	Down	NULL	PBS_peak_10466
peak1029	chr8	57897815	57897969	1	NR_0382	LINC0160	232943	NR_0382	LINC0096	425510	intronic	IMPAD1	20.84954	Down	NULL	NULL
peak1030	chr8	61600578	61600755	1	NM_00131	6690	53151	NR_1038	LINC0130	274289	intronic	CHD7	5.47133	Down	NULL	PBS_peak_10471
peak1031	chr8	61605242	61605413	1	NM_00131	6690	48490	NR_1038	LINC0130	278950	intronic	CHD7	9.39871	Down	NULL	PBS_peak_10474
peak1032	chr8	61636619	61636805	1	NM_00131	6690	17106	NR_1038	LINC0130	310335	intronic	CHD7	21.86463	Down	NULL	PBS_peak_10476
peak1033	chr8	61677094	61677255	2	NM_1735	CLVS1	523350	NR_1038	LINC0130	350797	intronic	CHD7	4.47248	Down	NULL	PBS_peak_10477
peak1034	chr8	63378459	63378611	2	NR_0273	UG0898H	511885	NM_0011	ASPH	751336	intronic	NKAIN3	4.97371	Down	NULL	PBS_peak_10481
peak1035	chr8	66709601	66709762	1	NM_0331	DNAJC5B	224109	NM_0026	PDE7A	8352	intronic	PDE7A	5.30226	Down	NULL	PBS_peak_10515
peak1036	chr8	68758725	68758901	0	NM_0251	PREX2	105790	NM_0203	CPA6	100193	intergenic	CPA6(dist	5.86598	Down	NULL	NULL
peak1037	chr8	68821342	68821510	0	NM_0251	PREX2	43177	NM_0203	CPA6	162806	intergenic	CPA6(dist	4.44747	Down	NULL	PBS_peak_10523
peak1038	chr8	68822398	68822549	0	NM_0251	PREX2	42129	NM_0203	CPA6	163853	intergenic	CPA6(dist	8.23714	Down	NULL	NULL
peak1039	chr8	69619447	69619597	1	NM_0011	SULF1	759337	NR_0388	C8orf34-A	375796	intronic	C8orf34	5.3587	Down	NULL	NULL
peak1040	chr8	70059497	70059658	0	NM_0011	SULF1	319281	NR_0399	LINC0159	43152	intergenic	LINC0159	5.31346	Down	NULL	PBS_peak_10570
peak1041	chr8	70163814	70163980	0	NM_0011	SULF1	214962	NR_0399	LINC0159	147472	intergenic	LINC0159	5.3807	Down	NULL	PBS_peak_10580
peak1042	chr8	70240846	70241006	0	NM_0011	SULF1	137933	NR_0399	LINC0159	224501	intergenic	LINC0159	3.91888	Down	NULL	PBS_peak_10586
peak1043	chr8	72106035	72106207	0	NR_0336	MSC-AS1	649237	NM_0160	LACTB2	524674	intergenic	XKR9(dist	7.68461	Down	NULL	PBS_peak_10602
peak1044	chr8	73003807	73003968	0	NM_0047	KCNB2	445738	NM_0073	TRPA1	16068	intergenic	TRPA1(di	18.68355	Down	NULL	NULL
peak1045	chr8	73031656	73031830	0	NM_0047	KCNB2	417883	NM_0073	TRPA1	43924	intergenic	TRPA1(di	9.94683	Down	NULL	PBS_peak_10605
peak1046	chr8	73132333	73132513	1	NM_0047	KCNB2	317203	NM_0073	TRPA1	144604	ncRNA_in	LOC3922	5.4045	Down	NULL	PBS_peak_10608
peak1047	chr8	73161763	73161959	1	NM_0047	KCNB2	287765	NM_0073	TRPA1	174042	ncRNA_in	LOC3922	4.88302	Down	NULL	PBS_peak_10611
peak1048	chr8	73266655	73266864	0	NM_0047	KCNB2	182866	NR_0338	LOC3922	102890	intergenic	LOC3922	20.51278	Down	NULL	PBS_peak_10617
peak1049	chr8	73275271	73275499	0	NM_0047	KCNB2	174241	NR_0338	LOC3922	111516	intergenic	LOC3922	4.86446	Down	NULL	PBS_peak_10618
peak1050	chr8	73326991	73327180	0	NM_0047	KCNB2	122540	NR_0338	LOC3922	163216	intergenic	LOC3922	16.11746	Down	NULL	PBS_peak_10621
peak1051	chr8	73381362	73381550	0	NM_0047	KCNB2	68170	NR_0338	LOC3922	217587	intergenic	LOC3922	8.64522	Down	NULL	PBS_peak_10623
peak1052	chr8	73381685	73381860	0	NM_0047	KCNB2	67853	NR_0338	LOC3922	217903	intergenic	LOC3922	4.87034	Down	NULL	PBS_peak_10624
peak1053	chr8	73547313	73547475	1	NR_1106	LOC1019	96886	NR_0338	LOC3922	383525	intronic	KCNB2	4.43414	Down	NULL	PBS_peak_10631
peak1054	chr8	73579428	73579579	1	NR_1106	LOC1019	64776	NR_0338	LOC3922	415634	intronic	KCNB2	3.97428	Down	NULL	PBS_peak_10632
peak1055	chr8	73615514	73615785	1	NR_1106	LOC1019	28630	NR_0338	LOC3922	451780	intronic	KCNB2	4.57992	Down	NULL	PBS_peak_10635
peak1056	chr8	73703711	73703888	1	NM_0032	TERF1	217297	NR_0338	LOC3922	539930	intronic	KCNB2	4.78269	Down	NULL	PBS_peak_10653
peak1057	chr8	74765189	74765357	6	NM_0010	TMEM70	123104	NM_0011	STAU2	105330	intronic	UBE2W	13.24002	Down	NULL	PBS_peak_10659
peak1058	chr8	74767270	74767434	6	NM_0010	TMEM70	121025	NM_0011	STAU2	107409	intronic	UBE2W	7.84305	Down	NULL	PBS_peak_10661
peak1059	chr8	74772027	74772185	6	NM_0010	TMEM70	116271	NM_0011	STAU2	112163	intronic	UBE2W	4.18709	Down	NULL	PBS_peak_10664
peak1060	chr8	74772983	74773250	6	NM_0010	TMEM70	115260	NM_0011	STAU2	113173	intronic	UBE2W	19.40923	Down	NULL	PBS_peak_10665
peak1061	chr8	74878232	74878433	9	NM_0010	TMEM70	10044	NR_0731	UBE2W	87187	intronic	ELOC	4.81966	Down	NULL	PBS_peak_10679
peak1062	chr8	74880225	74880378	9	NM_0010	TMEM70	8075	NR_0731	UBE2W	89156	intronic	ELOC	6.01915	Down	NULL	PBS_peak_10681
peak1063	chr8	76862934	76863095	0	NR_1050	LINC0111	455874	NR_1038	CASC9	671890	intergenic	HNF4G(d	4.54346	Down	NULL	PBS_peak_10684
peak1064	chr8	79184121	79184302	0	NM_0068	PKIA	244124	NM_0011	PEX2	1270931	intergenic	LOC1027	5.28778	Down	NULL	PBS_peak_10699
peak1065	chr8	80049237	80049388	0	NM_0011	STMN2	473736	NM_0011	IL7	331554	intergenic	IL7(dist=3	19.95578	Down	NULL	NULL
peak1066	chr8	80156572	80156772	0	NM_0011	STMN2	366377	NM_0011	IL7	438914	intergenic	IL7(dist=4	7.03388	Down	NULL	PBS_peak_10737
peak1067	chr8	80392833	80392988	0	NM_0011	STMN2	130138	NM_0011	IL7	675152	intergenic	IL7(dist=6	3.74865	Down	NULL	PBS_peak_10766
peak1068	chr8	82833044	82833276	0	NR_134295		991179	NM_1528	SNX16	78639	intergenic	SNX16(di	5.11393	Down	NULL	PBS_peak_10776
peak1069	chr8	83185370	83185568	0	NR_134295		638870	NM_1528	SNX16	430948	intergenic	SNX16(di	4.61627	Down	NULL	PBS_peak_10793
peak1070	chr8	83488636	83488825	0	NR_134295		335608	NM_1528	SNX16	734209	intergenic	SNX16(di	13.39192	Down	NULL	PBS_peak_10798
peak1071	chr8	83648617	83648806	0	NR_134295		175627	NM_1528	SNX16	894190	intergenic	SNX16(di	5.61274	Down	NULL	PBS_peak_10806
peak1072	chr8	83701045	83701206	0	NR_134295		123213	NM_1528	SNX16	946604	intergenic	SNX16(di	14.10396	Down	NULL	NULL
peak1073	chr8	84012682	84012858	0	NR_1220	LINC0141	303223	NM_1528	SNX16	1258249	intergenic	LOC1019	5.69924	Down	NULL	PBS_peak_10810
peak1074	chr8	84625429	84625591	0	NM_0011	RALYL	469943	NM_1528	SNX16	1870989	intergenic	LINC0141	5.56059	Down	NULL	NULL
peak1075	chr8	84833894	84834059	0	NM_0011	RALYL	261476	NM_1528	SNX16	2079455	intergenic	LINC0141	5.13369	Down	NULL	PBS_peak_10822
peak1076	chr8	85024153	85024313	0	NM_0011	RALYL	71220	NM_1528	SNX16	2269712	intergenic	LINC0141	9.52033	Down	NULL	PBS_peak_10832
peak1077	chr8	88040730	88040882	1	NM_0038	RIPK2	2729169	NM_0190	CNGB3	284903	intronic	CNBD1	4.64198	Down	NULL	PBS_peak_10845
peak1078	chr8	89771706	89771867	0	NM_0038	RIPK2	998188	NM_0059	MMP16	432069	intergenic	MMP16(d	3.57688	Down	NULL	PBS_peak_10858
peak1079	chr8	90392439	90392615	0	NM_0038	RIPK2	377448	NM_0059	MMP16	1052810	intergenic	MMP16(d	15.13558	Down	NULL	PBS_peak_10862
peak1080	chr8	90620827	90620979	0	NM_0038	RIPK2	149072	NM_0059	MMP16	1281186	intergenic	MMP16(d	6.18134	Down	NULL	NULL
peak1081	chr8	90674609	90674767	0	NM_0038	RIPK2	95287	NM_0059	MMP16	1334971	intergenic	MMP16(d	4.47803	Down	NULL	NULL
peak1082	chr8	91442894	91443047	0	NM_0223	NECAB1	360950	NM_0049	CALB1	347861	intergenic	LINC0053	4.2818	Down	NULL	PBS_peak_10873
peak1083	chr8	91510379	91510571	0	NM_0223	NECAB1	293446	NM_0049	CALB1	415366	intergenic	LINC0053	11.74998	Down	NULL	PBS_peak_10877
peak1084	chr8	92261357	92261522	4	NR_1070	MIR8084	1780539	NR_1104	OTUD6B-	179022	UTR5	SLC26A7	3.93055	Up	NULL	NULL
peak1085	chr8	96590823	96590977	9	NM_0012	PTDSS1	683214	NM_1779	C8orf37	309438	ncRNA_in	C8orf37-A	13.14288	Down	NULL	NULL
peak1086	chr8	98666003	98666176	1	NM_0184	LAPTM4B	121719	NR_1253	LOC1019	207364	intronic	MTDH	4.60707	Down	NULL	PBS_peak_10895
peak1087	chr8	101957172	101957342	6	NR_0339	FLJ42969	107025	NM_0025	PABPC1	222942	intronic	YWHAZ	3.68064	Down	NULL	PBS_peak_10929
peak1088	chr8	113406419	113406589	3	NR_0317	MIR2053	249218	NR_1254	LINC0160	1158072	intronic	CSMD3	8.02815	Down	NULL	PBS_peak_10977
peak1089	chr8	117670754	117670906	1	NM_0323	UTP23	107912	NR_0462	LINC0053	333533	intronic	EIF3H	5.02441	Down	NULL	PBS_peak_10979
peak1090	chr8	117718477	117718649	1	NM_0323	UTP23	60179	NR_0462	LINC0053	381266	intronic	EIF3H	7.14645	Down	NULL	PBS_peak_10988
peak1091	chr8	117754123	117754291	1	NM_0323	UTP23	24535	NR_0462	LINC0053	416910	intronic	EIF3H	4.92652	Down	NULL	PBS_peak_10996
peak1092	chr8	124395617	124396045	1	NM_0012	WDYHV1	33134	NR_0378	ZHX1	108050	intronic	ATAD2	17.07383	Down	NULL	PBS_peak_11021
peak1093	chr8	124396817	124396998	1	NM_0012	WDYHV1	32057	NR_0378	ZHX1	109126	intronic	ATAD2	12.00172	Down	NULL	PBS_peak_11022
peak1094	chr8	128823638	128823822	1	NR_0316	MIR1205	149149	NR_1171	CASC11	77517	ncRNA_in	PVT1	5.47973	Down	NULL	PBS_peak_11028
peak1095	chr8	128832237	128832460	1	NR_0316	MIR1205	140530	NR_1171	CASC11	86135	ncRNA_in	PVT1	7.95003	Down	NULL	PBS_peak_11030
peak1096	chr8	128832521	128832696	1	NR_0316	MIR1205	140270	NR_1171	CASC11	86395	ncRNA_in	PVT1	21.01452	Down	NULL	PBS_peak_11030
peak1097	chr8	128834737	128834924	1	NR_0316	MIR1205	138048	NR_1171	CASC11	88617	ncRNA_in	PVT1	5.11566	Down	NULL	PBS_peak_11032
peak1098	chr8	128836063	128836393	1	NR_0316	MIR1205	136651	NR_1171	CASC11	90015	ncRNA_in	PVT1	9.34335	Down	NULL	PBS_peak_11033
peak1099	chr8	128836567	128836731	1	NR_0316	MIR1205	136230	NR_1171	CASC11	90436	ncRNA_in	PVT1	3.89873	Down	NULL	NULL
peak1100	chr8	128839970	128840508	1	NR_0316	MIR1205	132640	NR_1171	CASC11	94026	ncRNA_in	PVT1	7.87727	Down	NULL	PBS_peak_11035
peak1101	chr8	128840884	128841086	1	NR_0316	MIR1205	131894	NR_1171	CASC11	94772	ncRNA_in	PVT1	15.2201	Down	NULL	PBS_peak_11036
peak1102	chr8	128842145	128842320	1	NR_0316	MIR1205	130646	NR_1171	CASC11	96019	ncRNA_in	PVT1	7.29383	Down	NULL	PBS_peak_11037
peak1103	chr8	128846138	128846314	1	NR_0316	MIR1205	126653	NR_1171	CASC11	100013	ncRNA_in	PVT1	19.98969	Down	NULL	NULL
peak1104	chr8	128848640	128848840	1	NR_0316	MIR1205	124139	NR_1171	CASC11	102527	ncRNA_in	PVT1	5.64777	Down	NULL	PBS_peak_11038

peak1105	chr8	128849051	128849242	1	NR_03161	MIR1205	123732	NR_11710	CASC11	102933	ncRNA_in	PVT1	9.8272	Down	NULL	PBS_peak_11039
peak1106	chr8	128851942	128852152	1	NR_03161	MIR1205	120832	NR_11710	CASC11	105834	ncRNA_in	PVT1	8.86217	Down	NULL	PBS_peak_11040
peak1107	chr8	128853875	128854062	1	NR_03161	MIR1205	118910	NR_11710	CASC11	107755	ncRNA_in	PVT1	6.1527	Down	NULL	PBS_peak_11041
peak1108	chr8	128858563	128858849	1	NR_03161	MIR1205	114173	NR_11710	CASC11	112493	ncRNA_in	PVT1	5.38688	Down	NULL	PBS_peak_11044
peak1109	chr8	128858918	128859082	1	NR_03161	MIR1205	113879	NR_11710	CASC11	112787	ncRNA_in	PVT1	6.02863	Down	NULL	PBS_peak_11044
peak1110	chr8	128859672	128859983	1	NR_03161	MIR1205	113051	NR_11710	CASC11	113614	ncRNA_in	PVT1	5.29766	Down	NULL	PBS_peak_11045
peak1111	chr8	128860534	128860690	1	NR_03161	MIR1205	112267	NR_11710	CASC11	114399	ncRNA_in	PVT1	4.63527	Down	NULL	PBS_peak_11046
peak1112	chr8	128861396	128861581	1	NR_03161	MIR1205	111390	NR_11710	CASC11	115275	ncRNA_in	PVT1	6.49274	Down	NULL	PBS_peak_11048
peak1113	chr8	128861665	128861886	1	NR_03161	MIR1205	111103	NR_11710	CASC11	115562	ncRNA_in	PVT1	9.44571	Down	NULL	PBS_peak_11048
peak1114	chr8	128863861	128864043	1	NR_03161	MIR1205	108927	NR_11710	CASC11	117739	ncRNA_in	PVT1	7.70091	Down	NULL	PBS_peak_11050
peak1115	chr8	128864370	128864651	1	NR_03161	MIR1205	108368	NR_11710	CASC11	118297	ncRNA_in	PVT1	4.60858	Down	NULL	PBS_peak_11051
peak1116	chr8	128866253	128866629	1	NR_03161	MIR1205	106438	NR_11710	CASC11	120228	ncRNA_in	PVT1	19.75126	Down	NULL	PBS_peak_11052
peak1117	chr8	128868666	128868823	1	NR_03161	MIR1205	104134	NR_11710	CASC11	122531	ncRNA_in	PVT1	9.93076	Down	NULL	NULL
peak1118	chr8	128869288	128869445	1	NR_03161	MIR1205	103512	NR_11710	CASC11	123153	ncRNA_in	PVT1	7.2414	Down	NULL	NULL
peak1119	chr8	128871420	128872128	1	NR_03161	MIR1205	101105	NR_11710	CASC11	125561	ncRNA_in	PVT1	9.60189	Down	NULL	PBS_peak_11055
peak1120	chr8	128873514	128873731	1	NR_03161	MIR1205	99256	NR_11710	CASC11	127409	ncRNA_in	PVT1	5.09443	Down	NULL	PBS_peak_11056
peak1121	chr8	128874258	128874467	1	NR_03161	MIR1205	98516	NR_11710	CASC11	128149	ncRNA_in	PVT1	20.69145	Down	NULL	PBS_peak_11057
peak1122	chr8	128875539	128875703	1	NR_03161	MIR1205	97258	NR_11710	CASC11	129408	ncRNA_in	PVT1	6.77264	Down	NULL	PBS_peak_11058
peak1123	chr8	128876318	128876566	1	NR_03161	MIR1205	96437	NR_11710	CASC11	130229	ncRNA_in	PVT1	5.443	Down	NULL	PBS_peak_11059
peak1124	chr8	128877057	128877230	1	NR_03161	MIR1205	95735	NR_11710	CASC11	130930	ncRNA_in	PVT1	3.50393	Down	NULL	PBS_peak_11060
peak1125	chr8	128877333	128877504	1	NR_03161	MIR1205	95460	NR_11710	CASC11	131205	ncRNA_in	PVT1	6.75024	Down	NULL	PBS_peak_11060
peak1126	chr8	128877697	128877857	1	NR_03161	MIR1205	95102	NR_11710	CASC11	131564	ncRNA_in	PVT1	7.84004	Down	NULL	PBS_peak_11060
peak1127	chr8	128878465	128878690	1	NR_03161	MIR1205	94301	NR_11710	CASC11	132364	ncRNA_in	PVT1	4.10658	Down	NULL	PBS_peak_11061
peak1128	chr8	128879104	128879542	1	NR_03161	MIR1205	93556	NR_11710	CASC11	133110	ncRNA_in	PVT1	5.62424	Down	NULL	PBS_peak_11062
peak1129	chr8	128886667	128886901	1	NR_03161	MIR1205	86095	NR_11710	CASC11	140571	ncRNA_in	PVT1	9.05129	Down	NULL	PBS_peak_11067
peak1130	chr8	128903179	128903339	1	NR_03161	MIR1205	69620	NR_11710	CASC11	157046	ncRNA_ex	PVT1	15.59424	Down	NULL	NULL
peak1131	chr8	128903570	128903936	1	NR_03161	MIR1205	69126	NR_11710	CASC11	157540	ncRNA_in	PVT1	10.08438	Down	NULL	PBS_peak_11070
peak1132	chr8	128906021	128906576	1	NR_03161	MIR1205	66580	NR_11710	CASC11	160085	ncRNA_in	PVT1	10.85133	Down	NULL	PBS_peak_11072
peak1133	chr8	128906970	128907231	1	NR_03161	MIR1205	65778	NR_11710	CASC11	160887	ncRNA_in	PVT1	4.86904	Down	NULL	PBS_peak_11073
peak1134	chr8	128908005	128908229	1	NR_03161	MIR1205	64762	NR_11710	CASC11	161904	ncRNA_in	PVT1	7.70876	Down	NULL	PBS_peak_11074
peak1135	chr8	128908502	128908666	1	NR_03161	MIR1205	64295	NR_11710	CASC11	162371	ncRNA_in	PVT1	5.93435	Down	NULL	NULL
peak1136	chr8	128909167	128909620	1	NR_03161	MIR1205	63485	NR_11710	CASC11	163180	ncRNA_in	PVT1	5.81235	Down	NULL	PBS_peak_11075
peak1137	chr8	128911893	128912182	1	NR_03161	MIR1205	60841	NR_11710	CASC11	165824	ncRNA_in	PVT1	13.76115	Down	NULL	PBS_peak_11076
peak1138	chr8	128912972	128913178	1	NR_03161	MIR1205	59804	NR_11710	CASC11	166862	ncRNA_in	PVT1	13.9911	Down	NULL	PBS_peak_11076
peak1139	chr8	128913403	128913587	1	NR_03161	MIR1205	59384	NR_11710	CASC11	167282	ncRNA_in	PVT1	3.99767	Down	NULL	PBS_peak_11077
peak1140	chr8	128914645	128915000	1	NR_03161	MIR1205	58056	NR_11710	CASC11	168609	ncRNA_in	PVT1	5.24053	Down	NULL	PBS_peak_11078
peak1141	chr8	128915925	128916114	1	NR_03161	MIR1205	56859	NR_11710	CASC11	169806	ncRNA_in	PVT1	6.61406	Down	NULL	PBS_peak_11079
peak1142	chr8	128916604	128917002	1	NR_03161	MIR1205	56076	NR_11710	CASC11	170590	ncRNA_in	PVT1	4.59748	Down	NULL	PBS_peak_11080
peak1143	chr8	128917490	128917674	1	NR_03161	MIR1205	55297	NR_11710	CASC11	171369	ncRNA_in	PVT1	5.78574	Down	NULL	PBS_peak_11081
peak1144	chr8	128917970	128918186	1	NR_03161	MIR1205	54801	NR_11710	CASC11	171865	ncRNA_in	PVT1	6.00754	Down	NULL	PBS_peak_11082
peak1145	chr8	128918364	128918624	1	NR_03161	MIR1205	54385	NR_11710	CASC11	172281	ncRNA_in	PVT1	6.65935	Down	NULL	PBS_peak_11083
peak1146	chr8	128919113	128919301	1	NR_03161	MIR1205	53672	NR_11710	CASC11	172994	ncRNA_in	PVT1	7.79142	Down	NULL	PBS_peak_11084
peak1147	chr8	128919771	128919944	1	NR_03161	MIR1205	53021	NR_11710	CASC11	173644	ncRNA_in	PVT1	20.62853	Down	NULL	NULL
peak1148	chr8	128930346	128930611	1	NR_03161	MIR1205	42400	NR_11710	CASC11	184265	ncRNA_in	PVT1	4.50062	Down	NULL	PBS_peak_11087
peak1149	chr8	128931903	128932097	1	NR_03161	MIR1205	40879	NR_11710	CASC11	185787	ncRNA_in	PVT1	7.00421	Down	NULL	PBS_peak_11089
peak1150	chr8	128935587	128935822	1	NR_03161	MIR1205	37174	NR_11710	CASC11	189491	ncRNA_in	PVT1	4.7937	Down	NULL	PBS_peak_11090
peak1151	chr8	128937939	128938124	1	NR_03161	MIR1205	34847	NR_11710	CASC11	191818	ncRNA_in	PVT1	17.51506	Down	NULL	PBS_peak_11091
peak1152	chr8	128943361	128943585	1	NR_03161	MIR1205	29406	NR_11710	CASC11	197260	ncRNA_in	PVT1	5.69376	Down	NULL	PBS_peak_11094
peak1153	chr8	134570388	134570543	2	NR_12542	LOC10192	328273	NM_0060	NDRG1	260918	intronic	ST3GAL1	4.3598	Down	NULL	PBS_peak_11112
peak1154	chr8	136237338	136237596	0	NR_02670	LINC0159	8907	NR_02959	MIR30D	420279	intergenic	LOC10192	5.0584	Down	NULL	PBS_peak_11120
peak1155	chr8	137862651	137862823	0	NM_0174	CHRAC1	3658660	NR_02959	MIR30D	2045549	ncRNA_in	LINC0205	3.86895	Down	NULL	PBS_peak_11162
peak1156	chr8	137928301	137928484	0	NM_0174	CHRAC1	3593004	NR_02959	MIR30D	2111204	intergenic	LINC0205	13.89422	Down	NULL	PBS_peak_11164
peak1157	chr8	138087501	138087659	0	NM_0174	CHRAC1	3433817	NR_02959	MIR30D	2270392	intergenic	LINC0205	11.79503	Down	NULL	NULL
peak1158	chr8	140137819	140137970	0	NM_0174	CHRAC1	1383502	NM_1528	COL22A1	211645	intergenic	COL22A1	5.12521	Down	NULL	NULL
peak1159	chr8	140209908	140210077	0	NM_0174	CHRAC1	1311404	NM_1528	COL22A1	283743	intergenic	COL22A1	4.40665	Down	NULL	NULL
peak1160	chr8	140270352	140270521	0	NM_0174	CHRAC1	1250960	NM_1528	COL22A1	344187	intergenic	COL22A1	15.63246	Down	NULL	NULL
peak1161	chr8	140582149	140582307	0	NM_0174	CHRAC1	939169	NM_1528	COL22A1	655979	intergenic	COL22A1	3.35648	Down	NULL	PBS_peak_11275
peak1162	chr8	145502340	145502545	1	NM_0055	HSF1	12827	NR_10705	MIR7112	15749	intronic	BOP1	5.4986	Down	NULL	PBS_peak_11317
peak1163	chr8	145517686	145517841	1	NM_0012	SLC52A2	64453	NM_0152	BOP1	2643	intronic	HSF1	3.79479	Down	NULL	PBS_peak_11318
peak1164	chr9	2841367	2841544	1	NR_12158	LINC0123	340133	NR_01537	VLDLR-A	219082	intronic	PUM3	4.00276	Down	NULL	PBS_peak_11325
peak1165	chr9	6422283	6422520	2	NM_0011	KDM4C	298461	NM_0124	RANBP6	406761	intronic	UHRF2	11.78706	Down	NULL	PBS_peak_11328
peak1166	chr9	6431388	6431560	2	NM_0011	KDM4C	289389	NM_0124	RANBP6	415834	intronic	UHRF2	13.41229	Down	NULL	PBS_peak_11329
peak1167	chr9	6444796	6444955	2	NM_0011	KDM4C	275987	NM_0124	RANBP6	429235	intronic	UHRF2	8.14674	Down	NULL	NULL
peak1168	chr9	6449804	6450127	2	NM_0011	KDM4C	270897	NM_0124	RANBP6	434325	intronic	UHRF2	6.09068	Down	NULL	PBS_peak_11332
peak1169	chr9	6458975	6459129	2	NM_0011	KDM4C	261811	NM_0124	RANBP6	443412	intronic	UHRF2	5.67238	Down	NULL	NULL
peak1170	chr9	8282660	8282817	0	NR_12159	PTPRD-A	575279	NM_0334	TMEM26	482932	intergenic	TMEM26	3.79954	Down	NULL	PBS_peak_11365
peak1171	chr9	9196762	9196967	1	NR_135135		602527	NM_1303	PTPRD	462918	intronic	PTPRD	4.38621	Down	NULL	PBS_peak_11394
peak1172	chr9	9393258	9393415	1	NR_135135		406055	NM_1303	PTPRD	659390	intronic	PTPRD	9.58199	Down	NULL	NULL
peak1173	chr9	9499192	9499362	1	NR_135135		300115	NM_1303	PTPRD	765331	intronic	PTPRD	6.23834	Down	NULL	PBS_peak_11409
peak1174	chr9	9521878	9522041	1	NR_135135		277432	NM_1303	PTPRD	788013	intronic	PTPRD	5.99521	Down	NULL	PBS_peak_11411
peak1175	chr9	9568790	9568957	1	NR_135135		230518	NM_1303	PTPRD	834927	intronic	PTPRD	7.07386	Down	NULL	PBS_peak_11412
peak1176	chr9	10134143	10134294	1	NR_11069	PTPRD-A	478987	NM_1303	PTPRD	1400272	intronic	PTPRD	5.27506	Down	NULL	PBS_peak_11417
peak1177	chr9	10370077	10370265	1	NR_11069	PTPRD-A	243035	NM_1303	PTPRD	1636225	intronic	PTPRD	6.74914	Down	NULL	PBS_peak_11420
peak1178	chr9	10704587	10704768	0	NM_0005	TYRP1	1988708	NM_0028	PTPRD	91954	intergenic	PTPRD-A	5.61616	Down	NULL	PBS_peak_11424
peak1179	chr9	11490285	11490454	0	NM_0005	TYRP1	1203016	NM_0028	PTPRD	877646	intergenic	PTPRD-A	4.36665	Down	NULL	PBS_peak_11429
peak1180	chr9	12082181	12082362	0	NM_0005	TYRP1	611114	NM_0028	PTPRD	1469548	intergenic	PTPRD-A	5.18377	Down	NULL	PBS_peak_11431
peak1181	chr9	13194957	13195198	3	NR_03819	LINC0058	732892	NR_132976		222445	intronic	MPDZ	4.9694	Down	NULL	PBS_peak_11446
peak1182	chr9	13212567	13212787	3	NR_03819	LINC0058	715293	NR_132976		240045	intronic	MPDZ	4.52378	Down	NULL	PBS_peak_11447
peak1183	chr9	13421761	13421945	1	NR_03819	LINC0058	506117	NM_0038	MPDZ	142290	ncRNA_in	FLJ41200	15.47718	Down	NULL	PBS_peak_11460
peak1184	chr9	13517621	13517773	0	NR_03819	LINC0058	410273	NR_03386	FLJ41200	86369	intergenic	FLJ41200(	4.75009	Down	NULL	PBS_peak_11467
peak1185	chr9	13536650	13536805	0	NR_03819	LINC0058	391242	NR_03386	FLJ41200	105399	intergenic	FLJ41200(	6.77292	Down	NULL	NULL
peak1186	chr9	13680484	13680644	0	NR_03819	LINC0058	247406	NR_03386	FLJ41200	249236	intergenic	FLJ41200(	5.50404	Down	NULL	PBS_peak_11478
peak1187	chr9	13761149	13761336	0	NR_03819	LINC0058	166727	NR_03386	FLJ41200	329914	intergenic	FLJ41200(	5.9442	Down	NULL	PBS_peak_11492
peak1188	chr9	13845543	13845705	0	NR_03819	LINC0058	82346	NR_03386	FLJ41200	414296	intergenic	FLJ41200(	4.12059	Down	NULL	PBS_peak_11507
peak1189	chr9	14052581	14052733	0	NR_00392	LOC38970	940668	NR_03386	FLJ41200	621329	intergenic	LINC0058	4.5885	Down	NULL	PBS_peak_11533
peak1190	chr9	14092210	14092444	4	NR_00392	LOC38970	900998	NR_03386	FLJ41200	660999	intronic	NFIB	6.18995	Down	NULL	PBS_peak_11539
peak1191	chr9	14126351	14126506	4	NR_00392	LOC38970	866896	NR_03386	FLJ41200	695100	intronic	NFIB	8.29783	Down	NULL	NULL
peak1192	chr9	14456568	14456760	0	NR_00392	LOC38970	536661	NM_0011	NFIB	57682	intergenic	NFIB(dist	5.03012	Down	NULL	PBS_peak_11548
peak1193	chr9	14563727	14563902	0	NR_00392	LOC38970	429510	NM_0011	NFIB	164832	intergenic	NFIB(dist	3.66194	Down	NULL	PBS_peak_11557
peak1194	chr9	14790702	14790968	1	NR_00392	LOC38970	202490	NM_0011	FREM1	11304	intronic	FREM1	3.94712	Down	NULL	PBS_peak_11563
peak1195	chr9	14812383	14812537	1	NR_00392	LOC38970	180865	NM_0011	FREM1	32929	intronic	FREM1	27.00004	Down	NULL	PBS_peak_11567
peak1196	chr9	15723027	15723224	1	NM_0012	CNTLN	1411863	NM_001317900		212108	intronic	CCDC171	5.00252	Down	NULL	PBS_peak_11584
peak1197	chr9	15795532	15795686	1	NM_0012	CNTLN	1339380	NM_001317900		284592	intronic	CCDC171	11.50584	Down	NULL	PBS_peak_11589
peak1198	chr9	16106389	16106578	0	NM_0012	CNTLN	1028505	NM_001317900		595466	intergenic	CCDC171	4.9109	Down	NULL	PBS_peak_11608
peak1199	chr9	16392291	16392447	0	NM_0012	CNTLN	742620	NR_07347	C9orf92	116058	intergenic	NONE(dis	5.95278	Down	NULL	NULL
peak1200	chr9	17850232	17850409	0	NM_0528	ADAMTS	623758	NM_0176	BNC2	979534	intergenic	SH3GL2(	5.20388	Down	NULL	PBS_peak_11626
peak1201	chr9	17986435	17986719	0	NM_0528	ADAMTS	487502	NM_0176	BNC2	1115791	intergenic	SH3GL2(	4.05798	Down	NULL	PBS_peak_11646
peak1202	chr9	18005727	18005879	0	NM_0528	ADAMTS	468276	NM_0176	BNC2	1135017	intergenic	SH3GL2(	9.93852	Down	NULL	NULL
peak1203	chr9	18113211	18113427	0	NM_0528	ADAMTS	360760	NM_0176	BNC2	1242533	intergenic	SH3GL2(	6.38387	Down	NULL	PBS_peak_11661
peak1204	chr9	18150700	18150917	0	NM_0528	ADAMTS	323270	NM_0176	BNC2	1280022	intergenic	SH3GL2(	4.55173	Down	NULL	PBS_peak_11665
peak1205	chr9	18187145	18187303	0	NM_0528	ADAMTS	286855	NM_0176	BNC2	1316438	intergenic	SH3GL2(	9.60449	Down	NULL	PBS_peak_11670
peak1206	chr9	18465781	18465949	0	NM_0528	ADAMTS	8214	NM_0176	BNC2	1595079	intergenic	SH3GL2(	9.39232	Down	NULL	NULL
peak1207	chr9	18630407	18630578	2	NM_0065	RRAGA	418879	NM_0176	BNC2	1759706	intronic	ADAMTS	13.55802	Down	NULL	PBS_peak_11675

peak1208	chr9	19816178	19816403	0	NM_0177	FOCAD	842017	NM_0203	SLC24A2	29273	intergenic	SLC24A2(	6.3174	Down	NULL	PBS_peak_11727
peak1209	chr9	19873151	19873306	0	NM_0177	FOCAD	785079	NM_0203	SLC24A2	86211	intergenic	SLC24A2(	8.38397	Down	NULL	NULL
peak1210	chr9	19992749	19992990	0	NM_0177	FOCAD	665438	NM_0203	SLC24A2	205852	intergenic	SLC24A2(	4.84771	Down	NULL	PBS_peak_11734
peak1211	chr9	20038279	20038445	0	NM_0177	FOCAD	619946	NM_0203	SLC24A2	251345	intergenic	SLC24A2(	5.00574	Down	NULL	NULL
peak1212	chr9	20170279	20170465	0	NM_0177	FOCAD	487936	NM_0203	SLC24A2	383355	intergenic	SLC24A2(	9.87545	Down	NULL	PBS_peak_11752
peak1213	chr9	32448142	32448294	2	NR_03399	TOPORS-	102924	NR_135134		1066730	intronic	ACO1	3.69489	Up	NULL	NULL
peak1214	chr9	32449795	32450003	2	NR_03399	TOPORS-	101243	NR_135134		1068411	exonic	ACO1	11.72581	Up	luks_peak	NULL
peak1215	chr9	32451064	32451256	0	NR_03399	TOPORS-	99982	NR_135134		1069672	downstrea	ACO1	9.40472	Up	NULL	NULL
peak1216	chr9	32453419	32453570	0	NR_03399	TOPORS-	97647	NR_135134		1072006	downstrea	DDX58	11.06131	Up	NULL	NULL
peak1217	chr9	32454703	32454860	0	NR_03399	TOPORS-	96360	NR_135134		1073293	downstrea	DDX58	10.93924	Up	NULL	NULL
peak1218	chr9	32468994	32469232	1	NR_03399	TOPORS-	82029	NR_135134		1087625	intronic	DDX58	4.78868	Up	NULL	NULL
peak1219	chr9	32486101	32486313	1	NR_03399	TOPORS-	64935	NR_135134		1104719	intronic	DDX58	26.71106	Up	NULL	NULL
peak1220	chr9	32487291	32487730	1	NR_03399	TOPORS-	63631	NR_135134		1106022	exonic	DDX58	5.33948	Up	luks_peak	NULL
peak1221	chr9	32488184	32488338	1	NR_03399	TOPORS-	62881	NR_135134		1106773	exonic	DDX58	6.10585	Up	NULL	NULL
peak1222	chr9	32490172	32490355	1	NR_03399	TOPORS-	60878	NR_135134		1108775	intronic	DDX58	10.02566	Up	NULL	NULL
peak1223	chr9	32490697	32490918	1	NR_03399	TOPORS-	60334	NR_135134		1109319	intronic	DDX58	5.63082	Up	luks_peak	NULL
peak1224	chr9	32501321	32501499	1	NR_03399	TOPORS-	49732	NR_135134		1119922	intronic	DDX58	22.20733	Up	NULL	NULL
peak1225	chr9	32504075	32504263	1	NR_03399	TOPORS-	46973	NR_135134		1122681	intronic	DDX58	4.97768	Up	luks_peak	NULL
peak1226	chr9	32505951	32506108	1	NR_03399	TOPORS-	45112	NR_135134		1124541	intronic	DDX58	6.48636	Up	NULL	NULL
peak1227	chr9	32509165	32509319	1	NR_03399	TOPORS-	41900	NR_135134		1127754	intronic	DDX58	36.39346	Up	NULL	NULL
peak1228	chr9	32509480	32509715	1	NR_03399	TOPORS-	41544	NR_135134		1128109	intronic	DDX58	6.66802	Up	luks_peak	NULL
peak1229	chr9	32510160	32510371	1	NR_03399	TOPORS-	40876	NR_135134		1128777	intronic	DDX58	16.82748	Up	luks_peak	NULL
peak1230	chr9	32514055	32514216	1	NR_03399	TOPORS-	37006	NR_135134		1132647	intronic	DDX58	8.87051	Up	NULL	NULL
peak1231	chr9	32514375	32514664	1	NR_03399	TOPORS-	36622	NR_135134		1133031	intronic	DDX58	5.06297	Up	luks_peak	NULL
peak1232	chr9	32516383	32516534	1	NR_03399	TOPORS-	34683	NR_135134		1134970	intronic	DDX58	3.51783	Up	luks_peak	NULL
peak1233	chr9	32516662	32516822	1	NR_03399	TOPORS-	34400	NR_135134		1135254	intronic	DDX58	3.55504	Up	luks_peak	NULL
peak1234	chr9	33138198	33138364	1	NR_1081(	B4GALT1	28665	NM_0182	SMU1	61567	intronic	B4GALT1	4.79172	Up	luks_peak	NULL
peak1235	chr9	33138432	33138614	1	NR_1081(	B4GALT1	28423	NM_0182	SMU1	61809	intronic	B4GALT1	3.8227	Up	luks_peak	NULL
peak1236	chr9	33139335	33139490	1	NR_1081(	B4GALT1	27533	NM_0182	SMU1	62698	intronic	B4GALT1	3.73233	Up	luks_peak	NULL
peak1237	chr9	33143530	33143688	1	NR_1081(	B4GALT1	23337	NM_0182	SMU1	66895	intronic	B4GALT1	7.18477	Up	luks_peak	NULL
peak1238	chr9	34031416	34031598	2	NM_0011	UBAP1	147496	NR_00368	SNORD12	78655	intronic	UBAP2	27.7403	Down	NULL	PBS_peak_11793
peak1239	chr9	34104618	34104774	1	NM_0011	UBAP1	74307	NM_0184	UBAP2	55749	intronic	DCAF12	4.79432	Down	NULL	NULL
peak1240	chr9	34113188	34113566	1	NM_0011	UBAP1	65626	NM_0184	UBAP2	64430	intronic	DCAF12	5.22482	Down	NULL	PBS_peak_11797
peak1241	chr9	34117274	34117450	1	NM_0011	UBAP1	61641	NM_0184	UBAP2	68415	intronic	DCAF12	16.03503	Down	NULL	NULL
peak1242	chr9	34119322	34119501	1	NM_0011	UBAP1	59591	NM_0184	UBAP2	70464	intronic	DCAF12	6.26509	Down	NULL	PBS_peak_11803
peak1243	chr9	34186912	34187090	6	NM_0011	NUDT2	142503	NM_0153	DCAF12	60230	intronic	UBAP1	7.7665	Down	NULL	PBS_peak_11809
peak1244	chr9	36980695	36980931	13	NR_03659	EBLN3	99080	NR_03968	MIR4476	87285	intronic	PAX5	6.27056	Up	NULL	NULL
peak1245	chr9	86299862	86300029	2	NM_0249	RMI1	295691	NM_1749	FRMD3	146597	intronic	UBQLN1	5.17638	Down	NULL	NULL
peak1246	chr9	86305287	86305567	2	NM_0249	RMI1	290210	NM_1749	FRMD3	152079	intronic	UBQLN1	4.27514	Down	NULL	PBS_peak_11849
peak1247	chr9	86309691	86309864	2	NM_0249	RMI1	285859	NM_1749	FRMD3	156429	intronic	UBQLN1	4.97088	Down	NULL	PBS_peak_11851
peak1248	chr9	91016870	91017052	1	NM_0011	NXNL2	133055	NM_0011	SPATA31	267061	intronic	SPIN1	11.47099	Down	NULL	PBS_peak_11855
peak1249	chr9	93570936	93571126	3	NM_0011	SYK	18671	NM_0175	DIRAS2	165644	intronic	SYK	8.27171	Down	NULL	PBS_peak_11857
peak1250	chr9	93571660	93571847	3	NM_0011	SYK	17948	NM_0175	DIRAS2	166366	intronic	SYK	6.08479	Down	NULL	PBS_peak_11858
peak1251	chr9	93574698	93574932	3	NM_0011	SYK	14887	NM_0175	DIRAS2	169428	intronic	SYK	4.47006	Down	NULL	PBS_peak_11859
peak1252	chr9	93602468	93602627	4	NR_135306		278796	NM_0175	DIRAS2	197160	intronic	SYK	4.77353	Down	NULL	PBS_peak_11867
peak1253	chr9	95523030	95523258	2	NR_0268(	ANKRD19	48749	NM_0227	IPPK	90597	intronic	BICD2	8.84713	Down	NULL	PBS_peak_11871
peak1254	chr9	95887286	95887439	1	NM_0012	WNK2	59849	NR_1215(	LOC1019	110136	exonic	NINJ1	5.16292	Up	NULL	NULL
peak1255	chr9	95888840	95889002	1	NM_0012	WNK2	58291	NR_1215(	LOC1019	111695	exonic	NINJ1	24.48628	Up	NULL	NULL
peak1256	chr9	96269866	96270065	4	NM_0053	PHF2	68943	NM_1988	FAM120A	54091	intronic	FAM120A	4.26383	Down	NULL	PBS_peak_11874
peak1257	chr9	99171933	99172115	1	NM_0142	HABP4	40413	NM_0070	SLC35D2	26032	intronic	ZNF367	29.7282	Down	NULL	PBS_peak_11878
peak1258	chr9	99752668	99752820	1	NR_03652	LOC1004	247964	NM_0010	ZNF782	136355	ncRNA_in	MFSD14C	17.4155	Down	NULL	PBS_peak_11879
peak1259	chr9	100190280	100190447	2	NM_0011	TMOD1	73098	NR_02684	LOC2863	31390	intronic	TDRD7	4.81675	Up	NULL	NULL
peak1260	chr9	100751687	100752071	1	NM_0189	NANS	67080	NM_0184	HEMGN	44682	intronic	ANP32B	15.04994	Down	NULL	PBS_peak_11881
peak1261	chr9	100753463	100753652	1	NM_0189	NANS	65401	NM_0184	HEMGN	46360	intronic	ANP32B	6.64164	Down	NULL	PBS_peak_11884
peak1262	chr9	100864409	100864569	2	NM_0246	GALNT12	705492	NM_0184	HEMGN	157292	intronic	TRIM14	9.34511	Up	NULL	NULL
peak1263	chr9	102853462	102853678	1	NM_001318381		7897	NR_03885	STX17-AS	184687	intronic	ERP44	4.70567	Down	NULL	PBS_peak_11888
peak1264	chr9	107657065	107657245	1	NM_0012	SLC44A1	349739	NM_0010	OR13C9	276670	intronic	ABCA1	3.49555	Down	NULL	PBS_peak_11890
peak1265	chr9	110053895	110054076	3	NM_0066	ACTL7A	1570522	NR_12602	LOC3405	188716	intronic	RAD23B	4.61739	Down	NULL	PBS_peak_11900
peak1266	chr9	110054638	110054848	3	NM_0066	ACTL7A	1569765	NR_12602	LOC3405	189474	intronic	RAD23B	27.58181	Down	NULL	PBS_peak_11901
peak1267	chr9	110055845	110056034	3	NM_0066	ACTL7A	1568568	NR_12602	LOC3405	190670	intronic	RAD23B	7.12943	Down	NULL	PBS_peak_11902
peak1268	chr9	118244022	118244180	0	NM_0025	PAPPA	671970	NR_11095	LOC1019	343413	intergenic	DEC1(dist	4.22631	Down	NULL	NULL
peak1269	chr9	118571410	118571602	0	NM_0025	PAPPA	344565	NR_1098(	LOC1019	64988	intergenic	LOC1019	10.07197	Down	NULL	PBS_peak_11915
peak1270	chr9	118987990	118988175	1	NR_03397	ASTN2-A	278479	NR_02403	LINC0047	300705	intronic	PAPPA	5.80255	Down	NULL	NULL
peak1271	chr9	121618907	121619107	0	NR_132393		1078331	NM_0140	ASTN2	1441690	intergenic	TLR4(dist	3.80705	Down	NULL	PBS_peak_11926
peak1272	chr9	122430119	122430308	0	NR_132393		267124	NM_0146	BRINP1	298474	intergenic	BRINP1(d	4.46095	Down	NULL	PBS_peak_11929
peak1273	chr9	127040038	127040215	4	NM_0011	NEK6	14123	NR_135129		268451	intronic	NEK6	5.85944	Down	NULL	PBS_peak_11943
peak1274	chr9	129593339	129593721	2	NM_0010	ZBTB34	29414	NR_0450(	NRON	420747	intronic	ZBTB43	4.17194	Up	luks_peak	NULL
peak1275	chr9	132394667	132394854	10	NM_0163	PRRX2	33159	NM_1993	C9orf50	11705	intronic	NTMT1	12.38685	Down	NULL	PBS_peak_11957
peak1276	chr9	136836773	136836936	2	NR_01542	LINC0009	53706	NM_0071	SARDH	231777	intronic	VAV2	4.05906	Down	NULL	PBS_peak_11988
peak1277	chr9	137274708	137274863	2	NM_0012	RXRA	23642	NR_02334	RNU6ATA	245099	intronic	RXRA	4.52521	Down	NULL	PBS_peak_12005
peak1278	chr9	137281606	137281784	2	NM_0012	RXRA	16733	NR_02334	RNU6ATA	252009	intronic	RXRA	3.95369	Down	NULL	PBS_peak_12008
peak1279	chr9	137283344	137283505	2	NM_0012	RXRA	15003	NR_02334	RNU6ATA	253738	intronic	RXRA	10.85065	Down	NULL	PBS_peak_12010
peak1280	chr9	138949166	138949324	1	NM_0155	GPSM1	272687	NM_0161	UBAC1	96019	intronic	NACC2	4.53129	Down	NULL	NULL
peak1281	chr9	140526135	140526305	2	NR_02434	EHMT1-IT	131254	NR_12203	ARRDC1-	12873	intronic	EHMT1	6.65151	Down	NULL	PBS_peak_12045
peak1282	chr9	140531108	140531266	2	NR_02434	EHMT1-IT	126287	NR_12203	ARRDC1-	17840	intronic	EHMT1	4.87787	Down	NULL	PBS_peak_12048
peak1283	chr9	140531330	140531514	2	NR_02434	EHMT1-IT	126052	NR_12203	ARRDC1-	18075	intronic	EHMT1	19.22341	Down	NULL	PBS_peak_12048
peak1284	chr9	140531661	140531971	2	NR_02434	EHMT1-IT	125658	NR_12203	ARRDC1-	18469	intronic	EHMT1	5.14282	Down	NULL	PBS_peak_12048
peak1285	chr9	140532746	140532968	2	NR_02434	EHMT1-IT	124617	NR_12203	ARRDC1-	19510	intronic	EHMT1	5.63107	Down	NULL	PBS_peak_12049
peak1286	chr9	140539409	140539625	2	NR_02434	EHMT1-IT	117957	NR_12203	ARRDC1-	26170	intronic	EHMT1	5.72444	Down	NULL	PBS_peak_12052
peak1287	chr9	140581505	140581691	2	NR_02434	EHMT1-IT	75876	NR_12203	ARRDC1-	68251	intronic	EHMT1	8.99676	Down	NULL	PBS_peak_12054
peak1288	chr9	140583186	140583340	2	NR_02434	EHMT1-IT	74211	NR_12203	ARRDC1-	69916	intronic	EHMT1	5.34502	Down	NULL	NULL
